# Global, regional, and national burdens of common micronutrient deficiencies from 1990 to 2019: A secondary trend analysis based on the Global Burden of Disease 2019 study

**DOI:** 10.1016/j.eclinm.2022.101299

**Published:** 2022-02-12

**Authors:** Xu Han, Shuangning Ding, Jinxin Lu, Yongze Li

**Affiliations:** a Department of Obstetrics, The First Affiliated Hospital of China Medical University, 155 Nanjing Bei Street, Shenyang, 110001, Liaoning, China; b Department of Endocrinology and Metabolism, Institute of Endocrinology, NHC Key Laboratory of Diagnosis and Treatment of Thyroid Disease, The First Affiliated Hospital of China Medical University, 155 Nanjing Bei Street, Shenyang, 110001, Liaoning, China; c Department of Clinical Nutrition, The First Affiliated Hospital of China Medical University, 155 Nanjing Bei Street, Shenyang, 110001, Liaoning, China

## Abstract

**Background:**

Understanding micronutrient deficiency burdens and trends can help guide effective intervention strategies. This study aims to elucidate trends in common micronutrient deficiencies, in particular, dietary iron, iodine and vitamin A deficiencies, from 1990 to 2019 using Global Burden of Disease (GBD) 2019 study data.

**Methods:**

We analyzed data from the GBD 2019 study to calculate the prevalence, incidence, and disability-adjusted life year (DALY) rates of micronutrient deficiencies in geographic populations worldwide from 1990 to 2019. The estimated annual percentage changes (EAPCs) and age-standardized rates were calculated to evaluate the temporal trends.

**Findings:**

Globally, the age-standardized prevalence rates of iodine deficiency, vitamin A deficiency, and dietary iron deficiency decreased, with EAPCs of -0.690 (95% CI, -0.842 to -0.538), -3.15 (95% CI, -3.20 to -3.02), and -0.546 (95% CI, -0.585 to -0.507) between 1999 and 2019, respectively. Regarding the sociodemographic index (SDI), the highest age-standardized prevalence, incidence, and DALY rates of micronutrient deficiency were found in low-SDI countries in 2019. There were linear associations between the SDI and the healthcare access and quality (HAQ) index and age-standardized prevalence, incidence, and DALY rates.

**Interpretation:**

Global micronutrient deficiency burdens have decreased since 1990. The potential burden of iodine deficiency in some developed countries is worthy of attention. The results of this study could guide policy makers in implementing cost-effective interventions to reduce micronutrient deficiency burdens, particularly in low-SDI and low-HAQ index countries.

**Funding:**

This work was supported by the National Natural Science Foundation of China (Grant No. 82000753) and the China Postdoctoral Science Foundation (Grant No. 2021MD703910).

**Research in context**

**Evidence before this study**

We searched for the key words “micronutrient deficiency”, “iodine deficiency”, “vitamin A deficiency”, “iron deficiency”, “prevalence”, “incidence”, “DALY”, and “epidemiology” in PubMed, Science Direct, and Web of Science to identify studies and reports on micronutrient deficiency published in English through Feb 1, 2021. Previous studies on micronutrient deficiencies have mainly focused on pregnant women and children. In addition, no study has provided detailed estimates of micronutrient deficiency prevalence, incidence, and DALYs in 204 countries and territories worldwide.

**Added value of this study**

We analyzed data from the Global Burden of Disease (GBD) 2019 study to provide up-to-date estimates of a wide range of health measures related to micronutrient deficiencies at global, regional, and national levels for 204 countries and territories as well as by age, sex, and sociodemographic index (SDI). We reported the detailed burdens of micronutrient deficiencies, including iodine deficiency, vitamin A deficiency, and dietary iron deficiency, and their associations with SDI and health access quality (HAQ) index.

**Implications of all the available evidence**

Our research shows that the global burdens of micronutrient deficiencies have decreased since 1990. However, the burdens of iodine deficiency and vitamin A deficiency remain high in Central Sub-Saharan Africa, and that of dietary iron deficiency remains high in South Asia. The potential burden of iodine deficiency in some developed countries is worthy of attention. The results of this study could guide policy makers in implementing cost-effective interventions to reduce the burdens of micronutrient deficiencies, particularly in low-SDI and low-HAQ index countries.

Alt-text: Unlabelled box

## Introduction

Micronutrient deficiencies are important contributors to the global burden of disease as contributors to increased rates of morbidity and mortality. Globally, an estimated one-third of people suffer from at least one form of micronutrient deficiency.^1^ The 2020 Global Nutrition Report highlighted dramatic inequities in the burden of micronutrient deficiencies.^2^ This burden exists worldwide and particularly affects children and pregnant women. Common micronutrient deficiencies, including iron, vitamin A, iodine, folate, and zinc deficiencies, contribute to severe and even life-threatening conditions.^2^^,^^3^

Iron deficiency is the most common micronutrient deficiency, affecting more than one-third of the population worldwide and leading to microcytic anemia, causing fatigue, weakness, shortage of breath and dizziness.^4^, ^5^, ^6^, ^7^ A report by the World Health Organization (WHO) indicated that more than 40% of children and pregnant women suffered from anemia in 2016, and a major portion of this was due to microcytic anemia.^8^^,^^9^ Vitamin A deficiency is the leading cause of preventable blindness in children and increases the risk of severe infections, such as diarrheal diseases and measles.^10^^,^^11^ The WHO estimated that more than 250 million children develop blindness due to vitamin A deficiency, and half of them die within one year of going blind.^12^ Iodine deficiency is a leading cause of preventable brain damage in childhood. An estimated two billion people globally have an inadequate iodine status, which has negative impacts on a country's overall health and productivity and hinders its socioeconomic development.^13^

Many of these deficiencies could be prevented through nutrition education and a healthy diet containing diverse foods, as well as food fortification and supplementation, such as universal salt iodization, vitamin A supplementation in children, and iron/folate supplementation in pregnant women, and the establishment of programs such as the Infant and Young Child Feeding program.^14^ However, micronutrient deficiencies remain a major public health problem worldwide. Identifying the current burden and trends of micronutrient deficiencies is critical to understanding their status under various conditions and developing intervention strategies. Previous studies on micronutrient deficiencies have mainly focused on subpopulations or been confined to local areas.^15^^,^^16^ Only one study has reported that substantial proportions of global disease burden are attributable to the three micronutrient deficiencies at the global and regional levels, using data from the Global Burden of Disease (GBD) Study 2000, but country-level information was not reported.^17^ In addition, sociodemographic development, economic transformation, and risk exposure have undergone significant changes in the past two decades worldwide. Therefore, the estimated overall micronutrient burden needs to be updated from 2000 estimates. A recent study using data from the GBD 2019 database reported disability-adjusted life-years (DALYs) of dietary iron deficiency globally from 1990 to 2019.^18^ This study did not analyze the burden of micronutrient deficiencies in further detail, although the Global Health Data Exchange GBD Results Tool provided data for each country in the database. A report by the High Level Panel of Experts highlighted three greatest public health concerns: vitamin A, iron and iodine.^1^ In addition, other nutritional deficiencies (such as vitamin B12, vitamin D, and folate deficiency) were classified as a single category in the GBD 2019, given their relatively limited burden, diversity in underlying causes and risk factors, and data availability.^18^ Moreover, no study has quantified the annual trend in the burden of micronutrient deficiencies such as iodine deficiency and vitamin A deficiency over a specified time period. Therefore, for the first time, we examined data from the GBD 2019 to determine the global, regional, and national prevalence of dietary iron deficiency, iodine deficiency, and vitamin A deficiency as well as the incidence and DALYs in terms of age-standardized rates from 1990 to 2019 by age, sex, and sociodemographic index (SDI) to provide a comprehensive and comparable analysis of micronutrient deficiency burdens.

## Methods

### Data sources

Data on micronutrient deficiency burdens in 204 countries and territories from 1990 to 2019 were obtained from the Global Health Data Exchange GBD Results Tool (http://ghdx.healthdata.org/gbd-results-tool) (date of data extraction, January 20, 2021). The GBD 2019 included all available up-to-date sources of epidemiological data and improved standardized methods to provide a comprehensive assessment of health loss considering 369 diseases and injuries and 87 risk factors in 204 countries and territories.^18^^,^^19^ Previous studies have described the general methods of the GBD 2019, including its main changes compared with previous years.^18^^,^^19^ In brief, the GBD 2019 study used standardized tools to model processed data to estimate each variable of interest based on age, sex, location, and year. Three main standardized tools were used, including an integrated model of cause of death (CODEm), spatiotemporal Gaussian process regression (ST-GPR) and DisMod-MR. CODEm is a highly systematized tool for analyzing cause of death data using an ensemble of different modeling methods for rates or cause fractions with varying covariate options that perform best with out-of-sample predictive validity testing. DisMod-MR is a Bayesian meta-regression tool that allows the evaluation of all available data on incidence, prevalence, remission, and mortality for a disease, enforcing consistency between epidemiological parameters. ST-GPR is a set of regression methods that borrow strength between locations and over time for single metrics of interest, such as risk factor exposures or mortality rates.^18^^,^^19^ The GBD 2019 used a variety of relevant indicators to measure population health loss, including the number of deaths and the mortality rate, the number of cases and the prevalence rate, years of life lost (YLLs) due to premature death, years of life lived with disability (YLDs), and DALYs. Uncertainty intervals (UIs) were calculated from 1000 draw-level estimates for each parameter, and 95% UIs were defined by the 25^th^ and 975^th^ values of the ordered 1,000 estimates; a 95% UI excluding 0 was considered to be statistically significant. The SDI is a comprehensive indicator of the development status of a geographic location. In the GBD 2019 study, the SDI was calculated based on income per capita, average educational attainment, and fertility rate.^20^ The SDI ranges from 0 to 1, where 0 represents the fewest years of education, the lowest per capita income, and the highest fertility rate, and vice versa. The geometric mean of these values for each location-year was recorded, and the 204 countries and territories were divided into 5 groups according to SDI quintile (low-SDI, low-middle-SDI, middle-SDI, high-middle-SDI, and high-SDI quintiles).^20^ The health care accessibility and quality (HAQ) index is an indicator of the performance of health systems in all areas in the GBD study, and it is calculated based on amenable mortality.^21^ The HAQ index ranges from 0 (worst) to 100 (best). This study does not contain personal or medical information about an identifiable living individual, and animal subjects were not involved. The institutional review board of the First Hospital of China Medical University determined that the study did not need approval because it used publicly available data.

### Case definitions

In GBD 2019, the assessment of vitamin A deficiency burden involved the quantification of total vitamin A deficiency (serum retinol < 0.7 µmol/L) as well as blindness and vision loss due to vitamin A deficiency, which are associated with corneal ulcerations and corneal scars.^18^ Dietary iron deficiency in the GBD cause analysis was defined as inadequate iron to meet the body's needs due to inadequate dietary intake of iron but not due to other causes of absolute or functional iron deficiency.^18^ The nonfatal iodine deficiency burden includes estimates of only iodine deficiency associated with visible goiter (grade 2) and its associated sequelae, including thyroid dysfunction, heart failure, and intellectual disability. It does not include estimates of subclinical iodine deficiency or nonvisible goiter (grade 1) induced by iodine deficiency.^18^ More details on the prevalence, incidence, and DALY estimation process are provided in the **Supplementary Methods**.

### Data analysis

To characterize micronutrient deficiency burdens by age, sex, year, and location, descriptive analyses were conducted. The age-standardized rates and their 95% UIs were directly downloaded from the IHME website. The age-standardized prevalence, incidence, and age-standardized DALY rate per 100,000 population in both sexes combined were obtained and compared at the global, regional, and national levels. Furthermore, we calculated their estimated annual percentage changes (EAPCs) to assess the secular trends using linear regression analysis. Finally, we examined the shape of the associations of the age-standardized prevalence and DALY rates of micronutrient deficiencies with the SDI and HAQ index using smoothing spline models. All statistical analyses were performed using GraphPad Prism (version 8.0), RStudio software (version 1.4.1106) and Joinpoint Regression Program (version 4.9.0.0).

#### Role of the funding source

The funding sources had no role in the study design, data analysis, interpretation, or decision to submit for publication. All authors had full access to all the data in the study and accepted responsibility to submit for publication.

## Results

### Iodine deficiency

**Figure 1:**
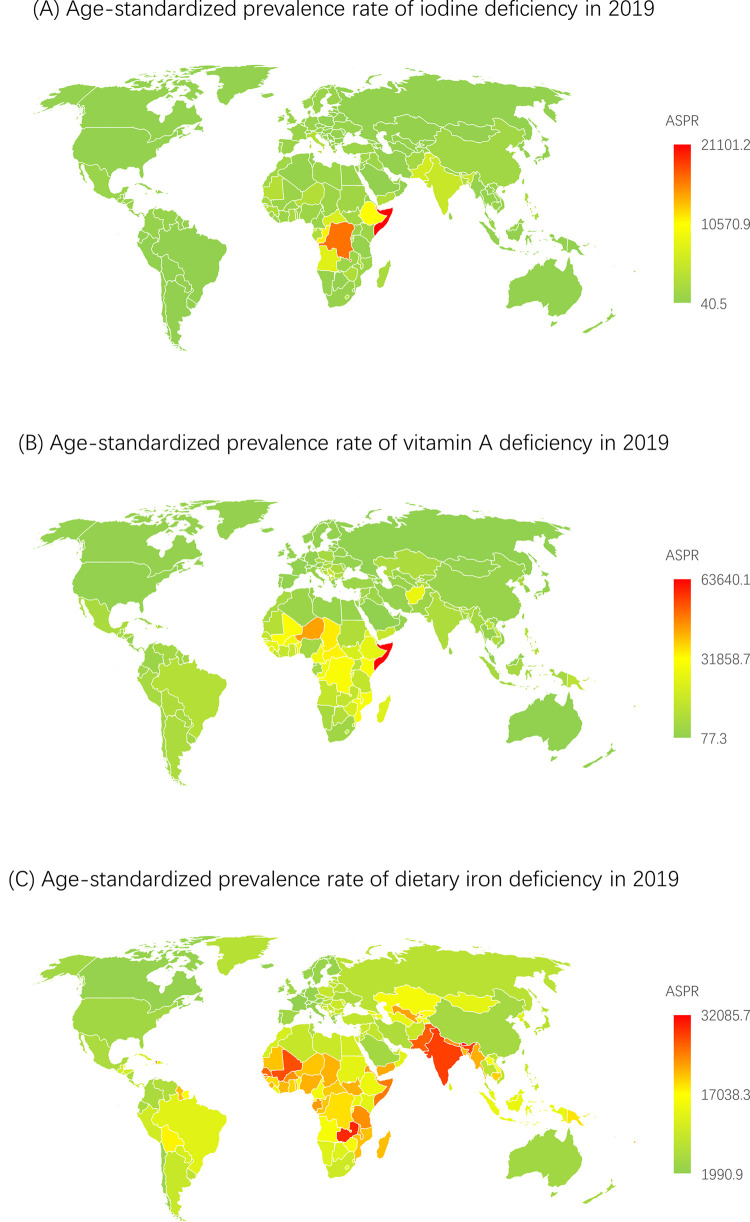
Global age-standardized prevalence rates of iodine deficiency, vitamin A deficiency, and dietary iron deficiency in 2019. Note: ASPR, age-standardized prevalence rate.

**Figure 2:**
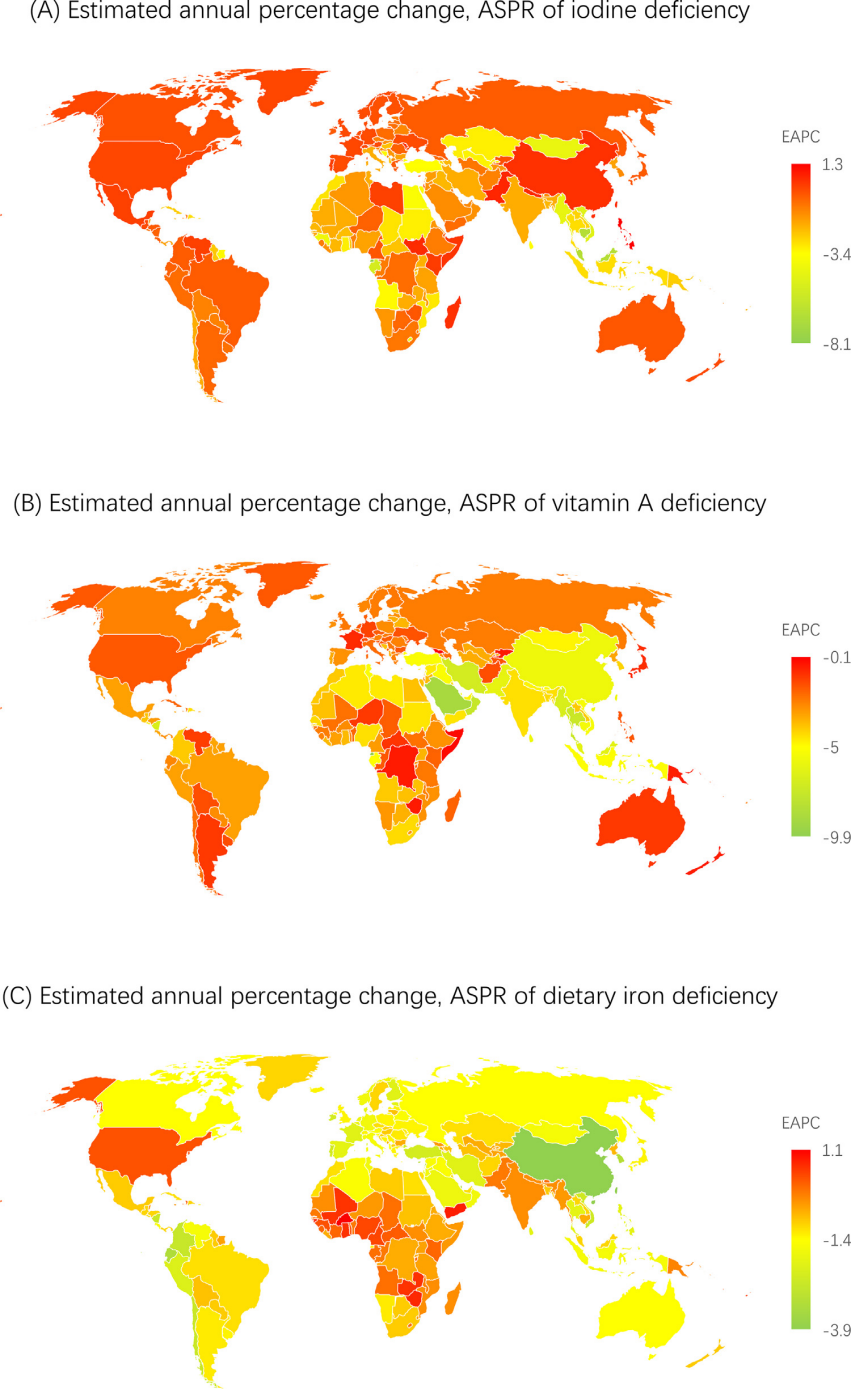
The estimated annual percentage changes (APCs) of the age-standardized prevalence rate worldwide from 1990 to 2019. Note: EAPC, estimated annual percentage change.

The global age-standardized prevalence rate of iodine deficiency was 2218 (95% UI, 1801-2736) and 2216 (1804-2743) per 100,000 population in 2018 and in 2019, respectively (**Supplementary Table 1**). The global age-standardized prevalence rate decreased, with an EAPC of -0.690 (95% CI, -0.842 to -0.538) from 1990 to 2019 (Table 1 and **Supplementary Table 2**). At the country level, the age-standardized prevalence rate of iodine deficiency per 100,000 population was highest in Somalia (21101 [17356-24960]), followed by the Democratic Republic of the Congo (16385 [13383-19590]), Djibouti (13295 [10704-16391]) and Republic of the Congo (11189 [9051-13535]) in 2019 (Figure 1 **and Supplementary Table 3**). In addition, the age-standardized prevalence rate showed an upward trend in the Philippines, Pakistan, South Sudan, Madagascar, Somalia, Andorra, Portugal, Mexico, Monaco, and San Marino (Figure 2 **and Supplementary Table 2**). Geographically, the age-standardized prevalence rates per 100,000 population were highest in Central sub-Saharan Africa (13484 [11017-16198]) and South Asia (5107 [4102-6327]) in 2019 (**Supplementary Table 4**). The age-standardized prevalence rate was higher in females than in males from 1990 to 2019 (**Supplementary Figure 1**). Moreover, the highest prevalence rate in 2019 was observed in individuals aged 30-34 years (**Supplementary Figure 4 and Supplementary Table 5**) as well as in low- and low-middle-SDI countries; it was lower in high- and high-middle-SDI countries than in other SDI countries in 2019 (**Supplementary Figure 2 and Supplementary Table 6**). The trends of the age-standardized incidence and DALY rates were relatively similar to that of the age-standardized prevalence rate (**Supplementary Figures 1-4 and Supplementary Tables 1-6**).

**Table 1:** Age-standardized prevalence rate (ASPR) for iodine deficiency, vitamin A deficiency and dietary iron deficiency in 1990 and 2019 for both sexes and estimated annual percentage changes (EAPCs) by Global Burden of Disease (GBD) region.

	Iodine deficiency			Vitamin A deficiency			Dietary iron deficiency		
	ASPR in 1990	ASPR in 2019	EAPC between 1990 and 2019	ASPR in 1990	ASPR in 2019	EAPC between 1990 and 2019	ASPR in 1990	ASPR in 2019	EAPC between 1990 and 2019
Global	2833.7 (2365.1-3461)	2215.5 (1803.8-2743.3)	-0.9 (-0.9 to -0.8)*	17323.2 (16526.5-18138.9)	6955.6 (6645.9-7294.2)	-3.1 (-3.3 to -2.9)*	16252.6 (15948.8-16509.4)	14106.4 (13850.7-14342.1)	-0.5 (-0.5 to -0.5)*
Andean Latin America	91 (68.4-118.5)	77.5 (57.3-103.2)	-0.5 (-0.6 to -0.5)*	12877.7 (11631.9-14260.4)	5904.7 (5259.4-6633.2)	-2.6 (-2.7 to -2.5)*	17306.5 (16286.3-18312.8)	10368.2 (9586.5-11205.9)	-1.8 (-1.8 to -1.7)*
Australasia	221.6 (169.5-281.3)	208.3 (158.6-264.3)	-0.2 (-0.2 to -0.2)*	237.4 (210.4-270)	148.8 (131.4-168.8)	-1.6 (-1.9 to -1.3)*	6179.5 (5174.3-7322.1)	4303.7 (3443.2-5345.6)	-1.2 (-1.3 to -1.2)*
Caribbean	735.1 (580.4-915.8)	555 (421.8-702.3)	-1.0 (-1.1 to -0.9)*	11146.7 (10301.1-11991.6)	6289.7 (5659.2-6996.4)	-2.0 (-2.0 to -1.9)*	17334.8 (16464.8-18223.9)	15877 (15015.8-16782.6)	-0.3 (-0.3 to -0.3)*
Central Asia	752.8 (593.8-936.6)	454.1 (349.6-578.4)	-1.7 (-1.9 to -1.6)*	8840.3 (8009.4-9737.2)	4272 (3883.1-4669.6)	-2.5 (-2.8 to -2.3)*	20422.8 (19625.2-21217.8)	17627.8 (16752.3-18562.8)	-0.5 (-0.5 to -0.5)*
Central Europe	302 (242.9-370)	220.7 (167.7-281)	-1.1 (-1.1 to -1.0)*	15274 (14477.1-16139.9)	7477.4 (7089.5-7916.5)	-2.4 (-2.6 to -2.3)*	11950.5 (11259.2-12685.1)	8550.7 (7934-9256.4)	-1.1 (-1.2 to -1.1)*
Central Latin America	692 (539.4-873.2)	694 (541.9-873.3)	0 (0 to 0.1)	14629.6 (13257.7-16046.8)	5717.6 (5096.1-6419.5)	-3.2 (-3.3 to -3.1)*	9540.4 (9192.8-9930.1)	6311.3 (6030.1-6616.3)	-1.4 (-1.4 to -1.4)*
Central sub-Saharan Africa	19019.9 (17452.7-20550.1)	13484.2 (11016.8-16197.7)	-1.2 (-1.3 to -1.1)*	43280.4 (39834.2-46536.7)	25905.2 (23288.4-28883)	-1.7 (-1.9 to -1.6)*	20607.6 (19300.2-21901.5)	18337.6 (17021.8-19462.4)	-0.4 (-0.5 to -0.4)*
East Asia	1268.1 (991.7-1648.7)	1402.9 (1121.6-1762.1)	0.3 (0.2 to 0.4)*	11210.8 (9382.3-13316.5)	2183.6 (1847.1-2605)	-5.5 (-5.8 to -5.3)*	12429.1 (11883.4-12972.4)	4724.1 (4350.7-5109.8)	-3.3 (-3.4 to -3.2)*
Eastern Europe	245.7 (186.7-310.9)	257.1 (197.2-325.5)	0.2 (-0.1 to 0.4)	1071.3 (979.5-1179.1)	530.9 (481.6-587)	-2.4 (-2.5 to -2.2)*	9525.7 (8723-10419.4)	7084.6 (6337.3-7948.8)	-1.0 (-1.1 to -1.0)*
Eastern sub-Saharan Africa	6489.1 (5316.3-7837.1)	4683.3 (3835-5727)	-1.2 (-1.3 to -1.1)*	46770.6 (45268.6-48255.9)	23500 (22337.2-24765.1)	-2.4 (-2.4 to -2.3)*	20122.6 (19655.5-20607.1)	18343.4 (17864.7-18830.3)	-0.3 (-0.3 to -0.3)*
High-income Asia Pacific	306.2 (235.3-389.7)	253.5 (193.8-320.5)	-0.6 (-0.7 to -0.6)*	1376.9 (1194.4-1589.9)	683.9 (599-780)	-2.4 (-2.5 to -2.2)*	12702.7 (11730.7-13697.8)	7590.7 (6761.2-8559.3)	-1.8 (-1.8 to -1.7)*
High-income North America	231.5 (177.6-294.9)	229.2 (174.5-291.8)	0 (-0.1 to 0)	811 (703.8-924.9)	485.6 (408.1-574)	-1.7 (-1.9 to -1.6)*	4126 (3714.9-4571.5)	4028 (3504.7-4598.9)	-0.1 (-0.1 to -0.1)*
North Africa and Middle East	1602.5 (1287.4-1943.5)	942.9 (752.5-1153.7)	-1.8 (-1.9 to -1.7)*	15427.7 (14749.2-16089.9)	5249.9 (4905.9-5602.5)	-3.7 (-3.8 to -3.5)*	14110.7 (13567.2-14688.3)	9644 (9162-10143.7)	-1.3 (-1.3 to -1.3)*
Oceania	128.6 (98-169.2)	75.2 (55.6-99.4)	-1.8 (-1.9 to -1.8)*	19889.7 (18034.3-21935.5)	13011.6 (11381.7-14879.8)	-1.5 (-1.6 to -1.3)*	20099.1 (18829-21265.2)	19034 (17674.2-20451.2)	-0.2 (-0.2 to -0.2)*
South Asia	8888.8 (7387.3-10821.8)	5107.5 (4102.3-6327)	-1.9 (-2.0 to -1.8)*	27177.4 (24364.6-30028.7)	7189.3 (6181.3-8389)	-4.5 (-4.8 to -4.1)*	30096.3 (29477.2-30625.4)	27403.7 (26798.8-27971.2)	-0.3 (-0.3 to -0.3)*
Southeast Asia	1549.6 (1281.3-1880.4)	771.5 (610.7-970.1)	-2.4 (-2.4 to -2.3)*	21792.8 (20300.6-23360.3)	5175.3 (4668-5733.8)	-4.8 (-5.0 to -4.7)*	19070.9 (18348.3-19791.3)	12636.6 (12040.5-13272.2)	-1.4 (-1.5 to -1.4)*
Southern Latin America	182.1 (136.5-228.2)	139.4 (101.8-179.9)	-0.9 (-0.9 to -0.9)*	10805.2 (9611.4-12213.7)	6672.8 (5811.8-7650.3)	-1.7 (-1.9 to -1.4)*	11217.9 (10273.6-12214.5)	7634.3 (6656.8-8776.8)	-1.3 (-1.4 to -1.3)*
Southern sub-Saharan Africa	1839.8 (1679.6-2025.5)	1058.6 (836.9-1340.1)	-1.9 (-1.9 to -1.8)*	18153.7 (16488.6-20003.7)	7834.6 (7001-8690.2)	-2.9 (-3.0 to -2.8)*	13674.1 (12694.4-14683.7)	11571.6 (10872.7-12347.4)	-0.6 (-0.6 to -0.5)*
Tropical Latin America	116 (87.1-151.7)	105.1 (78.7-138.4)	-0.3 (-0.4 to -0.3)*	24605.7 (22010.2-27545.3)	10005.4 (8592.7-11600.5)	-3.1 (-3.1 to -3.0)*	17514.1 (15954.6-19027)	12778.6 (11440.2-14081)	-1.1 (-1.1 to -1.1)*
Western Europe	1395.6 (1113.2-1749.6)	1103.1 (876.6-1397.1)	-0.8 (-0.9 to -0.7)*	1408.6 (1320.3-1508.6)	683.2 (637.3-735.6)	-2.5 (-2.6 to -2.4)*	4665.9 (4327.6-5043.9)	2903.6 (2639.2-3185.2)	-1.6 (-1.6 to -1.6)*
Western sub-Saharan Africa	2229.2 (1833.3-2734.7)	1421.7 (1114.8-1818.1)	-1.6 (-1.7 to -1.4)*	36703.6 (35417.5-38048.5)	15570.9 (14825.2-16315.9)	-2.9 (-3.1 to -2.8)*	19408.9 (18539.8-20307.1)	21258.6 (20245.4-22233.4)	0.3 (0.3 to 0.3)*

Note: *indicates a *P-value* less than 0.05.

### Vitamin A deficiency

The age-standardized prevalence rate per 100,000 population at the global level was 7168 (6852-7512) in 2018 and 6956 (6646-7294) in 2019 (**Supplementary Table 1**). The global age-standardized prevalence rate decreased from 1990 to 2019, with an EAPC of -3.15 (95% CI, -3.20 to -3.02) (Table 1 and **Supplementary Table 2**). At the country level, the age-standardized prevalence rate of vitamin A deficiency per 100,000 population was highest in Somalia (63640 [59280-67658]), followed by Niger (43502 [38968-48083]), Micronesia (34769 [30794-38842]) and Chad (34261 [29898-38896]) in 2019 (Figure 1 **and Supplementary Table 3**). Among all the GBD regions, the highest age-standardized prevalence rates were observed in Central and Eastern sub-Saharan Africa, with estimated prevalence rates per 100,000 population of 25905 (23288-28883) and 23500 (22337-24765), respectively (**Supplementary Table 4**). Regarding sex, the age-standardized prevalence rate per 100,000 population between 1990 and 2019 was higher in males than in females (**Supplementary Figure 1**). Regarding age, vitamin A deficiency prevalence peaked in children aged 1-4 years (**Supplementary Figure 4 and Supplementary Table 5**). Between 1990 and 2019, the age-standardized prevalence rate due to vitamin A deficiency was highest in low- and low-middle-SDI countries and showed a decreasing trend in all five SDI regions (**Supplementary Figure 2 and Supplementary Table 6**). The trends of the age-standardized incidence and DALY rates were relatively similar to that of the age-standardized prevalence rate of vitamin A deficiency (**Supplementary Figures 1-4 and Supplementary Tables 1-6**).

### Dietary iron deficiency

The global age-standardized prevalence rate per 100,000 population was 14191 (13932-14417) in 2018 and 14106 (13851-14342) in 2019 (**Supplementary Table 1**). The global age-standardized prevalence rate decreased with an EAPC of -0.546 (95% CI, -0.585 to -0.507) (Table 1 and **Supplementary Table 2**). Bhutan (32086 [34028-30283]), Zambia (29784 [27774-31630]), and India (28344 [27754-28888]) had the highest age-standardized prevalence rates in 2019 (Figure 1 **and Supplementary Table 3**). In addition, the age-standardized prevalence rate showed an upward trend in Burkina Faso, Yemen, Zimbabwe, Guinea-Bissau, Zambia, Ghana, Mali, Vanuatu, Fiji, Cameroon, Nigeria, Guinea, Central African Republic, Togo, Haiti, Kenya, and Sierra Leone (Figure 2 **and Supplementary Table 2**). At the regional level, the highest age-standardized prevalence rates of dietary iron deficiency per 100,000 population were observed in South Asia (27404 [26799-27971]) and Western sub-Saharan Africa (21259 [20245-22233]) in 2019 (**Supplementary Table 4**). Between 1990 and 2019, the worldwide age-standardized prevalence rate was higher in females than in males (**Supplementary Figure 1**) and highest in the low- and low-middle-SDI countries compared with other SDI countries (**Supplementary Figure 2 and Supplementary Table 6**); in 2019, it peaked among infants (**Supplementary Figure 4 and Supplementary Table 5**). The trend of the age-standardized DALY rate was similar to that of the age-standardized prevalence rate of dietary iron deficiency (**Supplementary Figures 1-4 and Supplementary Tables 1-6**).

### Burdens of micronutrient deficiencies by SDI and HAQ index

**Supplementary Figure 5** presents the observed global- and regional-level age-standardized DALY rates from 1990 to 2019 and their associations with the SDI. The expected trend was linear in nature, decreasing with increasing SDI values. South Asia, Central Asia and high-income Asia Pacific had a higher age-standardized DALY rate of dietary iron deficiency than expected based on the SDI between 1990 and 2019. Moreover, the highest age-standardized DALY rates of iodine deficiency and vitamin A deficiency were observed in Central sub-Saharan Africa (**Supplementary Figure 5**). The associations of age-standardized prevalence and incidence rates and SDI were similar to those of the age-standardized DALY rate and SDI (**Supplementary Figure 6**).

**Figure 3:**
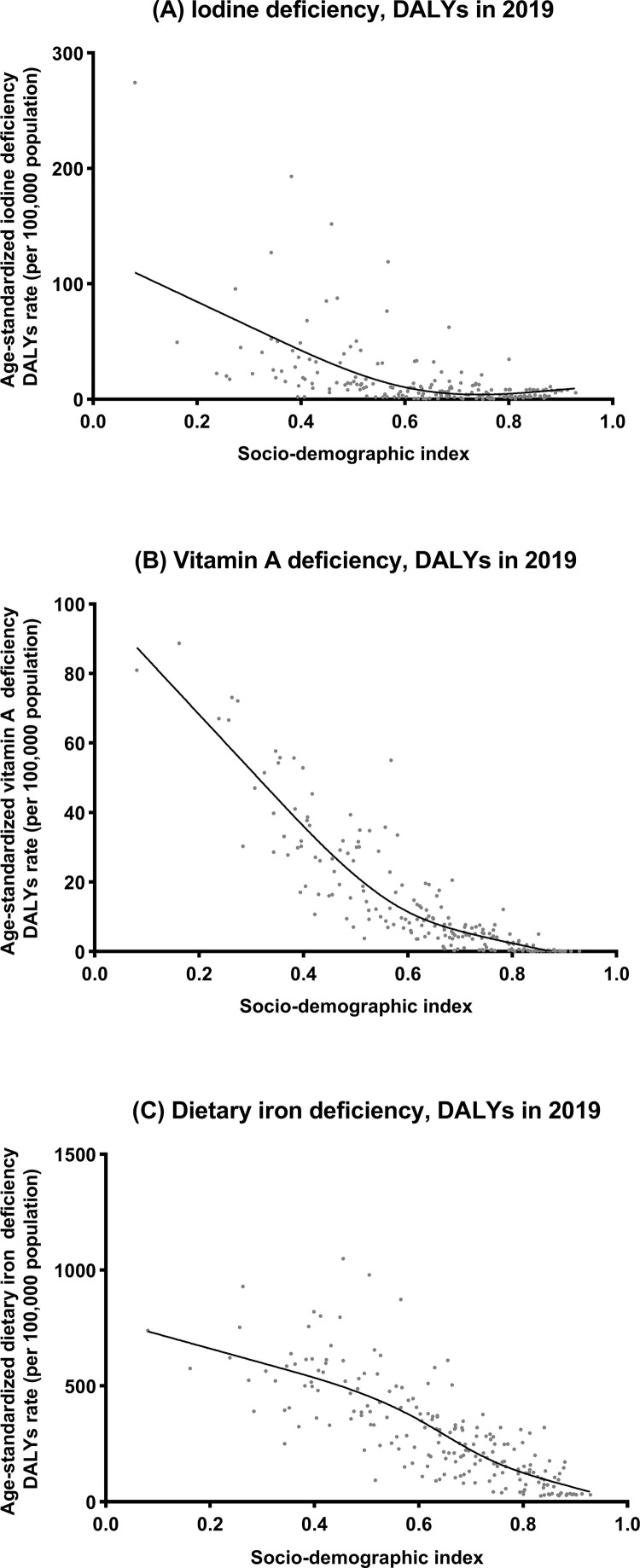
Age-standardized DALY rates of iodine deficiency, vitamin A deficiency, and dietary iron deficiency in 204 countries and territories by SDI, 2019. Note: The grey circles represent countries that were available on SDI data. DALY, disability-adjusted life year; SDI, sociodemographic index.

**Figure 4:**
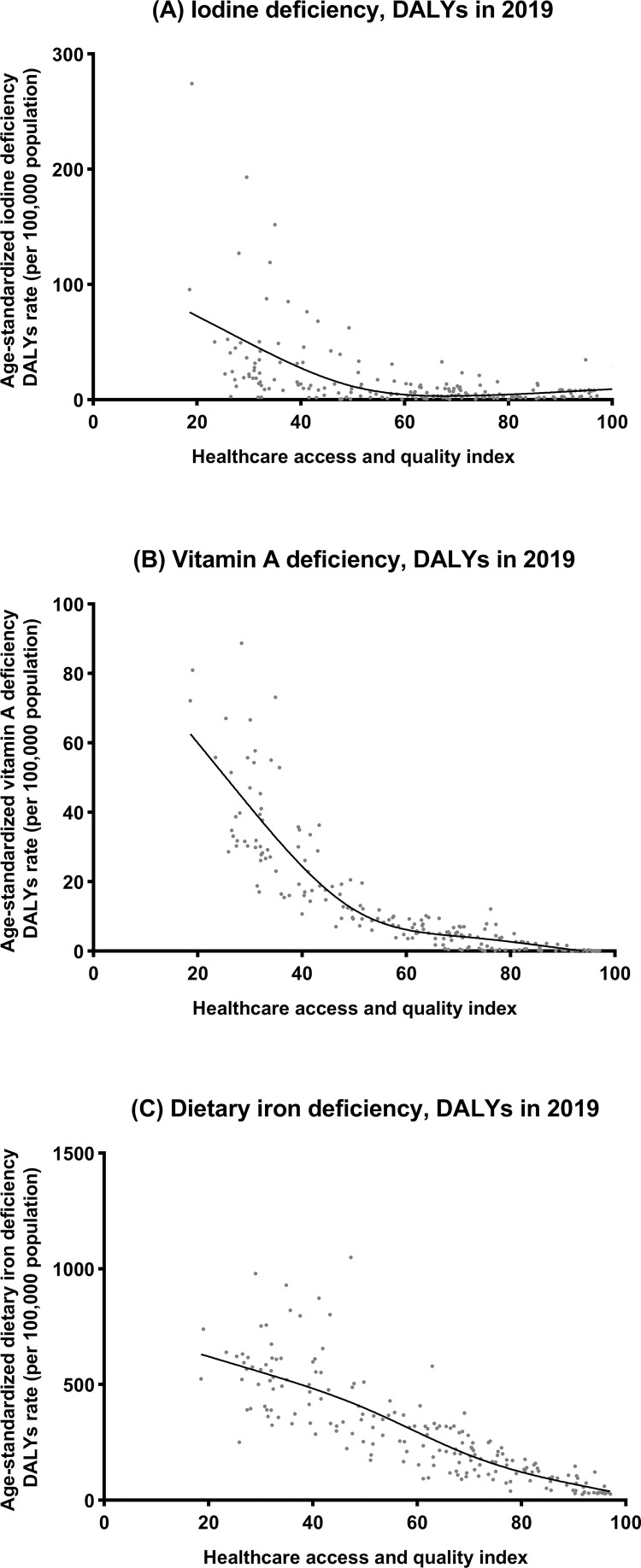
Age-standardized DALY rates of iodine deficiency, vitamin A deficiency, and dietary iron deficiency in 204 countries and territories by HAQ, 2019. Note: The grey circles represent countries that were available on HAQ index data. DALY, disability-adjusted life year; HAQ, healthcare access and quality.

Figure 3 and Figure 4 show the national-level age-standardized DALY rates and their associations with the SDI and HAQ index. The expected trends were linear in nature, decreasing with increasing SDI and HAQ index values. The age-standardized DALY rates were higher than the expected levels based on only the SDI for a number of countries/territories, such as Somalia, Niger and Bhutan. Notably, the age-standardized DALY rate of iodine deficiency rose accordingly after the SDI was greater than 0.75 and the HAQ was greater than 80. In addition, Italy's burden of iodine deficiency was the most prominent among these developed countries. These trends were also observed between the SDI and HAQ index and age-standardized prevalence and incidence rates (**Supplementary Figures 7-8**).

## Discussion

We conducted a broad literature search of multiple databases and found no similar study. This secondary analysis is the first to provide the most up-to-date estimates of trends in the prevalence, incidence, and DALYs for micronutrient deficiencies in 204 countries and territories from 1990 to 2019. The study comprehensively describes the burdens of dietary iron, vitamin A, and iodine deficiencies, allowing direct comparisons over time and among regions. Our results showed decreasing trends in the age-standardized prevalence, incidence and DALY rates in the past three decades globally. However, micronutrient deficiencies still result in a remarkable burden in sub-Saharan Africa and South Asia. Notably, we found an increasing prevalence trend of micronutrient deficiencies in several counties, even in high-income developed countries such as Andorra, Portugal, Monaco, and San Marino, although the burden remained at a fairly low level. Furthermore, the burden of iodine deficiency increased with higher SDI and HAQ index among developed countries.

Our data showed that the age-standardized prevalence rate declined from 1990 to 2019. In addition to this long-term downward trend, the prevalence rate of iodine deficiency decreased sharply between 1999 and 2000 worldwide. This might be due to the increase in the proportion of the population consuming iodized salt from less than 20% in 1990 to 70% in 2000.^22^^,^^23^ Despite the progress in iodine deficiency elimination programs, more than 2 billion people worldwide are still at risk of insufficient iodine intake.^24^^,^^25^ Sub-Saharan Africa and South Asia have high iodine deficiency burdens, consistent with previous studies. The high prevalence in sub-Saharan Africa could be due to an interaction among inadequate dietary diversity, poor sanitation and infectious diseases.^26^ As the most populous country in South Asia, India reported that household iodized salt consumption was only 51%, indicating that iodine intake is insufficient at the population level.^27^ However, this situation is not limited to underdeveloped countries. Iodine deficiency has also been observed in highly developed countries, even though these countries had been previously considered to have eliminated iodine deficiency disorders. This study showed that females and young adults were disproportionately affected by a high prevalence of iodine deficiency. Several studies in many countries also observed this sex difference.^27^ In addition, iodine deficiency in women can lead to devastating effects during pregnancy, leading to miscarriage, stillbirth, congenital malformations, neurosis, and increased perinatal and infant mortality.^26^ We also found that the burden of iodine deficiency increased with higher SDI and HAQ index among developed countries. Most governments of developed countries, however, still give low priority to addressing iodine deficiency in their populations.^28^^,^^29^ This could be because approximately 90% of salt consumption in developed countries is from purchased processed foods; if only household salt is iodized, it will not supply adequate iodine.^30^ Therefore, convincing the food industry to use iodized salt in their products is crucial for successful control of iodine deficiency in industrialized countries.

Consistent with previous studies, this study found that the age-standardized prevalence of vitamin A deficiency has decreased in the last three decades.^31^^,^^32^ The higher prevalence in males could be explained by physiological differences. Vitamin A deficiency mainly affects children under 5 years of age and populations in South Asia and sub-Saharan Africa. The persistent high prevalence of vitamin A deficiency in South Asia and sub-Saharan Africa might be due to insufficient dietary diversification, unsuccessful food fortification, and the restricted effect of vitamin A capsule supplementation on serum retinol. Frequent exposure to infections increases the risk of subclinical deficiency and reduces the serum retinol concentration in children with adequate liver stores, which might also lead to persistent vitamin A deficiency in regions with a high infection burden.^33^^,^^34^ Vitamin A supplementation is a cost-effective public health intervention. Vitamin A supplements play a vital role in countries with high mortality among children under 5 years of age; these countries are usually low-income countries with low nutritional levels. In these contexts, the delivery of vitamin A is crucial, but the progress made in the past decades has slowed.^35^ It is well known that vitamin A deficiency is associated with ocular pathologies and all-cause mortality, and vitamin A deficiency contributes to reduced resistance to infections, especially diarrheal diseases and measles, and increased mortality in children under 5 years of age.^36^ Consequently, the promotion of vitamin A supplementation programs as preventative public health measures has expanded worldwide. However, the coverage of these projects in East (67%) and Southern Africa (53%) is not sufficient, which emphasizes the need for better strategies to reduce the incidence and associated DALYs in these regions.^37^

From 1990 to 2019, the age-standardized prevalence rate of dietary iron deficiency declined worldwide. The global increase in the consumption of animal products, dietary diversification and improved access to foods might be possible reasons.^38^ Although the availability of animal products for human consumption has increased in East and Southeast Asia, dietary iron deficiency is still an important public health problem that requires comprehensive interventions.^39^^,^^40^ When iron intake no longer meets the requirement of normal iron turnover and loss, iron-deficiency anemia occurs, accounting for approximately half of anemia cases globally.^40^^,^^41^ The current study shows a sex difference in the dietary iron deficiency burden, as the prevalence was higher in females than in males. The main reason for sex differences in the prevalence of iron deficiency is that the requirements for iron are almost double for women of reproductive age compared to men, and in pregnancy, they even triple. When diets are largely plant-based, iron requirements further increase to compensate for the lower bioavailability of iron in plant foods. Bone marrow radioactive iron studies have shown that iron absorption depends on iron stores in the body and is not sex-specific.^42^ This indicates that the global sex difference might also be explained by differences in dietary intake of iron-rich foods. In addition, menstruation might also cause a negative iron balance in apparently healthy women.^43^ The public health impact of iron deficiency is more pronounced among women than among men, as it can lead to iron deficiency in future generations unless timely measures are taken. The prevalence of dietary iron deficiency is higher among children aged under five years than among children aged over five years, which is consistent with previous estimates.^44^ This could be due to a low rate of iron supplementation during pregnancy, as many pregnant women do not attend antenatal clinics or receive sufficient doses of supplements or because there is insufficient emphasis on behavioral aspects related to regular supplement use.^45^

We also found that the decreasing trends in the age-standardized incidence and DALY rates paralleled those of the age-standardized prevalence rates at the global and regional levels; however, the highest age-standardized incidence of iodine deficiency was observed in the population aged 10 to 19. This could be due to an increase in the demand for iodine during puberty, which is not sufficient considering the limited amount of iodine available in food and salt.^46^ Our analyses of the associations between the SDI and HAQ index and the burdens of micronutrient deficiencies have not been comprehensively reported previously. As shown in the results, the countries with the greatest micronutrient deficiency burdens were in regions with a lower SDI and HAQ index. The differences in micronutrient deficiency burdens among SDI and HAQ index levels were expected due to the interaction among inadequate dietary diversity, poor sanitation and infectious diseases. However, a higher age-standardized DALY rate of dietary iron deficiency than expected based on SDI between 1990 and 2019 was found in the High-income Asia Pacific region, which deserves further exploration.

This study found great regional differences in the burden of micronutrient deficiencies, which were significantly associated with economic and social development. These differences may increase in the future and trigger a greater public health crisis. In Somalia, for instance, our results showed that more than 20% and over 60% of the population suffered from iodine deficiency and vitamin A deficiency, respectively. Internal conflicts and continuous invasions by foreign governments have caused a series of health and nutrition crises.^47^ Of greatest concern is the fact that the cycle of micronutrient deficiencies perpetuates across generations, with far-reaching consequences for the future population.^48^ Thus, targeted measures for crucial regions are needed. The current analysis makes the data easy to interpret by nongovernmental organizations who are making decisions about what to invest in, and where to intervene. The global burden of dietary iron deficiency is higher than that of iodine deficiency, possibly because universal salt iodization is more achievable and less costly to implement than iron supplementation programs, especially in low- and middle-income countries.^48^ In addition, the age-standardized DALY rates of iodine deficiency and iron deficiency were associated differently with SDI > 0.75 and HAQ > 80. This result should alert health authorities in some developed countries that iodine deficiency disorders are not confined to underdeveloped and developing countries. Of note, the definition of dietary iron deficiency used in the current study did not quantify ferritin levels. To better understand the global burden of dietary iron deficiency, it is recommended that the WHO update the data by using gold standard biomarker measurements.

This study has several strengths. This study presents the latest epidemiologic patterns of micronutrient deficiencies burden at global, regional, and national levels among different age, sex, and SDI categories to help policy makers form a target management strategy from a global perspective. The current study explored the correlation between SDIs and HAQ indexes and micronutrient deficiency burden. In addition, this study is the first to describe the secular trends of micronutrient deficiency prevalence in 204 countries and territories from 1990 to 2019. Although the GBD study estimates were produced using robust data collection and analysis methods, the estimates in this study should be interpreted within the context of the following limitations. First, the study is limited by the gaps in data, especially among low- and middle-income countries, and variations in data quality in the GBD study. It must be noted that the data presented here were primarily derived from modeled data through the processes in DisMod-MR. True population-based national data on the incidence and prevalence of micronutrient deficiencies were available from very few countries; thus, the present study relies on modeling. As such, these national estimates should be interpreted with caution. Detailed methodological considerations of the overall estimation process for nonfatal outcomes have been discussed elsewhere.^18^ Second, there was a lack of high-quality data, particularly regarding the incidence and prevalence of other nutritional deficiencies, including zinc, calcium, folate, vitamin B12, and vitamin C deficiencies. Third, due to the absence of mortality data, the DALYs were calculated from only YLDs, which might have led to underestimation. Third, dietary iron deficiency incidence rates were unavailable in the GBD 2019; thus, we used only the prevalence and DALYs to describe the dietary iron deficiency burden. In addition, the trend in dietary iron deficiency with or without anemia is unclear due to the unavailability of relevant data in the GBD 2019. Last, iodine deficiency was estimated using only grade 2 goiter, which might have underestimated the prevalence. Including all forms of iodine deficiency is a goal of future GBD research. Despite these limitations, this study provides comprehensive estimates using available global evidence.

The global burdens of micronutrient deficiencies have decreased since 1990 due to tremendous efforts in recent decades. However, micronutrient deficiencies and associated DALYs are still high, especially iodine deficiency and vitamin A deficiency in Central sub-Saharan Africa and dietary iron deficiency in South Asia. Young adults, children aged under five, and infants are disproportionately affected by iodine, vitamin A, and dietary iron deficiency, respectively. The potential burden of iodine deficiency in some developed countries is worthy of attention. The results of this study could aid policy makers in implementing cost-effective interventions and reducing the burdens of micronutrient deficiencies, particularly in countries with low SDIs and HAQ indexes. To better understand the global burden of micronutrient deficiencies, there is a need for further GBD study by using gold standard biomarker measurements.

## Declaration of interests

No potential conflicts of interest relevant to this article were reported.

## Appendix Supplementary materials

Image, application 1

Image, application 1
