# Supplementary material for: Global, regional, and national burdens of common micronutrient deficiencies from 1990 to 2019: A secondary trend analysis based on the Global Burden of Disease 2019 study
Source: eClinicalMedicine. 2022 Feb 12;44:101299. doi: 10.1016/j.eclinm.2022.101299 (PMC8850322; doi:10.1016/j.eclinm.2022.101299)
Supplement: Supplementary file 1 [file mmc1.docx]

**SUPPLEMENT**

**Supplementary Methods.** Incidence, prevalence and DALY estimation process.

**Supplementary Figure 1.** Global age-standardized prevalence, incidence, and DALY rates of iodine deficiency, vitamin A deficiency, and dietary iron deficiency in males and females from 1990 to 2019.

**Supplementary Figure 2.** Age-standardized prevalence, incidence, and DALY rates by SDI quintile for iodine deficiency, vitamin A deficiency, and dietary iron deficiency from 1990 to 2019.

**Supplementary Figure 3.** Global age-standardized incidence and DALY rates of iodine deficiency, vitamin A deficiency, and dietary iron deficiency in 2019.

**Supplementary Figure 4.** Global age-specific prevalence, incidence, and DALY rates of iodine deficiency, vitamin A deficiency, and dietary iron deficiency in 2019.

**Supplementary Figure 5.** Age-standardized DALY rates of iodine deficiency, vitamin A deficiency, and dietary iron deficiency globally and in 21 GBD regions by SDI, 1990-2019.

**Supplementary Figure 6.** Age-standardized prevalence and incidence rates of iodine deficiency, vitamin A deficiency, and dietary iron deficiency globally and in 21 GBD regions by SDI, 1990-2019.

**Supplementary Figure 7.** Age-standardized prevalence and incidence rates of iodine deficiency, vitamin A deficiency, and dietary iron deficiency in 204 countries and territories by SDI, 2019.

**Supplementary Figure 8.** Age-standardized prevalence and incidence rates of iodine deficiency, vitamin A deficiency, and dietary iron deficiency in 204 countries and territories by HAQ index, 2019.

**Supplementary Table 1.** Global age-standardised prevalence, incidence, and DALYs rate of iodine deficiency, vitamin A deficiency, and dietary iron deficiency in males, females, and both sexes from 1990 to 2019.

**Supplementary Table 2.** The estimated annual percentage changes (APC) of age-standardized prevalence rate worldwide from 1990 to 2019.

**Supplementary Table 3.** Age-standarised prevalence, incidence, and DALYs rate of iodine deficiency, vitamin A deficiency, and dietary iron deficiency for 204 countries and territories in 2019.

**Supplementary Table 4.** Age-standarised prevalence, incidence, and DALYs rate of iodine deficiency, vitamin A deficiency, and dietary iron deficiency globally and for 21 GBD regions, 1990-2019.

**Supplementary Table 5.** Global age-specific prevalence, incidence, and DALYs rate of iodine deficiency, vitamin A deficiency, and dietary iron deficiency in 2019.

**Supplementary Table 6.** Age-standarised prevalence, incidence, and DALYs rate of iodine deficiency, vitamin A deficiency, and dietary iron deficiency grouped by SDI quintiles from 1990 to 2019.
