# Supplementary material for: Global, regional, and national burdens of common micronutrient deficiencies from 1990 to 2019: A secondary trend analysis based on the Global Burden of Disease 2019 study
Source: eClinicalMedicine. 2022 Feb 12;44:101299. doi: 10.1016/j.eclinm.2022.101299 (PMC8850322; doi:10.1016/j.eclinm.2022.101299)
Supplement: Supplementary file 2 [file mmc2.docx]

**SUPPLEMENT**

**Supplementary Methods.** Incidence, prevalence and DALY estimation process.

**Supplementary Figure 1.** Global age-standardized prevalence, incidence, and DALY rates of iodine deficiency, vitamin A deficiency, and dietary iron deficiency in males and females from 1990 to 2019.

**Supplementary Figure 2.** Age-standardized prevalence, incidence, and DALY rates by SDI quintile for iodine deficiency, vitamin A deficiency, and dietary iron deficiency from 1990 to 2019.

**Supplementary Figure 3.** Global age-standardized incidence and DALY rates of iodine deficiency, vitamin A deficiency, and dietary iron deficiency in 2019.

**Supplementary Figure 4.** Global age-specific prevalence, incidence, and DALY rates of iodine deficiency, vitamin A deficiency, and dietary iron deficiency in 2019.

**Supplementary Figure 5.** Age-standardized DALY rates of iodine deficiency, vitamin A deficiency, and dietary iron deficiency globally and in 21 GBD regions by SDI, 1990-2019.

**Supplementary Figure 6.** Age-standardized prevalence and incidence rates of iodine deficiency, vitamin A deficiency, and dietary iron deficiency globally and in 21 GBD regions by SDI, 1990-2019.

**Supplementary Figure 7.** Age-standardized prevalence and incidence rates of iodine deficiency, vitamin A deficiency, and dietary iron deficiency in 204 countries and territories by SDI, 2019.

**Supplementary Figure 8.** Age-standardized prevalence and incidence rates of iodine deficiency, vitamin A deficiency, and dietary iron deficiency in 204 countries and territories by HAQ index, 2019.

**Supplementary Table 1.** Global age-standardised prevalence, incidence, and DALYs rate of iodine deficiency, vitamin A deficiency, and dietary iron deficiency in males, females, and both sexes from 1990 to 2019.

**Supplementary Table 2.** The estimated annual percentage changes (APC) of age-standardized prevalence rate worldwide from 1990 to 2019.

**Supplementary Table 3.** Age-standarised prevalence, incidence, and DALYs rate of iodine deficiency, vitamin A deficiency, and dietary iron deficiency for 204 countries and territories in 2019.

**Supplementary Table 4.** Age-standarised prevalence, incidence, and DALYs rate of iodine deficiency, vitamin A deficiency, and dietary iron deficiency globally and for 21 GBD regions, 1990-2019.

**Supplementary Table 5.** Global age-specific prevalence, incidence, and DALYs rate of iodine deficiency, vitamin A deficiency, and dietary iron deficiency in 2019.

**Supplementary Table 6.** Age-standarised prevalence, incidence, and DALYs rate of iodine deficiency, vitamin A deficiency, and dietary iron deficiency grouped by SDI quintiles from 1990 to 2019.

**Supplementary Methods.** Incidence, prevalence, and DALY estimation process.

The 2019 GBD study modeled the prevalence of micronutrient deficiencies using DisMod-MR.^1^ It is a Bayesian meta-regression tool which combines epidemiological data from multiple sources, adjusts inconsistent data and forecasts and updates data for regions and parameters with no or little data. To model iodine deficiency, grade 2 goiter was chosen over grade 1 due to the greater reliability and consistency of the clinical diagnosis of grade 2 goiter worldwide.^1^ The modeling used a study-level covariate to indicate national observations, where nationally representative studies were set as the reference category and household iodized salt consumption proportion as a country-level covariate. The vitamin A deficiency estimates were made sequentially. First, vitamin A supplementation coverage was estimated. The case definition for the supplementation model was the proportion of children 6–59 months of age who received at least one dose of vitamin A in the previous 6 months. Second, age- and sex-specific prevalence of vitamin A deficiency (serum retinol < 0.7 µmol/L) was estimated. Third, for models of the prevalence of blindness and vision loss due to vitamin A deficiency, this was run as a single-parameter meta-regression on prevalence, so incidence estimates were not generated. Further details on modeling strategy are available elsewhere.^1^

Disability-adjusted life-year (DALY) were calculated by summing years of life lost (YLLs) due to premature mortality and years of life lived with disability (YLDs), thereby incorporating both fatal and non-fatal burden. As there was no mortality data, all DALY estimates for dietary iron, vitamin A and iodine deficiency were from the YLDs.

All parameter estimates generated in the GBD 2019 were accompanied by 95% uncertainty intervals (UIs). The uncertainty ranges reported around YLDs incorporate both uncertainty in prevalence and in the disability weight. To do this, 1000 samples of comorbidity-corrected YLDs and 1000 samples of the disability weight to generate 1000 samples of the YLD distribution were considered. The 95% uncertainty interval is reported as the 25th and 975th values of the distribution.

Reference:

[1] GBD 2019 Diseases and Injuries Collaborators. Global burden of 369 diseases and injuries in 204 countries and territories, 1990-2019: a systematic analysis for the Global Burden of Disease Study 2019. Lancet 2020; 396(10258), 1204-1222. doi:10.1016/S0140-6736(20)30925-9

**Supplementary Figure 1.** Global age-standardized prevalence, incidence, and DALY rates of iodine deficiency, vitamin A deficiency, and dietary iron deficiency in males and females from 1990 to 2019.


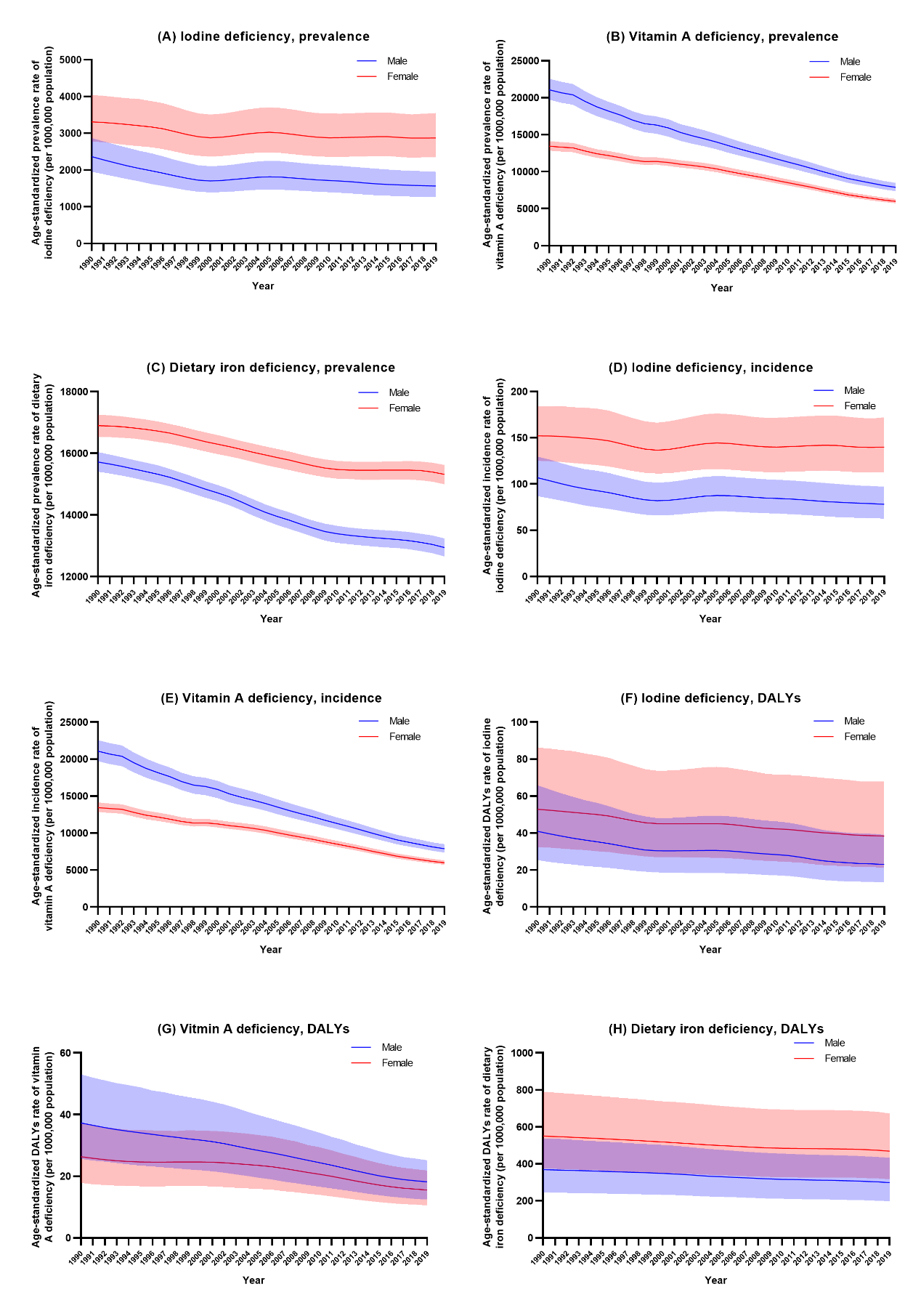


**Supplementary Figure 2.** Age-standardized prevalence, incidence, and DALY rates by SDI quintile for iodine deficiency, vitamin A deficiency, and dietary iron deficiency from 1990 to 2019.


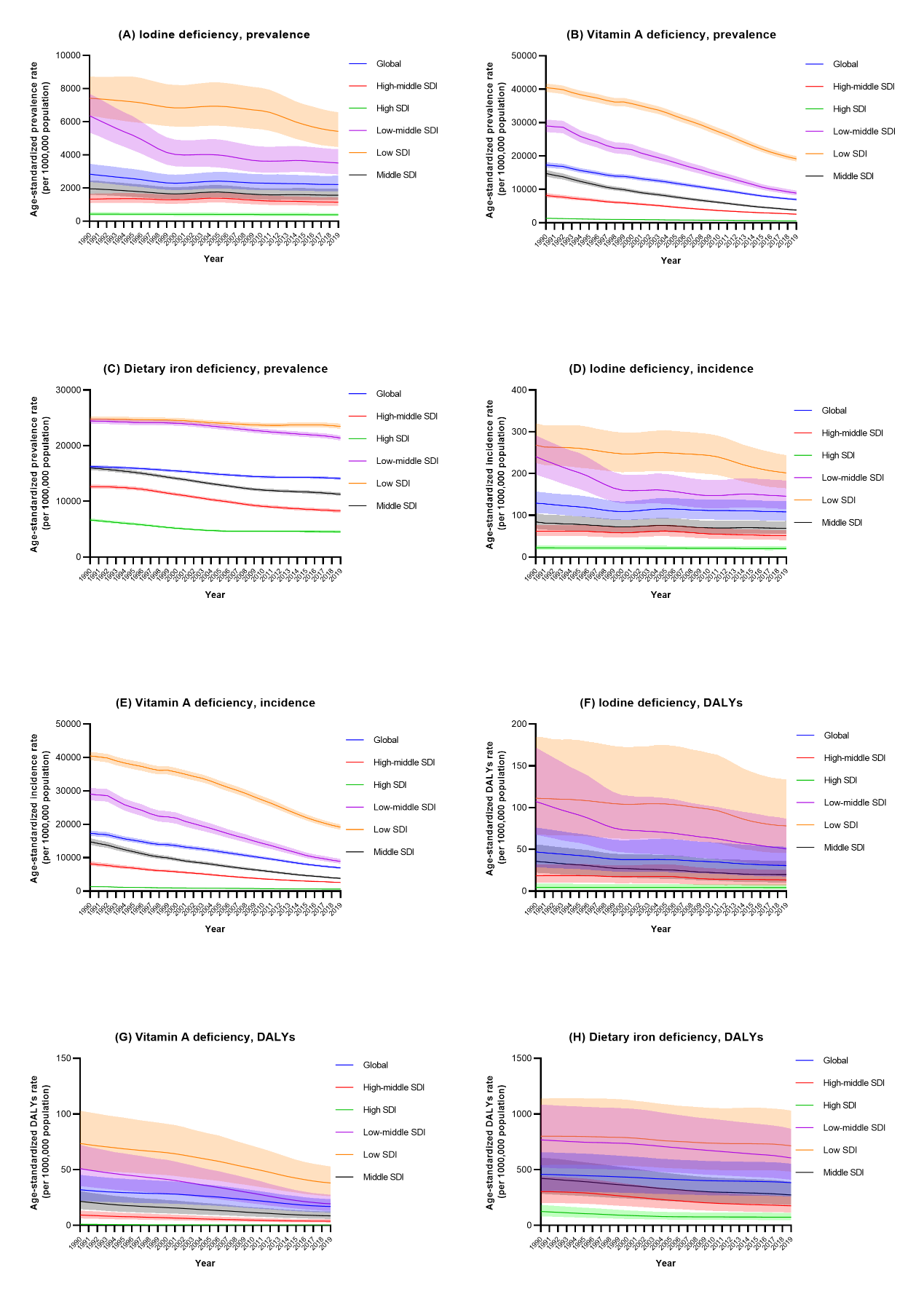


**Supplementary Figure 3.** Global age-standardized incidence and DALY rates of iodine deficiency, vitamin A deficiency, and dietary iron deficiency in 2019.


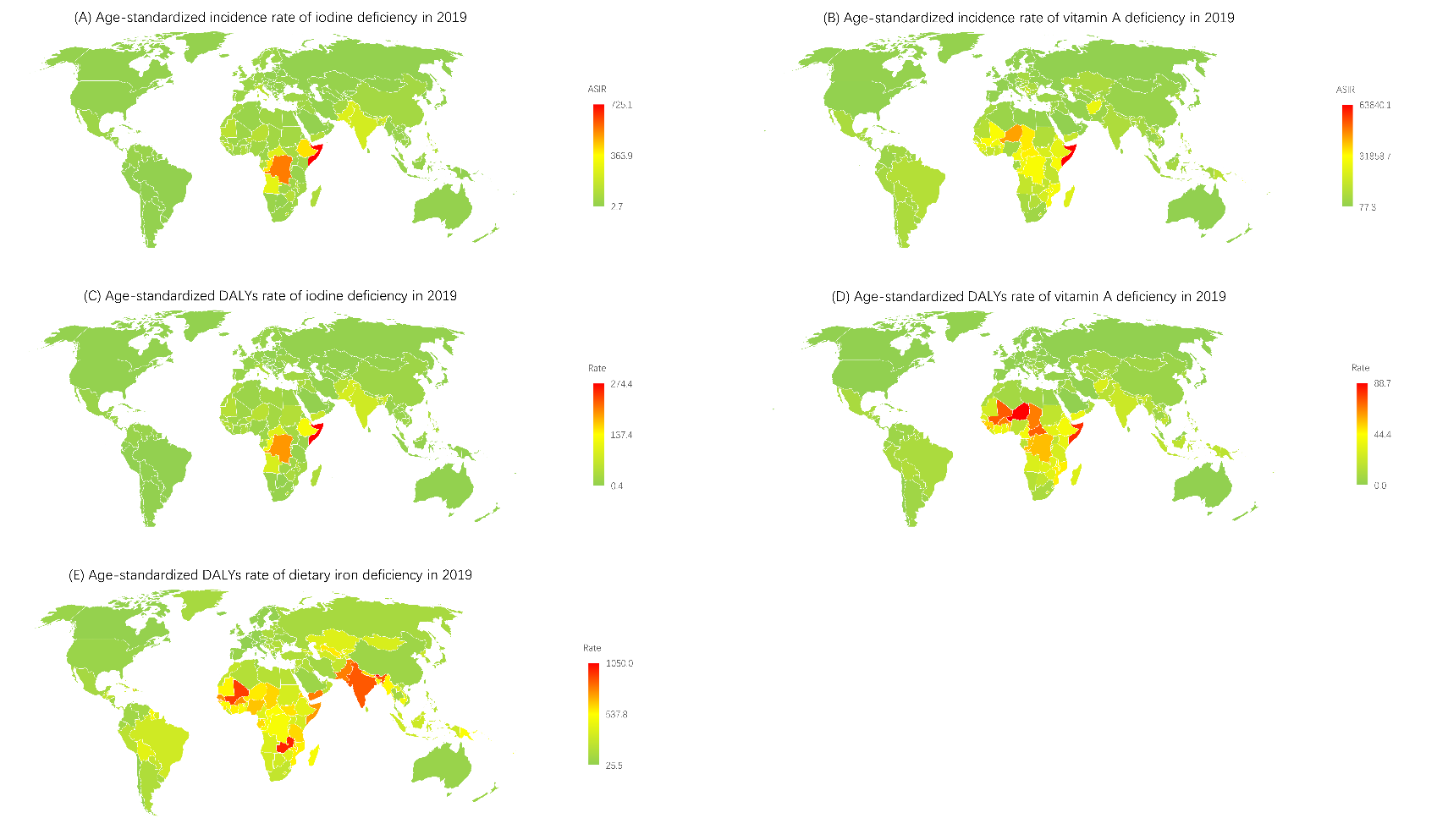


**Supplementary Figure 4.** Global age-specific prevalence, incidence, and DALY rates of iodine deficiency, vitamin A deficiency, and dietary iron deficiency in 2019.


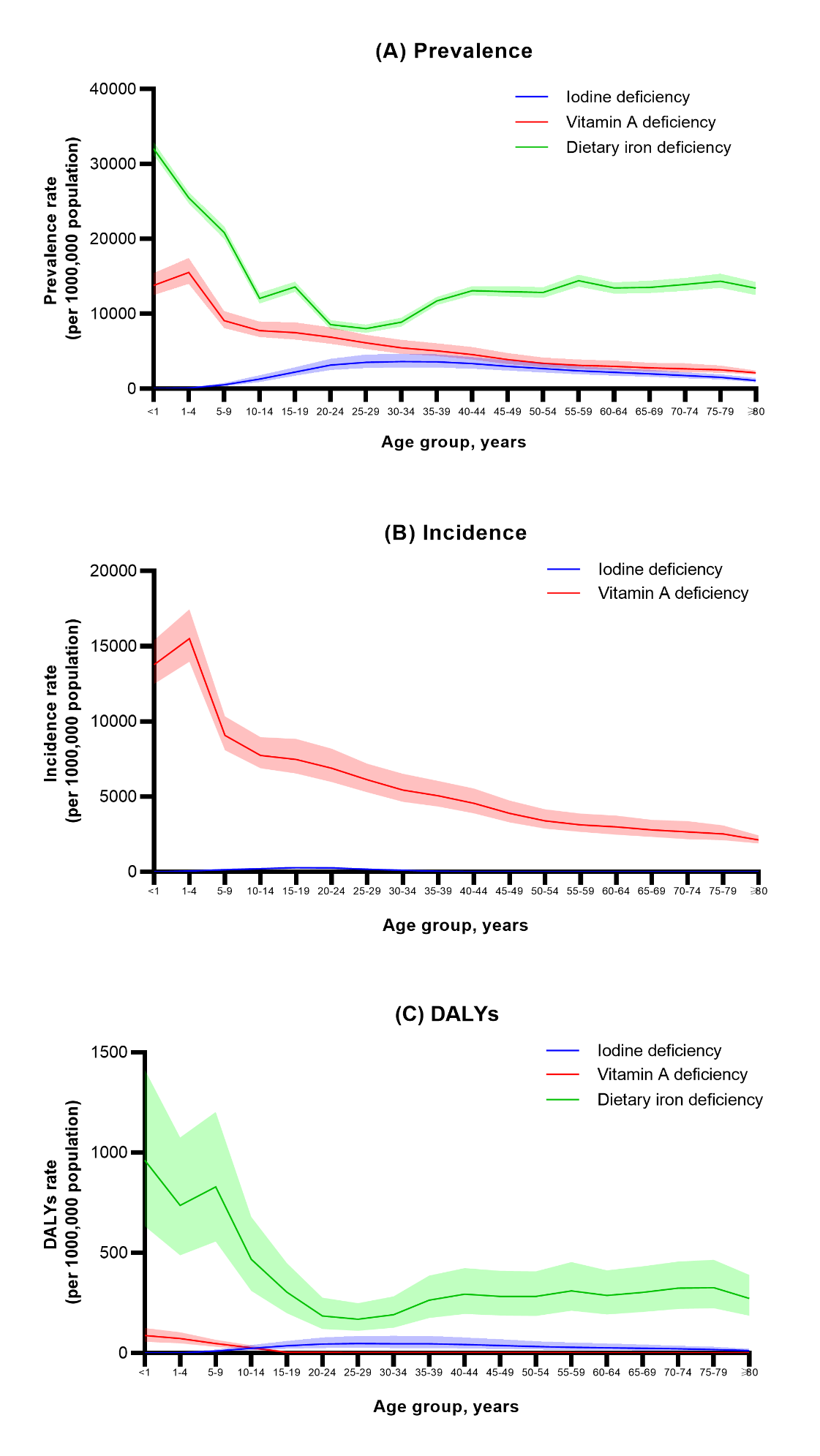


**Supplementary Figure 5.** Age-standardized DALY rates of iodine deficiency, vitamin A deficiency, and dietary iron deficiency globally and in 21 GBD regions by SDI, 1990-2019.


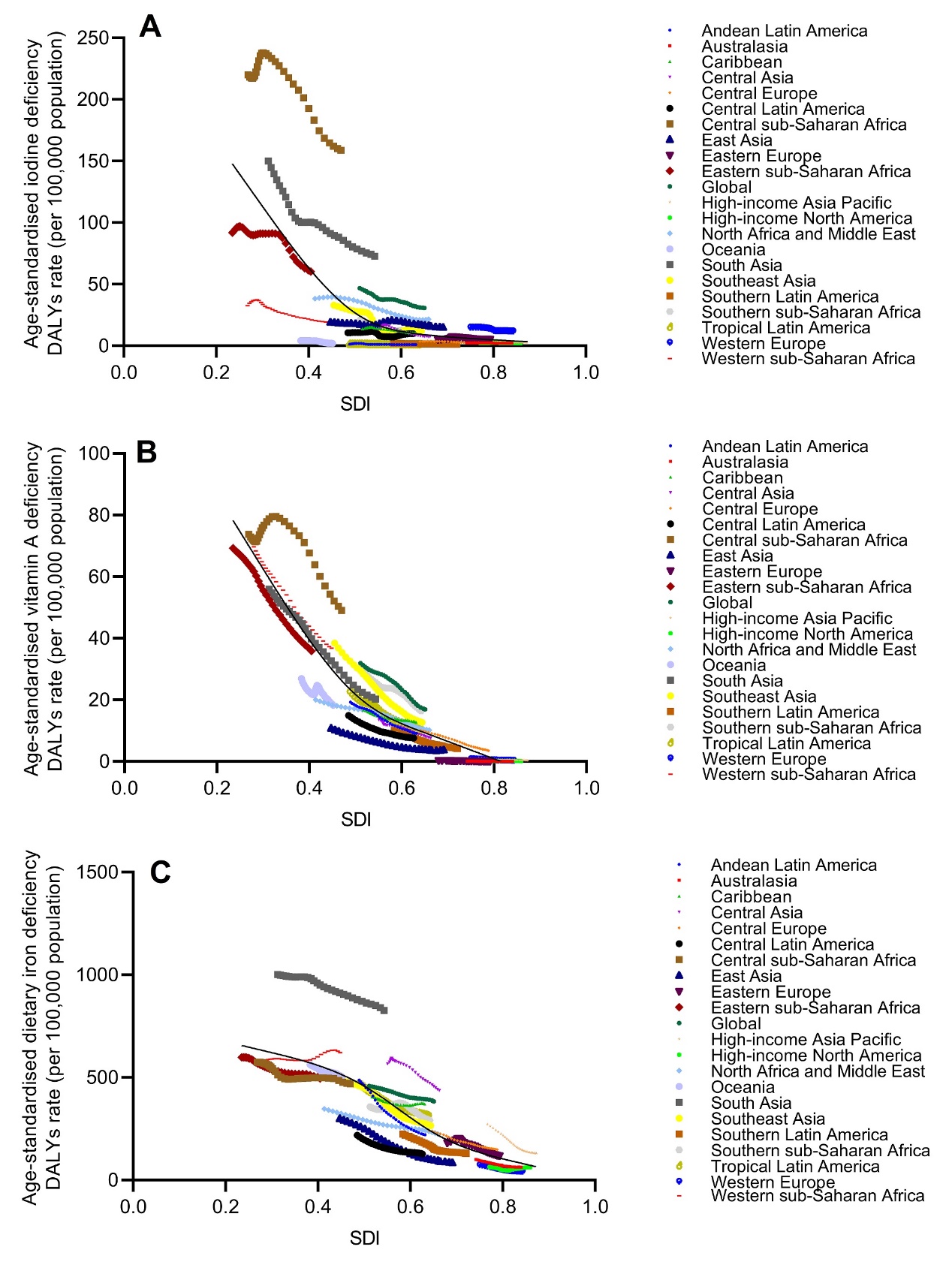


**Supplementary Figure 6.** Age-standardized prevalence and incidence rates of iodine deficiency, vitamin A deficiency, and dietary iron deficiency globally and in 21 GBD regions by SDI, 1990-2019.


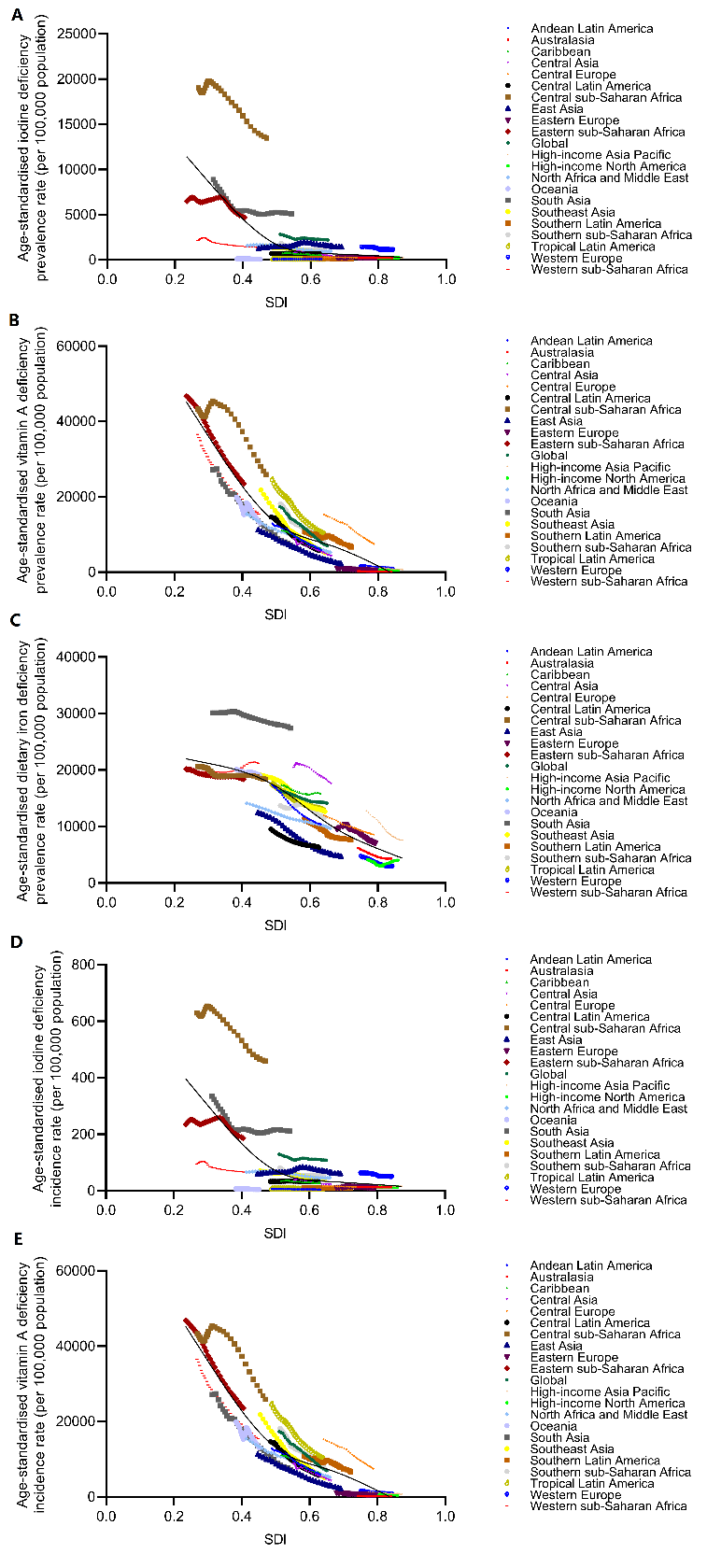


**Supplementary Figure 7.** Age-standardized prevalence and incidence rates of iodine deficiency, vitamin A deficiency, and dietary iron deficiency in 204 countries and territories by SDI, 2019.


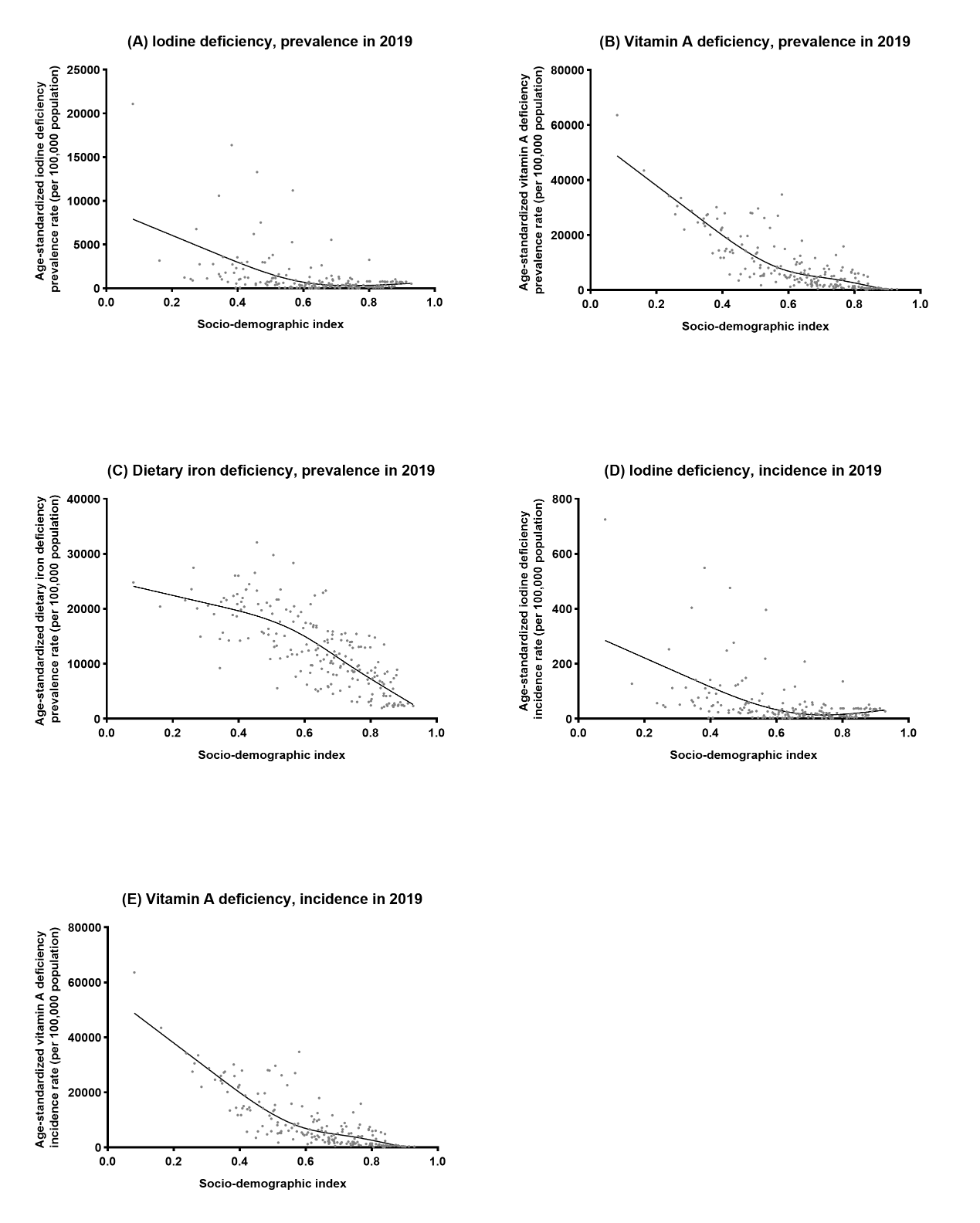


**Supplementary Figure 8.** Age-standardized prevalence and incidence rates of iodine deficiency, vitamin A deficiency, and dietary iron deficiency in 204 countries and territories by HAQ index, 2019.


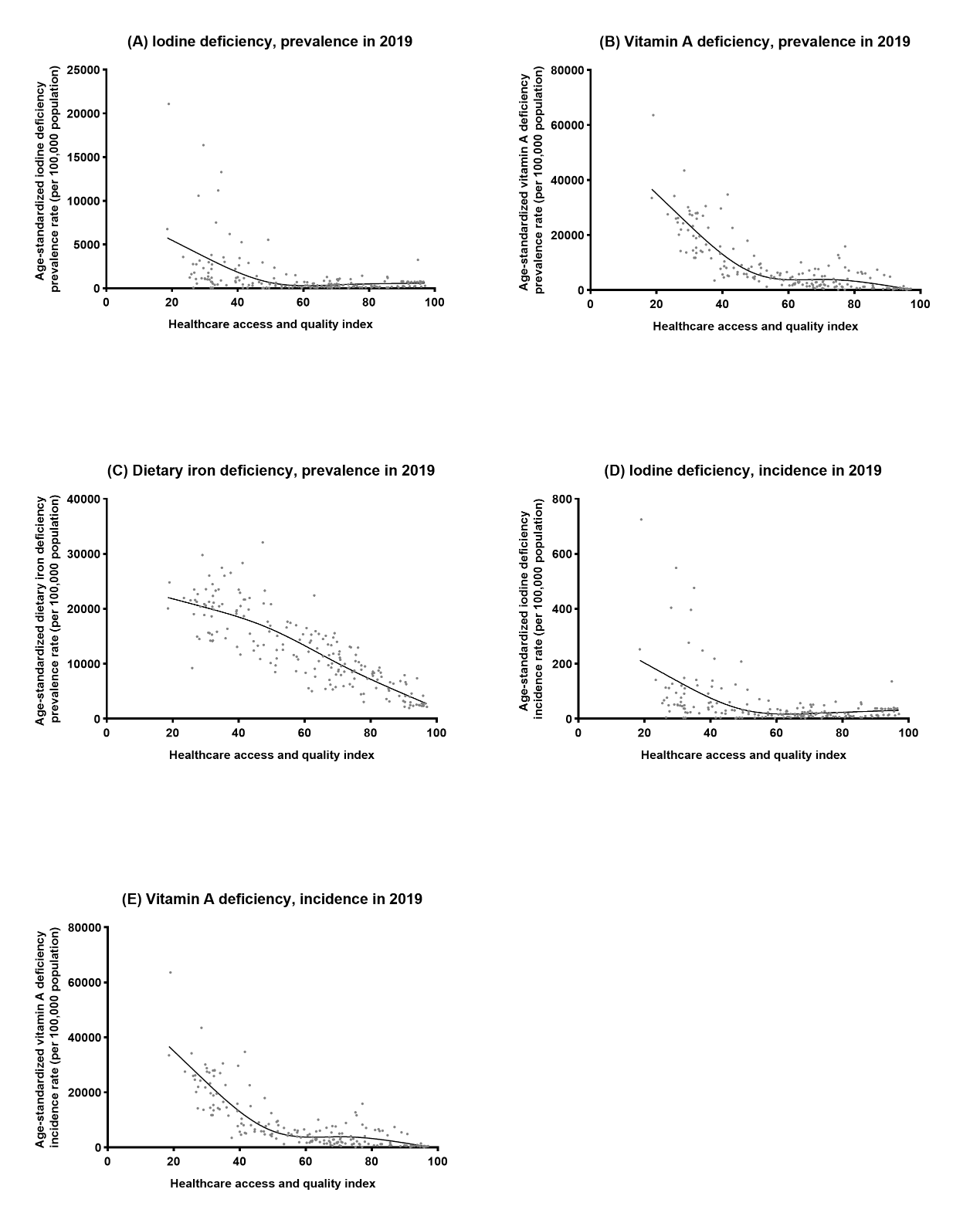


**Supplementary Table 1.** Global age-standardised prevalence, incidence, and DALYs rate of iodine deficiency, vitamin A deficiency, and dietary iron deficiency in males, females, and both sexes from 1990 to 2019.

| Measure | Location | Sex | Micronutrient deficiency | Year | Rate | 95% Upper UI | 95% Lower UI |
| --- | --- | --- | --- | --- | --- | --- | --- |
| DALYs | Global | Male | Dietary iron deficiency | 1990 | 368.9168 | 537.3213 | 245.5376 |
| DALYs | Global | Both | Dietary iron deficiency | 1990 | 458.5358 | 657.3919 | 308.8454 |
| DALYs | Global | Female | Dietary iron deficiency | 1990 | 550.124 | 789.8147 | 373.7397 |
| DALYs | Global | Male | Dietary iron deficiency | 1991 | 367.3173 | 536.0474 | 244.2575 |
| DALYs | Global | Both | Dietary iron deficiency | 1991 | 456.6104 | 654.9451 | 307.9288 |
| DALYs | Global | Female | Dietary iron deficiency | 1991 | 547.8961 | 785.7336 | 372.7026 |
| DALYs | Global | Male | Dietary iron deficiency | 1992 | 365.5008 | 531.8822 | 242.9674 |
| DALYs | Global | Both | Dietary iron deficiency | 1992 | 454.3528 | 652.0833 | 306.5409 |
| DALYs | Global | Female | Dietary iron deficiency | 1992 | 545.2028 | 780.2585 | 371.1168 |
| DALYs | Global | Male | Dietary iron deficiency | 1993 | 363.6319 | 528.0325 | 241.9094 |
| DALYs | Global | Both | Dietary iron deficiency | 1993 | 451.9747 | 649.6023 | 304.7111 |
| DALYs | Global | Female | Dietary iron deficiency | 1993 | 542.3007 | 776.1155 | 368.6812 |
| DALYs | Global | Male | Dietary iron deficiency | 1994 | 361.7588 | 523.967 | 240.8809 |
| DALYs | Global | Both | Dietary iron deficiency | 1994 | 449.5575 | 647.1189 | 303.0456 |
| DALYs | Global | Female | Dietary iron deficiency | 1994 | 539.2959 | 770.1098 | 366.4078 |
| DALYs | Global | Male | Dietary iron deficiency | 1995 | 359.9466 | 520.4209 | 239.7638 |
| DALYs | Global | Both | Dietary iron deficiency | 1995 | 447.2263 | 644.213 | 301.0856 |
| DALYs | Global | Female | Dietary iron deficiency | 1995 | 536.3794 | 765.5975 | 363.5308 |
| DALYs | Global | Male | Dietary iron deficiency | 1996 | 358.0184 | 516.7747 | 238.171 |
| DALYs | Global | Both | Dietary iron deficiency | 1996 | 444.7687 | 640.5014 | 298.8206 |
| DALYs | Global | Female | Dietary iron deficiency | 1996 | 533.307 | 759.7136 | 360.525 |
| DALYs | Global | Male | Dietary iron deficiency | 1997 | 355.7489 | 513.2367 | 236.8015 |
| DALYs | Global | Both | Dietary iron deficiency | 1997 | 441.8936 | 635.5126 | 296.6723 |
| DALYs | Global | Female | Dietary iron deficiency | 1997 | 529.724 | 754.8865 | 357.5518 |
| DALYs | Global | Male | Dietary iron deficiency | 1998 | 353.3476 | 508.6942 | 235.317 |
| DALYs | Global | Both | Dietary iron deficiency | 1998 | 438.8405 | 630.6256 | 294.9334 |
| DALYs | Global | Female | Dietary iron deficiency | 1998 | 525.9005 | 749.6064 | 355.6426 |
| DALYs | Global | Male | Dietary iron deficiency | 1999 | 350.9601 | 505.1319 | 234.1031 |
| DALYs | Global | Both | Dietary iron deficiency | 1999 | 435.836 | 625.461 | 293.498 |
| DALYs | Global | Female | Dietary iron deficiency | 1999 | 522.1661 | 743.7811 | 353.8322 |
| DALYs | Global | Male | Dietary iron deficiency | 2000 | 348.7168 | 502.409 | 232.5038 |
| DALYs | Global | Both | Dietary iron deficiency | 2000 | 433.0119 | 620.5633 | 291.8738 |
| DALYs | Global | Female | Dietary iron deficiency | 2000 | 518.648 | 737.9412 | 351.5451 |
| DALYs | Global | Male | Dietary iron deficiency | 2001 | 345.7833 | 497.7253 | 231.2186 |
| DALYs | Global | Both | Dietary iron deficiency | 2001 | 429.7477 | 615.8804 | 289.3065 |
| DALYs | Global | Female | Dietary iron deficiency | 2001 | 514.9543 | 734.4958 | 348.6304 |
| DALYs | Global | Male | Dietary iron deficiency | 2002 | 341.7662 | 492.2815 | 228.7353 |
| DALYs | Global | Both | Dietary iron deficiency | 2002 | 425.6324 | 610.2241 | 286.3729 |
| DALYs | Global | Female | Dietary iron deficiency | 2002 | 510.6546 | 729.9949 | 345.9122 |
| DALYs | Global | Male | Dietary iron deficiency | 2003 | 337.3364 | 485.5397 | 225.7644 |
| DALYs | Global | Both | Dietary iron deficiency | 2003 | 421.2226 | 603.4239 | 283.6653 |
| DALYs | Global | Female | Dietary iron deficiency | 2003 | 506.186 | 724.9576 | 343.2555 |
| DALYs | Global | Male | Dietary iron deficiency | 2004 | 333.2251 | 479.7349 | 223.2292 |
| DALYs | Global | Both | Dietary iron deficiency | 2004 | 417.1077 | 597.4486 | 281.265 |
| DALYs | Global | Female | Dietary iron deficiency | 2004 | 501.9864 | 719.146 | 340.3701 |
| DALYs | Global | Male | Dietary iron deficiency | 2005 | 330.0914 | 475.0378 | 221.2542 |
| DALYs | Global | Both | Dietary iron deficiency | 2005 | 413.819 | 592.5996 | 279.5002 |
| DALYs | Global | Female | Dietary iron deficiency | 2005 | 498.4687 | 713.1142 | 338.0477 |
| DALYs | Global | Male | Dietary iron deficiency | 2006 | 327.3527 | 470.034 | 219.4909 |
| DALYs | Global | Both | Dietary iron deficiency | 2006 | 410.8478 | 588.6966 | 277.4061 |
| DALYs | Global | Female | Dietary iron deficiency | 2006 | 495.2142 | 708.0636 | 335.7615 |
| DALYs | Global | Male | Dietary iron deficiency | 2007 | 324.1949 | 465.2098 | 217.1068 |
| DALYs | Global | Both | Dietary iron deficiency | 2007 | 407.5949 | 585.6243 | 275.1762 |
| DALYs | Global | Female | Dietary iron deficiency | 2007 | 491.8478 | 703.4399 | 333.155 |
| DALYs | Global | Male | Dietary iron deficiency | 2008 | 321.0791 | 461.1917 | 214.765 |
| DALYs | Global | Both | Dietary iron deficiency | 2008 | 404.4947 | 581.37 | 272.8702 |
| DALYs | Global | Female | Dietary iron deficiency | 2008 | 488.7556 | 698.7276 | 331.0535 |
| DALYs | Global | Male | Dietary iron deficiency | 2009 | 318.3954 | 458.2182 | 212.3427 |
| DALYs | Global | Both | Dietary iron deficiency | 2009 | 401.9274 | 577.916 | 270.5348 |
| DALYs | Global | Female | Dietary iron deficiency | 2009 | 486.2855 | 695.2782 | 329.1446 |
| DALYs | Global | Male | Dietary iron deficiency | 2010 | 316.4645 | 455.5117 | 210.9369 |
| DALYs | Global | Both | Dietary iron deficiency | 2010 | 400.1173 | 575.9823 | 269.1323 |
| DALYs | Global | Female | Dietary iron deficiency | 2010 | 484.5682 | 693.6626 | 327.9235 |
| DALYs | Global | Male | Dietary iron deficiency | 2011 | 315.0605 | 453.0438 | 209.5963 |
| DALYs | Global | Both | Dietary iron deficiency | 2011 | 398.8693 | 574.1561 | 267.8515 |
| DALYs | Global | Female | Dietary iron deficiency | 2011 | 483.461 | 691.9009 | 326.7201 |
| DALYs | Global | Male | Dietary iron deficiency | 2012 | 313.6815 | 450.4816 | 208.6978 |
| DALYs | Global | Both | Dietary iron deficiency | 2012 | 397.7082 | 572.6893 | 267.1498 |
| DALYs | Global | Female | Dietary iron deficiency | 2012 | 482.5178 | 691.5953 | 326.1071 |
| DALYs | Global | Male | Dietary iron deficiency | 2013 | 312.3386 | 448.6611 | 207.563 |
| DALYs | Global | Both | Dietary iron deficiency | 2013 | 396.6503 | 572 | 266.2916 |
| DALYs | Global | Female | Dietary iron deficiency | 2013 | 481.7422 | 690.3444 | 325.8772 |
| DALYs | Global | Male | Dietary iron deficiency | 2014 | 311.0755 | 446.8919 | 206.915 |
| DALYs | Global | Both | Dietary iron deficiency | 2014 | 395.6864 | 570.5814 | 265.7798 |
| DALYs | Global | Female | Dietary iron deficiency | 2014 | 481.0728 | 690.2488 | 326.1018 |
| DALYs | Global | Male | Dietary iron deficiency | 2015 | 309.7621 | 445.3834 | 205.7062 |
| DALYs | Global | Both | Dietary iron deficiency | 2015 | 394.6269 | 569.392 | 265.575 |
| DALYs | Global | Female | Dietary iron deficiency | 2015 | 480.2651 | 689.7665 | 326.3655 |
| DALYs | Global | Male | Dietary iron deficiency | 2016 | 308.0035 | 443.5129 | 204.8276 |
| DALYs | Global | Both | Dietary iron deficiency | 2016 | 393.2438 | 568.6337 | 264.8928 |
| DALYs | Global | Female | Dietary iron deficiency | 2016 | 479.2639 | 687.6523 | 325.1193 |
| DALYs | Global | Male | Dietary iron deficiency | 2017 | 305.7454 | 440.4793 | 203.2539 |
| DALYs | Global | Both | Dietary iron deficiency | 2017 | 391.229 | 566.2842 | 262.976 |
| DALYs | Global | Female | Dietary iron deficiency | 2017 | 477.4973 | 685.1975 | 323.2448 |
| DALYs | Global | Male | Dietary iron deficiency | 2018 | 302.7279 | 437.2701 | 200.7048 |
| DALYs | Global | Both | Dietary iron deficiency | 2018 | 387.9294 | 560.7653 | 260.5189 |
| DALYs | Global | Female | Dietary iron deficiency | 2018 | 473.912 | 680.9551 | 320.8858 |
| DALYs | Global | Male | Dietary iron deficiency | 2019 | 298.8218 | 433.148 | 197.8943 |
| DALYs | Global | Both | Dietary iron deficiency | 2019 | 383.3828 | 553.4581 | 257.0536 |
| DALYs | Global | Female | Dietary iron deficiency | 2019 | 468.7196 | 672.87 | 317.2709 |
| DALYs | Global | Male | Iodine deficiency | 1990 | 40.92492 | 65.76052 | 25.36948 |
| DALYs | Global | Both | Iodine deficiency | 1990 | 46.8457 | 76.01194 | 28.64907 |
| DALYs | Global | Female | Iodine deficiency | 1990 | 52.87622 | 86.21474 | 32.41516 |
| DALYs | Global | Male | Iodine deficiency | 1991 | 39.65632 | 63.63032 | 24.3956 |
| DALYs | Global | Both | Iodine deficiency | 1991 | 45.95789 | 74.52893 | 28.08828 |
| DALYs | Global | Female | Iodine deficiency | 1991 | 52.38245 | 85.73789 | 32.12353 |
| DALYs | Global | Male | Iodine deficiency | 1992 | 38.41572 | 61.42909 | 23.66277 |
| DALYs | Global | Both | Iodine deficiency | 1992 | 45.05073 | 73.02274 | 27.49434 |
| DALYs | Global | Female | Iodine deficiency | 1992 | 51.81999 | 84.85079 | 31.62879 |
| DALYs | Global | Male | Iodine deficiency | 1993 | 37.24939 | 59.57912 | 22.98468 |
| DALYs | Global | Both | Iodine deficiency | 1993 | 44.16685 | 71.56659 | 27.07899 |
| DALYs | Global | Female | Iodine deficiency | 1993 | 51.22768 | 84.25791 | 31.13387 |
| DALYs | Global | Male | Iodine deficiency | 1994 | 36.19285 | 57.71875 | 22.17379 |
| DALYs | Global | Both | Iodine deficiency | 1994 | 43.33879 | 69.96861 | 26.68011 |
| DALYs | Global | Female | Iodine deficiency | 1994 | 50.63268 | 82.94841 | 30.79176 |
| DALYs | Global | Male | Iodine deficiency | 1995 | 35.31312 | 56.25391 | 21.69619 |
| DALYs | Global | Both | Iodine deficiency | 1995 | 42.61684 | 68.78965 | 26.31597 |
| DALYs | Global | Female | Iodine deficiency | 1995 | 50.06796 | 81.98696 | 30.16055 |
| DALYs | Global | Male | Iodine deficiency | 1996 | 34.32343 | 54.4662 | 21.16633 |
| DALYs | Global | Both | Iodine deficiency | 1996 | 41.70715 | 67.27838 | 25.63178 |
| DALYs | Global | Female | Iodine deficiency | 1996 | 49.23377 | 80.65396 | 29.72345 |
| DALYs | Global | Male | Iodine deficiency | 1997 | 33.10217 | 52.36521 | 20.45364 |
| DALYs | Global | Both | Iodine deficiency | 1997 | 40.4808 | 65.1673 | 24.81479 |
| DALYs | Global | Female | Iodine deficiency | 1997 | 47.99467 | 78.34541 | 28.80539 |
| DALYs | Global | Male | Iodine deficiency | 1998 | 31.87773 | 50.34656 | 19.61007 |
| DALYs | Global | Both | Iodine deficiency | 1998 | 39.21751 | 63.2819 | 24.11405 |
| DALYs | Global | Female | Iodine deficiency | 1998 | 46.68364 | 76.48946 | 28.09862 |
| DALYs | Global | Male | Iodine deficiency | 1999 | 30.90946 | 49.06655 | 19.10786 |
| DALYs | Global | Both | Iodine deficiency | 1999 | 38.19529 | 61.56252 | 23.34278 |
| DALYs | Global | Female | Iodine deficiency | 1999 | 45.59863 | 74.5732 | 27.3405 |
| DALYs | Global | Male | Iodine deficiency | 2000 | 30.45411 | 48.0671 | 18.73787 |
| DALYs | Global | Both | Iodine deficiency | 2000 | 37.72022 | 60.80874 | 23.01316 |
| DALYs | Global | Female | Iodine deficiency | 2000 | 45.09623 | 73.67207 | 26.93433 |
| DALYs | Global | Male | Iodine deficiency | 2001 | 30.40514 | 48.13373 | 18.61689 |
| DALYs | Global | Both | Iodine deficiency | 2001 | 37.6739 | 61.08238 | 22.96347 |
| DALYs | Global | Female | Iodine deficiency | 2001 | 45.04707 | 73.79688 | 26.938 |
| DALYs | Global | Male | Iodine deficiency | 2002 | 30.45814 | 48.4404 | 18.5543 |
| DALYs | Global | Both | Iodine deficiency | 2002 | 37.71193 | 61.20142 | 23.00959 |
| DALYs | Global | Female | Iodine deficiency | 2002 | 45.06646 | 74.21728 | 26.91563 |
| DALYs | Global | Male | Iodine deficiency | 2003 | 30.55298 | 48.84471 | 18.40506 |
| DALYs | Global | Both | Iodine deficiency | 2003 | 37.79068 | 61.92848 | 22.90277 |
| DALYs | Global | Female | Iodine deficiency | 2003 | 45.12607 | 74.85807 | 26.74628 |
| DALYs | Global | Male | Iodine deficiency | 2004 | 30.62165 | 49.30653 | 18.41568 |
| DALYs | Global | Both | Iodine deficiency | 2004 | 37.84418 | 62.24312 | 22.76815 |
| DALYs | Global | Female | Iodine deficiency | 2004 | 45.16168 | 75.58124 | 26.67816 |
| DALYs | Global | Male | Iodine deficiency | 2005 | 30.6243 | 49.37673 | 18.42411 |
| DALYs | Global | Both | Iodine deficiency | 2005 | 37.82222 | 62.38719 | 22.68771 |
| DALYs | Global | Female | Iodine deficiency | 2005 | 45.11224 | 75.77493 | 26.66431 |
| DALYs | Global | Male | Iodine deficiency | 2006 | 30.39394 | 49.05944 | 18.19959 |
| DALYs | Global | Both | Iodine deficiency | 2006 | 37.54692 | 61.90288 | 22.47828 |
| DALYs | Global | Female | Iodine deficiency | 2006 | 44.78943 | 75.36405 | 26.33629 |
| DALYs | Global | Male | Iodine deficiency | 2007 | 29.91055 | 48.4362 | 18.00329 |
| DALYs | Global | Both | Iodine deficiency | 2007 | 36.97186 | 61.11741 | 22.16399 |
| DALYs | Global | Female | Iodine deficiency | 2007 | 44.1203 | 74.38815 | 26.02344 |
| DALYs | Global | Male | Iodine deficiency | 2008 | 29.32269 | 47.51566 | 17.75237 |
| DALYs | Global | Both | Iodine deficiency | 2008 | 36.28425 | 59.94802 | 21.66954 |
| DALYs | Global | Female | Iodine deficiency | 2008 | 43.32995 | 73.40347 | 25.45135 |
| DALYs | Global | Male | Iodine deficiency | 2009 | 28.77149 | 46.81541 | 17.37752 |
| DALYs | Global | Both | Iodine deficiency | 2009 | 35.66836 | 59.10658 | 21.20645 |
| DALYs | Global | Female | Iodine deficiency | 2009 | 42.64551 | 72.24133 | 25.01235 |
| DALYs | Global | Male | Iodine deficiency | 2010 | 28.37624 | 46.30597 | 17.09952 |
| DALYs | Global | Both | Iodine deficiency | 2010 | 35.27527 | 58.63853 | 20.92985 |
| DALYs | Global | Female | Iodine deficiency | 2010 | 42.25158 | 71.55879 | 24.67943 |
| DALYs | Global | Male | Iodine deficiency | 2011 | 27.8535 | 45.60193 | 16.80415 |
| DALYs | Global | Both | Iodine deficiency | 2011 | 34.85968 | 58.05321 | 20.57691 |
| DALYs | Global | Female | Iodine deficiency | 2011 | 41.94142 | 71.42714 | 24.3851 |
| DALYs | Global | Male | Iodine deficiency | 2012 | 26.94013 | 44.45023 | 16.09085 |
| DALYs | Global | Both | Iodine deficiency | 2012 | 34.12412 | 57.30199 | 20.07906 |
| DALYs | Global | Female | Iodine deficiency | 2012 | 41.38179 | 71.00026 | 24.00457 |
| DALYs | Global | Male | Iodine deficiency | 2013 | 25.87505 | 42.99291 | 15.33141 |
| DALYs | Global | Both | Iodine deficiency | 2013 | 33.26057 | 56.40164 | 19.48257 |
| DALYs | Global | Female | Iodine deficiency | 2013 | 40.71773 | 70.49178 | 23.24034 |
| DALYs | Global | Male | Iodine deficiency | 2014 | 24.94046 | 41.55415 | 14.56997 |
| DALYs | Global | Both | Iodine deficiency | 2014 | 32.50861 | 55.50377 | 18.67584 |
| DALYs | Global | Female | Iodine deficiency | 2014 | 40.1465 | 69.77149 | 22.649 |
| DALYs | Global | Male | Iodine deficiency | 2015 | 24.35625 | 40.83305 | 14.08704 |
| DALYs | Global | Both | Iodine deficiency | 2015 | 32.02073 | 54.88281 | 18.35961 |
| DALYs | Global | Female | Iodine deficiency | 2015 | 39.75533 | 69.32219 | 22.32171 |
| DALYs | Global | Male | Iodine deficiency | 2016 | 23.92439 | 40.15697 | 13.80367 |
| DALYs | Global | Both | Iodine deficiency | 2016 | 31.53436 | 54.21696 | 17.98475 |
| DALYs | Global | Female | Iodine deficiency | 2016 | 39.21557 | 68.66951 | 21.93847 |
| DALYs | Global | Male | Iodine deficiency | 2017 | 23.58055 | 39.77081 | 13.70071 |
| DALYs | Global | Both | Iodine deficiency | 2017 | 31.13762 | 53.72532 | 17.70699 |
| DALYs | Global | Female | Iodine deficiency | 2017 | 38.76769 | 67.96598 | 21.6112 |
| DALYs | Global | Male | Iodine deficiency | 2018 | 23.37579 | 39.61876 | 13.56449 |
| DALYs | Global | Both | Iodine deficiency | 2018 | 30.96511 | 53.42635 | 17.59933 |
| DALYs | Global | Female | Iodine deficiency | 2018 | 38.62901 | 67.94524 | 21.55298 |
| DALYs | Global | Male | Iodine deficiency | 2019 | 23.06721 | 39.11294 | 13.26284 |
| DALYs | Global | Both | Iodine deficiency | 2019 | 30.69678 | 53.12892 | 17.32121 |
| DALYs | Global | Female | Iodine deficiency | 2019 | 38.40271 | 67.92254 | 21.41632 |
| DALYs | Global | Female | Vitamin A deficiency | 1990 | 26.30429 | 37.37333 | 17.82604 |
| DALYs | Global | Both | Vitamin A deficiency | 1990 | 31.94694 | 45.29591 | 22.11394 |
| DALYs | Global | Male | Vitamin A deficiency | 1990 | 37.29006 | 53.04386 | 25.60299 |
| DALYs | Global | Female | Vitamin A deficiency | 1991 | 25.81552 | 36.69417 | 17.36249 |
| DALYs | Global | Both | Vitamin A deficiency | 1991 | 31.31326 | 44.39571 | 21.53861 |
| DALYs | Global | Male | Vitamin A deficiency | 1991 | 36.51092 | 51.98319 | 25.09858 |
| DALYs | Global | Female | Vitamin A deficiency | 1992 | 25.39193 | 35.93439 | 17.13288 |
| DALYs | Global | Both | Vitamin A deficiency | 1992 | 30.7401 | 43.6555 | 21.18196 |
| DALYs | Global | Male | Vitamin A deficiency | 1992 | 35.78805 | 51.0829 | 24.72298 |
| DALYs | Global | Female | Vitamin A deficiency | 1993 | 25.04882 | 35.42679 | 16.94209 |
| DALYs | Global | Both | Vitamin A deficiency | 1993 | 30.24169 | 42.93707 | 20.92769 |
| DALYs | Global | Male | Vitamin A deficiency | 1993 | 35.13508 | 50.16261 | 24.26312 |
| DALYs | Global | Female | Vitamin A deficiency | 1994 | 24.79453 | 35.04994 | 16.83206 |
| DALYs | Global | Both | Vitamin A deficiency | 1994 | 29.82245 | 42.33291 | 20.69718 |
| DALYs | Global | Male | Vitamin A deficiency | 1994 | 34.55329 | 49.51981 | 23.83105 |
| DALYs | Global | Female | Vitamin A deficiency | 1995 | 24.64047 | 35.00414 | 16.70323 |
| DALYs | Global | Both | Vitamin A deficiency | 1995 | 29.49179 | 41.8809 | 20.39137 |
| DALYs | Global | Male | Vitamin A deficiency | 1995 | 34.05035 | 48.8251 | 23.43347 |
| DALYs | Global | Female | Vitamin A deficiency | 1996 | 24.57479 | 34.80224 | 16.69993 |
| DALYs | Global | Both | Vitamin A deficiency | 1996 | 29.21344 | 41.24975 | 20.20179 |
| DALYs | Global | Male | Vitamin A deficiency | 1996 | 33.56713 | 47.7971 | 23.18313 |
| DALYs | Global | Female | Vitamin A deficiency | 1997 | 24.57261 | 34.70254 | 16.74877 |
| DALYs | Global | Both | Vitamin A deficiency | 1997 | 28.96664 | 41.00584 | 20.11696 |
| DALYs | Global | Male | Vitamin A deficiency | 1997 | 33.08684 | 47.2365 | 22.84425 |
| DALYs | Global | Female | Vitamin A deficiency | 1998 | 24.60484 | 34.68006 | 16.83061 |
| DALYs | Global | Both | Vitamin A deficiency | 1998 | 28.73335 | 40.52078 | 19.92239 |
| DALYs | Global | Male | Vitamin A deficiency | 1998 | 32.60179 | 46.30578 | 22.50376 |
| DALYs | Global | Female | Vitamin A deficiency | 1999 | 24.64165 | 34.86408 | 16.7585 |
| DALYs | Global | Both | Vitamin A deficiency | 1999 | 28.51669 | 40.17551 | 19.70865 |
| DALYs | Global | Male | Vitamin A deficiency | 1999 | 32.14586 | 45.66389 | 22.18038 |
| DALYs | Global | Female | Vitamin A deficiency | 2000 | 24.64298 | 34.77836 | 16.8099 |
| DALYs | Global | Both | Vitamin A deficiency | 2000 | 28.30732 | 39.7922 | 19.51693 |
| DALYs | Global | Male | Vitamin A deficiency | 2000 | 31.73813 | 45.03848 | 21.91583 |
| DALYs | Global | Female | Vitamin A deficiency | 2001 | 24.54998 | 34.69731 | 16.69472 |
| DALYs | Global | Both | Vitamin A deficiency | 2001 | 28.00485 | 39.29583 | 19.29745 |
| DALYs | Global | Male | Vitamin A deficiency | 2001 | 31.23885 | 44.19626 | 21.55574 |
| DALYs | Global | Female | Vitamin A deficiency | 2002 | 24.35006 | 34.49228 | 16.49944 |
| DALYs | Global | Both | Vitamin A deficiency | 2002 | 27.55924 | 38.72778 | 19.01497 |
| DALYs | Global | Male | Vitamin A deficiency | 2002 | 30.56299 | 43.28042 | 21.14573 |
| DALYs | Global | Female | Vitamin A deficiency | 2003 | 24.0719 | 34.11879 | 16.23092 |
| DALYs | Global | Both | Vitamin A deficiency | 2003 | 27.02159 | 37.85088 | 18.59125 |
| DALYs | Global | Male | Vitamin A deficiency | 2003 | 29.78246 | 42.11221 | 20.55644 |
| DALYs | Global | Female | Vitamin A deficiency | 2004 | 23.76335 | 33.78337 | 16.02506 |
| DALYs | Global | Both | Vitamin A deficiency | 2004 | 26.46691 | 37.05647 | 18.22877 |
| DALYs | Global | Male | Vitamin A deficiency | 2004 | 28.99773 | 40.81646 | 20.017 |
| DALYs | Global | Female | Vitamin A deficiency | 2005 | 23.46209 | 33.25256 | 15.83795 |
| DALYs | Global | Both | Vitamin A deficiency | 2005 | 25.95727 | 36.33537 | 17.89107 |
| DALYs | Global | Male | Vitamin A deficiency | 2005 | 28.29367 | 39.65683 | 19.43133 |
| DALYs | Global | Female | Vitamin A deficiency | 2006 | 23.06739 | 32.82299 | 15.63386 |
| DALYs | Global | Both | Vitamin A deficiency | 2006 | 25.40737 | 35.60461 | 17.4639 |
| DALYs | Global | Male | Vitamin A deficiency | 2006 | 27.59941 | 38.6054 | 18.90315 |
| DALYs | Global | Female | Vitamin A deficiency | 2007 | 22.52301 | 32.03233 | 15.14138 |
| DALYs | Global | Both | Vitamin A deficiency | 2007 | 24.73674 | 34.72389 | 16.98899 |
| DALYs | Global | Male | Vitamin A deficiency | 2007 | 26.81177 | 37.45533 | 18.22457 |
| DALYs | Global | Female | Vitamin A deficiency | 2008 | 21.88708 | 31.26356 | 14.75662 |
| DALYs | Global | Both | Vitamin A deficiency | 2008 | 23.99892 | 33.69267 | 16.4356 |
| DALYs | Global | Male | Vitamin A deficiency | 2008 | 25.97976 | 36.44312 | 17.72528 |
| DALYs | Global | Female | Vitamin A deficiency | 2009 | 21.22449 | 30.10389 | 14.3497 |
| DALYs | Global | Both | Vitamin A deficiency | 2009 | 23.25055 | 32.52642 | 15.86199 |
| DALYs | Global | Male | Vitamin A deficiency | 2009 | 25.15238 | 35.21074 | 17.13456 |
| DALYs | Global | Female | Vitamin A deficiency | 2010 | 20.61701 | 29.37833 | 13.90589 |
| DALYs | Global | Both | Vitamin A deficiency | 2010 | 22.56024 | 31.51853 | 15.31278 |
| DALYs | Global | Male | Vitamin A deficiency | 2010 | 24.38572 | 34.08273 | 16.57581 |
| DALYs | Global | Female | Vitamin A deficiency | 2011 | 19.98545 | 28.36271 | 13.44581 |
| DALYs | Global | Both | Vitamin A deficiency | 2011 | 21.8422 | 30.43526 | 14.81723 |
| DALYs | Global | Male | Vitamin A deficiency | 2011 | 23.58754 | 32.78769 | 16.04174 |
| DALYs | Global | Female | Vitamin A deficiency | 2012 | 19.245 | 27.24157 | 12.90933 |
| DALYs | Global | Both | Vitamin A deficiency | 2012 | 21.01923 | 29.3521 | 14.26685 |
| DALYs | Global | Male | Vitamin A deficiency | 2012 | 22.6878 | 31.70708 | 15.47389 |
| DALYs | Global | Female | Vitamin A deficiency | 2013 | 18.48206 | 26.26127 | 12.41645 |
| DALYs | Global | Both | Vitamin A deficiency | 2013 | 20.17551 | 28.0892 | 13.75199 |
| DALYs | Global | Male | Vitamin A deficiency | 2013 | 21.7687 | 30.32347 | 14.93218 |
| DALYs | Global | Female | Vitamin A deficiency | 2014 | 17.758 | 25.185 | 11.97279 |
| DALYs | Global | Both | Vitamin A deficiency | 2014 | 19.37836 | 27.1074 | 13.16061 |
| DALYs | Global | Male | Vitamin A deficiency | 2014 | 20.90338 | 29.27472 | 14.33645 |
| DALYs | Global | Female | Vitamin A deficiency | 2015 | 17.13229 | 24.22117 | 11.57189 |
| DALYs | Global | Both | Vitamin A deficiency | 2015 | 18.68561 | 26.29797 | 12.69819 |
| DALYs | Global | Male | Vitamin A deficiency | 2015 | 20.14804 | 28.08139 | 13.81385 |
| DALYs | Global | Female | Vitamin A deficiency | 2016 | 16.58708 | 23.37493 | 11.20903 |
| DALYs | Global | Both | Vitamin A deficiency | 2016 | 18.07453 | 25.22324 | 12.2689 |
| DALYs | Global | Male | Vitamin A deficiency | 2016 | 19.47537 | 27.11445 | 13.31883 |
| DALYs | Global | Female | Vitamin A deficiency | 2017 | 16.13696 | 22.73652 | 10.9228 |
| DALYs | Global | Both | Vitamin A deficiency | 2017 | 17.56915 | 24.45465 | 11.97243 |
| DALYs | Global | Male | Vitamin A deficiency | 2017 | 18.91839 | 26.24354 | 12.98547 |
| DALYs | Global | Female | Vitamin A deficiency | 2018 | 15.80038 | 22.26293 | 10.77762 |
| DALYs | Global | Both | Vitamin A deficiency | 2018 | 17.19203 | 23.91902 | 11.70354 |
| DALYs | Global | Male | Vitamin A deficiency | 2018 | 18.50344 | 25.72451 | 12.62676 |
| DALYs | Global | Female | Vitamin A deficiency | 2019 | 15.54358 | 21.80347 | 10.54883 |
| DALYs | Global | Both | Vitamin A deficiency | 2019 | 16.91073 | 23.47367 | 11.52898 |
| DALYs | Global | Male | Vitamin A deficiency | 2019 | 18.19922 | 25.16216 | 12.41292 |
| Incidence | Global | Male | Dietary iron deficiency | 1990 | No data | No data | No data |
| Incidence | Global | Female | Dietary iron deficiency | 1990 | No data | No data | No data |
| Incidence | Global | Both | Dietary iron deficiency | 1990 | No data | No data | No data |
| Incidence | Global | Male | Dietary iron deficiency | 1991 | No data | No data | No data |
| Incidence | Global | Female | Dietary iron deficiency | 1991 | No data | No data | No data |
| Incidence | Global | Both | Dietary iron deficiency | 1991 | No data | No data | No data |
| Incidence | Global | Male | Dietary iron deficiency | 1992 | No data | No data | No data |
| Incidence | Global | Female | Dietary iron deficiency | 1992 | No data | No data | No data |
| Incidence | Global | Both | Dietary iron deficiency | 1992 | No data | No data | No data |
| Incidence | Global | Male | Dietary iron deficiency | 1993 | No data | No data | No data |
| Incidence | Global | Female | Dietary iron deficiency | 1993 | No data | No data | No data |
| Incidence | Global | Both | Dietary iron deficiency | 1993 | No data | No data | No data |
| Incidence | Global | Male | Dietary iron deficiency | 1994 | No data | No data | No data |
| Incidence | Global | Female | Dietary iron deficiency | 1994 | No data | No data | No data |
| Incidence | Global | Both | Dietary iron deficiency | 1994 | No data | No data | No data |
| Incidence | Global | Male | Dietary iron deficiency | 1995 | No data | No data | No data |
| Incidence | Global | Female | Dietary iron deficiency | 1995 | No data | No data | No data |
| Incidence | Global | Both | Dietary iron deficiency | 1995 | No data | No data | No data |
| Incidence | Global | Male | Dietary iron deficiency | 1996 | No data | No data | No data |
| Incidence | Global | Female | Dietary iron deficiency | 1996 | No data | No data | No data |
| Incidence | Global | Both | Dietary iron deficiency | 1996 | No data | No data | No data |
| Incidence | Global | Male | Dietary iron deficiency | 1997 | No data | No data | No data |
| Incidence | Global | Female | Dietary iron deficiency | 1997 | No data | No data | No data |
| Incidence | Global | Both | Dietary iron deficiency | 1997 | No data | No data | No data |
| Incidence | Global | Male | Dietary iron deficiency | 1998 | No data | No data | No data |
| Incidence | Global | Female | Dietary iron deficiency | 1998 | No data | No data | No data |
| Incidence | Global | Both | Dietary iron deficiency | 1998 | No data | No data | No data |
| Incidence | Global | Male | Dietary iron deficiency | 1999 | No data | No data | No data |
| Incidence | Global | Female | Dietary iron deficiency | 1999 | No data | No data | No data |
| Incidence | Global | Both | Dietary iron deficiency | 1999 | No data | No data | No data |
| Incidence | Global | Male | Dietary iron deficiency | 2000 | No data | No data | No data |
| Incidence | Global | Female | Dietary iron deficiency | 2000 | No data | No data | No data |
| Incidence | Global | Both | Dietary iron deficiency | 2000 | No data | No data | No data |
| Incidence | Global | Male | Dietary iron deficiency | 2001 | No data | No data | No data |
| Incidence | Global | Female | Dietary iron deficiency | 2001 | No data | No data | No data |
| Incidence | Global | Both | Dietary iron deficiency | 2001 | No data | No data | No data |
| Incidence | Global | Male | Dietary iron deficiency | 2002 | No data | No data | No data |
| Incidence | Global | Female | Dietary iron deficiency | 2002 | No data | No data | No data |
| Incidence | Global | Both | Dietary iron deficiency | 2002 | No data | No data | No data |
| Incidence | Global | Male | Dietary iron deficiency | 2003 | No data | No data | No data |
| Incidence | Global | Female | Dietary iron deficiency | 2003 | No data | No data | No data |
| Incidence | Global | Both | Dietary iron deficiency | 2003 | No data | No data | No data |
| Incidence | Global | Male | Dietary iron deficiency | 2004 | No data | No data | No data |
| Incidence | Global | Female | Dietary iron deficiency | 2004 | No data | No data | No data |
| Incidence | Global | Both | Dietary iron deficiency | 2004 | No data | No data | No data |
| Incidence | Global | Male | Dietary iron deficiency | 2005 | No data | No data | No data |
| Incidence | Global | Female | Dietary iron deficiency | 2005 | No data | No data | No data |
| Incidence | Global | Both | Dietary iron deficiency | 2005 | No data | No data | No data |
| Incidence | Global | Male | Dietary iron deficiency | 2006 | No data | No data | No data |
| Incidence | Global | Female | Dietary iron deficiency | 2006 | No data | No data | No data |
| Incidence | Global | Both | Dietary iron deficiency | 2006 | No data | No data | No data |
| Incidence | Global | Male | Dietary iron deficiency | 2007 | No data | No data | No data |
| Incidence | Global | Female | Dietary iron deficiency | 2007 | No data | No data | No data |
| Incidence | Global | Both | Dietary iron deficiency | 2007 | No data | No data | No data |
| Incidence | Global | Male | Dietary iron deficiency | 2008 | No data | No data | No data |
| Incidence | Global | Female | Dietary iron deficiency | 2008 | No data | No data | No data |
| Incidence | Global | Both | Dietary iron deficiency | 2008 | No data | No data | No data |
| Incidence | Global | Male | Dietary iron deficiency | 2009 | No data | No data | No data |
| Incidence | Global | Female | Dietary iron deficiency | 2009 | No data | No data | No data |
| Incidence | Global | Both | Dietary iron deficiency | 2009 | No data | No data | No data |
| Incidence | Global | Male | Dietary iron deficiency | 2010 | No data | No data | No data |
| Incidence | Global | Female | Dietary iron deficiency | 2010 | No data | No data | No data |
| Incidence | Global | Both | Dietary iron deficiency | 2010 | No data | No data | No data |
| Incidence | Global | Male | Dietary iron deficiency | 2011 | No data | No data | No data |
| Incidence | Global | Female | Dietary iron deficiency | 2011 | No data | No data | No data |
| Incidence | Global | Both | Dietary iron deficiency | 2011 | No data | No data | No data |
| Incidence | Global | Male | Dietary iron deficiency | 2012 | No data | No data | No data |
| Incidence | Global | Female | Dietary iron deficiency | 2012 | No data | No data | No data |
| Incidence | Global | Both | Dietary iron deficiency | 2012 | No data | No data | No data |
| Incidence | Global | Male | Dietary iron deficiency | 2013 | No data | No data | No data |
| Incidence | Global | Female | Dietary iron deficiency | 2013 | No data | No data | No data |
| Incidence | Global | Both | Dietary iron deficiency | 2013 | No data | No data | No data |
| Incidence | Global | Male | Dietary iron deficiency | 2014 | No data | No data | No data |
| Incidence | Global | Female | Dietary iron deficiency | 2014 | No data | No data | No data |
| Incidence | Global | Both | Dietary iron deficiency | 2014 | No data | No data | No data |
| Incidence | Global | Male | Dietary iron deficiency | 2015 | No data | No data | No data |
| Incidence | Global | Female | Dietary iron deficiency | 2015 | No data | No data | No data |
| Incidence | Global | Both | Dietary iron deficiency | 2015 | No data | No data | No data |
| Incidence | Global | Male | Dietary iron deficiency | 2016 | No data | No data | No data |
| Incidence | Global | Female | Dietary iron deficiency | 2016 | No data | No data | No data |
| Incidence | Global | Both | Dietary iron deficiency | 2016 | No data | No data | No data |
| Incidence | Global | Male | Dietary iron deficiency | 2017 | No data | No data | No data |
| Incidence | Global | Female | Dietary iron deficiency | 2017 | No data | No data | No data |
| Incidence | Global | Both | Dietary iron deficiency | 2017 | No data | No data | No data |
| Incidence | Global | Male | Dietary iron deficiency | 2018 | No data | No data | No data |
| Incidence | Global | Female | Dietary iron deficiency | 2018 | No data | No data | No data |
| Incidence | Global | Both | Dietary iron deficiency | 2018 | No data | No data | No data |
| Incidence | Global | Male | Dietary iron deficiency | 2019 | No data | No data | No data |
| Incidence | Global | Female | Dietary iron deficiency | 2019 | No data | No data | No data |
| Incidence | Global | Both | Dietary iron deficiency | 2019 | No data | No data | No data |
| Incidence | Global | Male | Iodine deficiency | 1990 | 106.7492 | 129.4235 | 86.77909 |
| Incidence | Global | Both | Iodine deficiency | 1990 | 129.0933 | 156.7226 | 105.1989 |
| Incidence | Global | Female | Iodine deficiency | 1990 | 152.1105 | 183.9451 | 125.015 |
| Incidence | Global | Male | Iodine deficiency | 1991 | 103.5183 | 126.4883 | 84.34043 |
| Incidence | Global | Both | Iodine deficiency | 1991 | 127.3385 | 154.505 | 104.4932 |
| Incidence | Global | Female | Iodine deficiency | 1991 | 151.9287 | 183.8617 | 124.6084 |
| Incidence | Global | Male | Iodine deficiency | 1992 | 100.3132 | 122.4255 | 81.97109 |
| Incidence | Global | Both | Iodine deficiency | 1992 | 125.4201 | 152.0483 | 102.5405 |
| Incidence | Global | Female | Iodine deficiency | 1992 | 151.3871 | 184.2059 | 123.7573 |
| Incidence | Global | Male | Iodine deficiency | 1993 | 97.32748 | 118.9161 | 79.40114 |
| Incidence | Global | Both | Iodine deficiency | 1993 | 123.4837 | 150.6446 | 100.8672 |
| Incidence | Global | Female | Iodine deficiency | 1993 | 150.5768 | 182.932 | 122.6037 |
| Incidence | Global | Male | Iodine deficiency | 1994 | 94.74777 | 115.998 | 76.65586 |
| Incidence | Global | Both | Iodine deficiency | 1994 | 121.6703 | 149.1584 | 98.84098 |
| Incidence | Global | Female | Iodine deficiency | 1994 | 149.5849 | 182.3732 | 121.5569 |
| Incidence | Global | Male | Iodine deficiency | 1995 | 92.73766 | 114.2305 | 74.7256 |
| Incidence | Global | Both | Iodine deficiency | 1995 | 120.0902 | 147.3933 | 97.01989 |
| Incidence | Global | Female | Iodine deficiency | 1995 | 148.4652 | 181.4756 | 120.3378 |
| Incidence | Global | Male | Iodine deficiency | 1996 | 90.58561 | 111.715 | 72.96721 |
| Incidence | Global | Both | Iodine deficiency | 1996 | 118.0371 | 144.6752 | 95.40535 |
| Incidence | Global | Female | Iodine deficiency | 1996 | 146.5239 | 179.1823 | 118.7763 |
| Incidence | Global | Male | Iodine deficiency | 1997 | 87.85948 | 108.3349 | 70.76741 |
| Incidence | Global | Both | Iodine deficiency | 1997 | 115.1945 | 141.1956 | 93.16206 |
| Incidence | Global | Female | Iodine deficiency | 1997 | 143.5696 | 175.1172 | 116.4022 |
| Incidence | Global | Male | Iodine deficiency | 1998 | 85.11703 | 105.1775 | 68.57779 |
| Incidence | Global | Both | Iodine deficiency | 1998 | 112.2316 | 137.457 | 90.81375 |
| Incidence | Global | Female | Iodine deficiency | 1998 | 140.3868 | 171.1089 | 113.8769 |
| Incidence | Global | Male | Iodine deficiency | 1999 | 82.9456 | 102.4555 | 66.78384 |
| Incidence | Global | Both | Iodine deficiency | 1999 | 109.8529 | 134.2823 | 88.94388 |
| Incidence | Global | Female | Iodine deficiency | 1999 | 137.8009 | 167.7195 | 112.0191 |
| Incidence | Global | Male | Iodine deficiency | 2000 | 81.95691 | 101.4808 | 65.86437 |
| Incidence | Global | Both | Iodine deficiency | 2000 | 108.7884 | 132.9963 | 88.0628 |
| Incidence | Global | Female | Iodine deficiency | 2000 | 136.6659 | 166.2307 | 111.177 |
| Incidence | Global | Male | Iodine deficiency | 2001 | 82.41482 | 102.0894 | 66.26626 |
| Incidence | Global | Both | Iodine deficiency | 2001 | 109.3788 | 133.9253 | 88.50931 |
| Incidence | Global | Female | Iodine deficiency | 2001 | 137.404 | 167.7136 | 111.6462 |
| Incidence | Global | Male | Iodine deficiency | 2002 | 83.78366 | 103.8223 | 67.27661 |
| Incidence | Global | Both | Iodine deficiency | 2002 | 111.0196 | 136.0584 | 89.74566 |
| Incidence | Global | Female | Iodine deficiency | 2002 | 139.3396 | 170.1841 | 113.0525 |
| Incidence | Global | Male | Iodine deficiency | 2003 | 85.49652 | 105.8717 | 68.55349 |
| Incidence | Global | Both | Iodine deficiency | 2003 | 113.0384 | 138.1261 | 91.07775 |
| Incidence | Global | Female | Iodine deficiency | 2003 | 141.6903 | 172.9865 | 114.5512 |
| Incidence | Global | Male | Iodine deficiency | 2004 | 86.98321 | 107.7048 | 69.79314 |
| Incidence | Global | Both | Iodine deficiency | 2004 | 114.7561 | 140.3844 | 92.42462 |
| Incidence | Global | Female | Iodine deficiency | 2004 | 143.6619 | 175.351 | 115.7571 |
| Incidence | Global | Male | Iodine deficiency | 2005 | 87.65062 | 108.5022 | 70.42292 |
| Incidence | Global | Both | Iodine deficiency | 2005 | 115.4704 | 141.3023 | 92.98201 |
| Incidence | Global | Female | Iodine deficiency | 2005 | 144.438 | 176.3293 | 116.0751 |
| Incidence | Global | Male | Iodine deficiency | 2006 | 87.37467 | 108.1204 | 70.25875 |
| Incidence | Global | Both | Iodine deficiency | 2006 | 115.0454 | 140.9248 | 92.59949 |
| Incidence | Global | Female | Iodine deficiency | 2006 | 143.8673 | 175.5882 | 115.5263 |
| Incidence | Global | Male | Iodine deficiency | 2007 | 86.63162 | 106.9627 | 69.65027 |
| Incidence | Global | Both | Iodine deficiency | 2007 | 114.0752 | 139.8754 | 91.83475 |
| Incidence | Global | Female | Iodine deficiency | 2007 | 142.6636 | 174.3612 | 114.6214 |
| Incidence | Global | Male | Iodine deficiency | 2008 | 85.72044 | 106.0238 | 68.95432 |
| Incidence | Global | Both | Iodine deficiency | 2008 | 112.9515 | 138.7697 | 90.91828 |
| Incidence | Global | Female | Iodine deficiency | 2008 | 141.317 | 172.5465 | 113.6335 |
| Incidence | Global | Male | Iodine deficiency | 2009 | 84.9137 | 105.1193 | 68.34136 |
| Incidence | Global | Both | Iodine deficiency | 2009 | 112.0363 | 137.756 | 90.10851 |
| Incidence | Global | Female | Iodine deficiency | 2009 | 140.2819 | 171.5876 | 112.9421 |
| Incidence | Global | Male | Iodine deficiency | 2010 | 84.44977 | 104.5667 | 68.06484 |
| Incidence | Global | Both | Iodine deficiency | 2010 | 111.6506 | 137.5127 | 89.77915 |
| Incidence | Global | Female | Iodine deficiency | 2010 | 139.9709 | 171.5467 | 112.7123 |
| Incidence | Global | Male | Iodine deficiency | 2011 | 83.98505 | 103.98 | 67.45841 |
| Incidence | Global | Both | Iodine deficiency | 2011 | 111.596 | 137.4521 | 89.66319 |
| Incidence | Global | Female | Iodine deficiency | 2011 | 140.341 | 172.1304 | 113.0447 |
| Incidence | Global | Male | Iodine deficiency | 2012 | 83.14863 | 103.0413 | 66.79027 |
| Incidence | Global | Both | Iodine deficiency | 2012 | 111.4583 | 137.3993 | 89.48363 |
| Incidence | Global | Female | Iodine deficiency | 2012 | 140.9266 | 172.8925 | 113.5457 |
| Incidence | Global | Male | Iodine deficiency | 2013 | 82.13025 | 102.0531 | 65.89589 |
| Incidence | Global | Both | Iodine deficiency | 2013 | 111.222 | 137.1 | 89.3398 |
| Incidence | Global | Female | Iodine deficiency | 2013 | 141.5005 | 173.61 | 114.0929 |
| Incidence | Global | Male | Iodine deficiency | 2014 | 81.13111 | 101.11 | 65.08831 |
| Incidence | Global | Both | Iodine deficiency | 2014 | 110.8817 | 136.9543 | 89.11356 |
| Incidence | Global | Female | Iodine deficiency | 2014 | 141.8451 | 173.9759 | 114.2212 |
| Incidence | Global | Male | Iodine deficiency | 2015 | 80.35296 | 100.134 | 64.23179 |
| Incidence | Global | Both | Iodine deficiency | 2015 | 110.4241 | 136.4742 | 88.73154 |
| Incidence | Global | Female | Iodine deficiency | 2015 | 141.7275 | 173.9893 | 114.1372 |
| Incidence | Global | Male | Iodine deficiency | 2016 | 79.71231 | 99.01085 | 63.75243 |
| Incidence | Global | Both | Iodine deficiency | 2016 | 109.5608 | 135.2975 | 87.92417 |
| Incidence | Global | Female | Iodine deficiency | 2016 | 140.6468 | 172.6315 | 113.1988 |
| Incidence | Global | Male | Iodine deficiency | 2017 | 79.17021 | 98.32896 | 63.30299 |
| Incidence | Global | Both | Iodine deficiency | 2017 | 108.8229 | 134.3676 | 87.29996 |
| Incidence | Global | Female | Iodine deficiency | 2017 | 139.7191 | 171.495 | 112.472 |
| Incidence | Global | Male | Iodine deficiency | 2018 | 78.6724 | 97.81145 | 62.87908 |
| Incidence | Global | Both | Iodine deficiency | 2018 | 108.5281 | 133.8893 | 86.86589 |
| Incidence | Global | Female | Iodine deficiency | 2018 | 139.6464 | 170.9929 | 112.5406 |
| Incidence | Global | Male | Iodine deficiency | 2019 | 78.09877 | 97.14128 | 62.55444 |
| Incidence | Global | Both | Iodine deficiency | 2019 | 108.3239 | 133.339 | 86.83091 |
| Incidence | Global | Female | Iodine deficiency | 2019 | 139.8367 | 171.8475 | 112.5158 |
| Incidence | Global | Female | Vitamin A deficiency | 1990 | 13456.51 | 14141.38 | 12848.51 |
| Incidence | Global | Both | Vitamin A deficiency | 1990 | 17323.23 | 18138.92 | 16526.51 |
| Incidence | Global | Male | Vitamin A deficiency | 1990 | 21073.77 | 22557.32 | 19711.46 |
| Incidence | Global | Female | Vitamin A deficiency | 1991 | 13325.45 | 13993.55 | 12721.85 |
| Incidence | Global | Both | Vitamin A deficiency | 1991 | 17063.93 | 17854.17 | 16290.5 |
| Incidence | Global | Male | Vitamin A deficiency | 1991 | 20686.7 | 22156.18 | 19316.36 |
| Incidence | Global | Female | Vitamin A deficiency | 1992 | 13217.18 | 13887.14 | 12623.15 |
| Incidence | Global | Both | Vitamin A deficiency | 1992 | 16863.27 | 17663.36 | 16107.66 |
| Incidence | Global | Male | Vitamin A deficiency | 1992 | 20393.41 | 21840.45 | 19033.98 |
| Incidence | Global | Female | Vitamin A deficiency | 1993 | 12798.73 | 13442.59 | 12218.47 |
| Incidence | Global | Both | Vitamin A deficiency | 1993 | 16212.75 | 16986.94 | 15514.39 |
| Incidence | Global | Male | Vitamin A deficiency | 1993 | 19515.71 | 20886.21 | 18201.29 |
| Incidence | Global | Female | Vitamin A deficiency | 1994 | 12427.08 | 13026.55 | 11871.95 |
| Incidence | Global | Both | Vitamin A deficiency | 1994 | 15643.83 | 16369.95 | 14953.08 |
| Incidence | Global | Male | Vitamin A deficiency | 1994 | 18754.45 | 20066.89 | 17484.22 |
| Incidence | Global | Female | Vitamin A deficiency | 1995 | 12180.7 | 12784.32 | 11642.84 |
| Incidence | Global | Both | Vitamin A deficiency | 1995 | 15232.28 | 15927.14 | 14563.21 |
| Incidence | Global | Male | Vitamin A deficiency | 1995 | 18182.36 | 19435.98 | 16961.75 |
| Incidence | Global | Female | Vitamin A deficiency | 1996 | 11883.9 | 12484.02 | 11372.02 |
| Incidence | Global | Both | Vitamin A deficiency | 1996 | 14806.46 | 15489.4 | 14166.08 |
| Incidence | Global | Male | Vitamin A deficiency | 1996 | 17631.43 | 18871.11 | 16445.74 |
| Incidence | Global | Female | Vitamin A deficiency | 1997 | 11567.73 | 12130.87 | 11082.82 |
| Incidence | Global | Both | Vitamin A deficiency | 1997 | 14316.25 | 14981.96 | 13689.06 |
| Incidence | Global | Male | Vitamin A deficiency | 1997 | 16973.03 | 18162.73 | 15811.67 |
| Incidence | Global | Female | Vitamin A deficiency | 1998 | 11374.25 | 11922.5 | 10908.16 |
| Incidence | Global | Both | Vitamin A deficiency | 1998 | 13979.77 | 14614.04 | 13355.4 |
| Incidence | Global | Male | Vitamin A deficiency | 1998 | 16498.76 | 17667.64 | 15323.17 |
| Incidence | Global | Female | Vitamin A deficiency | 1999 | 11392.64 | 11927.18 | 10918.86 |
| Incidence | Global | Both | Vitamin A deficiency | 1999 | 13883.51 | 14515.87 | 13264.48 |
| Incidence | Global | Male | Vitamin A deficiency | 1999 | 16292.51 | 17471.9 | 15114.33 |
| Incidence | Global | Female | Vitamin A deficiency | 2000 | 11238.77 | 11763.95 | 10765.05 |
| Incidence | Global | Both | Vitamin A deficiency | 2000 | 13608.26 | 14239.93 | 12985.06 |
| Incidence | Global | Male | Vitamin A deficiency | 2000 | 15900.83 | 17079.78 | 14710.86 |
| Incidence | Global | Female | Vitamin A deficiency | 2001 | 11016.12 | 11538.58 | 10557.74 |
| Incidence | Global | Both | Vitamin A deficiency | 2001 | 13191.04 | 13791.59 | 12574.64 |
| Incidence | Global | Male | Vitamin A deficiency | 2001 | 15296.26 | 16436.09 | 14149.86 |
| Incidence | Global | Female | Vitamin A deficiency | 2002 | 10840.95 | 11360.03 | 10382.87 |
| Incidence | Global | Both | Vitamin A deficiency | 2002 | 12879.59 | 13473.47 | 12273.45 |
| Incidence | Global | Male | Vitamin A deficiency | 2002 | 14853.76 | 16009.75 | 13758.5 |
| Incidence | Global | Female | Vitamin A deficiency | 2003 | 10640.81 | 11172.69 | 10183.5 |
| Incidence | Global | Both | Vitamin A deficiency | 2003 | 12578.86 | 13169.65 | 11982.78 |
| Incidence | Global | Male | Vitamin A deficiency | 2003 | 14456.48 | 15583.34 | 13423.06 |
| Incidence | Global | Female | Vitamin A deficiency | 2004 | 10384.8 | 10911.02 | 9923.255 |
| Incidence | Global | Both | Vitamin A deficiency | 2004 | 12230.95 | 12820.15 | 11650.1 |
| Incidence | Global | Male | Vitamin A deficiency | 2004 | 14020.4 | 15101.78 | 13030.54 |
| Incidence | Global | Female | Vitamin A deficiency | 2005 | 10049.33 | 10567.44 | 9602.397 |
| Incidence | Global | Both | Vitamin A deficiency | 2005 | 11812.49 | 12375.62 | 11242.35 |
| Incidence | Global | Male | Vitamin A deficiency | 2005 | 13522.32 | 14563.69 | 12548.03 |
| Incidence | Global | Female | Vitamin A deficiency | 2006 | 9733.912 | 10237.08 | 9311.619 |
| Incidence | Global | Both | Vitamin A deficiency | 2006 | 11417.63 | 11961.97 | 10883.32 |
| Incidence | Global | Male | Vitamin A deficiency | 2006 | 13050.97 | 14058.07 | 12132.09 |
| Incidence | Global | Female | Vitamin A deficiency | 2007 | 9439.911 | 9929.166 | 9030.173 |
| Incidence | Global | Both | Vitamin A deficiency | 2007 | 11050.13 | 11594.25 | 10537.28 |
| Incidence | Global | Male | Vitamin A deficiency | 2007 | 12612.46 | 13571.57 | 11733.95 |
| Incidence | Global | Female | Vitamin A deficiency | 2008 | 9151.351 | 9630.622 | 8744.418 |
| Incidence | Global | Both | Vitamin A deficiency | 2008 | 10698.49 | 11207.65 | 10204.46 |
| Incidence | Global | Male | Vitamin A deficiency | 2008 | 12199.89 | 13123.7 | 11349.3 |
| Incidence | Global | Female | Vitamin A deficiency | 2009 | 8825.799 | 9275.975 | 8426.477 |
| Incidence | Global | Both | Vitamin A deficiency | 2009 | 10300.99 | 10782.19 | 9829.239 |
| Incidence | Global | Male | Vitamin A deficiency | 2009 | 11733.08 | 12595.9 | 10934.7 |
| Incidence | Global | Female | Vitamin A deficiency | 2010 | 8503.13 | 8942.989 | 8114.223 |
| Incidence | Global | Both | Vitamin A deficiency | 2010 | 9920.194 | 10386 | 9468.799 |
| Incidence | Global | Male | Vitamin A deficiency | 2010 | 11296.51 | 12111.79 | 10535.68 |
| Incidence | Global | Female | Vitamin A deficiency | 2011 | 8206.337 | 8640.965 | 7825.621 |
| Incidence | Global | Both | Vitamin A deficiency | 2011 | 9565.189 | 10015.69 | 9140.367 |
| Incidence | Global | Male | Vitamin A deficiency | 2011 | 10885.46 | 11669.66 | 10140.5 |
| Incidence | Global | Female | Vitamin A deficiency | 2012 | 7875.076 | 8288.102 | 7500.219 |
| Incidence | Global | Both | Vitamin A deficiency | 2012 | 9171.265 | 9613.016 | 8772.946 |
| Incidence | Global | Male | Vitamin A deficiency | 2012 | 10431.02 | 11189.22 | 9709.717 |
| Incidence | Global | Female | Vitamin A deficiency | 2013 | 7541.251 | 7933.017 | 7181.344 |
| Incidence | Global | Both | Vitamin A deficiency | 2013 | 8777.158 | 9200.957 | 8388.662 |
| Incidence | Global | Male | Vitamin A deficiency | 2013 | 9978.647 | 10696.62 | 9308.066 |
| Incidence | Global | Female | Vitamin A deficiency | 2014 | 7212.18 | 7586.229 | 6862 |
| Incidence | Global | Both | Vitamin A deficiency | 2014 | 8388.131 | 8780.854 | 8027.771 |
| Incidence | Global | Male | Vitamin A deficiency | 2014 | 9531.629 | 10218.31 | 8904.067 |
| Incidence | Global | Female | Vitamin A deficiency | 2015 | 6889.308 | 7241.005 | 6559.311 |
| Incidence | Global | Both | Vitamin A deficiency | 2015 | 8009.326 | 8379.828 | 7669.854 |
| Incidence | Global | Male | Vitamin A deficiency | 2015 | 9098.639 | 9782.177 | 8495.632 |
| Incidence | Global | Female | Vitamin A deficiency | 2016 | 6640.697 | 6979.829 | 6318.76 |
| Incidence | Global | Both | Vitamin A deficiency | 2016 | 7713.412 | 8074.516 | 7379.538 |
| Incidence | Global | Male | Vitamin A deficiency | 2016 | 8756.81 | 9417.57 | 8177.239 |
| Incidence | Global | Female | Vitamin A deficiency | 2017 | 6406.097 | 6737.445 | 6098.774 |
| Incidence | Global | Both | Vitamin A deficiency | 2017 | 7438.826 | 7789.849 | 7113.375 |
| Incidence | Global | Male | Vitamin A deficiency | 2017 | 8443.387 | 9087.267 | 7895.852 |
| Incidence | Global | Female | Vitamin A deficiency | 2018 | 6178.644 | 6501.174 | 5887.416 |
| Incidence | Global | Both | Vitamin A deficiency | 2018 | 7168.303 | 7511.796 | 6852.082 |
| Incidence | Global | Male | Vitamin A deficiency | 2018 | 8131.034 | 8734.196 | 7597.767 |
| Incidence | Global | Female | Vitamin A deficiency | 2019 | 5999.11 | 6307.265 | 5719.022 |
| Incidence | Global | Both | Vitamin A deficiency | 2019 | 6955.65 | 7294.227 | 6645.869 |
| Incidence | Global | Male | Vitamin A deficiency | 2019 | 7886.237 | 8489.752 | 7367.67 |
| Prevalence | Global | Male | Dietary iron deficiency | 1990 | 15718.21 | 16036.38 | 15401.38 |
| Prevalence | Global | Both | Dietary iron deficiency | 1990 | 16252.62 | 16509.38 | 15948.78 |
| Prevalence | Global | Female | Dietary iron deficiency | 1990 | 16895.08 | 17250.54 | 16524.08 |
| Prevalence | Global | Male | Dietary iron deficiency | 1991 | 15653.74 | 15959.84 | 15342.48 |
| Prevalence | Global | Both | Dietary iron deficiency | 1991 | 16217 | 16465.21 | 15923.12 |
| Prevalence | Global | Female | Dietary iron deficiency | 1991 | 16886.8 | 17231.88 | 16524.96 |
| Prevalence | Global | Male | Dietary iron deficiency | 1992 | 15577.55 | 15872.69 | 15274.44 |
| Prevalence | Global | Both | Dietary iron deficiency | 1992 | 16166.81 | 16411.81 | 15878.5 |
| Prevalence | Global | Female | Dietary iron deficiency | 1992 | 16860.83 | 17195.53 | 16504.65 |
| Prevalence | Global | Male | Dietary iron deficiency | 1993 | 15494.83 | 15783.85 | 15195.76 |
| Prevalence | Global | Both | Dietary iron deficiency | 1993 | 16107.53 | 16347.9 | 15826.38 |
| Prevalence | Global | Female | Dietary iron deficiency | 1993 | 16823.02 | 17146.75 | 16476.83 |
| Prevalence | Global | Male | Dietary iron deficiency | 1994 | 15408.47 | 15698.27 | 15109.3 |
| Prevalence | Global | Both | Dietary iron deficiency | 1994 | 16041.7 | 16283.48 | 15764.59 |
| Prevalence | Global | Female | Dietary iron deficiency | 1994 | 16775.52 | 17097.5 | 16426.63 |
| Prevalence | Global | Male | Dietary iron deficiency | 1995 | 15320.78 | 15610.94 | 15027.74 |
| Prevalence | Global | Both | Dietary iron deficiency | 1995 | 15972.19 | 16210.48 | 15691.87 |
| Prevalence | Global | Female | Dietary iron deficiency | 1995 | 16721.85 | 17034.91 | 16369.38 |
| Prevalence | Global | Male | Dietary iron deficiency | 1996 | 15220.96 | 15494.99 | 14930.98 |
| Prevalence | Global | Both | Dietary iron deficiency | 1996 | 15890.28 | 16121.9 | 15617.57 |
| Prevalence | Global | Female | Dietary iron deficiency | 1996 | 16655.23 | 16953.82 | 16310.5 |
| Prevalence | Global | Male | Dietary iron deficiency | 1997 | 15098.91 | 15367.27 | 14808.8 |
| Prevalence | Global | Both | Dietary iron deficiency | 1997 | 15786.99 | 16019.78 | 15510.38 |
| Prevalence | Global | Female | Dietary iron deficiency | 1997 | 16567.68 | 16866.98 | 16229.65 |
| Prevalence | Global | Male | Dietary iron deficiency | 1998 | 14966.94 | 15231.32 | 14681.19 |
| Prevalence | Global | Both | Dietary iron deficiency | 1998 | 15675.01 | 15902.38 | 15402.3 |
| Prevalence | Global | Female | Dietary iron deficiency | 1998 | 16472.25 | 16768.15 | 16145.03 |
| Prevalence | Global | Male | Dietary iron deficiency | 1999 | 14837.23 | 15094.29 | 14555.57 |
| Prevalence | Global | Both | Dietary iron deficiency | 1999 | 15565.48 | 15788.66 | 15297.25 |
| Prevalence | Global | Female | Dietary iron deficiency | 1999 | 16379.52 | 16675.86 | 16056.51 |
| Prevalence | Global | Male | Dietary iron deficiency | 2000 | 14717.67 | 14972.54 | 14445.64 |
| Prevalence | Global | Both | Dietary iron deficiency | 2000 | 15465.67 | 15688.55 | 15201.76 |
| Prevalence | Global | Female | Dietary iron deficiency | 2000 | 16295.98 | 16590.83 | 15973.26 |
| Prevalence | Global | Male | Dietary iron deficiency | 2001 | 14585.35 | 14839.27 | 14314.25 |
| Prevalence | Global | Both | Dietary iron deficiency | 2001 | 15360.72 | 15583.47 | 15108.27 |
| Prevalence | Global | Female | Dietary iron deficiency | 2001 | 16215.2 | 16498.86 | 15897.27 |
| Prevalence | Global | Male | Dietary iron deficiency | 2002 | 14421.95 | 14667.55 | 14152.3 |
| Prevalence | Global | Both | Dietary iron deficiency | 2002 | 15235.76 | 15455.8 | 14985.25 |
| Prevalence | Global | Female | Dietary iron deficiency | 2002 | 16125.71 | 16398.41 | 15811.37 |
| Prevalence | Global | Male | Dietary iron deficiency | 2003 | 14248.67 | 14506.01 | 13974.64 |
| Prevalence | Global | Both | Dietary iron deficiency | 2003 | 15104.48 | 15322.83 | 14852.9 |
| Prevalence | Global | Female | Dietary iron deficiency | 2003 | 16033.76 | 16312.26 | 15716.94 |
| Prevalence | Global | Male | Dietary iron deficiency | 2004 | 14085.79 | 14343.4 | 13815.17 |
| Prevalence | Global | Both | Dietary iron deficiency | 2004 | 14980.08 | 15202 | 14724.17 |
| Prevalence | Global | Female | Dietary iron deficiency | 2004 | 15945 | 16224.78 | 15619.86 |
| Prevalence | Global | Male | Dietary iron deficiency | 2005 | 13952.71 | 14209.07 | 13672.95 |
| Prevalence | Global | Both | Dietary iron deficiency | 2005 | 14874.64 | 15095.74 | 14619.09 |
| Prevalence | Global | Female | Dietary iron deficiency | 2005 | 15864.62 | 16141.82 | 15536.73 |
| Prevalence | Global | Male | Dietary iron deficiency | 2006 | 13831.84 | 14089.09 | 13548.98 |
| Prevalence | Global | Both | Dietary iron deficiency | 2006 | 14773.28 | 14993.09 | 14522.39 |
| Prevalence | Global | Female | Dietary iron deficiency | 2006 | 15780.43 | 16057.6 | 15458.93 |
| Prevalence | Global | Male | Dietary iron deficiency | 2007 | 13698.97 | 13949.53 | 13420.7 |
| Prevalence | Global | Both | Dietary iron deficiency | 2007 | 14660.48 | 14878.42 | 14412.57 |
| Prevalence | Global | Female | Dietary iron deficiency | 2007 | 15685.8 | 15958.79 | 15373.24 |
| Prevalence | Global | Male | Dietary iron deficiency | 2008 | 13570.9 | 13815.08 | 13293.85 |
| Prevalence | Global | Both | Dietary iron deficiency | 2008 | 14552.43 | 14770.3 | 14302.33 |
| Prevalence | Global | Female | Dietary iron deficiency | 2008 | 15596.17 | 15870.98 | 15293.08 |
| Prevalence | Global | Male | Dietary iron deficiency | 2009 | 13463.53 | 13716.42 | 13167.1 |
| Prevalence | Global | Both | Dietary iron deficiency | 2009 | 14463.7 | 14680.27 | 14215.66 |
| Prevalence | Global | Female | Dietary iron deficiency | 2009 | 15524.45 | 15800.85 | 15220.77 |
| Prevalence | Global | Male | Dietary iron deficiency | 2010 | 13390.6 | 13647.26 | 13093.24 |
| Prevalence | Global | Both | Dietary iron deficiency | 2010 | 14405.5 | 14620.99 | 14159.74 |
| Prevalence | Global | Female | Dietary iron deficiency | 2010 | 15479.46 | 15753.33 | 15176.24 |
| Prevalence | Global | Male | Dietary iron deficiency | 2011 | 13343.56 | 13593.32 | 13052.09 |
| Prevalence | Global | Both | Dietary iron deficiency | 2011 | 14372.51 | 14585.95 | 14123.2 |
| Prevalence | Global | Female | Dietary iron deficiency | 2011 | 15459.4 | 15733.89 | 15165.67 |
| Prevalence | Global | Male | Dietary iron deficiency | 2012 | 13302.3 | 13553.92 | 13010.65 |
| Prevalence | Global | Both | Dietary iron deficiency | 2012 | 14348.27 | 14557.52 | 14100.51 |
| Prevalence | Global | Female | Dietary iron deficiency | 2012 | 15451.3 | 15720.74 | 15141.22 |
| Prevalence | Global | Male | Dietary iron deficiency | 2013 | 13266.18 | 13525.75 | 12979.2 |
| Prevalence | Global | Both | Dietary iron deficiency | 2013 | 14331.02 | 14543.99 | 14081.39 |
| Prevalence | Global | Female | Dietary iron deficiency | 2013 | 15452 | 15720.19 | 15136.72 |
| Prevalence | Global | Male | Dietary iron deficiency | 2014 | 13235.98 | 13503.37 | 12949.27 |
| Prevalence | Global | Both | Dietary iron deficiency | 2014 | 14319.05 | 14534.23 | 14072.89 |
| Prevalence | Global | Female | Dietary iron deficiency | 2014 | 15457.25 | 15731.27 | 15146.39 |
| Prevalence | Global | Male | Dietary iron deficiency | 2015 | 13206.78 | 13481.17 | 12925.64 |
| Prevalence | Global | Both | Dietary iron deficiency | 2015 | 14304.72 | 14525.14 | 14056.94 |
| Prevalence | Global | Female | Dietary iron deficiency | 2015 | 15456.83 | 15735.93 | 15151.22 |
| Prevalence | Global | Male | Dietary iron deficiency | 2016 | 13166.31 | 13445.36 | 12886.25 |
| Prevalence | Global | Both | Dietary iron deficiency | 2016 | 14283.97 | 14505 | 14029.68 |
| Prevalence | Global | Female | Dietary iron deficiency | 2016 | 15455.13 | 15746.63 | 15148.11 |
| Prevalence | Global | Male | Dietary iron deficiency | 2017 | 13114.24 | 13394.39 | 12825.84 |
| Prevalence | Global | Both | Dietary iron deficiency | 2017 | 14250.81 | 14475.5 | 13990.36 |
| Prevalence | Global | Female | Dietary iron deficiency | 2017 | 15440.32 | 15732.29 | 15120.99 |
| Prevalence | Global | Male | Dietary iron deficiency | 2018 | 13042.16 | 13331.07 | 12757 |
| Prevalence | Global | Both | Dietary iron deficiency | 2018 | 14191.2 | 14416.78 | 13931.9 |
| Prevalence | Global | Female | Dietary iron deficiency | 2018 | 15392.35 | 15690.34 | 15077.74 |
| Prevalence | Global | Male | Dietary iron deficiency | 2019 | 12944.59 | 13239.63 | 12651.39 |
| Prevalence | Global | Both | Dietary iron deficiency | 2019 | 14106.39 | 14342.09 | 13850.72 |
| Prevalence | Global | Female | Dietary iron deficiency | 2019 | 15319.38 | 15624.46 | 14991.34 |
| Prevalence | Global | Male | Iodine deficiency | 1990 | 2362.572 | 2869.569 | 1950.169 |
| Prevalence | Global | Both | Iodine deficiency | 1990 | 2833.699 | 3460.952 | 2365.135 |
| Prevalence | Global | Female | Iodine deficiency | 1990 | 3310.995 | 4037.816 | 2773.609 |
| Prevalence | Global | Male | Iodine deficiency | 1991 | 2277.407 | 2779.895 | 1884.269 |
| Prevalence | Global | Both | Iodine deficiency | 1991 | 2781.641 | 3394.871 | 2323.89 |
| Prevalence | Global | Female | Iodine deficiency | 1991 | 3293.05 | 4019.406 | 2755.506 |
| Prevalence | Global | Male | Iodine deficiency | 1992 | 2194.089 | 2689.795 | 1816.776 |
| Prevalence | Global | Both | Iodine deficiency | 1992 | 2727.2 | 3340.194 | 2278.592 |
| Prevalence | Global | Female | Iodine deficiency | 1992 | 3268.364 | 3984.219 | 2715.306 |
| Prevalence | Global | Male | Iodine deficiency | 1993 | 2115.358 | 2606.797 | 1753.54 |
| Prevalence | Global | Both | Iodine deficiency | 1993 | 2672.801 | 3277.11 | 2216.186 |
| Prevalence | Global | Female | Iodine deficiency | 1993 | 3238.953 | 3953.307 | 2678.337 |
| Prevalence | Global | Male | Iodine deficiency | 1994 | 2043.581 | 2523.861 | 1687.974 |
| Prevalence | Global | Both | Iodine deficiency | 1994 | 2620.738 | 3207.996 | 2159.878 |
| Prevalence | Global | Female | Iodine deficiency | 1994 | 3206.921 | 3931.995 | 2647.614 |
| Prevalence | Global | Male | Iodine deficiency | 1995 | 1981.021 | 2456.361 | 1620.583 |
| Prevalence | Global | Both | Iodine deficiency | 1995 | 2572.716 | 3152.224 | 2107.36 |
| Prevalence | Global | Female | Iodine deficiency | 1995 | 3173.385 | 3877.35 | 2618.175 |
| Prevalence | Global | Male | Iodine deficiency | 1996 | 1916.162 | 2374.452 | 1565.428 |
| Prevalence | Global | Both | Iodine deficiency | 1996 | 2514.673 | 3076.705 | 2059.992 |
| Prevalence | Global | Female | Iodine deficiency | 1996 | 3121.789 | 3819.81 | 2569.192 |
| Prevalence | Global | Male | Iodine deficiency | 1997 | 1843.467 | 2278.213 | 1505.776 |
| Prevalence | Global | Both | Iodine deficiency | 1997 | 2441.039 | 2990.457 | 1999.716 |
| Prevalence | Global | Female | Iodine deficiency | 1997 | 3046.615 | 3727.319 | 2502.403 |
| Prevalence | Global | Male | Iodine deficiency | 1998 | 1775.363 | 2195.99 | 1449.844 |
| Prevalence | Global | Both | Iodine deficiency | 1998 | 2367.759 | 2897.715 | 1939.912 |
| Prevalence | Global | Female | Iodine deficiency | 1998 | 2967.442 | 3622.264 | 2430.012 |
| Prevalence | Global | Male | Iodine deficiency | 1999 | 1724.651 | 2126.309 | 1408.073 |
| Prevalence | Global | Both | Iodine deficiency | 1999 | 2311.346 | 2827.948 | 1894.79 |
| Prevalence | Global | Female | Iodine deficiency | 1999 | 2904.612 | 3540.344 | 2383.524 |
| Prevalence | Global | Male | Iodine deficiency | 2000 | 1703.999 | 2100.894 | 1389.729 |
| Prevalence | Global | Both | Iodine deficiency | 2000 | 2288.467 | 2801.515 | 1872.7 |
| Prevalence | Global | Female | Iodine deficiency | 2000 | 2878.898 | 3510.545 | 2365.12 |
| Prevalence | Global | Male | Iodine deficiency | 2001 | 1714.697 | 2113.189 | 1396.298 |
| Prevalence | Global | Both | Iodine deficiency | 2001 | 2301.931 | 2817.822 | 1883.972 |
| Prevalence | Global | Female | Iodine deficiency | 2001 | 2894.691 | 3531.219 | 2378.388 |
| Prevalence | Global | Male | Iodine deficiency | 2002 | 1741.756 | 2149.434 | 1416.077 |
| Prevalence | Global | Both | Iodine deficiency | 2002 | 2334.168 | 2859.687 | 1907.992 |
| Prevalence | Global | Female | Iodine deficiency | 2002 | 2931.818 | 3580.036 | 2408.365 |
| Prevalence | Global | Male | Iodine deficiency | 2003 | 1774.498 | 2194.022 | 1440.36 |
| Prevalence | Global | Both | Iodine deficiency | 2003 | 2372.68 | 2914.551 | 1937.664 |
| Prevalence | Global | Female | Iodine deficiency | 2003 | 2975.864 | 3637.925 | 2442.281 |
| Prevalence | Global | Male | Iodine deficiency | 2004 | 1802.226 | 2231.164 | 1460.423 |
| Prevalence | Global | Both | Iodine deficiency | 2004 | 2404.737 | 2955.192 | 1962.272 |
| Prevalence | Global | Female | Iodine deficiency | 2004 | 3012.034 | 3684.67 | 2469.637 |
| Prevalence | Global | Male | Iodine deficiency | 2005 | 1813.967 | 2247.4 | 1464.362 |
| Prevalence | Global | Both | Iodine deficiency | 2005 | 2417.211 | 2973.054 | 1971.655 |
| Prevalence | Global | Female | Iodine deficiency | 2005 | 3025.013 | 3700.889 | 2475.626 |
| Prevalence | Global | Male | Iodine deficiency | 2006 | 1805.124 | 2237.993 | 1460.784 |
| Prevalence | Global | Both | Iodine deficiency | 2006 | 2404.082 | 2961.827 | 1961.936 |
| Prevalence | Global | Female | Iodine deficiency | 2006 | 3007.41 | 3687.006 | 2459.788 |
| Prevalence | Global | Male | Iodine deficiency | 2007 | 1783.217 | 2212.388 | 1445.692 |
| Prevalence | Global | Both | Iodine deficiency | 2007 | 2374.743 | 2932.216 | 1938.26 |
| Prevalence | Global | Female | Iodine deficiency | 2007 | 2970.504 | 3644.077 | 2428.958 |
| Prevalence | Global | Male | Iodine deficiency | 2008 | 1756.04 | 2179.147 | 1426.269 |
| Prevalence | Global | Both | Iodine deficiency | 2008 | 2339.953 | 2891.543 | 1910.143 |
| Prevalence | Global | Female | Iodine deficiency | 2008 | 2927.931 | 3589.752 | 2393.962 |
| Prevalence | Global | Male | Iodine deficiency | 2009 | 1730.795 | 2150.505 | 1404.79 |
| Prevalence | Global | Both | Iodine deficiency | 2009 | 2309.852 | 2848.527 | 1886.11 |
| Prevalence | Global | Female | Iodine deficiency | 2009 | 2892.724 | 3547.955 | 2366.722 |
| Prevalence | Global | Male | Iodine deficiency | 2010 | 1714.066 | 2131.632 | 1391.755 |
| Prevalence | Global | Both | Iodine deficiency | 2010 | 2293.973 | 2831.104 | 1873.908 |
| Prevalence | Global | Female | Iodine deficiency | 2010 | 2877.473 | 3538.478 | 2354.37 |
| Prevalence | Global | Male | Iodine deficiency | 2011 | 1698.614 | 2114.521 | 1377.555 |
| Prevalence | Global | Both | Iodine deficiency | 2011 | 2287.999 | 2823.872 | 1866.965 |
| Prevalence | Global | Female | Iodine deficiency | 2011 | 2880.877 | 3535.139 | 2354.153 |
| Prevalence | Global | Male | Iodine deficiency | 2012 | 1675.563 | 2086.279 | 1356.68 |
| Prevalence | Global | Both | Iodine deficiency | 2012 | 2280.966 | 2825.256 | 1858.858 |
| Prevalence | Global | Female | Iodine deficiency | 2012 | 2889.802 | 3542.252 | 2363.095 |
| Prevalence | Global | Male | Iodine deficiency | 2013 | 1649.398 | 2062.159 | 1332.846 |
| Prevalence | Global | Both | Iodine deficiency | 2013 | 2272.827 | 2802.209 | 1852.799 |
| Prevalence | Global | Female | Iodine deficiency | 2013 | 2899.611 | 3557.794 | 2370.266 |
| Prevalence | Global | Male | Iodine deficiency | 2014 | 1624.81 | 2036.481 | 1311.765 |
| Prevalence | Global | Both | Iodine deficiency | 2014 | 2263.695 | 2788.36 | 1842.806 |
| Prevalence | Global | Female | Iodine deficiency | 2014 | 2905.882 | 3565.485 | 2375.819 |
| Prevalence | Global | Male | Iodine deficiency | 2015 | 1606.852 | 2008.96 | 1299.191 |
| Prevalence | Global | Both | Iodine deficiency | 2015 | 2253.906 | 2780.365 | 1832.617 |
| Prevalence | Global | Female | Iodine deficiency | 2015 | 2904.33 | 3562.512 | 2375.219 |
| Prevalence | Global | Male | Iodine deficiency | 2016 | 1592.734 | 1989.035 | 1285.645 |
| Prevalence | Global | Both | Iodine deficiency | 2016 | 2236.91 | 2756.729 | 1819.276 |
| Prevalence | Global | Female | Iodine deficiency | 2016 | 2884.622 | 3535.236 | 2352.977 |
| Prevalence | Global | Male | Iodine deficiency | 2017 | 1580.962 | 1975.674 | 1274.333 |
| Prevalence | Global | Both | Iodine deficiency | 2017 | 2222.497 | 2735.729 | 1802.384 |
| Prevalence | Global | Female | Iodine deficiency | 2017 | 2867.744 | 3515.429 | 2331.219 |
| Prevalence | Global | Male | Iodine deficiency | 2018 | 1571.976 | 1963.561 | 1266.194 |
| Prevalence | Global | Both | Iodine deficiency | 2018 | 2217.71 | 2736.063 | 1800.676 |
| Prevalence | Global | Female | Iodine deficiency | 2018 | 2867.334 | 3529.439 | 2342.377 |
| Prevalence | Global | Male | Iodine deficiency | 2019 | 1563.469 | 1948.374 | 1261.53 |
| Prevalence | Global | Both | Iodine deficiency | 2019 | 2215.541 | 2743.312 | 1803.813 |
| Prevalence | Global | Female | Iodine deficiency | 2019 | 2871.715 | 3546.289 | 2346.848 |
| Prevalence | Global | Female | Vitamin A deficiency | 1990 | 13456.51 | 14141.38 | 12848.51 |
| Prevalence | Global | Both | Vitamin A deficiency | 1990 | 17323.23 | 18138.92 | 16526.51 |
| Prevalence | Global | Male | Vitamin A deficiency | 1990 | 21073.77 | 22557.32 | 19711.46 |
| Prevalence | Global | Female | Vitamin A deficiency | 1991 | 13325.45 | 13993.55 | 12721.85 |
| Prevalence | Global | Both | Vitamin A deficiency | 1991 | 17063.93 | 17854.17 | 16290.5 |
| Prevalence | Global | Male | Vitamin A deficiency | 1991 | 20686.7 | 22156.18 | 19316.36 |
| Prevalence | Global | Female | Vitamin A deficiency | 1992 | 13217.18 | 13887.14 | 12623.15 |
| Prevalence | Global | Both | Vitamin A deficiency | 1992 | 16863.27 | 17663.36 | 16107.66 |
| Prevalence | Global | Male | Vitamin A deficiency | 1992 | 20393.41 | 21840.45 | 19033.98 |
| Prevalence | Global | Female | Vitamin A deficiency | 1993 | 12798.73 | 13442.59 | 12218.47 |
| Prevalence | Global | Both | Vitamin A deficiency | 1993 | 16212.75 | 16986.94 | 15514.39 |
| Prevalence | Global | Male | Vitamin A deficiency | 1993 | 19515.71 | 20886.21 | 18201.29 |
| Prevalence | Global | Female | Vitamin A deficiency | 1994 | 12427.08 | 13026.55 | 11871.95 |
| Prevalence | Global | Both | Vitamin A deficiency | 1994 | 15643.83 | 16369.95 | 14953.08 |
| Prevalence | Global | Male | Vitamin A deficiency | 1994 | 18754.45 | 20066.89 | 17484.22 |
| Prevalence | Global | Female | Vitamin A deficiency | 1995 | 12180.7 | 12784.32 | 11642.84 |
| Prevalence | Global | Both | Vitamin A deficiency | 1995 | 15232.28 | 15927.14 | 14563.21 |
| Prevalence | Global | Male | Vitamin A deficiency | 1995 | 18182.36 | 19435.98 | 16961.75 |
| Prevalence | Global | Female | Vitamin A deficiency | 1996 | 11883.9 | 12484.02 | 11372.02 |
| Prevalence | Global | Both | Vitamin A deficiency | 1996 | 14806.46 | 15489.4 | 14166.08 |
| Prevalence | Global | Male | Vitamin A deficiency | 1996 | 17631.43 | 18871.11 | 16445.74 |
| Prevalence | Global | Female | Vitamin A deficiency | 1997 | 11567.73 | 12130.87 | 11082.82 |
| Prevalence | Global | Both | Vitamin A deficiency | 1997 | 14316.25 | 14981.96 | 13689.06 |
| Prevalence | Global | Male | Vitamin A deficiency | 1997 | 16973.03 | 18162.73 | 15811.67 |
| Prevalence | Global | Female | Vitamin A deficiency | 1998 | 11374.25 | 11922.5 | 10908.16 |
| Prevalence | Global | Both | Vitamin A deficiency | 1998 | 13979.77 | 14614.04 | 13355.4 |
| Prevalence | Global | Male | Vitamin A deficiency | 1998 | 16498.76 | 17667.64 | 15323.17 |
| Prevalence | Global | Female | Vitamin A deficiency | 1999 | 11392.64 | 11927.18 | 10918.86 |
| Prevalence | Global | Both | Vitamin A deficiency | 1999 | 13883.51 | 14515.87 | 13264.48 |
| Prevalence | Global | Male | Vitamin A deficiency | 1999 | 16292.51 | 17471.9 | 15114.33 |
| Prevalence | Global | Female | Vitamin A deficiency | 2000 | 11238.77 | 11763.95 | 10765.05 |
| Prevalence | Global | Both | Vitamin A deficiency | 2000 | 13608.26 | 14239.93 | 12985.06 |
| Prevalence | Global | Male | Vitamin A deficiency | 2000 | 15900.83 | 17079.78 | 14710.86 |
| Prevalence | Global | Female | Vitamin A deficiency | 2001 | 11016.12 | 11538.58 | 10557.74 |
| Prevalence | Global | Both | Vitamin A deficiency | 2001 | 13191.04 | 13791.59 | 12574.64 |
| Prevalence | Global | Male | Vitamin A deficiency | 2001 | 15296.26 | 16436.09 | 14149.86 |
| Prevalence | Global | Female | Vitamin A deficiency | 2002 | 10840.95 | 11360.03 | 10382.87 |
| Prevalence | Global | Both | Vitamin A deficiency | 2002 | 12879.59 | 13473.47 | 12273.45 |
| Prevalence | Global | Male | Vitamin A deficiency | 2002 | 14853.76 | 16009.75 | 13758.5 |
| Prevalence | Global | Female | Vitamin A deficiency | 2003 | 10640.81 | 11172.69 | 10183.5 |
| Prevalence | Global | Both | Vitamin A deficiency | 2003 | 12578.86 | 13169.65 | 11982.78 |
| Prevalence | Global | Male | Vitamin A deficiency | 2003 | 14456.48 | 15583.34 | 13423.06 |
| Prevalence | Global | Female | Vitamin A deficiency | 2004 | 10384.8 | 10911.02 | 9923.255 |
| Prevalence | Global | Both | Vitamin A deficiency | 2004 | 12230.95 | 12820.15 | 11650.1 |
| Prevalence | Global | Male | Vitamin A deficiency | 2004 | 14020.4 | 15101.78 | 13030.54 |
| Prevalence | Global | Female | Vitamin A deficiency | 2005 | 10049.33 | 10567.44 | 9602.397 |
| Prevalence | Global | Both | Vitamin A deficiency | 2005 | 11812.49 | 12375.62 | 11242.35 |
| Prevalence | Global | Male | Vitamin A deficiency | 2005 | 13522.32 | 14563.69 | 12548.03 |
| Prevalence | Global | Female | Vitamin A deficiency | 2006 | 9733.912 | 10237.08 | 9311.619 |
| Prevalence | Global | Both | Vitamin A deficiency | 2006 | 11417.63 | 11961.97 | 10883.32 |
| Prevalence | Global | Male | Vitamin A deficiency | 2006 | 13050.97 | 14058.07 | 12132.09 |
| Prevalence | Global | Female | Vitamin A deficiency | 2007 | 9439.911 | 9929.166 | 9030.173 |
| Prevalence | Global | Both | Vitamin A deficiency | 2007 | 11050.13 | 11594.25 | 10537.28 |
| Prevalence | Global | Male | Vitamin A deficiency | 2007 | 12612.46 | 13571.57 | 11733.95 |
| Prevalence | Global | Female | Vitamin A deficiency | 2008 | 9151.351 | 9630.622 | 8744.418 |
| Prevalence | Global | Both | Vitamin A deficiency | 2008 | 10698.49 | 11207.65 | 10204.46 |
| Prevalence | Global | Male | Vitamin A deficiency | 2008 | 12199.89 | 13123.7 | 11349.3 |
| Prevalence | Global | Female | Vitamin A deficiency | 2009 | 8825.799 | 9275.975 | 8426.477 |
| Prevalence | Global | Both | Vitamin A deficiency | 2009 | 10300.99 | 10782.19 | 9829.239 |
| Prevalence | Global | Male | Vitamin A deficiency | 2009 | 11733.08 | 12595.9 | 10934.7 |
| Prevalence | Global | Female | Vitamin A deficiency | 2010 | 8503.13 | 8942.989 | 8114.223 |
| Prevalence | Global | Both | Vitamin A deficiency | 2010 | 9920.194 | 10386 | 9468.799 |
| Prevalence | Global | Male | Vitamin A deficiency | 2010 | 11296.51 | 12111.79 | 10535.68 |
| Prevalence | Global | Female | Vitamin A deficiency | 2011 | 8206.337 | 8640.965 | 7825.621 |
| Prevalence | Global | Both | Vitamin A deficiency | 2011 | 9565.189 | 10015.69 | 9140.367 |
| Prevalence | Global | Male | Vitamin A deficiency | 2011 | 10885.46 | 11669.66 | 10140.5 |
| Prevalence | Global | Female | Vitamin A deficiency | 2012 | 7875.076 | 8288.102 | 7500.219 |
| Prevalence | Global | Both | Vitamin A deficiency | 2012 | 9171.265 | 9613.016 | 8772.946 |
| Prevalence | Global | Male | Vitamin A deficiency | 2012 | 10431.02 | 11189.22 | 9709.717 |
| Prevalence | Global | Female | Vitamin A deficiency | 2013 | 7541.251 | 7933.017 | 7181.344 |
| Prevalence | Global | Both | Vitamin A deficiency | 2013 | 8777.158 | 9200.957 | 8388.662 |
| Prevalence | Global | Male | Vitamin A deficiency | 2013 | 9978.647 | 10696.62 | 9308.066 |
| Prevalence | Global | Female | Vitamin A deficiency | 2014 | 7212.18 | 7586.229 | 6862 |
| Prevalence | Global | Both | Vitamin A deficiency | 2014 | 8388.131 | 8780.854 | 8027.771 |
| Prevalence | Global | Male | Vitamin A deficiency | 2014 | 9531.629 | 10218.31 | 8904.067 |
| Prevalence | Global | Female | Vitamin A deficiency | 2015 | 6889.308 | 7241.005 | 6559.311 |
| Prevalence | Global | Both | Vitamin A deficiency | 2015 | 8009.326 | 8379.828 | 7669.854 |
| Prevalence | Global | Male | Vitamin A deficiency | 2015 | 9098.639 | 9782.177 | 8495.632 |
| Prevalence | Global | Female | Vitamin A deficiency | 2016 | 6640.697 | 6979.829 | 6318.76 |
| Prevalence | Global | Both | Vitamin A deficiency | 2016 | 7713.412 | 8074.516 | 7379.538 |
| Prevalence | Global | Male | Vitamin A deficiency | 2016 | 8756.81 | 9417.57 | 8177.239 |
| Prevalence | Global | Female | Vitamin A deficiency | 2017 | 6406.097 | 6737.445 | 6098.774 |
| Prevalence | Global | Both | Vitamin A deficiency | 2017 | 7438.826 | 7789.849 | 7113.375 |
| Prevalence | Global | Male | Vitamin A deficiency | 2017 | 8443.387 | 9087.267 | 7895.852 |
| Prevalence | Global | Female | Vitamin A deficiency | 2018 | 6178.644 | 6501.174 | 5887.416 |
| Prevalence | Global | Both | Vitamin A deficiency | 2018 | 7168.303 | 7511.796 | 6852.082 |
| Prevalence | Global | Male | Vitamin A deficiency | 2018 | 8131.034 | 8734.196 | 7597.767 |
| Prevalence | Global | Female | Vitamin A deficiency | 2019 | 5999.11 | 6307.265 | 5719.022 |
| Prevalence | Global | Both | Vitamin A deficiency | 2019 | 6955.65 | 7294.227 | 6645.869 |
| Prevalence | Global | Male | Vitamin A deficiency | 2019 | 7886.237 | 8489.752 | 7367.67 |

**Supplementary Table 2.** The estimated annual percentage changes (APC) of age-standardized prevalence rate worldwide from 1990 to 2019.

| Location | Iodine deficiency | |  | Vitamin A deficiency | |  | Dietary iron deficiency | | |
| --- | --- | --- | --- | --- | --- | --- | --- | --- | --- |
|  | APC | Lower CI | Upper CI | APC | Lower CI | Upper CI | APC | Lower CI | Upper CI |
| Global | -0.7* | -0.8 | -0.5 | -3.1* | -3.2 | -3 | -0.5* | -0.6 | -0.5 |
| China | 0.4 | 0 | 0.9 | -5.7* | -5.9 | -5.5 | -3.9* | -4.1 | -3.7 |
| Democratic People's Republic of Korea | -1.7* | -2 | -1.5 | -3.3* | -3.6 | -3.1 | -0.7* | -0.9 | -0.5 |
| Taiwan (Province of China) | -1.7* | -1.8 | -1.7 | -5.5* | -5.8 | -5.3 | -2.0* | -2.1 | -1.8 |
| Cambodia | -6.6* | -7.2 | -6 | -5.4* | -5.5 | -5.2 | -0.6* | -0.7 | -0.6 |
| Indonesia | -2.8* | -2.8 | -2.7 | -5.1* | -5.2 | -5 | -1.6* | -1.7 | -1.5 |
| Lao People's Democratic Republic | -2.6* | -2.6 | -2.5 | -4.0* | -4.3 | -3.7 | -1.0* | -1 | -0.9 |
| Malaysia | -6.6* | -7 | -6.3 | -6.2* | -6.8 | -5.7 | -1.0* | -1 | -1 |
| Maldives | -5.1* | -5.3 | -5 | -8.1* | -8.5 | -7.6 | -2.8* | -3 | -2.6 |
| Myanmar | -4.4* | -4.7 | -4.2 | -6.7* | -6.9 | -6.5 | -0.4* | -0.4 | -0.4 |
| Philippines | 1.3* | 0.2 | 2.4 | -1.8* | -2.2 | -1.4 | -2.0* | -2.2 | -1.9 |
| Sri Lanka | -3.4* | -3.5 | -3.3 | -5.7* | -5.9 | -5.5 | -2.0* | -2.1 | -1.9 |
| Thailand | -2.8* | -3.1 | -2.5 | -7.4* | -7.9 | -7 | -2.0* | -2.1 | -1.9 |
| Timor-Leste | -3.6* | -3.9 | -3.3 | -6.0* | -6.4 | -5.6 | -0.8* | -1 | -0.7 |
| Viet Nam | -4.7* | -4.7 | -4.6 | -5.3* | -5.6 | -5 | -2.5* | -2.6 | -2.4 |
| Fiji | -2.1* | -2.3 | -1.8 | -2.6* | -2.8 | -2.4 | 0.4* | 0.3 | 0.6 |
| Kiribati | -2.2* | -2.6 | -1.8 | -0.1 | -0.3 | 0 | -0.1* | -0.1 | -0.1 |
| Marshall Islands | -2.3* | -2.7 | -1.9 | -2.4* | -2.5 | -2.4 | -0.2* | -0.2 | -0.1 |
| Micronesia (Federated States of) | -2.3* | -2.6 | -2 | -1.3* | -1.3 | -1.2 | -0.3* | -0.4 | -0.2 |
| Papua New Guinea | -2.5* | -2.9 | -2.1 | -0.7* | -1.1 | -0.2 | -0.2* | -0.3 | -0.2 |
| Samoa | -1.1* | -1.2 | -1.1 | -0.7* | -0.9 | -0.5 | -0.3* | -0.4 | -0.2 |
| Solomon Islands | -0.8* | -1 | -0.7 | -1.2* | -1.4 | -1 | 0 | -0.1 | 0 |
| Tonga | -2.2* | -2.4 | -1.9 | -1.7* | -2 | -1.5 | -0.1* | -0.2 | -0.1 |
| Vanuatu | -1.0* | -1.1 | -0.9 | -1.2* | -1.3 | -1 | 0.5* | 0.4 | 0.6 |
| Armenia | -2.7* | -3.4 | -2 | -2.8* | -3.2 | -2.3 | -0.6* | -0.7 | -0.5 |
| Azerbaijan | -2.1* | -2.7 | -1.5 | -2.2* | -2.9 | -1.6 | -0.8* | -0.9 | -0.6 |
| Georgia | -0.7* | -0.8 | -0.6 | -0.2 | -0.8 | 0.5 | -0.4* | -0.4 | -0.3 |
| Kazakhstan | -3.1* | -3.6 | -2.7 | -2.5* | -2.6 | -2.4 | -1.1* | -1.3 | -1 |
| Kyrgyzstan | -0.2* | -0.3 | -0.2 | -0.6* | -0.9 | -0.3 | -0.7* | -0.8 | -0.6 |
| Mongolia | -4.3* | -4.6 | -3.9 | -4.8* | -5 | -4.6 | -1.6* | -1.8 | -1.4 |
| Tajikistan | -1.5* | -2.5 | -0.6 | -1.7* | -2.2 | -1.1 | -0.5* | -0.6 | -0.4 |
| Turkmenistan | -0.9* | -1 | -0.9 | -3.4* | -3.8 | -3.1 | -0.8* | -0.9 | -0.7 |
| Uzbekistan | -3.2* | -3.6 | -2.7 | -3.4* | -3.6 | -3.1 | -0.6* | -0.7 | -0.5 |
| Albania | -2.3* | -2.5 | -2.1 | -3.5* | -3.7 | -3.2 | -1.4* | -1.5 | -1.3 |
| Bosnia and Herzegovina | -3.3* | -3.9 | -2.8 | -3.8* | -4.1 | -3.5 | -1.3* | -1.4 | -1.1 |
| Bulgaria | -1.7* | -1.8 | -1.6 | -1.8* | -2 | -1.5 | -0.6* | -0.6 | -0.5 |
| Croatia | -1.3* | -1.4 | -1.1 | -1.9* | -2.1 | -1.7 | -1.0* | -1.1 | -0.9 |
| Czechia | -1.1* | -1.3 | -1 | -1.9* | -2 | -1.7 | -1.1* | -1.3 | -1 |
| Hungary | -2.3* | -2.5 | -2 | -1.9* | -2 | -1.8 | -0.9* | -1.1 | -0.8 |
| North Macedonia | -1.4* | -1.5 | -1.3 | -2.7* | -2.9 | -2.6 | -1.0* | -1.1 | -0.9 |
| Montenegro | -1.3* | -1.4 | -1.2 | -2.0* | -2.5 | -1.5 | -0.7* | -0.8 | -0.6 |
| Poland | -0.7* | -0.7 | -0.6 | -2.8* | -2.9 | -2.8 | -1.3* | -1.4 | -1.2 |
| Romania | -0.5* | -0.6 | -0.3 | -2.6* | -2.7 | -2.4 | -1.2* | -1.2 | -1.2 |
| Serbia | -1.5* | -1.7 | -1.4 | -2.4* | -2.6 | -2.2 | -1.3* | -1.3 | -1.2 |
| Slovakia | -1.4* | -1.6 | -1.3 | -2.5* | -2.6 | -2.4 | -1.0* | -1.1 | -1 |
| Slovenia | -1.3* | -1.4 | -1.1 | -1.9* | -1.9 | -1.8 | -1.4* | -1.5 | -1.4 |
| Belarus | -1.2* | -1.5 | -0.8 | -3.6* | -3.8 | -3.3 | -1.5* | -1.8 | -1.3 |
| Estonia | -1.0* | -1 | -0.9 | -3.5* | -3.6 | -3.4 | -2.0* | -2.2 | -1.9 |
| Latvia | -0.8* | -1 | -0.5 | -2.9* | -3.1 | -2.7 | -1.3* | -1.5 | -1.2 |
| Lithuania | -0.8* | -1 | -0.6 | -3.5* | -3.6 | -3.4 | -0.8* | -0.9 | -0.8 |
| Republic of Moldova | 0.4 | -0.2 | 1 | -2.4* | -2.8 | -2 | -1.2* | -1.3 | -1.1 |
| Russian Federation | -0.4 | -0.7 | 0 | -2.5* | -2.6 | -2.4 | -1.4* | -1.7 | -1.2 |
| Ukraine | -0.1 | -0.7 | 0.5 | -1.7* | -1.9 | -1.5 | -1.3* | -1.5 | -1.2 |
| Brunei Darussalam | -0.2* | -0.3 | -0.2 | -3.0* | -3 | -2.9 | -1.0* | -1.1 | -0.9 |
| Japan | -0.4* | -0.4 | -0.4 | -1.0* | -1.1 | -0.9 | -1.3* | -1.4 | -1.2 |
| Republic of Korea | -1.3* | -1.4 | -1.2 | -4.2* | -4.4 | -4 | -3.1* | -3.5 | -2.8 |
| Singapore | -0.7* | -0.7 | -0.6 | -3.6* | -3.7 | -3.5 | -2.3* | -2.4 | -2.2 |
| Australia | -0.3* | -0.3 | -0.2 | -1.2* | -1.6 | -0.8 | -1.4* | -1.6 | -1.3 |
| New Zealand | -0.1* | -0.1 | -0.1 | -0.7* | -1 | -0.5 | -0.9* | -0.9 | -0.8 |
| Andorra | 0.1* | 0.1 | 0.2 | -1.6* | -1.8 | -1.4 | -1.5* | -1.7 | -1.3 |
| Austria | -0.1* | -0.2 | -0.1 | -2.6* | -2.9 | -2.3 | -1.5* | -1.7 | -1.4 |
| Belgium | -0.2* | -0.2 | -0.2 | -2.5* | -2.8 | -2.2 | -1.8* | -1.9 | -1.7 |
| Cyprus | -0.2* | -0.2 | -0.1 | -4.6* | -5.1 | -4 | -3.1* | -3.3 | -2.8 |
| Denmark | -0.2* | -0.3 | -0.2 | -2.0* | -2.2 | -1.8 | -1.9* | -2 | -1.8 |
| Finland | -0.2* | -0.3 | -0.2 | -2.4* | -2.7 | -2.2 | -2.1* | -2.3 | -1.9 |
| France | -0.0* | 0 | 0 | -0.9* | -1.1 | -0.7 | -2.1* | -2.2 | -1.9 |
| Germany | -0.1* | -0.2 | -0.1 | -1.4* | -1.5 | -1.3 | -1.7* | -2 | -1.4 |
| Greece | -0.4* | -0.5 | -0.3 | -2.6* | -3.1 | -2.2 | -1.3* | -1.5 | -1.1 |
| Iceland | -0.2* | -0.2 | -0.1 | -2.9* | -3.1 | -2.7 | -1.3* | -1.5 | -1 |
| Ireland | -0.4* | -0.5 | -0.4 | -2.9* | -3.3 | -2.5 | -2.4* | -2.6 | -2.3 |
| Israel | -0.2* | -0.2 | -0.2 | -2.6* | -2.9 | -2.2 | -1.8* | -1.9 | -1.7 |
| Italy | -1.9* | -2.2 | -1.5 | -2.2* | -2.6 | -1.8 | -1.6* | -1.8 | -1.5 |
| Luxembourg | -0.1* | -0.1 | -0.1 | -2.0* | -2.2 | -1.7 | -2.2* | -2.4 | -1.9 |
| Malta | -0.5* | -0.5 | -0.5 | -3.7* | -4.1 | -3.4 | -1.7* | -1.8 | -1.7 |
| Netherlands | -0.2* | -0.2 | -0.1 | -1.8* | -2.1 | -1.6 | -1.8* | -1.9 | -1.7 |
| Norway | -0.1* | -0.2 | -0.1 | -2.7* | -3 | -2.5 | -1.7* | -1.9 | -1.5 |
| Portugal | 0.1* | 0 | 0.1 | -3.4* | -3.7 | -3 | -2.4* | -2.6 | -2.3 |
| Spain | -0.1* | -0.2 | -0.1 | -2.9* | -3.3 | -2.5 | -2.0* | -2.2 | -1.9 |
| Sweden | -0.2* | -0.2 | -0.2 | -2.5* | -2.9 | -2.2 | -1.0* | -1.2 | -0.9 |
| Switzerland | -0.1* | -0.1 | 0 | -1.6* | -1.8 | -1.5 | -1.3* | -1.5 | -1.2 |
| United Kingdom | -0.1* | -0.1 | 0 | -2.4* | -2.7 | -2.1 | -1.3* | -1.4 | -1.1 |
| Argentina | -0.6* | -0.6 | -0.6 | -1.2* | -1.4 | -1 | -1.2* | -1.4 | -1.1 |
| Chile | -1.9* | -2.1 | -1.7 | -2.3* | -2.5 | -2 | -2.6* | -2.8 | -2.5 |
| Uruguay | -0.8* | -0.8 | -0.7 | -1.6* | -1.8 | -1.3 | -1.3* | -1.4 | -1.2 |
| Canada | -0.2* | -0.3 | -0.2 | -2.6* | -2.9 | -2.4 | -1.4* | -1.5 | -1.2 |
| United States of America | 0 | 0 | 0 | -1.8* | -2 | -1.6 | 0.3 | -0.2 | 0.8 |
| Antigua and Barbuda | -2.7* | -2.9 | -2.5 | -2.8* | -3 | -2.7 | -0.8* | -0.9 | -0.8 |
| Bahamas | -1.8* | -1.9 | -1.6 | -2.7* | -2.9 | -2.5 | -0.5* | -0.5 | -0.4 |
| Barbados | -0.6* | -0.8 | -0.5 | -1.9* | -1.9 | -1.8 | -0.8* | -0.9 | -0.7 |
| Belize | -1.6* | -1.7 | -1.6 | -3.3* | -3.4 | -3.1 | -0.4* | -0.5 | -0.3 |
| Cuba | -2.5* | -3.1 | -1.8 | -2.1* | -2.3 | -1.9 | -1.1* | -1.2 | -1 |
| Dominica | -2.9* | -3.1 | -2.7 | -3.6* | -3.9 | -3.3 | -0.4* | -0.5 | -0.2 |
| Dominican Republic | -2.8* | -3 | -2.7 | -4.4* | -4.7 | -4 | -1.0* | -1 | -0.9 |
| Grenada | -3.7* | -3.9 | -3.6 | -3.6* | -4 | -3.3 | -0.5* | -0.6 | -0.4 |
| Guyana | -2.4* | -2.6 | -2.2 | -3.3* | -3.4 | -3.1 | -0.9* | -0.9 | -0.8 |
| Haiti | -0.9* | -1.2 | -0.6 | -2.4* | -2.5 | -2.4 | 0.1* | 0 | 0.1 |
| Jamaica | -2.1* | -2.2 | -1.9 | -3.3* | -3.4 | -3.1 | -0.4* | -0.5 | -0.3 |
| Saint Lucia | -1.5* | -1.5 | -1.4 | -2.8* | -3 | -2.6 | -0.6* | -0.7 | -0.6 |
| Saint Vincent and the Grenadines | -3.4* | -3.6 | -3.2 | -3.7* | -3.9 | -3.5 | -0.3* | -0.4 | -0.3 |
| Suriname | -3.3* | -3.7 | -2.8 | -3.2* | -3.3 | -3 | -0.5* | -0.6 | -0.5 |
| Trinidad and Tobago | -4.3* | -4.8 | -3.8 | -3.5* | -3.7 | -3.3 | -0.8* | -0.9 | -0.8 |
| Bolivia (Plurinational State of) | -1.1* | -1.1 | -1 | -1.6* | -1.8 | -1.4 | -0.8* | -0.8 | -0.7 |
| Ecuador | -0.5* | -0.6 | -0.4 | -2.7* | -3 | -2.4 | -3.2* | -3.3 | -3.1 |
| Peru | -0.7* | -0.8 | -0.6 | -3.2* | -3.3 | -3 | -2.2* | -2.3 | -2.1 |
| Colombia | -0.4* | -0.5 | -0.3 | -4.0* | -4.1 | -3.9 | -2.7* | -2.8 | -2.6 |
| Costa Rica | -0.5* | -0.6 | -0.4 | -3.3* | -3.4 | -3.2 | -1.5* | -1.5 | -1.4 |
| El Salvador | -0.9* | -1 | -0.8 | -4.7* | -5 | -4.3 | -1.1* | -1.3 | -1 |
| Guatemala | -0.1 | -0.2 | 0 | -3.9* | -4.1 | -3.8 | -1.0* | -1.2 | -0.7 |
| Honduras | -0.4* | -0.6 | -0.3 | -3.1* | -3.3 | -2.8 | -1.0* | -1 | -0.9 |
| Mexico | 0.1* | 0.1 | 0.2 | -3.2* | -3.3 | -3.2 | -0.9* | -1.1 | -0.8 |
| Nicaragua | -0.5* | -0.8 | -0.2 | -6.7* | -7.3 | -6.2 | -2.6* | -2.7 | -2.6 |
| Panama | -1.7* | -1.9 | -1.5 | -2.9* | -3.1 | -2.7 | -1.0* | -1.1 | -0.9 |
| Venezuela (Bolivarian Republic of) | 0.1 | -0.1 | 0.2 | -1.3* | -1.9 | -0.8 | -1.6* | -1.8 | -1.5 |
| Brazil | -0.4* | -0.5 | -0.4 | -3.2* | -3.3 | -3.1 | -1.1* | -1.1 | -1 |
| Paraguay | -1.1* | -1.2 | -1 | -2.8* | -2.9 | -2.6 | -0.9* | -1 | -0.9 |
| Algeria | -1.5* | -1.7 | -1.3 | -4.6* | -4.8 | -4.4 | -1.5* | -1.5 | -1.5 |
| Bahrain | -1.1* | -1.2 | -1.1 | -3.8* | -4 | -3.7 | -2.1* | -2.1 | -2 |
| Egypt | -3.6* | -3.9 | -3.3 | -3.8* | -4.2 | -3.5 | -1.1* | -1.1 | -1 |
| Iran (Islamic Republic of) | -1.9* | -2.1 | -1.7 | -7.0* | -7.6 | -6.5 | -2.1* | -2.2 | -2 |
| Iraq | -2.1* | -2.7 | -1.5 | -5.2* | -5.5 | -4.8 | -1.5* | -1.6 | -1.4 |
| Jordan | -1.4* | -1.6 | -1.3 | -3.7* | -4.1 | -3.3 | -1.7* | -1.8 | -1.7 |
| Kuwait | -1.0* | -1.1 | -0.9 | -4.3* | -4.5 | -4 | -1.1* | -1.2 | -1 |
| Lebanon | -2.5* | -2.9 | -2.1 | -4.8* | -5 | -4.5 | -3.1* | -3.3 | -3 |
| Libya | 0.1 | -0.3 | 0.5 | -4.6* | -5.2 | -4 | -0.9* | -1 | -0.8 |
| Morocco | -2.9* | -3.1 | -2.6 | -4.0* | -4.2 | -3.7 | -1.1* | -1.1 | -1 |
| Palestine | -1.7* | -1.9 | -1.6 | -6.8* | -7.2 | -6.4 | -1.2* | -1.3 | -1 |
| Oman | -1.4* | -1.5 | -1.3 | -7.5* | -8.1 | -7 | -1.7* | -1.8 | -1.5 |
| Qatar | -1.3* | -1.5 | -1.2 | -5.2* | -5.5 | -5 | -3.2* | -3.4 | -3 |
| Saudi Arabia | -1.3* | -1.4 | -1.2 | -8.9* | -9.7 | -8 | -1.8* | -2 | -1.7 |
| Syrian Arab Republic | -1.6* | -1.9 | -1.2 | -4.6* | -4.8 | -4.4 | -1.5* | -1.7 | -1.2 |
| Tunisia | -1.9* | -2 | -1.8 | -5.2* | -5.5 | -5 | -1.7* | -1.8 | -1.7 |
| Turkey | -3.4* | -3.7 | -3.1 | -5.2* | -5.3 | -5.1 | -2.4* | -2.5 | -2.2 |
| United Arab Emirates | -1.3* | -1.5 | -1.1 | -4.8* | -5.2 | -4.4 | -1.1* | -1.2 | -0.9 |
| Yemen | -0.7* | -1.2 | -0.1 | -4.5* | -4.8 | -4.2 | 0.8* | 0.7 | 0.9 |
| Afghanistan | -1.1* | -2.1 | -0.1 | -1.6* | -2.1 | -1.2 | -1.0* | -1.1 | -0.9 |
| Bangladesh | -2.1* | -2.9 | -1.4 | -4.3* | -4.6 | -4 | -0.9* | -1 | -0.9 |
| Bhutan | -1.3* | -1.8 | -0.9 | -5.0* | -5.1 | -5 | 0 | -0.1 | 0.1 |
| India | -1.9* | -2.3 | -1.4 | -4.4* | -4.7 | -4.1 | -0.3* | -0.4 | -0.3 |
| Nepal | 0.5 | -0.1 | 1.2 | -4.8* | -5 | -4.7 | -0.3* | -0.3 | -0.3 |
| Pakistan | 0.6* | 0.5 | 0.7 | -6.1* | -6.5 | -5.8 | -0.1* | -0.2 | 0 |
| Angola | -3.3* | -3.8 | -2.7 | -4.0* | -4.4 | -3.7 | 0 | -0.1 | 0 |
| Central African Republic | -2.3* | -2.7 | -1.8 | -1.1* | -1.2 | -1 | 0.2* | 0.1 | 0.3 |
| Republic of the Congo | -1.0* | -1.1 | -0.9 | -1.8* | -2.2 | -1.5 | -0.1* | -0.1 | 0 |
| Democratic Republic of the Congo | -0.7* | -1 | -0.3 | -0.6* | -1.2 | -0.1 | -0.6* | -0.6 | -0.5 |
| Equatorial Guinea | -8.1* | -9.1 | -7.1 | -9.9* | -10.4 | -9.4 | -1.3* | -1.4 | -1.2 |
| Gabon | -5.2* | -5.9 | -4.5 | -5.0* | -5.1 | -4.8 | 0 | -0.1 | 0.2 |
| Burundi | -2.5* | -2.6 | -2.3 | -2.0* | -2.2 | -1.8 | 0.1 | -0.1 | 0.2 |
| Comoros | -0.1* | -0.2 | -0.1 | -2.6* | -2.7 | -2.4 | -0.6* | -0.7 | -0.5 |
| Djibouti | -0.4 | -0.8 | 0.1 | -3.5* | -3.8 | -3.3 | -0.7* | -0.8 | -0.6 |
| Eritrea | -1.5* | -1.7 | -1.2 | -2.9* | -3 | -2.8 | -0.6* | -0.6 | -0.5 |
| Ethiopia | -1.0* | -1.6 | -0.4 | -2.9* | -3.3 | -2.5 | -0.6* | -0.7 | -0.6 |
| Kenya | 0.2 | -0.1 | 0.4 | -2.0* | -2.2 | -1.9 | 0.1* | 0.1 | 0.2 |
| Madagascar | 0.4* | 0.2 | 0.6 | -2.0* | -2.2 | -1.7 | -0.3* | -0.5 | -0.2 |
| Malawi | -1.6* | -1.9 | -1.2 | -2.6* | -2.9 | -2.4 | -0.2* | -0.3 | -0.1 |
| Mauritius | -2.8* | -2.8 | -2.7 | -6.0* | -6.3 | -5.7 | -1.0* | -1.1 | -1 |
| Mozambique | -3.0* | -3.4 | -2.6 | -2.9* | -3 | -2.8 | -0.3* | -0.3 | -0.2 |
| Rwanda | -2.0* | -2.4 | -1.6 | -3.1* | -3.5 | -2.8 | -0.6* | -0.7 | -0.5 |
| Seychelles | -2.4* | -2.5 | -2.4 | -5.3* | -5.9 | -4.8 | -1.5* | -1.6 | -1.5 |
| Somalia | 0.3* | 0.2 | 0.4 | -0.3* | -0.3 | -0.3 | -0.4* | -0.4 | -0.3 |
| United Republic of Tanzania | -1.7* | -1.9 | -1.5 | -2.4* | -2.6 | -2.1 | -0.5* | -0.6 | -0.4 |
| Uganda | -2.0* | -2.1 | -1.8 | -3.6* | -3.8 | -3.4 | -0.5* | -0.7 | -0.4 |
| Zambia | -1.9* | -2.2 | -1.5 | -3.8* | -4.2 | -3.3 | 0.6* | 0.5 | 0.7 |
| Botswana | -0.6* | -0.7 | -0.5 | -3.5* | -3.6 | -3.3 | -0.9* | -1 | -0.8 |
| Lesotho | -4.3* | -5.2 | -3.4 | -3.0* | -3.1 | -2.8 | 0 | -0.1 | 0.1 |
| Namibia | -1.5* | -1.6 | -1.4 | -3.0* | -3.2 | -2.8 | -1.2* | -1.3 | -1 |
| South Africa | -0.9* | -1 | -0.9 | -4.3* | -4.4 | -4.2 | -0.9* | -1.1 | -0.7 |
| Eswatini | -1.0* | -1 | -0.9 | -3.7* | -3.8 | -3.6 | -0.5* | -0.6 | -0.3 |
| Zimbabwe | -0.3 | -0.7 | 0.1 | -0.6* | -1 | -0.3 | 0.7* | 0.5 | 0.9 |
| Benin | -1.1* | -1.2 | -1 | -2.1* | -2.2 | -2 | -0.1 | -0.2 | 0 |
| Burkina Faso | -2.2* | -2.3 | -2 | -2.8* | -2.9 | -2.7 | 1.1* | 1 | 1.3 |
| Cameroon | -0.3* | -0.5 | -0.2 | -3.1* | -3.3 | -2.9 | 0.4* | 0.3 | 0.6 |
| Cabo Verde | -1.8* | -1.9 | -1.6 | -6.1* | -6.3 | -5.8 | -0.4* | -0.5 | -0.3 |
| Chad | -2.9* | -3 | -2.7 | -1.9* | -2 | -1.8 | 0 | 0 | 0.1 |
| Côte d'Ivoire | -2.1* | -2.3 | -2 | -3.2* | -3.3 | -3 | 0.1 | 0 | 0.3 |
| Gambia | -1.5* | -1.9 | -1.2 | -2.9* | -3 | -2.9 | 0 | 0 | 0.1 |
| Ghana | -3.1* | -3.3 | -2.8 | -3.7* | -3.9 | -3.5 | 0.6* | 0.5 | 0.7 |
| Guinea | -4.0* | -4.2 | -3.7 | -2.5* | -2.5 | -2.4 | 0.3* | 0.3 | 0.3 |
| Guinea-Bissau | -1.8* | -2 | -1.6 | -2.4* | -2.5 | -2.4 | 0.7* | 0.6 | 0.8 |
| Liberia | -1.4* | -1.9 | -0.9 | -3.7* | -4 | -3.4 | -0.6* | -0.7 | -0.6 |
| Mali | -1.9* | -2 | -1.8 | -2.3* | -2.4 | -2.3 | 0.6* | 0.4 | 0.8 |
| Mauritania | -1.9* | -2.1 | -1.6 | -3.8* | -3.9 | -3.7 | -0.3* | -0.4 | -0.3 |
| Niger | -0.5* | -0.7 | -0.4 | -1.2* | -1.3 | -1.1 | -0.4* | -0.6 | -0.3 |
| Nigeria | -1.7* | -2.2 | -1.2 | -4.4* | -4.9 | -3.8 | 0.4* | 0.3 | 0.5 |
| Sao Tome and Principe | -1.1* | -1.4 | -0.8 | -4.6* | -4.7 | -4.5 | 0.2 | -0.3 | 0.7 |
| Senegal | -2.1* | -2.4 | -1.9 | -4.3* | -4.8 | -3.9 | -0.1* | -0.1 | 0 |
| Sierra Leone | -1.0* | -1.5 | -0.5 | -2.6* | -2.8 | -2.5 | 0.1* | 0.1 | 0.2 |
| Togo | -2.4* | -2.7 | -2 | -2.8* | -3 | -2.7 | 0.2* | 0 | 0.4 |
| American Samoa | -1.2* | -1.3 | -1.1 | -1.9* | -2.1 | -1.6 | -0.3* | -0.4 | -0.3 |
| Bermuda | -2.4* | -2.5 | -2.2 | -2.7* | -2.9 | -2.6 | -1.8* | -1.9 | -1.6 |
| Cook Islands | -1.4* | -1.5 | -1.3 | -2.9* | -3.1 | -2.7 | -0.7* | -0.7 | -0.6 |
| Greenland | 0 | -0.1 | 0 | -1.8* | -2.1 | -1.5 | -1.0* | -1 | -0.9 |
| Guam | -1.1* | -1.2 | -0.9 | -2.6* | -2.8 | -2.4 | 0 | -0.1 | 0.2 |
| Monaco | 0.1* | 0.1 | 0.2 | -1.5* | -1.7 | -1.2 | -1.2* | -1.3 | -1.2 |
| Nauru | -1.0* | -1.7 | -0.3 | -1.0* | -1.7 | -0.3 | -0.2* | -0.4 | 0 |
| Niue | -1.9* | -2.2 | -1.7 | -3.0* | -3.2 | -2.9 | -0.9* | -0.9 | -0.8 |
| Northern Mariana Islands | -1.0* | -1.1 | -0.8 | -0.4* | -0.6 | -0.1 | -0.3* | -0.4 | -0.1 |
| Palau | -1.6* | -1.8 | -1.5 | -2.3* | -2.4 | -2.1 | -0.6* | -0.7 | -0.5 |
| Puerto Rico | -2.6* | -2.7 | -2.5 | -3.2* | -3.3 | -3.1 | -1.2* | -1.4 | -1.1 |
| Saint Kitts and Nevis | -3.3* | -3.4 | -3.2 | -3.4* | -3.6 | -3.3 | -0.8* | -0.9 | -0.7 |
| San Marino | 0.1* | 0.1 | 0.1 | -1.8* | -2.2 | -1.5 | -1.4* | -1.6 | -1.3 |
| Tokelau | -2.8* | -3.1 | -2.5 | -3.0* | -3.2 | -2.8 | -0.8* | -0.9 | -0.8 |
| Tuvalu | -2.9* | -3.1 | -2.7 | -1.9* | -2 | -1.7 | -0.5* | -0.5 | -0.4 |
| United States Virgin Islands | -3.0* | -3.3 | -2.7 | -4.0* | -4.2 | -3.8 | -0.9* | -1 | -0.7 |
| South Sudan | 0.5* | 0.3 | 0.6 | -2.3* | -2.5 | -2.2 | -0.3* | -0.3 | -0.2 |
| Sudan | -3.1* | -3.6 | -2.6 | -4.5* | -4.8 | -4.2 | -0.8* | -0.8 | -0.7 |

**Supplementary Table 3.** Age-standarised prevalence, incidence, and DALYs rate of iodine deficiency, vitamin A deficiency, and dietary iron deficiency for 204 countries and territories in 2019.

| Measure | Location | Micronutrient deficiency | Rate | 95% Upper UI | 95% Lower UI |
| --- | --- | --- | --- | --- | --- |
| DALYs | Kiribati | Iodine deficiency | 2.680918 | 4.304584 | 1.559831 |
| DALYs | Kiribati | Vitamin A deficiency | 34.82003 | 52.41676 | 21.59498 |
| DALYs | Kiribati | Dietary iron deficiency | 632.0961 | 939.8403 | 421.4115 |
| DALYs | Equatorial Guinea | Iodine deficiency | 62.43435 | 118.1105 | 30.12446 |
| DALYs | Equatorial Guinea | Vitamin A deficiency | 20.50817 | 30.29079 | 12.90434 |
| DALYs | Equatorial Guinea | Dietary iron deficiency | 348.2785 | 536.4745 | 205.3888 |
| DALYs | Luxembourg | Iodine deficiency | 7.717164 | 15.27015 | 3.541129 |
| DALYs | Luxembourg | Vitamin A deficiency | 0.140431 | 0.31244 | 0.050659 |
| DALYs | Luxembourg | Dietary iron deficiency | 31.98302 | 56.76878 | 16.96246 |
| DALYs | Cyprus | Vitamin A deficiency | 0.255002 | 0.585072 | 0.095337 |
| DALYs | Cyprus | Dietary iron deficiency | 29.52703 | 53.47225 | 16.66677 |
| DALYs | Malaysia | Iodine deficiency | 13.6674 | 22.34548 | 7.407953 |
| DALYs | Malaysia | Vitamin A deficiency | 0.476039 | 0.872626 | 0.24063 |
| DALYs | Marshall Islands | Iodine deficiency | 1.771302 | 2.830492 | 1.074637 |
| DALYs | Marshall Islands | Vitamin A deficiency | 28.9109 | 44.37668 | 18.12981 |
| DALYs | Marshall Islands | Dietary iron deficiency | 476.3176 | 697.0892 | 313.4265 |
| DALYs | Mauritius | Iodine deficiency | 7.32569 | 11.83386 | 4.293625 |
| DALYs | Mauritius | Vitamin A deficiency | 4.31315 | 6.654016 | 2.63019 |
| DALYs | Mauritius | Dietary iron deficiency | 248.0232 | 379.091 | 155.7259 |
| DALYs | Senegal | Iodine deficiency | 27.75519 | 46.28623 | 15.30273 |
| DALYs | Senegal | Vitamin A deficiency | 29.86356 | 45.55146 | 18.45265 |
| DALYs | Senegal | Dietary iron deficiency | 756.3456 | 1100.569 | 499.041 |
| DALYs | Qatar | Iodine deficiency | 3.152977 | 5.901 | 1.420987 |
| DALYs | Qatar | Vitamin A deficiency | 0.38291 | 0.722762 | 0.173051 |
| DALYs | Qatar | Dietary iron deficiency | 84.38691 | 140.7458 | 47.17103 |
| DALYs | Azerbaijan | Iodine deficiency | 2.216017 | 4.26198 | 1.004606 |
| DALYs | Azerbaijan | Vitamin A deficiency | 1.793695 | 2.945963 | 0.959745 |
| DALYs | Azerbaijan | Dietary iron deficiency | 319.1485 | 480.5812 | 203.1735 |
| DALYs | Greenland | Iodine deficiency | 2.261134 | 4.272961 | 1.037464 |
| DALYs | Greenland | Vitamin A deficiency | 0.51028 | 0.972076 | 0.220592 |
| DALYs | Bulgaria | Iodine deficiency | 1.525285 | 2.926839 | 0.685012 |
| DALYs | Bulgaria | Vitamin A deficiency | 4.434171 | 7.636624 | 2.314894 |
| DALYs | Bulgaria | Dietary iron deficiency | 173.7763 | 271.1331 | 106.835 |
| DALYs | Namibia | Dietary iron deficiency | 320.3552 | 484.402 | 200.4209 |
| DALYs | Malaysia | Dietary iron deficiency | 292.3707 | 437.7032 | 184.9486 |
| DALYs | Cuba | Iodine deficiency | 6.886611 | 11.44586 | 3.564698 |
| DALYs | Cuba | Vitamin A deficiency | 1.441872 | 2.657927 | 0.702956 |
| DALYs | Cuba | Dietary iron deficiency | 211.2172 | 334.1016 | 121.3917 |
| DALYs | Gambia | Iodine deficiency | 48.83703 | 85.64773 | 27.48946 |
| DALYs | Gambia | Vitamin A deficiency | 52.8354 | 77.71385 | 34.71552 |
| DALYs | Gambia | Dietary iron deficiency | 820.4181 | 1198.637 | 527.0608 |
| DALYs | Bolivia (Plurinational State of) | Iodine deficiency | 2.002494 | 3.343632 | 1.12547 |
| DALYs | Bolivia (Plurinational State of) | Vitamin A deficiency | 12.49346 | 18.66694 | 7.847737 |
| DALYs | Maldives | Iodine deficiency | 4.47081 | 7.217149 | 2.53415 |
| DALYs | Maldives | Vitamin A deficiency | 6.959362 | 11.26772 | 3.987206 |
| DALYs | Maldives | Dietary iron deficiency | 227.64 | 350.4174 | 138.4938 |
| DALYs | Saint Kitts and Nevis | Iodine deficiency | 3.391727 | 5.783781 | 1.673449 |
| DALYs | Saint Kitts and Nevis | Vitamin A deficiency | 5.203474 | 7.896991 | 3.23779 |
| DALYs | Croatia | Iodine deficiency | 1.894857 | 3.592163 | 0.87792 |
| DALYs | Croatia | Vitamin A deficiency | 2.194776 | 3.857782 | 1.09366 |
| DALYs | Croatia | Dietary iron deficiency | 95.1428 | 152.7931 | 56.3212 |
| DALYs | Georgia | Iodine deficiency | 3.152901 | 6.12636 | 1.389693 |
| DALYs | Georgia | Vitamin A deficiency | 1.819698 | 3.199599 | 0.919945 |
| DALYs | Georgia | Dietary iron deficiency | 321.1065 | 467.9714 | 205.8479 |
| DALYs | Saint Kitts and Nevis | Dietary iron deficiency | 249.2534 | 379.6785 | 154.1077 |
| DALYs | Brunei Darussalam | Iodine deficiency | 2.562933 | 4.882481 | 1.163757 |
| DALYs | Brunei Darussalam | Vitamin A deficiency | 0.829971 | 1.454206 | 0.403398 |
| DALYs | Brunei Darussalam | Dietary iron deficiency | 200.1555 | 315.4058 | 125.0594 |
| DALYs | Czechia | Iodine deficiency | 1.131757 | 2.163075 | 0.49566 |
| DALYs | Czechia | Vitamin A deficiency | 1.935517 | 3.453397 | 0.968458 |
| DALYs | Czechia | Dietary iron deficiency | 108.1847 | 174.8663 | 63.89491 |
| DALYs | Belarus | Iodine deficiency | 2.992767 | 4.915939 | 1.657259 |
| DALYs | Belarus | Vitamin A deficiency | 0.281071 | 0.545344 | 0.117296 |
| DALYs | Belarus | Dietary iron deficiency | 115.4871 | 183.055 | 69.60054 |
| DALYs | Malta | Iodine deficiency | 8.481021 | 16.66757 | 3.912506 |
| DALYs | Malta | Vitamin A deficiency | 0.346315 | 0.732953 | 0.134979 |
| DALYs | Malta | Dietary iron deficiency | 43.30919 | 74.28155 | 23.94029 |
| DALYs | Sierra Leone | Iodine deficiency | 25.44751 | 40.93792 | 14.20724 |
| DALYs | Sierra Leone | Vitamin A deficiency | 57.73495 | 83.64435 | 37.68982 |
| DALYs | Sierra Leone | Dietary iron deficiency | 585.11 | 871.092 | 374.4074 |
| DALYs | Syrian Arab Republic | Iodine deficiency | 32.88334 | 52.00297 | 18.73244 |
| DALYs | Syrian Arab Republic | Vitamin A deficiency | 3.647427 | 6.520103 | 1.877124 |
| DALYs | Togo | Iodine deficiency | 34.51962 | 57.59158 | 19.10145 |
| DALYs | Togo | Vitamin A deficiency | 45.4106 | 68.04596 | 29.2723 |
| DALYs | Togo | Dietary iron deficiency | 559.5942 | 830.4718 | 365.4122 |
| DALYs | Egypt | Iodine deficiency | 16.0543 | 24.75214 | 9.381634 |
| DALYs | Egypt | Vitamin A deficiency | 3.83519 | 5.95435 | 2.353339 |
| DALYs | Egypt | Dietary iron deficiency | 210.5343 | 326.5681 | 127.3078 |
| DALYs | China | Iodine deficiency | 15.56575 | 30.66884 | 7.164371 |
| DALYs | China | Vitamin A deficiency | 3.691773 | 5.551212 | 2.342691 |
| DALYs | China | Dietary iron deficiency | 80.69197 | 122.7274 | 51.1196 |
| DALYs | Pakistan | Iodine deficiency | 85.01445 | 148.2696 | 47.21139 |
| DALYs | Pakistan | Vitamin A deficiency | 16.05873 | 23.37229 | 10.43974 |
| DALYs | Pakistan | Dietary iron deficiency | 796.3678 | 1157.767 | 514.0666 |
| DALYs | Singapore | Iodine deficiency | 2.547099 | 4.937335 | 1.145406 |
| DALYs | Singapore | Vitamin A deficiency | 0.282743 | 0.55011 | 0.119454 |
| DALYs | Singapore | Dietary iron deficiency | 106.0103 | 167.9623 | 61.97064 |
| DALYs | San Marino | Iodine deficiency | 8.094296 | 15.94241 | 3.722755 |
| DALYs | San Marino | Vitamin A deficiency | 0.151354 | 0.343741 | 0.054491 |
| DALYs | San Marino | Dietary iron deficiency | 32.59554 | 55.61124 | 16.80659 |
| DALYs | Dominica | Iodine deficiency | 5.796592 | 9.748105 | 2.880901 |
| DALYs | Dominica | Vitamin A deficiency | 5.085146 | 7.735815 | 3.148596 |
| DALYs | Dominica | Dietary iron deficiency | 283.722 | 429.3311 | 177.039 |
| DALYs | Seychelles | Iodine deficiency | 3.229832 | 6.207636 | 1.493664 |
| DALYs | Seychelles | Vitamin A deficiency | 3.660276 | 5.78054 | 2.200529 |
| DALYs | Seychelles | Dietary iron deficiency | 210.1272 | 325.5319 | 126.6525 |
| DALYs | Tokelau | Iodine deficiency | 0.562529 | 1.10244 | 0.239219 |
| DALYs | Tokelau | Vitamin A deficiency | 12.62757 | 19.91408 | 7.416334 |
| DALYs | Tokelau | Dietary iron deficiency | 355.2453 | 531.4206 | 228.3033 |
| DALYs | Democratic People's Republic of Korea | Dietary iron deficiency | 280.1265 | 420.2735 | 171.9051 |
| DALYs | Ghana | Iodine deficiency | 31.33733 | 54.50733 | 17.04469 |
| DALYs | Ghana | Vitamin A deficiency | 35.78727 | 52.75741 | 22.11762 |
| DALYs | Ghana | Dietary iron deficiency | 499.6198 | 735.5154 | 313.6778 |
| DALYs | Dominican Republic | Iodine deficiency | 6.736226 | 11.4506 | 3.30839 |
| DALYs | Dominican Republic | Vitamin A deficiency | 6.770188 | 10.35241 | 4.083033 |
| DALYs | Dominican Republic | Dietary iron deficiency | 235.6721 | 359.1526 | 145.9977 |
| DALYs | Montenegro | Iodine deficiency | 1.970222 | 3.727276 | 0.899743 |
| DALYs | Montenegro | Vitamin A deficiency | 2.749646 | 4.983747 | 1.481823 |
| DALYs | Venezuela (Bolivarian Republic of) | Iodine deficiency | 9.522744 | 15.87134 | 4.660283 |
| DALYs | Venezuela (Bolivarian Republic of) | Vitamin A deficiency | 5.429103 | 8.167094 | 3.299075 |
| DALYs | Greenland | Dietary iron deficiency | 125.8282 | 192.4029 | 75.18893 |
| DALYs | Venezuela (Bolivarian Republic of) | Dietary iron deficiency | 108.9399 | 172.9052 | 66.97376 |
| DALYs | Ecuador | Iodine deficiency | 0.812465 | 1.617363 | 0.350551 |
| DALYs | Ecuador | Vitamin A deficiency | 5.28448 | 8.242392 | 3.330937 |
| DALYs | Ecuador | Dietary iron deficiency | 97.0261 | 148.7447 | 62.45903 |
| DALYs | Tajikistan | Iodine deficiency | 13.26402 | 21.25274 | 7.5912 |
| DALYs | Tajikistan | Vitamin A deficiency | 12.18661 | 17.86059 | 7.675647 |
| DALYs | Tajikistan | Dietary iron deficiency | 364.9947 | 530.9712 | 236.6886 |
| DALYs | Uruguay | Iodine deficiency | 1.531701 | 2.864142 | 0.69476 |
| DALYs | Uruguay | Vitamin A deficiency | 4.18214 | 7.719581 | 2.047507 |
| DALYs | Uruguay | Dietary iron deficiency | 131.8023 | 212.398 | 77.28086 |
| DALYs | Tuvalu | Iodine deficiency | 1.705057 | 2.710254 | 1.024293 |
| DALYs | Tuvalu | Vitamin A deficiency | 19.09966 | 30.02756 | 11.42138 |
| DALYs | Tuvalu | Dietary iron deficiency | 446.1253 | 655.9004 | 285.1024 |
| DALYs | Chile | Iodine deficiency | 1.494802 | 2.889057 | 0.658296 |
| DALYs | Chile | Vitamin A deficiency | 0.428504 | 0.998779 | 0.156954 |
| DALYs | Chile | Dietary iron deficiency | 38.90541 | 64.06771 | 22.71034 |
| DALYs | Brazil | Iodine deficiency | 1.116081 | 2.143479 | 0.499454 |
| DALYs | Brazil | Vitamin A deficiency | 10.22265 | 15.15666 | 6.202424 |
| DALYs | Brazil | Dietary iron deficiency | 310.3023 | 470.0668 | 198.9828 |
| DALYs | Turkmenistan | Iodine deficiency | 3.069649 | 5.753675 | 1.365984 |
| DALYs | Turkmenistan | Vitamin A deficiency | 7.806528 | 11.62698 | 4.999346 |
| DALYs | Turkmenistan | Dietary iron deficiency | 319.2863 | 478.5415 | 202.9269 |
| DALYs | United Arab Emirates | Vitamin A deficiency | 0.577122 | 1.023706 | 0.277155 |
| DALYs | United Arab Emirates | Dietary iron deficiency | 172.4601 | 264.8027 | 98.6001 |
| DALYs | Guinea | Iodine deficiency | 40.6991 | 70.67638 | 22.29826 |
| DALYs | Guinea | Vitamin A deficiency | 51.4348 | 74.77223 | 33.00881 |
| DALYs | Guinea | Dietary iron deficiency | 521.3342 | 758.5076 | 341.8359 |
| DALYs | Democratic People's Republic of Korea | Iodine deficiency | 1.917257 | 3.179896 | 1.076649 |
| DALYs | Democratic People's Republic of Korea | Vitamin A deficiency | 8.822544 | 14.70277 | 4.91605 |
| DALYs | United Arab Emirates | Iodine deficiency | 10.94505 | 20.93799 | 4.953967 |
| DALYs | Peru | Iodine deficiency | 0.79694 | 1.562785 | 0.34229 |
| DALYs | Peru | Vitamin A deficiency | 9.724773 | 14.62635 | 6.219218 |
| DALYs | Peru | Dietary iron deficiency | 215.7112 | 331.1419 | 135.3957 |
| DALYs | France | Iodine deficiency | 8.208438 | 16.21185 | 3.744336 |
| DALYs | France | Vitamin A deficiency | 0.053656 | 0.110923 | 0.02091 |
| DALYs | Estonia | Iodine deficiency | 1.976844 | 3.272532 | 1.069792 |
| DALYs | Estonia | Vitamin A deficiency | 0.137584 | 0.263189 | 0.056248 |
| DALYs | Estonia | Dietary iron deficiency | 100.2104 | 164.3151 | 61.84323 |
| DALYs | Grenada | Iodine deficiency | 4.760215 | 8.042429 | 2.352631 |
| DALYs | Grenada | Vitamin A deficiency | 7.17368 | 10.80622 | 4.515854 |
| DALYs | Grenada | Dietary iron deficiency | 314.1469 | 475.8544 | 193.163 |
| DALYs | Somalia | Iodine deficiency | 274.3838 | 481.4295 | 152.6752 |
| DALYs | Somalia | Vitamin A deficiency | 80.92423 | 118.4527 | 52.54456 |
| DALYs | Somalia | Dietary iron deficiency | 739.8155 | 1092.84 | 492.0116 |
| DALYs | Poland | Iodine deficiency | 1.955807 | 3.744241 | 0.886018 |
| DALYs | Poland | Vitamin A deficiency | 2.660581 | 4.666438 | 1.31135 |
| DALYs | Poland | Dietary iron deficiency | 153.2274 | 243.2915 | 91.30561 |
| DALYs | Guyana | Iodine deficiency | 9.633539 | 15.86329 | 5.095504 |
| DALYs | Guyana | Vitamin A deficiency | 9.294554 | 13.85353 | 5.516945 |
| DALYs | Guyana | Dietary iron deficiency | 510.155 | 738.3894 | 320.1141 |
| DALYs | Yemen | Iodine deficiency | 68.1652 | 103.8951 | 41.56676 |
| DALYs | Yemen | Vitamin A deficiency | 36.30049 | 53.57101 | 23.66938 |
| DALYs | Yemen | Dietary iron deficiency | 801.6066 | 1178.651 | 541.4635 |
| DALYs | Japan | Iodine deficiency | 2.817899 | 5.37114 | 1.269429 |
| DALYs | Japan | Vitamin A deficiency | 0.364046 | 0.675364 | 0.169765 |
| DALYs | Kuwait | Iodine deficiency | 3.710969 | 6.92257 | 1.670596 |
| DALYs | Kuwait | Vitamin A deficiency | 5.018461 | 7.743626 | 3.053701 |
| DALYs | Kuwait | Dietary iron deficiency | 177.4147 | 270.325 | 108.4016 |
| DALYs | Guam | Iodine deficiency | 0.47196 | 0.933835 | 0.210479 |
| DALYs | Guam | Vitamin A deficiency | 3.08354 | 5.085297 | 1.568866 |
| DALYs | Guam | Dietary iron deficiency | 296.5019 | 448.6822 | 179.6858 |
| DALYs | Australia | Iodine deficiency | 2.149868 | 4.150463 | 0.974199 |
| DALYs | Australia | Vitamin A deficiency | 0.00889 | 0.017745 | 0.003882 |
| DALYs | Australia | Dietary iron deficiency | 60.72587 | 102.4438 | 33.48867 |
| DALYs | Philippines | Iodine deficiency | 33.26609 | 58.42204 | 18.45814 |
| DALYs | Philippines | Vitamin A deficiency | 13.16073 | 20.504 | 8.010134 |
| DALYs | Philippines | Dietary iron deficiency | 196.011 | 296.8948 | 123.4432 |
| DALYs | Spain | Iodine deficiency | 8.199949 | 15.78119 | 3.724809 |
| DALYs | Spain | Vitamin A deficiency | 0.271257 | 0.595835 | 0.098854 |
| DALYs | Spain | Dietary iron deficiency | 57.03208 | 101.7348 | 29.77785 |
| DALYs | Colombia | Iodine deficiency | 9.252283 | 15.35887 | 4.557805 |
| DALYs | Colombia | Vitamin A deficiency | 4.306368 | 6.493805 | 2.643984 |
| DALYs | Colombia | Dietary iron deficiency | 90.22566 | 139.8167 | 55.20482 |
| DALYs | Solomon Islands | Iodine deficiency | 2.181898 | 3.43188 | 1.337419 |
| DALYs | Solomon Islands | Vitamin A deficiency | 37.67582 | 57.26519 | 23.03728 |
| DALYs | Liberia | Iodine deficiency | 15.46205 | 24.7767 | 9.171913 |
| DALYs | Liberia | Vitamin A deficiency | 27.80437 | 42.84262 | 17.41085 |
| DALYs | Liberia | Dietary iron deficiency | 324.3039 | 488.7991 | 191.9981 |
| DALYs | Haiti | Iodine deficiency | 23.04617 | 37.21068 | 12.85033 |
| DALYs | Taiwan (Province of China) | Iodine deficiency | 0.961318 | 1.900328 | 0.434497 |
| DALYs | Taiwan (Province of China) | Vitamin A deficiency | 0.323184 | 0.632329 | 0.133192 |
| DALYs | Taiwan (Province of China) | Dietary iron deficiency | 95.55064 | 156.6891 | 57.4244 |
| DALYs | Solomon Islands | Dietary iron deficiency | 480.2585 | 713.1096 | 310.1989 |
| DALYs | Lebanon | Iodine deficiency | 14.03287 | 26.79895 | 6.33603 |
| DALYs | Lebanon | Vitamin A deficiency | 1.047845 | 2.051904 | 0.510759 |
| DALYs | Lebanon | Dietary iron deficiency | 81.34096 | 137.0643 | 45.24695 |
| DALYs | Netherlands | Iodine deficiency | 7.719536 | 15.39979 | 3.508081 |
| DALYs | Netherlands | Vitamin A deficiency | 0.142384 | 0.342909 | 0.051697 |
| DALYs | Netherlands | Dietary iron deficiency | 28.27152 | 50.07751 | 14.88061 |
| DALYs | Haiti | Vitamin A deficiency | 26.12072 | 39.39106 | 16.68108 |
| DALYs | Haiti | Dietary iron deficiency | 674.7773 | 995.7651 | 449.2381 |
| DALYs | Russian Federation | Iodine deficiency | 3.696369 | 6.081617 | 2.023601 |
| DALYs | Tunisia | Iodine deficiency | 7.17524 | 11.48404 | 4.171743 |
| DALYs | Tunisia | Vitamin A deficiency | 4.663756 | 7.102 | 2.916069 |
| DALYs | Tunisia | Dietary iron deficiency | 102.2883 | 163.4129 | 59.28667 |
| DALYs | Sudan | Iodine deficiency | 42.25368 | 63.89419 | 24.96132 |
| DALYs | Sudan | Vitamin A deficiency | 18.5907 | 27.84251 | 11.3473 |
| DALYs | Sudan | Dietary iron deficiency | 337.6947 | 507.6687 | 217.0051 |
| DALYs | Sri Lanka | Iodine deficiency | 9.644634 | 19.30864 | 4.387843 |
| DALYs | Sri Lanka | Vitamin A deficiency | 4.702718 | 7.514875 | 2.717802 |
| DALYs | Russian Federation | Vitamin A deficiency | 0.039484 | 0.076077 | 0.018154 |
| DALYs | Russian Federation | Dietary iron deficiency | 124.8351 | 191.8513 | 77.32793 |
| DALYs | Monaco | Iodine deficiency | 7.331161 | 14.25103 | 3.381649 |
| DALYs | Monaco | Vitamin A deficiency | 0.10117 | 0.235862 | 0.040525 |
| DALYs | Monaco | Dietary iron deficiency | 30.1589 | 53.62231 | 15.926 |
| DALYs | Zimbabwe | Iodine deficiency | 31.73534 | 63.16298 | 14.61925 |
| DALYs | Zimbabwe | Vitamin A deficiency | 31.88537 | 48.24645 | 20.66659 |
| DALYs | Costa Rica | Iodine deficiency | 4.85932 | 9.339661 | 2.178843 |
| DALYs | Costa Rica | Vitamin A deficiency | 4.077483 | 6.249367 | 2.50782 |
| DALYs | Costa Rica | Dietary iron deficiency | 112.1451 | 177.6539 | 66.781 |
| DALYs | Timor-Leste | Iodine deficiency | 8.483346 | 13.5034 | 4.972857 |
| DALYs | Timor-Leste | Vitamin A deficiency | 18.81557 | 28.45456 | 11.58185 |
| DALYs | Timor-Leste | Dietary iron deficiency | 332.2616 | 492.5947 | 210.7119 |
| DALYs | Norway | Iodine deficiency | 8.056636 | 15.73699 | 3.723231 |
| DALYs | Norway | Vitamin A deficiency | 0.156634 | 0.33474 | 0.061116 |
| DALYs | Norway | Dietary iron deficiency | 34.57812 | 64.00956 | 18.17838 |
| DALYs | Sri Lanka | Dietary iron deficiency | 223.3573 | 345.4967 | 133.4113 |
| DALYs | Angola | Iodine deficiency | 87.74652 | 164.4267 | 43.12869 |
| DALYs | Romania | Iodine deficiency | 4.808315 | 9.037413 | 2.161934 |
| DALYs | Romania | Vitamin A deficiency | 3.998582 | 6.477453 | 2.15288 |
| DALYs | Romania | Dietary iron deficiency | 171.5678 | 266.0533 | 104.4084 |
| DALYs | Nauru | Dietary iron deficiency | 380.461 | 584.633 | 241.7262 |
| DALYs | Jamaica | Vitamin A deficiency | 5.76838 | 8.889353 | 3.610084 |
| DALYs | Jamaica | Dietary iron deficiency | 309.9634 | 470.3563 | 193.25 |
| DALYs | Kenya | Iodine deficiency | 12.83415 | 25.00195 | 5.928358 |
| DALYs | Kenya | Vitamin A deficiency | 34.94966 | 51.89068 | 22.69603 |
| DALYs | Kenya | Dietary iron deficiency | 332.9146 | 475.0111 | 224.2041 |
| DALYs | Viet Nam | Iodine deficiency | 13.93484 | 22.33389 | 7.925831 |
| DALYs | Viet Nam | Vitamin A deficiency | 3.59519 | 5.693243 | 2.149822 |
| DALYs | Viet Nam | Dietary iron deficiency | 152.5127 | 240.9133 | 93.02705 |
| DALYs | Tonga | Iodine deficiency | 0.606528 | 1.180182 | 0.265262 |
| DALYs | Tonga | Vitamin A deficiency | 11.92154 | 19.08529 | 7.070238 |
| DALYs | Tonga | Dietary iron deficiency | 371.4014 | 564.1178 | 234.186 |
| DALYs | Uzbekistan | Iodine deficiency | 11.07118 | 18.33266 | 6.216155 |
| DALYs | Uzbekistan | Vitamin A deficiency | 8.947256 | 13.19539 | 5.634366 |
| DALYs | Uzbekistan | Dietary iron deficiency | 579.7073 | 846.3915 | 371.183 |
| DALYs | Libya | Iodine deficiency | 23.26404 | 37.13836 | 13.26724 |
| DALYs | Libya | Vitamin A deficiency | 7.038585 | 10.4039 | 4.428443 |
| DALYs | Libya | Dietary iron deficiency | 196.3181 | 304.9714 | 119.3605 |
| DALYs | Antigua and Barbuda | Iodine deficiency | 3.446901 | 5.817438 | 1.700563 |
| DALYs | Antigua and Barbuda | Vitamin A deficiency | 5.434831 | 8.14012 | 3.401685 |
| DALYs | Antigua and Barbuda | Dietary iron deficiency | 280.8728 | 426.5961 | 173.6605 |
| DALYs | Ukraine | Iodine deficiency | 12.59146 | 19.91015 | 6.995888 |
| DALYs | Ukraine | Vitamin A deficiency | 0.272058 | 0.513438 | 0.114619 |
| DALYs | Ukraine | Dietary iron deficiency | 89.80092 | 144.6015 | 54.91538 |
| DALYs | United States Virgin Islands | Iodine deficiency | 2.296011 | 4.025379 | 1.01988 |
| DALYs | United States Virgin Islands | Vitamin A deficiency | 0.760237 | 1.342406 | 0.380911 |
| DALYs | United States Virgin Islands | Dietary iron deficiency | 255.3147 | 394.4317 | 153.0703 |
| DALYs | Thailand | Iodine deficiency | 9.689733 | 15.46244 | 5.774052 |
| DALYs | Thailand | Vitamin A deficiency | 5.187257 | 8.517548 | 3.033882 |
| DALYs | Thailand | Dietary iron deficiency | 118.0864 | 191.6202 | 71.48128 |
| DALYs | Paraguay | Iodine deficiency | 1.226544 | 2.370904 | 0.541377 |
| DALYs | Paraguay | Vitamin A deficiency | 9.040475 | 13.49143 | 5.578191 |
| DALYs | Paraguay | Dietary iron deficiency | 255.0262 | 378.9472 | 158.0592 |
| DALYs | Portugal | Iodine deficiency | 12.67243 | 25.22249 | 5.936444 |
| DALYs | Portugal | Vitamin A deficiency | 0.500083 | 1.121245 | 0.197577 |
| DALYs | Portugal | Dietary iron deficiency | 43.5823 | 78.9074 | 22.94995 |
| DALYs | Serbia | Dietary iron deficiency | 152.4776 | 244.9312 | 89.07045 |
| DALYs | Sweden | Iodine deficiency | 8.522923 | 16.94184 | 3.961679 |
| DALYs | Sweden | Vitamin A deficiency | 0.176014 | 0.369717 | 0.065257 |
| DALYs | Sweden | Dietary iron deficiency | 35.39677 | 61.24691 | 19.01448 |
| DALYs | Morocco | Iodine deficiency | 30.75239 | 48.99471 | 17.60996 |
| DALYs | Morocco | Vitamin A deficiency | 10.02245 | 14.93616 | 5.914409 |
| DALYs | Morocco | Dietary iron deficiency | 253.5971 | 388.3676 | 154.6306 |
| DALYs | Nauru | Iodine deficiency | 0.54218 | 1.077386 | 0.246026 |
| DALYs | Nauru | Vitamin A deficiency | 13.51372 | 21.77404 | 7.954315 |
| DALYs | Madagascar | Iodine deficiency | 36.34656 | 60.98231 | 20.25699 |
| DALYs | Madagascar | Vitamin A deficiency | 30.27006 | 44.43691 | 19.09821 |
| DALYs | Madagascar | Dietary iron deficiency | 498.5339 | 721.6824 | 321.2531 |
| DALYs | Mexico | Iodine deficiency | 10.02269 | 18.20864 | 4.948352 |
| DALYs | Mexico | Vitamin A deficiency | 9.454114 | 13.81091 | 6.3111 |
| DALYs | Mexico | Dietary iron deficiency | 118.1436 | 171.9284 | 78.51307 |
| DALYs | Cook Islands | Iodine deficiency | 0.492567 | 0.977662 | 0.208441 |
| DALYs | Central African Republic | Iodine deficiency | 95.65109 | 164.3884 | 52.88348 |
| DALYs | Central African Republic | Vitamin A deficiency | 72.16178 | 105.7635 | 46.89516 |
| DALYs | Central African Republic | Dietary iron deficiency | 524.2116 | 825.3128 | 313.9161 |
| DALYs | Botswana | Iodine deficiency | 3.139426 | 5.999142 | 1.434029 |
| DALYs | Botswana | Vitamin A deficiency | 19.65223 | 29.66572 | 12.56737 |
| DALYs | Botswana | Dietary iron deficiency | 313.8441 | 471.3962 | 198.3011 |
| DALYs | Mali | Iodine deficiency | 17.4943 | 27.83381 | 10.08685 |
| DALYs | Mali | Vitamin A deficiency | 73.08865 | 105.5315 | 46.87691 |
| DALYs | Mali | Dietary iron deficiency | 929.6706 | 1366.447 | 586.3037 |
| DALYs | Palestine | Iodine deficiency | 4.292466 | 8.160926 | 1.94079 |
| DALYs | Palestine | Vitamin A deficiency | 9.735992 | 14.47636 | 6.04156 |
| DALYs | Albania | Iodine deficiency | 2.004356 | 3.768094 | 0.927429 |
| DALYs | Albania | Vitamin A deficiency | 7.858287 | 13.21069 | 4.439443 |
| DALYs | Albania | Dietary iron deficiency | 194.7599 | 293.9723 | 123.702 |
| DALYs | Palestine | Dietary iron deficiency | 166.8726 | 251.2985 | 102.9902 |
| DALYs | Vanuatu | Dietary iron deficiency | 532.4556 | 781.1276 | 352.4342 |
| DALYs | Afghanistan | Iodine deficiency | 52.3446 | 76.05692 | 34.28009 |
| DALYs | Afghanistan | Vitamin A deficiency | 28.62644 | 42.32579 | 17.95768 |
| DALYs | Afghanistan | Dietary iron deficiency | 250.0609 | 374.0108 | 158.3107 |
| DALYs | Cambodia | Iodine deficiency | 14.39396 | 22.86491 | 8.685834 |
| DALYs | Cambodia | Vitamin A deficiency | 19.2558 | 30.49331 | 11.4165 |
| DALYs | Chad | Iodine deficiency | 22.45416 | 36.42818 | 12.58263 |
| DALYs | Vanuatu | Iodine deficiency | 12.49181 | 19.90851 | 7.648583 |
| DALYs | Vanuatu | Vitamin A deficiency | 28.30128 | 42.84333 | 17.2248 |
| DALYs | Bangladesh | Iodine deficiency | 39.41596 | 71.20161 | 20.95966 |
| DALYs | Bangladesh | Vitamin A deficiency | 9.448013 | 14.61821 | 5.794542 |
| DALYs | Bangladesh | Dietary iron deficiency | 488.6974 | 738.735 | 313.2281 |
| DALYs | Oman | Iodine deficiency | 3.852624 | 7.249333 | 1.750008 |
| DALYs | Oman | Vitamin A deficiency | 12.1387 | 18.04358 | 7.7123 |
| DALYs | Oman | Dietary iron deficiency | 226.2673 | 352.1442 | 130.6053 |
| DALYs | Chad | Vitamin A deficiency | 67.03216 | 97.15641 | 44.681 |
| DALYs | Chad | Dietary iron deficiency | 621.7812 | 925.188 | 401.405 |
| DALYs | Bosnia and Herzegovina | Iodine deficiency | 4.160186 | 7.96799 | 1.896784 |
| DALYs | Bosnia and Herzegovina | Vitamin A deficiency | 4.5852 | 7.921502 | 2.503452 |
| DALYs | Bosnia and Herzegovina | Dietary iron deficiency | 179.2469 | 282.3319 | 111.4755 |
| DALYs | Democratic Republic of the Congo | Iodine deficiency | 193.065 | 358.7216 | 100.6337 |
| DALYs | Democratic Republic of the Congo | Vitamin A deficiency | 55.71853 | 80.22938 | 36.28315 |
| DALYs | Malawi | Iodine deficiency | 42.2486 | 70.76622 | 23.49146 |
| DALYs | Malawi | Vitamin A deficiency | 41.04173 | 60.22699 | 25.83736 |
| DALYs | Malawi | Dietary iron deficiency | 614.732 | 894.4956 | 397.4261 |
| DALYs | Congo | Iodine deficiency | 119.2249 | 227.4957 | 55.10588 |
| DALYs | Congo | Vitamin A deficiency | 55.05836 | 81.2775 | 34.91673 |
| DALYs | Congo | Dietary iron deficiency | 493.3778 | 764.9675 | 297.7027 |
| DALYs | Germany | Iodine deficiency | 7.966132 | 15.74591 | 3.627833 |
| DALYs | Germany | Vitamin A deficiency | 0.094684 | 0.197602 | 0.040015 |
| DALYs | Germany | Dietary iron deficiency | 33.45763 | 56.10166 | 18.65878 |
| DALYs | Lesotho | Iodine deficiency | 50.31732 | 89.77662 | 27.18221 |
| DALYs | Lesotho | Vitamin A deficiency | 30.14069 | 45.05315 | 19.20949 |
| DALYs | Lesotho | Dietary iron deficiency | 388.7574 | 567.9961 | 252.5267 |
| DALYs | Benin | Iodine deficiency | 18.69493 | 30.07308 | 10.64899 |
| DALYs | Benin | Vitamin A deficiency | 54.2674 | 80.58592 | 34.87401 |
| DALYs | Benin | Dietary iron deficiency | 405.2834 | 593.1296 | 262.0627 |
| DALYs | Bahamas | Iodine deficiency | 3.050057 | 5.250719 | 1.498939 |
| DALYs | Bahamas | Vitamin A deficiency | 5.050606 | 7.610597 | 3.210801 |
| DALYs | Bahamas | Dietary iron deficiency | 311.8131 | 468.3743 | 194.2745 |
| DALYs | Cote d'Ivoire | Iodine deficiency | 17.28897 | 29.15072 | 9.740666 |
| DALYs | Cote d'Ivoire | Vitamin A deficiency | 38.70339 | 57.45675 | 24.4933 |
| DALYs | Cote d'Ivoire | Dietary iron deficiency | 567.2766 | 843.2711 | 367.8549 |
| DALYs | Austria | Iodine deficiency | 8.129488 | 16.23202 | 3.705679 |
| DALYs | Austria | Vitamin A deficiency | 0.214429 | 0.493711 | 0.076631 |
| DALYs | Austria | Dietary iron deficiency | 33.37608 | 57.62124 | 17.89059 |
| DALYs | Switzerland | Iodine deficiency | 5.700664 | 10.99266 | 2.615343 |
| DALYs | Switzerland | Vitamin A deficiency | 0.11192 | 0.24255 | 0.041003 |
| DALYs | Switzerland | Dietary iron deficiency | 30.00769 | 52.24083 | 15.90635 |
| DALYs | Democratic Republic of the Congo | Dietary iron deficiency | 500.2986 | 732.8162 | 317.6339 |
| DALYs | Italy | Iodine deficiency | 34.73049 | 68.39398 | 16.25187 |
| DALYs | Italy | Vitamin A deficiency | 0.368017 | 0.759399 | 0.148508 |
| DALYs | Italy | Dietary iron deficiency | 32.69606 | 54.52085 | 17.85424 |
| DALYs | Serbia | Iodine deficiency | 1.471691 | 2.804271 | 0.647719 |
| DALYs | Serbia | Vitamin A deficiency | 7.672416 | 13.18276 | 3.887277 |
| DALYs | Mauritania | Dietary iron deficiency | 553.9238 | 849.5322 | 302.9303 |
| DALYs | Sao Tome and Principe | Iodine deficiency | 15.4047 | 25.04648 | 8.537245 |
| DALYs | Belgium | Iodine deficiency | 7.907417 | 15.641 | 3.63032 |
| DALYs | Belgium | Vitamin A deficiency | 0.189777 | 0.410731 | 0.068261 |
| DALYs | Belgium | Dietary iron deficiency | 30.7053 | 53.37743 | 16.21467 |
| DALYs | Greece | Iodine deficiency | 8.463256 | 16.64944 | 3.907336 |
| DALYs | Greece | Vitamin A deficiency | 0.370116 | 0.809106 | 0.136169 |
| DALYs | Greece | Dietary iron deficiency | 42.06781 | 70.66482 | 22.84464 |
| DALYs | Niue | Iodine deficiency | 0.527847 | 1.04631 | 0.229071 |
| DALYs | Niue | Vitamin A deficiency | 7.704302 | 12.44205 | 4.439881 |
| DALYs | Niue | Dietary iron deficiency | 283.2704 | 431.6003 | 172.8916 |
| DALYs | Sao Tome and Principe | Vitamin A deficiency | 30.02685 | 48.49528 | 16.83616 |
| DALYs | Burkina Faso | Dietary iron deficiency | 753.4001 | 1138.152 | 470.6641 |
| DALYs | Jamaica | Iodine deficiency | 6.932643 | 11.38116 | 3.557772 |
| DALYs | Trinidad and Tobago | Iodine deficiency | 6.018311 | 10.48587 | 2.825095 |
| DALYs | Trinidad and Tobago | Vitamin A deficiency | 5.224658 | 7.934757 | 3.24933 |
| DALYs | Trinidad and Tobago | Dietary iron deficiency | 321.0296 | 494.6507 | 191.0647 |
| DALYs | Barbados | Dietary iron deficiency | 215.6907 | 343.11 | 128.9353 |
| DALYs | Armenia | Iodine deficiency | 10.95224 | 20.72309 | 5.022523 |
| DALYs | Armenia | Vitamin A deficiency | 0.32187 | 0.567922 | 0.16003 |
| DALYs | Armenia | Dietary iron deficiency | 248.3376 | 370.1648 | 155.3061 |
| DALYs | Northern Mariana Islands | Iodine deficiency | 0.486544 | 0.943009 | 0.210055 |
| DALYs | Northern Mariana Islands | Vitamin A deficiency | 4.086465 | 7.358656 | 2.129287 |
| DALYs | Northern Mariana Islands | Dietary iron deficiency | 249.0154 | 385.1413 | 147.0751 |
| DALYs | El Salvador | Iodine deficiency | 10.7009 | 17.97635 | 5.385025 |
| DALYs | El Salvador | Vitamin A deficiency | 7.762119 | 11.8642 | 4.774181 |
| DALYs | El Salvador | Dietary iron deficiency | 151.1713 | 237.0187 | 90.56672 |
| DALYs | Saint Lucia | Iodine deficiency | 2.770046 | 4.523538 | 1.487847 |
| DALYs | Saint Lucia | Vitamin A deficiency | 6.317713 | 9.665789 | 3.783006 |
| DALYs | Saint Lucia | Dietary iron deficiency | 331.5328 | 512.6095 | 207.0866 |
| DALYs | Guatemala | Iodine deficiency | 10.66755 | 17.57272 | 5.48832 |
| DALYs | Guatemala | Vitamin A deficiency | 9.264322 | 14.0067 | 5.840195 |
| DALYs | Guatemala | Dietary iron deficiency | 242.9697 | 365.5467 | 154.2335 |
| DALYs | Slovakia | Iodine deficiency | 1.857131 | 3.631473 | 0.836084 |
| DALYs | Slovakia | Vitamin A deficiency | 2.466177 | 4.401882 | 1.24076 |
| DALYs | Slovakia | Dietary iron deficiency | 126.1037 | 198.9997 | 75.64018 |
| DALYs | Fiji | Iodine deficiency | 0.551103 | 1.100086 | 0.246177 |
| DALYs | Fiji | Vitamin A deficiency | 12.78723 | 21.36108 | 7.598095 |
| DALYs | Fiji | Dietary iron deficiency | 503.8111 | 752.8833 | 321.6681 |
| DALYs | Honduras | Iodine deficiency | 13.74898 | 22.44287 | 7.12621 |
| DALYs | Honduras | Vitamin A deficiency | 7.021661 | 10.77733 | 4.377683 |
| DALYs | Honduras | Dietary iron deficiency | 223.4635 | 332.2408 | 140.0601 |
| DALYs | Saint Vincent and the Grenadines | Iodine deficiency | 5.469053 | 9.060599 | 2.822838 |
| DALYs | Saint Vincent and the Grenadines | Vitamin A deficiency | 7.551628 | 11.39286 | 4.707124 |
| DALYs | Saint Vincent and the Grenadines | Dietary iron deficiency | 367.5422 | 555.6747 | 227.6671 |
| DALYs | Iran (Islamic Republic of) | Iodine deficiency | 5.926476 | 9.605902 | 3.447633 |
| DALYs | Iran (Islamic Republic of) | Vitamin A deficiency | 0.757421 | 1.375214 | 0.400613 |
| DALYs | Iran (Islamic Republic of) | Dietary iron deficiency | 104.2582 | 163.5305 | 63.80557 |
| DALYs | Turkey | Iodine deficiency | 21.13723 | 33.64709 | 12.2129 |
| DALYs | Turkey | Vitamin A deficiency | 2.025736 | 3.511624 | 1.036429 |
| DALYs | Turkey | Dietary iron deficiency | 166.7839 | 267.1771 | 100.723 |
| DALYs | Cook Islands | Vitamin A deficiency | 6.194632 | 9.62486 | 3.644526 |
| DALYs | Cook Islands | Dietary iron deficiency | 248.164 | 390.6783 | 153.3212 |
| DALYs | Bhutan | Iodine deficiency | 5.9291 | 11.55984 | 2.688112 |
| DALYs | Bhutan | Vitamin A deficiency | 16.40089 | 24.35851 | 9.893962 |
| DALYs | Bhutan | Dietary iron deficiency | 1049.982 | 1533.852 | 699.6416 |
| DALYs | Kazakhstan | Iodine deficiency | 3.956921 | 7.688465 | 1.776001 |
| DALYs | Kazakhstan | Vitamin A deficiency | 6.778932 | 11.50688 | 3.805892 |
| DALYs | India | Iodine deficiency | 76.39944 | 130.7694 | 44.30467 |
| DALYs | India | Vitamin A deficiency | 22.89816 | 33.34481 | 14.85598 |
| DALYs | India | Dietary iron deficiency | 873.2865 | 1243.617 | 595.9169 |
| DALYs | Nepal | Iodine deficiency | 13.61579 | 26.59842 | 6.351408 |
| DALYs | Nepal | Vitamin A deficiency | 10.70202 | 16.5227 | 6.57163 |
| DALYs | Nepal | Dietary iron deficiency | 598.6067 | 895.6339 | 391.2413 |
| DALYs | Latvia | Iodine deficiency | 2.116798 | 3.495573 | 1.146674 |
| DALYs | Latvia | Vitamin A deficiency | 0.177956 | 0.332658 | 0.079031 |
| DALYs | Latvia | Dietary iron deficiency | 126.9491 | 196.6811 | 77.46355 |
| DALYs | Hungary | Iodine deficiency | 2.097332 | 4.014153 | 0.929779 |
| DALYs | Hungary | Vitamin A deficiency | 2.725093 | 4.982198 | 1.38023 |
| DALYs | Hungary | Dietary iron deficiency | 122.1218 | 198.9403 | 71.965 |
| DALYs | Angola | Vitamin A deficiency | 29.2126 | 42.73981 | 18.48256 |
| DALYs | Angola | Dietary iron deficiency | 358.1413 | 537.0098 | 219.9479 |
| DALYs | Uganda | Iodine deficiency | 18.61838 | 29.90991 | 10.86266 |
| DALYs | Uganda | Vitamin A deficiency | 18.75173 | 28.28298 | 12.05552 |
| DALYs | Uganda | Dietary iron deficiency | 362.1721 | 544.1805 | 233.8698 |
| DALYs | Djibouti | Iodine deficiency | 151.7721 | 284.4205 | 74.28407 |
| DALYs | Djibouti | Vitamin A deficiency | 23.02464 | 34.223 | 13.79313 |
| DALYs | Djibouti | Dietary iron deficiency | 520.5744 | 780.7816 | 326.9631 |
| DALYs | Nicaragua | Iodine deficiency | 13.41159 | 22.01408 | 6.965156 |
| DALYs | Nicaragua | Vitamin A deficiency | 3.768121 | 5.894893 | 2.29741 |
| DALYs | Nicaragua | Dietary iron deficiency | 93.08533 | 148.3145 | 54.58322 |
| DALYs | Iraq | Iodine deficiency | 23.65494 | 36.96583 | 13.56053 |
| DALYs | Iraq | Vitamin A deficiency | 7.322 | 11.05227 | 4.503332 |
| DALYs | Iraq | Dietary iron deficiency | 172.9195 | 273.0318 | 102.243 |
| DALYs | Kyrgyzstan | Iodine deficiency | 3.362652 | 6.411599 | 1.482024 |
| DALYs | Kyrgyzstan | Vitamin A deficiency | 9.268696 | 14.08506 | 5.851456 |
| DALYs | Kyrgyzstan | Dietary iron deficiency | 407.733 | 622.4932 | 263.4636 |
| DALYs | Bolivia (Plurinational State of) | Dietary iron deficiency | 406.5767 | 606.3252 | 256.3325 |
| DALYs | Myanmar | Iodine deficiency | 9.658278 | 15.58661 | 5.544819 |
| DALYs | Myanmar | Vitamin A deficiency | 14.38993 | 22.81592 | 8.676646 |
| DALYs | Myanmar | Dietary iron deficiency | 551.3812 | 831.4756 | 348.0456 |
| DALYs | Guinea-Bissau | Iodine deficiency | 50.23895 | 86.84585 | 27.75021 |
| DALYs | Guinea-Bissau | Vitamin A deficiency | 55.81117 | 82.64643 | 35.61523 |
| DALYs | Guinea-Bissau | Dietary iron deficiency | 639.5688 | 950.8077 | 416.0078 |
| DALYs | Panama | Iodine deficiency | 2.77254 | 5.19318 | 1.234989 |
| DALYs | Panama | Vitamin A deficiency | 5.021316 | 7.603052 | 3.120835 |
| DALYs | Panama | Dietary iron deficiency | 201.1003 | 302.7944 | 123.01 |
| DALYs | Zambia | Iodine deficiency | 19.17854 | 31.33621 | 10.62025 |
| DALYs | Zambia | Vitamin A deficiency | 31.6352 | 46.92697 | 20.59392 |
| DALYs | Zambia | Dietary iron deficiency | 979.6966 | 1408.828 | 657.7141 |
| DALYs | Burkina Faso | Iodine deficiency | 20.16678 | 32.6747 | 11.63091 |
| DALYs | Burkina Faso | Vitamin A deficiency | 66.61885 | 96.07792 | 42.10649 |
| DALYs | Eritrea | Iodine deficiency | 11.52755 | 18.36351 | 6.739253 |
| DALYs | Eritrea | Vitamin A deficiency | 31.7615 | 48.31446 | 19.59393 |
| DALYs | Eritrea | Dietary iron deficiency | 616.9938 | 912.3006 | 397.2361 |
| DALYs | Jordan | Iodine deficiency | 11.8002 | 18.59165 | 6.911512 |
| DALYs | Jordan | Vitamin A deficiency | 7.133077 | 10.70524 | 4.398972 |
| DALYs | Jordan | Dietary iron deficiency | 190.6872 | 287.7705 | 120.3206 |
| DALYs | Micronesia (Federated States of) | Iodine deficiency | 1.884625 | 2.966874 | 1.142909 |
| DALYs | Micronesia (Federated States of) | Vitamin A deficiency | 33.50453 | 50.51806 | 20.77559 |
| DALYs | Micronesia (Federated States of) | Dietary iron deficiency | 438.0104 | 643.1285 | 285.3782 |
| DALYs | North Macedonia | Iodine deficiency | 2.019609 | 3.859242 | 0.942026 |
| DALYs | North Macedonia | Vitamin A deficiency | 6.384239 | 10.28017 | 3.447658 |
| DALYs | North Macedonia | Dietary iron deficiency | 164.535 | 255.4817 | 100.8988 |
| DALYs | Mauritania | Iodine deficiency | 45.67012 | 80.75559 | 25.14132 |
| DALYs | Mauritania | Vitamin A deficiency | 26.11614 | 38.90119 | 16.20171 |
| DALYs | Iceland | Iodine deficiency | 3.26689 | 6.28453 | 1.520282 |
| DALYs | Iceland | Vitamin A deficiency | 0.148723 | 0.314226 | 0.052337 |
| DALYs | Iceland | Dietary iron deficiency | 27.03113 | 48.13998 | 14.0645 |
| DALYs | Slovenia | Iodine deficiency | 1.830521 | 3.399267 | 0.815541 |
| DALYs | Slovenia | Vitamin A deficiency | 1.584998 | 2.917601 | 0.758697 |
| DALYs | Slovenia | Dietary iron deficiency | 95.7499 | 155.513 | 57.09155 |
| DALYs | Mongolia | Iodine deficiency | 6.927828 | 11.29189 | 3.822593 |
| DALYs | Mongolia | Vitamin A deficiency | 6.036652 | 9.089716 | 3.74884 |
| DALYs | Mongolia | Dietary iron deficiency | 349.7442 | 515.7296 | 227.3682 |
| DALYs | Papua New Guinea | Iodine deficiency | 2.0576 | 3.416631 | 1.191301 |
| DALYs | Papua New Guinea | Vitamin A deficiency | 17.03134 | 25.73429 | 10.13453 |
| DALYs | Papua New Guinea | Dietary iron deficiency | 517.3913 | 745.2729 | 329.9897 |
| DALYs | American Samoa | Iodine deficiency | 0.453251 | 0.893207 | 0.192796 |
| DALYs | American Samoa | Vitamin A deficiency | 4.826575 | 8.463461 | 2.51491 |
| DALYs | American Samoa | Dietary iron deficiency | 320.3252 | 478.7899 | 207.6547 |
| DALYs | Ethiopia | Iodine deficiency | 127.0035 | 236.4075 | 66.52725 |
| DALYs | Ethiopia | Vitamin A deficiency | 39.82658 | 57.95619 | 26.27626 |
| DALYs | Ethiopia | Dietary iron deficiency | 396.0342 | 579.2279 | 261.7534 |
| DALYs | Montenegro | Dietary iron deficiency | 140.4215 | 217.8875 | 83.68753 |
| DALYs | Niger | Iodine deficiency | 49.36238 | 83.53921 | 26.93018 |
| DALYs | Niger | Vitamin A deficiency | 88.74985 | 128.321 | 57.9093 |
| DALYs | Niger | Dietary iron deficiency | 575.3129 | 856.4254 | 359.4583 |
| DALYs | Cameroon | Iodine deficiency | 14.24588 | 23.32504 | 7.955327 |
| DALYs | Cameroon | Vitamin A deficiency | 39.36711 | 60.73353 | 24.52883 |
| DALYs | Cameroon | Dietary iron deficiency | 386.7291 | 579.1306 | 244.4932 |
| DALYs | Ireland | Iodine deficiency | 7.949053 | 15.77728 | 3.639638 |
| DALYs | Ireland | Vitamin A deficiency | 0.133608 | 0.281458 | 0.050449 |
| DALYs | Ireland | Dietary iron deficiency | 29.84407 | 50.78499 | 16.16026 |
| DALYs | Samoa | Iodine deficiency | 0.43026 | 0.850263 | 0.189428 |
| DALYs | Samoa | Vitamin A deficiency | 19.2982 | 31.04972 | 11.68577 |
| DALYs | Samoa | Dietary iron deficiency | 288.6102 | 444.6009 | 180.2204 |
| DALYs | Bermuda | Iodine deficiency | 2.118263 | 3.706816 | 0.947966 |
| DALYs | Bermuda | Vitamin A deficiency | 0.594324 | 1.148475 | 0.263307 |
| DALYs | Bermuda | Dietary iron deficiency | 138.7316 | 219.4899 | 82.15058 |
| DALYs | Cabo Verde | Iodine deficiency | 17.34071 | 33.77341 | 8.072991 |
| DALYs | Cabo Verde | Vitamin A deficiency | 11.90753 | 18.41279 | 7.382502 |
| DALYs | Cabo Verde | Dietary iron deficiency | 429.9594 | 647.6994 | 265.3855 |
| DALYs | Nigeria | Iodine deficiency | 12.48249 | 24.31193 | 5.790492 |
| DALYs | Nigeria | Vitamin A deficiency | 17.46848 | 25.36787 | 11.6648 |
| DALYs | Nigeria | Dietary iron deficiency | 656.2497 | 966.498 | 435.3857 |
| DALYs | Suriname | Iodine deficiency | 4.853261 | 8.053334 | 2.514287 |
| DALYs | Suriname | Vitamin A deficiency | 9.335889 | 13.89036 | 5.814832 |
| DALYs | New Zealand | Iodine deficiency | 2.568497 | 4.908126 | 1.154995 |
| DALYs | New Zealand | Vitamin A deficiency | 0.272196 | 0.521928 | 0.125327 |
| DALYs | New Zealand | Dietary iron deficiency | 74.56349 | 123.649 | 41.1734 |
| DALYs | Israel | Iodine deficiency | 8.465978 | 16.74793 | 3.924695 |
| DALYs | Israel | Vitamin A deficiency | 2.207469 | 4.442215 | 0.971698 |
| DALYs | Israel | Dietary iron deficiency | 51.83934 | 88.05291 | 27.85285 |
| DALYs | Sao Tome and Principe | Dietary iron deficiency | 414.4924 | 619.8187 | 254.3426 |
| DALYs | United Kingdom | Iodine deficiency | 8.151771 | 16.04449 | 3.776776 |
| DALYs | United Kingdom | Vitamin A deficiency | 0.341407 | 0.632849 | 0.16898 |
| DALYs | United Kingdom | Dietary iron deficiency | 73.28843 | 115.3494 | 44.24114 |
| DALYs | United Republic of Tanzania | Iodine deficiency | 9.506704 | 15.17847 | 5.424967 |
| DALYs | United Republic of Tanzania | Vitamin A deficiency | 27.1449 | 39.72941 | 17.6061 |
| DALYs | United Republic of Tanzania | Dietary iron deficiency | 613.2512 | 902.1296 | 399.2885 |
| DALYs | Argentina | Iodine deficiency | 1.484305 | 2.792925 | 0.660519 |
| DALYs | Argentina | Vitamin A deficiency | 5.527977 | 9.353559 | 3.078714 |
| DALYs | Argentina | Dietary iron deficiency | 163.5437 | 253.3928 | 100.8165 |
| DALYs | Palau | Iodine deficiency | 0.504365 | 1.001752 | 0.217929 |
| DALYs | Palau | Vitamin A deficiency | 7.632457 | 12.03742 | 4.551182 |
| DALYs | Palau | Dietary iron deficiency | 269.405 | 409.495 | 167.3943 |
| DALYs | Puerto Rico | Iodine deficiency | 2.66449 | 4.573804 | 1.257864 |
| DALYs | Puerto Rico | Vitamin A deficiency | 0.6252 | 1.144607 | 0.308735 |
| DALYs | Puerto Rico | Dietary iron deficiency | 159.4112 | 257.2762 | 94.22718 |
| DALYs | Gabon | Iodine deficiency | 32.40381 | 58.33311 | 17.28635 |
| DALYs | Gabon | Vitamin A deficiency | 16.04972 | 23.71412 | 10.34173 |
| DALYs | Gabon | Dietary iron deficiency | 610.3701 | 917.3303 | 379.2777 |
| DALYs | Cyprus | Iodine deficiency | 8.523052 | 17.01015 | 3.951412 |
| DALYs | Algeria | Dietary iron deficiency | 186.5125 | 299.0817 | 113.3912 |
| DALYs | Lithuania | Iodine deficiency | 1.990221 | 3.320503 | 1.06769 |
| DALYs | Lithuania | Vitamin A deficiency | 0.164899 | 0.310136 | 0.070382 |
| DALYs | Lithuania | Dietary iron deficiency | 133.7447 | 209.2346 | 80.44124 |
| DALYs | Namibia | Iodine deficiency | 10.13252 | 16.80537 | 5.429174 |
| DALYs | Namibia | Vitamin A deficiency | 14.77412 | 22.16798 | 9.15972 |
| DALYs | Republic of Moldova | Iodine deficiency | 1.935214 | 3.212187 | 1.063434 |
| DALYs | Republic of Moldova | Vitamin A deficiency | 0.726325 | 1.304723 | 0.359945 |
| DALYs | Republic of Moldova | Dietary iron deficiency | 221.1879 | 335.9148 | 139.8702 |
| DALYs | South Africa | Iodine deficiency | 6.252891 | 11.95528 | 2.907348 |
| DALYs | South Africa | Vitamin A deficiency | 9.706491 | 14.16827 | 6.373217 |
| DALYs | South Africa | Dietary iron deficiency | 252.6991 | 365.734 | 166.9453 |
| DALYs | Eswatini | Iodine deficiency | 9.87774 | 19.35595 | 4.583017 |
| DALYs | Eswatini | Vitamin A deficiency | 17.0311 | 25.25162 | 11.02886 |
| DALYs | Eswatini | Dietary iron deficiency | 285.4957 | 427.431 | 180.384 |
| DALYs | Zimbabwe | Dietary iron deficiency | 390.7529 | 573.2126 | 250.7111 |
| DALYs | Andorra | Iodine deficiency | 7.791656 | 15.38468 | 3.564506 |
| DALYs | Andorra | Vitamin A deficiency | 0.142459 | 0.326801 | 0.054094 |
| DALYs | Andorra | Dietary iron deficiency | 32.2645 | 58.62046 | 16.979 |
| DALYs | Barbados | Iodine deficiency | 8.1291 | 13.83035 | 3.889226 |
| DALYs | Barbados | Vitamin A deficiency | 3.885664 | 5.903192 | 2.41905 |
| DALYs | Belize | Iodine deficiency | 2.199918 | 3.656824 | 1.191097 |
| DALYs | Belize | Vitamin A deficiency | 8.276876 | 12.62749 | 5.106154 |
| DALYs | Belize | Dietary iron deficiency | 379.7227 | 566.4789 | 234.6939 |
| DALYs | Kazakhstan | Dietary iron deficiency | 376.6783 | 563.4856 | 237.0704 |
| DALYs | Saudi Arabia | Iodine deficiency | 1.485721 | 2.781926 | 0.656892 |
| DALYs | Saudi Arabia | Vitamin A deficiency | 0.136284 | 0.267182 | 0.057422 |
| DALYs | Saudi Arabia | Dietary iron deficiency | 74.92241 | 120.5141 | 43.17149 |
| DALYs | Indonesia | Iodine deficiency | 3.886927 | 7.222664 | 1.936575 |
| DALYs | Indonesia | Vitamin A deficiency | 17.59542 | 25.68215 | 11.23823 |
| DALYs | Indonesia | Dietary iron deficiency | 299.5254 | 444.663 | 192.793 |
| DALYs | Lao People's Democratic Republic | Iodine deficiency | 8.112619 | 13.22518 | 4.792668 |
| DALYs | Lao People's Democratic Republic | Vitamin A deficiency | 15.44308 | 24.48511 | 9.10412 |
| DALYs | Lao People's Democratic Republic | Dietary iron deficiency | 373.2586 | 558.3818 | 236.6064 |
| DALYs | Suriname | Dietary iron deficiency | 366.3231 | 552.274 | 227.8964 |
| DALYs | Canada | Iodine deficiency | 2.335072 | 4.455811 | 1.067761 |
| DALYs | Canada | Vitamin A deficiency | 0.187768 | 0.379413 | 0.071672 |
| DALYs | Canada | Dietary iron deficiency | 37.86024 | 61.31772 | 21.87009 |
| DALYs | Japan | Dietary iron deficiency | 121.9495 | 188.1824 | 73.95119 |
| DALYs | United States of America | Iodine deficiency | 2.43569 | 4.645379 | 1.107089 |
| DALYs | United States of America | Vitamin A deficiency | 0.145443 | 0.264526 | 0.069394 |
| DALYs | United States of America | Dietary iron deficiency | 65.43268 | 103.3082 | 40.2839 |
| DALYs | Republic of Korea | Iodine deficiency | 2.5334 | 4.957026 | 1.143308 |
| DALYs | Republic of Korea | Vitamin A deficiency | 0.331785 | 0.599075 | 0.156369 |
| DALYs | Republic of Korea | Dietary iron deficiency | 147.0975 | 236.1717 | 90.55164 |
| DALYs | Algeria | Iodine deficiency | 12.50126 | 19.73224 | 7.26173 |
| DALYs | Algeria | Vitamin A deficiency | 7.335871 | 11.20639 | 4.571091 |
| DALYs | Bahrain | Iodine deficiency | 3.822572 | 7.216192 | 1.745 |
| DALYs | Bahrain | Vitamin A deficiency | 1.20127 | 2.116833 | 0.601313 |
| DALYs | Bahrain | Dietary iron deficiency | 152.5364 | 248.9573 | 92.67629 |
| DALYs | South Sudan | Iodine deficiency | 24.48168 | 42.7136 | 13.20651 |
| DALYs | South Sudan | Vitamin A deficiency | 33.13307 | 48.45974 | 21.44258 |
| DALYs | South Sudan | Dietary iron deficiency | 594.6572 | 890.3488 | 373.4267 |
| DALYs | Mozambique | Iodine deficiency | 22.2338 | 35.23748 | 12.8248 |
| DALYs | Rwanda | Iodine deficiency | 32.29304 | 63.00451 | 14.93952 |
| DALYs | Rwanda | Vitamin A deficiency | 16.42944 | 24.92675 | 10.11928 |
| DALYs | Rwanda | Dietary iron deficiency | 330.0868 | 487.8625 | 208.023 |
| DALYs | Mozambique | Vitamin A deficiency | 47.03097 | 70.03564 | 30.01688 |
| DALYs | Mozambique | Dietary iron deficiency | 564.5302 | 831.0527 | 362.9939 |
| DALYs | Cambodia | Dietary iron deficiency | 468.0985 | 684.7027 | 303.4405 |
| DALYs | Syrian Arab Republic | Dietary iron deficiency | 201.9485 | 316.4278 | 124.2096 |
| DALYs | Burundi | Iodine deficiency | 44.9804 | 74.82732 | 25.30271 |
| DALYs | Burundi | Vitamin A deficiency | 30.26912 | 45.9278 | 19.00585 |
| DALYs | Burundi | Dietary iron deficiency | 390.8342 | 575.436 | 254.8494 |
| DALYs | Denmark | Iodine deficiency | 5.602687 | 10.986 | 2.591429 |
| DALYs | Denmark | Vitamin A deficiency | 0.181499 | 0.401937 | 0.065827 |
| DALYs | Denmark | Dietary iron deficiency | 35.59752 | 63.53828 | 18.70303 |
| DALYs | Comoros | Iodine deficiency | 8.816833 | 14.2901 | 5.16188 |
| DALYs | Comoros | Vitamin A deficiency | 26.72769 | 40.84452 | 16.45653 |
| DALYs | Comoros | Dietary iron deficiency | 609.521 | 909.0759 | 379.4667 |
| DALYs | Finland | Iodine deficiency | 8.107372 | 15.95058 | 3.721414 |
| DALYs | Finland | Vitamin A deficiency | 0.198234 | 0.471233 | 0.064239 |
| DALYs | Finland | Dietary iron deficiency | 35.00419 | 60.00527 | 18.53007 |
| DALYs | France | Dietary iron deficiency | 25.54528 | 44.88521 | 13.44404 |
| Incidence | Slovakia | Iodine deficiency | 10.1226 | 12.84069 | 7.632375 |
| Incidence | Slovakia | Vitamin A deficiency | 6072.27 | 7025.261 | 5178.874 |
| Incidence | United States of America | Iodine deficiency | 13.60569 | 17.01169 | 10.66894 |
| Incidence | United States of America | Vitamin A deficiency | 440.0833 | 541.2735 | 357.0883 |
| Incidence | United States of America | Dietary iron deficiency | 0 | 0 | 0 |
| Incidence | China | Dietary iron deficiency | 0 | 0 | 0 |
| Incidence | Denmark | Iodine deficiency | 27.27532 | 34.89581 | 21.20159 |
| Incidence | Denmark | Vitamin A deficiency | 439.5851 | 533.7205 | 359.8166 |
| Incidence | Puerto Rico | Iodine deficiency | 7.906166 | 10.26737 | 6.018397 |
| Incidence | Puerto Rico | Vitamin A deficiency | 1204.482 | 1419.383 | 1024.807 |
| Incidence | American Samoa | Iodine deficiency | 2.787999 | 3.685767 | 2.02108 |
| Incidence | American Samoa | Vitamin A deficiency | 6562.33 | 8099.877 | 5256.222 |
| Incidence | Portugal | Iodine deficiency | 53.75911 | 68.77051 | 41.68703 |
| Incidence | Niger | Iodine deficiency | 127.7654 | 162.4305 | 99.6249 |
| Incidence | Niger | Vitamin A deficiency | 43501.51 | 48083.42 | 38967.65 |
| Incidence | Guatemala | Iodine deficiency | 26.2042 | 33.70626 | 19.7327 |
| Incidence | Guatemala | Vitamin A deficiency | 5880.068 | 7117.035 | 4913.624 |
| Incidence | Saint Kitts and Nevis | Iodine deficiency | 9.490133 | 12.14053 | 7.309665 |
| Incidence | Saint Kitts and Nevis | Vitamin A deficiency | 1914.566 | 2266.927 | 1601.517 |
| Incidence | Albania | Iodine deficiency | 10.77058 | 13.75628 | 8.142288 |
| Incidence | Albania | Vitamin A deficiency | 11731.87 | 13478.61 | 10138.25 |
| Incidence | Bermuda | Iodine deficiency | 6.621168 | 8.547153 | 5.019866 |
| Incidence | Bermuda | Vitamin A deficiency | 1210.927 | 1416.254 | 1037.319 |
| Incidence | Finland | Iodine deficiency | 37.15302 | 47.35389 | 28.58134 |
| Incidence | Nigeria | Iodine deficiency | 57.02176 | 71.76463 | 44.88146 |
| Incidence | Nigeria | Vitamin A deficiency | 5128.399 | 6114.312 | 4206.235 |
| Incidence | Nigeria | Dietary iron deficiency | 0 | 0 | 0 |
| Incidence | Suriname | Vitamin A deficiency | 4317.255 | 5090.376 | 3567.129 |
| Incidence | Mexico | Iodine deficiency | 39.15966 | 49.3665 | 30.02929 |
| Incidence | Cook Islands | Iodine deficiency | 3.006433 | 3.945742 | 2.181383 |
| Incidence | Cook Islands | Vitamin A deficiency | 3858.086 | 4773.769 | 3068.388 |
| Incidence | Honduras | Iodine deficiency | 32.11541 | 41.1339 | 24.31289 |
| Incidence | Honduras | Vitamin A deficiency | 5901.577 | 7014.21 | 4946.865 |
| Incidence | Iran (Islamic Republic of) | Iodine deficiency | 16.57817 | 20.61219 | 13.07251 |
| Incidence | Iran (Islamic Republic of) | Vitamin A deficiency | 1344.704 | 1651.792 | 1118.861 |
| Incidence | Iran (Islamic Republic of) | Dietary iron deficiency | 0 | 0 | 0 |
| Incidence | Tunisia | Iodine deficiency | 18.45428 | 23.06074 | 14.42568 |
| Incidence | Tunisia | Vitamin A deficiency | 1754.96 | 2071.49 | 1468.023 |
| Incidence | Ethiopia | Iodine deficiency | 404.4757 | 510.8509 | 316.9253 |
| Incidence | Ethiopia | Vitamin A deficiency | 24329.13 | 28388.42 | 20909.75 |
| Incidence | Ethiopia | Dietary iron deficiency | 0 | 0 | 0 |
| Incidence | Chad | Vitamin A deficiency | 34260.53 | 38895.56 | 29897.54 |
| Incidence | Nicaragua | Iodine deficiency | 31.49382 | 40.1042 | 23.68544 |
| Incidence | Czechia | Iodine deficiency | 6.509806 | 8.294983 | 4.890628 |
| Incidence | Czechia | Vitamin A deficiency | 5505.387 | 6331.709 | 4722.655 |
| Incidence | Kenya | Iodine deficiency | 58.36389 | 73.58923 | 45.83146 |
| Incidence | Kenya | Vitamin A deficiency | 29712.62 | 33973.7 | 26085.15 |
| Incidence | Latvia | Iodine deficiency | 5.420656 | 6.99874 | 4.021157 |
| Incidence | Latvia | Vitamin A deficiency | 757.1049 | 891.0874 | 642.8161 |
| Incidence | New Zealand | Dietary iron deficiency | 0 | 0 | 0 |
| Incidence | Bahamas | Iodine deficiency | 8.705342 | 11.1582 | 6.65557 |
| Incidence | Bahamas | Vitamin A deficiency | 1434.223 | 1682.365 | 1203.767 |
| Incidence | Lao People's Democratic Republic | Iodine deficiency | 20.28492 | 26.30913 | 15.52855 |
| Incidence | Lao People's Democratic Republic | Vitamin A deficiency | 11596.34 | 13603.04 | 9592.967 |
| Incidence | Tuvalu | Iodine deficiency | 3.859175 | 5.073454 | 2.875104 |
| Incidence | Tuvalu | Vitamin A deficiency | 14979.88 | 17994.06 | 12572.78 |
| Incidence | Cote d'Ivoire | Iodine deficiency | 49.77999 | 63.0679 | 39.18246 |
| Incidence | Cote d'Ivoire | Vitamin A deficiency | 14240.01 | 17152.56 | 11642.12 |
| Incidence | Jordan | Iodine deficiency | 28.6781 | 35.55357 | 22.43454 |
| Incidence | Jordan | Vitamin A deficiency | 5045.489 | 6131.246 | 4187.364 |
| Incidence | Hungary | Iodine deficiency | 11.24774 | 14.41213 | 8.476292 |
| Incidence | Hungary | Vitamin A deficiency | 6651.651 | 7631.892 | 5727.188 |
| Incidence | Kazakhstan | Iodine deficiency | 18.81274 | 24.03924 | 14.34022 |
| Incidence | Kazakhstan | Vitamin A deficiency | 7611.972 | 9212.276 | 6200.297 |
| Incidence | Turkey | Iodine deficiency | 49.81958 | 60.86121 | 39.14707 |
| Incidence | Namibia | Iodine deficiency | 30.27766 | 38.21875 | 23.92023 |
| Incidence | Namibia | Vitamin A deficiency | 6569.442 | 7995.872 | 5370.48 |
| Incidence | Kenya | Dietary iron deficiency | 0 | 0 | 0 |
| Incidence | South Sudan | Iodine deficiency | 77.06001 | 97.34763 | 60.85582 |
| Incidence | South Sudan | Vitamin A deficiency | 20173.61 | 23709.22 | 17093.99 |
| Incidence | Lithuania | Iodine deficiency | 5.149511 | 6.731633 | 3.768315 |
| Incidence | Lithuania | Vitamin A deficiency | 681.8058 | 800.9652 | 582.9476 |
| Incidence | Norway | Vitamin A deficiency | 399.4748 | 499.2764 | 324.3725 |
| Incidence | Norway | Dietary iron deficiency | 0 | 0 | 0 |
| Incidence | Ghana | Iodine deficiency | 92.46522 | 117.4514 | 71.99873 |
| Incidence | Ghana | Vitamin A deficiency | 15915.73 | 18795.72 | 13097.57 |
| Incidence | Marshall Islands | Iodine deficiency | 3.894129 | 5.14016 | 2.865524 |
| Incidence | Turkey | Vitamin A deficiency | 3184.302 | 3724.763 | 2713.447 |
| Incidence | Barbados | Iodine deficiency | 22.00686 | 28.25628 | 17.0255 |
| Incidence | Barbados | Vitamin A deficiency | 2031.763 | 2371.303 | 1719.501 |
| Incidence | New Zealand | Iodine deficiency | 14.19868 | 17.76954 | 11.13756 |
| Incidence | New Zealand | Vitamin A deficiency | 541.3629 | 650.7298 | 444.7329 |
| Incidence | Marshall Islands | Vitamin A deficiency | 22663.67 | 26326.44 | 19353.74 |
| Incidence | Togo | Iodine deficiency | 91.83026 | 116.1979 | 72.42537 |
| Incidence | Togo | Vitamin A deficiency | 18885.65 | 22054.03 | 15809.46 |
| Incidence | Malaysia | Iodine deficiency | 40.88704 | 52.95292 | 30.92343 |
| Incidence | Malaysia | Vitamin A deficiency | 546.7082 | 669.5191 | 445.2848 |
| Incidence | Ireland | Iodine deficiency | 36.47864 | 46.93928 | 28.23392 |
| Incidence | Ireland | Vitamin A deficiency | 479.6504 | 580.4343 | 383.6712 |
| Incidence | Kyrgyzstan | Iodine deficiency | 16.49378 | 21.38378 | 12.23891 |
| Incidence | Kyrgyzstan | Vitamin A deficiency | 4423.488 | 5355.656 | 3637.697 |
| Incidence | Benin | Iodine deficiency | 48.03207 | 60.41276 | 37.82765 |
| Incidence | Benin | Vitamin A deficiency | 27262.28 | 31358.09 | 23522.25 |
| Incidence | Nicaragua | Vitamin A deficiency | 1750.252 | 2099.561 | 1455.03 |
| Incidence | Italy | Iodine deficiency | 136.1818 | 173.7347 | 104.5297 |
| Incidence | Italy | Vitamin A deficiency | 1415.512 | 1660.52 | 1206.425 |
| Incidence | Italy | Dietary iron deficiency | 0 | 0 | 0 |
| Incidence | Guinea | Iodine deficiency | 113.3904 | 143.331 | 88.47026 |
| Incidence | Guinea | Vitamin A deficiency | 24602.8 | 28472.22 | 20937.38 |
| Incidence | Kuwait | Iodine deficiency | 20.58865 | 25.73828 | 16.11953 |
| Incidence | Kuwait | Vitamin A deficiency | 745.6719 | 912.1799 | 619.932 |
| Incidence | Micronesia (Federated States of) | Iodine deficiency | 3.993699 | 5.231525 | 2.961119 |
| Incidence | Micronesia (Federated States of) | Vitamin A deficiency | 34768.53 | 38842.13 | 30793.63 |
| Incidence | United States Virgin Islands | Iodine deficiency | 7.053564 | 9.181846 | 5.382418 |
| Incidence | United States Virgin Islands | Vitamin A deficiency | 1070.232 | 1256.163 | 903.6089 |
| Incidence | Monaco | Iodine deficiency | 34.03357 | 43.85714 | 26.31473 |
| Incidence | Monaco | Vitamin A deficiency | 301.5993 | 367.065 | 249.0219 |
| Incidence | Israel | Iodine deficiency | 38.32463 | 49.13025 | 29.89063 |
| Incidence | Israel | Vitamin A deficiency | 4414.096 | 5554.151 | 3439.742 |
| Incidence | Greenland | Iodine deficiency | 12.64212 | 16.02192 | 9.78289 |
| Incidence | Greenland | Vitamin A deficiency | 977.1553 | 1206.444 | 797.9537 |
| Incidence | Belize | Iodine deficiency | 5.965002 | 7.712766 | 4.494018 |
| Incidence | Belize | Vitamin A deficiency | 4673.406 | 5506.833 | 3900.079 |
| Incidence | Maldives | Iodine deficiency | 12.23219 | 15.7395 | 9.476732 |
| Incidence | Maldives | Vitamin A deficiency | 2948.436 | 3559.24 | 2340.179 |
| Incidence | Nauru | Iodine deficiency | 3.273434 | 4.300298 | 2.385225 |
| Incidence | Nauru | Vitamin A deficiency | 11349.06 | 13719.79 | 9161.41 |
| Incidence | Mongolia | Iodine deficiency | 16.75601 | 21.51162 | 12.55048 |
| Incidence | Niue | Iodine deficiency | 3.19446 | 4.187229 | 2.335306 |
| Incidence | Niue | Vitamin A deficiency | 5712.037 | 7092.964 | 4530.453 |
| Incidence | Papua New Guinea | Iodine deficiency | 3.9332 | 5.166246 | 2.917747 |
| Incidence | Mauritius | Iodine deficiency | 19.75645 | 25.31776 | 15.23214 |
| Incidence | Mauritius | Vitamin A deficiency | 1311.458 | 1609.531 | 1059.559 |
| Incidence | Eswatini | Iodine deficiency | 44.8225 | 56.97243 | 35.3616 |
| Incidence | Eswatini | Vitamin A deficiency | 8688.369 | 10730 | 7026.227 |
| Incidence | Lebanon | Iodine deficiency | 59.92442 | 73.64539 | 47.75525 |
| Incidence | Lebanon | Vitamin A deficiency | 1682.653 | 2043.834 | 1399.464 |
| Incidence | Austria | Iodine deficiency | 37.04217 | 47.47321 | 28.95127 |
| Incidence | Austria | Vitamin A deficiency | 565.0928 | 691.1856 | 470.3827 |
| Incidence | Sweden | Iodine deficiency | 40.35637 | 51.94176 | 31.34671 |
| Incidence | Sweden | Vitamin A deficiency | 507.8366 | 622.633 | 415.4368 |
| Incidence | Sweden | Dietary iron deficiency | 0 | 0 | 0 |
| Incidence | Malta | Iodine deficiency | 38.50813 | 49.30896 | 29.73693 |
| Incidence | Malta | Vitamin A deficiency | 759.6067 | 942.0992 | 627.821 |
| Incidence | Luxembourg | Iodine deficiency | 35.68991 | 44.96217 | 27.63138 |
| Incidence | Luxembourg | Vitamin A deficiency | 408.1687 | 494.7124 | 331.0745 |
| Incidence | Burkina Faso | Iodine deficiency | 49.66116 | 63.08548 | 39.03192 |
| Incidence | Belgium | Iodine deficiency | 36.31538 | 46.70646 | 28.24817 |
| Incidence | Belgium | Vitamin A deficiency | 536.0939 | 656.646 | 435.8247 |
| Incidence | Northern Mariana Islands | Iodine deficiency | 2.967232 | 3.922531 | 2.171263 |
| Incidence | Northern Mariana Islands | Vitamin A deficiency | 4083.038 | 5089.132 | 3267.317 |
| Incidence | Poland | Iodine deficiency | 10.61862 | 13.31434 | 8.12663 |
| Incidence | Poland | Vitamin A deficiency | 5933.509 | 6864.496 | 5066.371 |
| Incidence | Poland | Dietary iron deficiency | 0 | 0 | 0 |
| Incidence | Mozambique | Iodine deficiency | 51.84738 | 65.84599 | 40.72683 |
| Incidence | Mozambique | Vitamin A deficiency | 28865.43 | 33338.42 | 24964.81 |
| Incidence | Zimbabwe | Iodine deficiency | 120.8764 | 153.1308 | 94.55048 |
| Incidence | Netherlands | Iodine deficiency | 35.59807 | 45.4905 | 27.3296 |
| Incidence | Netherlands | Vitamin A deficiency | 481.1534 | 594.3412 | 395.0142 |
| Incidence | Cyprus | Iodine deficiency | 38.39861 | 48.98961 | 29.67962 |
| Incidence | Cyprus | Vitamin A deficiency | 645.6531 | 791.7477 | 524.8659 |
| Incidence | Rwanda | Iodine deficiency | 121.9076 | 153.6725 | 94.81554 |
| Incidence | Rwanda | Vitamin A deficiency | 14592.39 | 17073.35 | 12290.56 |
| Incidence | Dominican Republic | Iodine deficiency | 18.20534 | 23.29304 | 13.98998 |
| Incidence | Dominican Republic | Vitamin A deficiency | 4835.136 | 5695.192 | 4044.739 |
| Incidence | Burkina Faso | Vitamin A deficiency | 27594.06 | 31725.92 | 23930.23 |
| Incidence | Russian Federation | Iodine deficiency | 9.054415 | 11.58407 | 6.757335 |
| Incidence | Russian Federation | Vitamin A deficiency | 199.5045 | 233.0428 | 169.9333 |
| Incidence | Russian Federation | Dietary iron deficiency | 0 | 0 | 0 |
| Incidence | Costa Rica | Iodine deficiency | 22.91563 | 29.21912 | 17.35411 |
| Incidence | Costa Rica | Vitamin A deficiency | 2864.118 | 3417.047 | 2411.396 |
| Incidence | Serbia | Iodine deficiency | 8.265194 | 10.58262 | 6.17171 |
| Incidence | Serbia | Vitamin A deficiency | 15871.77 | 17944.52 | 13945.39 |
| Incidence | Ukraine | Iodine deficiency | 27.39894 | 35.71611 | 20.80376 |
| Incidence | Ukraine | Vitamin A deficiency | 1357.395 | 1590.471 | 1158.006 |
| Incidence | Ukraine | Dietary iron deficiency | 0 | 0 | 0 |
| Incidence | India | Vitamin A deficiency | 8129.879 | 9719.208 | 6844.022 |
| Incidence | India | Dietary iron deficiency | 0 | 0 | 0 |
| Incidence | Saint Lucia | Iodine deficiency | 7.472398 | 9.652804 | 5.673628 |
| Incidence | Saint Lucia | Vitamin A deficiency | 3007.208 | 3490.052 | 2581.265 |
| Incidence | El Salvador | Iodine deficiency | 26.75538 | 34.21965 | 20.11798 |
| Incidence | El Salvador | Vitamin A deficiency | 5828.645 | 6986.558 | 4813.197 |
| Incidence | Saint Vincent and the Grenadines | Iodine deficiency | 14.1698 | 17.99883 | 10.91035 |
| Incidence | Saint Vincent and the Grenadines | Vitamin A deficiency | 3982.819 | 4724.69 | 3363.278 |
| Incidence | Pakistan | Iodine deficiency | 248.0408 | 310.5305 | 196.2426 |
| Incidence | Pakistan | Vitamin A deficiency | 3521.685 | 4256.642 | 2883.567 |
| Incidence | Pakistan | Dietary iron deficiency | 0 | 0 | 0 |
| Incidence | Suriname | Iodine deficiency | 12.78926 | 16.33225 | 9.821376 |
| Incidence | Democratic People's Republic of Korea | Iodine deficiency | 3.902989 | 5.057722 | 2.920992 |
| Incidence | Democratic People's Republic of Korea | Vitamin A deficiency | 7105.379 | 8577.585 | 5858.878 |
| Incidence | Panama | Iodine deficiency | 14.52497 | 18.4463 | 11.0501 |
| Incidence | Panama | Vitamin A deficiency | 2811.884 | 3337.038 | 2339.63 |
| Incidence | Congo | Iodine deficiency | 396.0355 | 482.1588 | 317.6548 |
| Incidence | Congo | Vitamin A deficiency | 27043.51 | 30947.28 | 23493.53 |
| Incidence | Brunei Darussalam | Iodine deficiency | 14.05725 | 17.67876 | 11.00365 |
| Incidence | Brunei Darussalam | Vitamin A deficiency | 1112.336 | 1358.553 | 898.5916 |
| Incidence | Democratic Republic of the Congo | Iodine deficiency | 549.2017 | 665.6769 | 443.4121 |
| Incidence | Democratic Republic of the Congo | Vitamin A deficiency | 30146.05 | 34484.43 | 26304.29 |
| Incidence | Equatorial Guinea | Iodine deficiency | 208.175 | 263.7419 | 164.5973 |
| Incidence | Equatorial Guinea | Vitamin A deficiency | 4574.185 | 5768.954 | 3671.029 |
| Incidence | Brazil | Iodine deficiency | 6.329765 | 8.137042 | 4.797415 |
| Incidence | Brazil | Vitamin A deficiency | 10128.64 | 11793.54 | 8668.303 |
| Incidence | Brazil | Dietary iron deficiency | 0 | 0 | 0 |
| Incidence | Somalia | Iodine deficiency | 725.1491 | 869.5342 | 591.2675 |
| Incidence | Somalia | Vitamin A deficiency | 63640.11 | 67657.56 | 59279.85 |
| Incidence | Trinidad and Tobago | Iodine deficiency | 17.92247 | 23.01327 | 13.84661 |
| Incidence | Trinidad and Tobago | Vitamin A deficiency | 1982.991 | 2315.83 | 1691.944 |
| Incidence | Sudan | Iodine deficiency | 60.8474 | 75.162 | 48.18113 |
| Incidence | Sudan | Vitamin A deficiency | 9067.546 | 11071.48 | 7431.238 |
| Incidence | Finland | Vitamin A deficiency | 527.5514 | 645.2803 | 434.1381 |
| Incidence | Venezuela (Bolivarian Republic of) | Iodine deficiency | 24.62041 | 30.88257 | 18.47642 |
| Incidence | Venezuela (Bolivarian Republic of) | Vitamin A deficiency | 5461.028 | 6475.999 | 4549.232 |
| Incidence | Norway | Iodine deficiency | 38.58343 | 49.06186 | 30.20259 |
| Incidence | Gambia | Iodine deficiency | 141.6182 | 178.7382 | 111.5282 |
| Incidence | Gambia | Vitamin A deficiency | 22716.8 | 26722.24 | 19313.06 |
| Incidence | France | Iodine deficiency | 37.31723 | 47.68099 | 28.8011 |
| Incidence | France | Vitamin A deficiency | 172.5094 | 210.3543 | 141.6564 |
| Incidence | Indonesia | Iodine deficiency | 19.14641 | 24.61518 | 14.72487 |
| Incidence | Indonesia | Vitamin A deficiency | 5770.57 | 6863.662 | 4762.1 |
| Incidence | Indonesia | Dietary iron deficiency | 0 | 0 | 0 |
| Incidence | Bolivia (Plurinational State of) | Iodine deficiency | 5.23221 | 6.734821 | 3.947772 |
| Incidence | Bolivia (Plurinational State of) | Vitamin A deficiency | 8475.208 | 10156.55 | 6977.247 |
| Incidence | Sri Lanka | Iodine deficiency | 43.8179 | 58.09139 | 32.90977 |
| Incidence | Sri Lanka | Vitamin A deficiency | 3499.452 | 4281.622 | 2892.138 |
| Incidence | Germany | Iodine deficiency | 36.45687 | 46.48625 | 28.12041 |
| Incidence | Germany | Vitamin A deficiency | 334.9781 | 399.4373 | 282.3568 |
| Incidence | Republic of Korea | Iodine deficiency | 13.87844 | 17.49109 | 10.73318 |
| Incidence | Republic of Korea | Vitamin A deficiency | 620.7092 | 750.3216 | 521.6364 |
| Incidence | Portugal | Vitamin A deficiency | 1015.76 | 1219.854 | 829.0114 |
| Incidence | Mongolia | Vitamin A deficiency | 1242.084 | 1511.345 | 1020.407 |
| Incidence | Spain | Iodine deficiency | 37.37633 | 47.92811 | 29.02321 |
| Incidence | Spain | Vitamin A deficiency | 667.8607 | 800.2035 | 566.1579 |
| Incidence | Tajikistan | Iodine deficiency | 28.49264 | 36.82285 | 21.40302 |
| Incidence | Tajikistan | Vitamin A deficiency | 8084.766 | 9556.331 | 6676.167 |
| Incidence | Turkmenistan | Iodine deficiency | 15.4828 | 19.81945 | 11.72417 |
| Incidence | Turkmenistan | Vitamin A deficiency | 2273.884 | 2740.357 | 1881.51 |
| Incidence | Republic of Moldova | Iodine deficiency | 4.97962 | 6.501248 | 3.626112 |
| Incidence | Republic of Moldova | Vitamin A deficiency | 1857.994 | 2177.424 | 1571.52 |
| Incidence | Palau | Iodine deficiency | 3.078264 | 4.069416 | 2.196436 |
| Incidence | Palau | Vitamin A deficiency | 5160.581 | 6343.552 | 4154.474 |
| Incidence | Guinea-Bissau | Iodine deficiency | 141.7765 | 178.3473 | 111.5119 |
| Incidence | Guinea-Bissau | Vitamin A deficiency | 27544.15 | 31740.5 | 23660 |
| Incidence | Switzerland | Iodine deficiency | 27.79487 | 35.41765 | 21.70604 |
| Incidence | Switzerland | Vitamin A deficiency | 322.8196 | 396.447 | 264.3923 |
| Incidence | Liberia | Iodine deficiency | 36.66859 | 46.49159 | 28.81447 |
| Incidence | Liberia | Vitamin A deficiency | 13461.13 | 16479.8 | 10787.56 |
| Incidence | Grenada | Iodine deficiency | 12.61071 | 15.92982 | 9.680632 |
| Incidence | South Africa | Iodine deficiency | 31.91516 | 39.77672 | 25.22488 |
| Incidence | South Africa | Vitamin A deficiency | 4066.682 | 4986.806 | 3287.257 |
| Incidence | South Africa | Dietary iron deficiency | 0 | 0 | 0 |
| Incidence | Grenada | Vitamin A deficiency | 3614.875 | 4298.71 | 3008.893 |
| Incidence | Mali | Iodine deficiency | 43.00724 | 54.62884 | 33.71774 |
| Incidence | Mali | Vitamin A deficiency | 30561.22 | 35008.25 | 26304.47 |
| Incidence | Guyana | Iodine deficiency | 23.32993 | 29.84973 | 18.11453 |
| Incidence | Guyana | Vitamin A deficiency | 4953.899 | 5881.248 | 4161.422 |
| Incidence | Zimbabwe | Vitamin A deficiency | 19669.56 | 22884.43 | 16646.11 |
| Incidence | United Arab Emirates | Iodine deficiency | 51.93528 | 64.1724 | 41.09186 |
| Incidence | United Arab Emirates | Vitamin A deficiency | 729.8427 | 919.2031 | 578.0731 |
| Incidence | Papua New Guinea | Vitamin A deficiency | 11735.76 | 14094.3 | 9736.368 |
| Incidence | Zambia | Vitamin A deficiency | 13680.37 | 16283.86 | 11466.73 |
| Incidence | Eritrea | Vitamin A deficiency | 22128.39 | 25563.98 | 18798.03 |
| Incidence | Sao Tome and Principe | Iodine deficiency | 41.27529 | 52.24423 | 32.54626 |
| Incidence | Sao Tome and Principe | Vitamin A deficiency | 13261.5 | 16156.53 | 10897.33 |
| Incidence | Myanmar | Iodine deficiency | 23.67136 | 30.50113 | 17.99503 |
| Incidence | Myanmar | Vitamin A deficiency | 5309.209 | 6548.45 | 4283.055 |
| Incidence | Samoa | Iodine deficiency | 2.650045 | 3.504933 | 1.89528 |
| Incidence | Samoa | Vitamin A deficiency | 17957.84 | 21397.02 | 14963.12 |
| Incidence | Cuba | Iodine deficiency | 17.12688 | 21.72264 | 13.23672 |
| Incidence | Cuba | Vitamin A deficiency | 2264.937 | 2660.788 | 1936.662 |
| Incidence | Gabon | Iodine deficiency | 117.5416 | 147.1362 | 91.57816 |
| Incidence | Gabon | Vitamin A deficiency | 4611.673 | 5688.785 | 3770.01 |
| Incidence | Solomon Islands | Iodine deficiency | 3.762172 | 4.974885 | 2.780149 |
| Incidence | Solomon Islands | Vitamin A deficiency | 27989.22 | 32287.78 | 23742.88 |
| Incidence | Fiji | Iodine deficiency | 3.342779 | 4.368502 | 2.451186 |
| Incidence | Fiji | Vitamin A deficiency | 7759.075 | 9738.158 | 6053.246 |
| Incidence | Algeria | Iodine deficiency | 29.93689 | 37.1662 | 23.53794 |
| Incidence | Algeria | Vitamin A deficiency | 2690.399 | 3276.271 | 2223.701 |
| Incidence | Bahrain | Iodine deficiency | 21.34565 | 26.52673 | 16.58589 |
| Incidence | Bahrain | Vitamin A deficiency | 1459.857 | 1773.067 | 1201 |
| Incidence | North Macedonia | Iodine deficiency | 10.89512 | 13.67248 | 8.357366 |
| Incidence | North Macedonia | Vitamin A deficiency | 12794.14 | 14829.76 | 11158.74 |
| Incidence | Greece | Iodine deficiency | 38.26603 | 48.60362 | 29.60384 |
| Incidence | Greece | Vitamin A deficiency | 762.9608 | 932.8873 | 630.8775 |
| Incidence | Qatar | Iodine deficiency | 18.53972 | 23.2709 | 14.35307 |
| Incidence | Guam | Iodine deficiency | 2.880993 | 3.800458 | 2.106165 |
| Incidence | Guam | Vitamin A deficiency | 3113.348 | 3874.458 | 2469.01 |
| Incidence | Burundi | Iodine deficiency | 112.6114 | 145.7099 | 88.68481 |
| Incidence | Burundi | Vitamin A deficiency | 22039.35 | 25405.87 | 18730.43 |
| Incidence | Palestine | Iodine deficiency | 23.15203 | 29.13393 | 18.29354 |
| Incidence | Palestine | Vitamin A deficiency | 5435.633 | 6784.057 | 4444.949 |
| Incidence | Nepal | Iodine deficiency | 60.56966 | 76.07061 | 46.90564 |
| Incidence | Comoros | Iodine deficiency | 22.15644 | 27.82918 | 17.40196 |
| Incidence | Comoros | Vitamin A deficiency | 19533.69 | 22517.14 | 16776.57 |
| Incidence | Oman | Iodine deficiency | 21.4986 | 26.82844 | 16.99449 |
| Incidence | Oman | Vitamin A deficiency | 1300.799 | 1633.511 | 1050.088 |
| Incidence | Djibouti | Iodine deficiency | 476.6281 | 582.4846 | 379.2161 |
| Incidence | Djibouti | Vitamin A deficiency | 16576.49 | 19322.85 | 14006.6 |
| Incidence | Eritrea | Iodine deficiency | 27.84927 | 35.5233 | 21.93999 |
| Incidence | Libya | Iodine deficiency | 51.91655 | 63.9815 | 41.45668 |
| Incidence | Libya | Vitamin A deficiency | 1829.158 | 2213.545 | 1515.347 |
| Incidence | Bangladesh | Iodine deficiency | 124.759 | 157.7503 | 95.88141 |
| Incidence | Bangladesh | Vitamin A deficiency | 4761.947 | 5647.548 | 4024.377 |
| Incidence | Bhutan | Iodine deficiency | 30.70134 | 38.88228 | 23.79935 |
| Incidence | Bhutan | Vitamin A deficiency | 5858.628 | 6905.898 | 4894.756 |
| Incidence | Dominica | Iodine deficiency | 15.04031 | 18.9928 | 11.61125 |
| Incidence | Dominica | Vitamin A deficiency | 2129.113 | 2528.768 | 1789.019 |
| Incidence | Seychelles | Iodine deficiency | 17.78945 | 22.4604 | 13.70195 |
| Incidence | Seychelles | Vitamin A deficiency | 1370.172 | 1707.768 | 1121.33 |
| Incidence | Haiti | Iodine deficiency | 49.43354 | 62.58877 | 37.6441 |
| Incidence | Haiti | Vitamin A deficiency | 13512.71 | 15853.4 | 11514.77 |
| Incidence | Jamaica | Iodine deficiency | 17.31645 | 22.07975 | 13.24026 |
| Incidence | Jamaica | Vitamin A deficiency | 2504.458 | 2927.247 | 2122.729 |
| Incidence | San Marino | Iodine deficiency | 36.87867 | 47.38912 | 28.9295 |
| Incidence | San Marino | Vitamin A deficiency | 443.7076 | 532.0876 | 366.6617 |
| Incidence | Argentina | Iodine deficiency | 8.313059 | 10.61546 | 6.188064 |
| Incidence | Argentina | Vitamin A deficiency | 7788.852 | 9183.883 | 6532.276 |
| Incidence | Montenegro | Iodine deficiency | 10.62523 | 13.49468 | 8.071641 |
| Incidence | Montenegro | Vitamin A deficiency | 6333.41 | 7256.333 | 5458.873 |
| Incidence | Chile | Iodine deficiency | 8.364687 | 10.67806 | 6.2605 |
| Incidence | Chile | Vitamin A deficiency | 4106.518 | 4908.798 | 3404.172 |
| Incidence | Tokelau | Iodine deficiency | 3.384642 | 4.422291 | 2.473876 |
| Incidence | Tokelau | Vitamin A deficiency | 10190.53 | 12598.05 | 8207.301 |
| Incidence | Romania | Iodine deficiency | 21.92794 | 27.19401 | 16.93993 |
| Incidence | Romania | Vitamin A deficiency | 7124.726 | 8178.258 | 6167.448 |
| Incidence | Vanuatu | Iodine deficiency | 23.94851 | 31.75783 | 18.58658 |
| Incidence | Vanuatu | Vitamin A deficiency | 28080.41 | 32084.44 | 24054.27 |
| Incidence | Uruguay | Iodine deficiency | 8.540732 | 10.81317 | 6.422794 |
| Incidence | Japan | Iodine deficiency | 15.41699 | 19.26417 | 12.10664 |
| Incidence | Japan | Vitamin A deficiency | 712.4944 | 851.5888 | 592.1525 |
| Incidence | Japan | Dietary iron deficiency | 0 | 0 | 0 |
| Incidence | Singapore | Iodine deficiency | 13.9292 | 17.52123 | 10.97121 |
| Incidence | Singapore | Vitamin A deficiency | 665.9455 | 796.3803 | 552.456 |
| Incidence | Colombia | Vitamin A deficiency | 2925.538 | 3459.789 | 2459.16 |
| Incidence | Armenia | Iodine deficiency | 40.81375 | 51.31257 | 31.42977 |
| Incidence | Armenia | Vitamin A deficiency | 494.5752 | 579.7326 | 414.769 |
| Incidence | Australia | Iodine deficiency | 12.0844 | 15.13225 | 9.369069 |
| Incidence | Australia | Vitamin A deficiency | 77.34004 | 90.92446 | 66.03838 |
| Incidence | Thailand | Iodine deficiency | 25.15747 | 32.36667 | 19.07127 |
| Incidence | Thailand | Vitamin A deficiency | 1594.68 | 1918.043 | 1341.931 |
| Incidence | Andorra | Iodine deficiency | 35.96294 | 45.95466 | 27.99597 |
| Incidence | Andorra | Vitamin A deficiency | 365.0513 | 437.4743 | 300.6646 |
| Incidence | Mexico | Vitamin A deficiency | 7181.881 | 8558.128 | 5990.597 |
| Incidence | Mexico | Dietary iron deficiency | 0 | 0 | 0 |
| Incidence | Timor-Leste | Iodine deficiency | 21.23322 | 27.28823 | 16.08324 |
| Incidence | Timor-Leste | Vitamin A deficiency | 8083.782 | 10002.27 | 6551.794 |
| Incidence | Viet Nam | Iodine deficiency | 35.90708 | 46.59364 | 27.18323 |
| Incidence | Viet Nam | Vitamin A deficiency | 1674.852 | 2240.9 | 1280.325 |
| Incidence | Yemen | Iodine deficiency | 111.1142 | 139.4141 | 87.18252 |
| Incidence | Yemen | Vitamin A deficiency | 15059.25 | 17887.4 | 12423.07 |
| Incidence | Uzbekistan | Iodine deficiency | 25.61271 | 32.77418 | 19.44811 |
| Incidence | Uzbekistan | Vitamin A deficiency | 2979.091 | 3597.284 | 2484.751 |
| Incidence | Afghanistan | Iodine deficiency | 67.86243 | 82.95129 | 53.45287 |
| Incidence | Afghanistan | Vitamin A deficiency | 26013.77 | 30088.73 | 22250.86 |
| Incidence | Paraguay | Iodine deficiency | 6.881224 | 8.805883 | 5.248707 |
| Incidence | Paraguay | Vitamin A deficiency | 6379.223 | 7614.219 | 5318.967 |
| Incidence | Tonga | Iodine deficiency | 3.614809 | 4.723537 | 2.613028 |
| Incidence | Tonga | Vitamin A deficiency | 12528.03 | 15246.97 | 10191.31 |
| Incidence | Morocco | Iodine deficiency | 66.27282 | 80.23279 | 52.27096 |
| Incidence | Morocco | Vitamin A deficiency | 6117.33 | 7318.736 | 5055.529 |
| Incidence | India | Iodine deficiency | 218.6812 | 275.9222 | 169.7753 |
| Incidence | Nepal | Vitamin A deficiency | 5693.313 | 6728.387 | 4776.572 |
| Incidence | Botswana | Iodine deficiency | 17.53899 | 22.09796 | 13.71012 |
| Incidence | Angola | Iodine deficiency | 277.2535 | 342.3174 | 220.5886 |
| Incidence | Angola | Vitamin A deficiency | 14328.4 | 17269.88 | 11671.06 |
| Incidence | Central African Republic | Iodine deficiency | 253.2356 | 317.1381 | 201.7967 |
| Incidence | Central African Republic | Vitamin A deficiency | 33496.12 | 37726.42 | 29500.63 |
| Incidence | Kiribati | Iodine deficiency | 4.701699 | 6.061238 | 3.517174 |
| Incidence | Kiribati | Vitamin A deficiency | 26227.13 | 30289.12 | 22408.78 |
| Incidence | Azerbaijan | Iodine deficiency | 11.64004 | 14.95369 | 8.791786 |
| Incidence | Azerbaijan | Vitamin A deficiency | 2574.202 | 3099.158 | 2141.14 |
| Incidence | Madagascar | Iodine deficiency | 95.20386 | 119.2617 | 73.93015 |
| Incidence | Madagascar | Vitamin A deficiency | 21864.71 | 25124.57 | 18803.93 |
| Incidence | Qatar | Vitamin A deficiency | 725.7576 | 927.3606 | 575.058 |
| Incidence | Saudi Arabia | Iodine deficiency | 9.072398 | 11.50005 | 6.985363 |
| Incidence | Saudi Arabia | Vitamin A deficiency | 190.0431 | 229.044 | 156.4873 |
| Incidence | Syrian Arab Republic | Iodine deficiency | 57.14162 | 70.04754 | 44.76236 |
| Incidence | Syrian Arab Republic | Vitamin A deficiency | 3722.355 | 4442.218 | 3067.316 |
| Incidence | Senegal | Iodine deficiency | 76.87614 | 97.02168 | 60.22279 |
| Incidence | Senegal | Vitamin A deficiency | 14427.53 | 17808.84 | 11765.99 |
| Incidence | Iceland | Iodine deficiency | 17.67158 | 22.44941 | 13.84671 |
| Incidence | Iceland | Vitamin A deficiency | 500.9516 | 608.7013 | 414.122 |
| Incidence | Sierra Leone | Iodine deficiency | 61.40191 | 77.50867 | 48.16238 |
| Incidence | Sierra Leone | Vitamin A deficiency | 23318.25 | 27130.19 | 19733.15 |
| Incidence | Ecuador | Iodine deficiency | 4.749168 | 6.264678 | 3.562477 |
| Incidence | Ecuador | Vitamin A deficiency | 3770.762 | 4652.233 | 3036.987 |
| Incidence | United Republic of Tanzania | Iodine deficiency | 24.24264 | 30.76565 | 19.15218 |
| Incidence | United Republic of Tanzania | Vitamin A deficiency | 13929.63 | 16214.66 | 11912.06 |
| Incidence | Cameroon | Iodine deficiency | 37.83901 | 47.95348 | 29.70421 |
| Incidence | Cameroon | Vitamin A deficiency | 27949.82 | 31961.96 | 23794.23 |
| Incidence | Uganda | Iodine deficiency | 46.34455 | 59.04 | 36.30175 |
| Incidence | Uganda | Vitamin A deficiency | 11785.58 | 13818.26 | 10055.06 |
| Incidence | Cabo Verde | Iodine deficiency | 71.7486 | 91.47697 | 56.14673 |
| Incidence | Cabo Verde | Vitamin A deficiency | 5248.875 | 6489.234 | 4294.078 |
| Incidence | Zambia | Iodine deficiency | 52.5634 | 66.45359 | 41.00643 |
| Incidence | Chad | Iodine deficiency | 56.99231 | 72.63755 | 44.24558 |
| Incidence | Philippines | Iodine deficiency | 106.3795 | 135.6028 | 81.05421 |
| Incidence | Philippines | Vitamin A deficiency | 9309.22 | 11135.15 | 7668.954 |
| Incidence | Philippines | Dietary iron deficiency | 0 | 0 | 0 |
| Incidence | Mauritania | Iodine deficiency | 138.8962 | 176.3797 | 108.2545 |
| Incidence | Mauritania | Vitamin A deficiency | 10418.29 | 12756.34 | 8536.492 |
| Incidence | Antigua and Barbuda | Iodine deficiency | 9.580832 | 12.39256 | 7.380468 |
| Incidence | Antigua and Barbuda | Vitamin A deficiency | 1945.287 | 2294.083 | 1628.891 |
| Incidence | Malawi | Iodine deficiency | 110.7435 | 140.1829 | 86.42006 |
| Incidence | Malawi | Vitamin A deficiency | 25987.25 | 29970.98 | 22397.78 |
| Incidence | Lesotho | Iodine deficiency | 149.0398 | 190.1704 | 117.0909 |
| Incidence | Lesotho | Vitamin A deficiency | 15405.14 | 18346.03 | 13011.94 |
| Incidence | Bosnia and Herzegovina | Iodine deficiency | 19.60677 | 24.54451 | 14.91547 |
| Incidence | Bosnia and Herzegovina | Vitamin A deficiency | 8900.141 | 10266.41 | 7662.504 |
| Incidence | Botswana | Vitamin A deficiency | 9632.487 | 11592.91 | 7920.46 |
| Incidence | United Kingdom | Iodine deficiency | 38.95152 | 49.39947 | 30.42792 |
| Incidence | United Kingdom | Vitamin A deficiency | 598.8678 | 737.247 | 485.2744 |
| Incidence | United Kingdom | Dietary iron deficiency | 0 | 0 | 0 |
| Incidence | Peru | Iodine deficiency | 4.647072 | 6.043169 | 3.471862 |
| Incidence | Peru | Vitamin A deficiency | 6087.75 | 7231.62 | 5098.068 |
| Incidence | Colombia | Iodine deficiency | 24.5753 | 30.71174 | 18.59874 |
| Incidence | Georgia | Iodine deficiency | 15.48026 | 19.97762 | 11.72927 |
| Incidence | Georgia | Vitamin A deficiency | 2335.211 | 2798.308 | 1971.623 |
| Incidence | Egypt | Iodine deficiency | 35.30775 | 43.72743 | 27.60882 |
| Incidence | Egypt | Vitamin A deficiency | 1925.178 | 2320.363 | 1590.418 |
| Incidence | Iraq | Iodine deficiency | 53.06042 | 65.00984 | 41.9119 |
| Incidence | Iraq | Vitamin A deficiency | 3209.164 | 3937.858 | 2619.096 |
| Incidence | China | Iodine deficiency | 62.39887 | 78.16427 | 48.57565 |
| Incidence | China | Vitamin A deficiency | 2113.341 | 2553.636 | 1764.061 |
| Incidence | Slovenia | Iodine deficiency | 10.03629 | 12.67036 | 7.60382 |
| Incidence | Slovenia | Vitamin A deficiency | 4867.492 | 5622.748 | 4198.266 |
| Incidence | Taiwan (Province of China) | Iodine deficiency | 5.720474 | 7.431407 | 4.343303 |
| Incidence | Taiwan (Province of China) | Vitamin A deficiency | 647.0691 | 785.2294 | 531.9855 |
| Incidence | Belarus | Iodine deficiency | 7.256276 | 9.352313 | 5.389647 |
| Incidence | Belarus | Vitamin A deficiency | 1286.971 | 1503.087 | 1099.852 |
| Incidence | Cambodia | Iodine deficiency | 34.11322 | 44.87386 | 25.73304 |
| Incidence | Cambodia | Vitamin A deficiency | 7755.021 | 9471.093 | 6235.502 |
| Incidence | Estonia | Iodine deficiency | 5.128558 | 6.630464 | 3.764792 |
| Incidence | Estonia | Vitamin A deficiency | 713.553 | 827.2051 | 606.5046 |
| Incidence | Uruguay | Vitamin A deficiency | 5121.545 | 6222.503 | 4190.822 |
| Incidence | Bulgaria | Iodine deficiency | 8.402248 | 10.76914 | 6.324848 |
| Incidence | Bulgaria | Vitamin A deficiency | 8299.087 | 9591.05 | 7160.684 |
| Incidence | Croatia | Iodine deficiency | 10.32172 | 13.11115 | 7.817472 |
| Incidence | Croatia | Vitamin A deficiency | 7409.325 | 8556.062 | 6414.407 |
| Incidence | Canada | Iodine deficiency | 12.88369 | 16.1894 | 10.07568 |
| Incidence | Canada | Vitamin A deficiency | 906.4726 | 1120.87 | 739.0465 |
| Prevalence | Lao People's Democratic Republic | Iodine deficiency | 366.9132 | 464.8493 | 286.0927 |
| Prevalence | Lao People's Democratic Republic | Vitamin A deficiency | 11596.34 | 13603.04 | 9592.967 |
| Prevalence | Lao People's Democratic Republic | Dietary iron deficiency | 16381.19 | 18081.76 | 14819 |
| Prevalence | Dominican Republic | Iodine deficiency | 354.0778 | 455.3374 | 269.1931 |
| Prevalence | Dominican Republic | Vitamin A deficiency | 4835.136 | 5695.192 | 4044.739 |
| Prevalence | Dominican Republic | Dietary iron deficiency | 12218.03 | 13676.44 | 10698.26 |
| Prevalence | Japan | Iodine deficiency | 262.6113 | 334.6104 | 201.347 |
| Prevalence | Japan | Vitamin A deficiency | 712.4944 | 851.5888 | 592.1525 |
| Prevalence | Bulgaria | Iodine deficiency | 142.248 | 183.8269 | 106.1439 |
| Prevalence | Bulgaria | Vitamin A deficiency | 8299.087 | 9591.05 | 7160.684 |
| Prevalence | Bulgaria | Dietary iron deficiency | 9708.875 | 11430.59 | 8139.017 |
| Prevalence | Bolivia (Plurinational State of) | Iodine deficiency | 88.07964 | 116.2374 | 65.33425 |
| Prevalence | Bolivia (Plurinational State of) | Vitamin A deficiency | 8475.208 | 10156.55 | 6977.247 |
| Prevalence | Bolivia (Plurinational State of) | Dietary iron deficiency | 17694.73 | 19677.34 | 15959.04 |
| Prevalence | Canada | Iodine deficiency | 218.6115 | 279.7141 | 165.7012 |
| Prevalence | Canada | Vitamin A deficiency | 906.4726 | 1120.87 | 739.0465 |
| Prevalence | Canada | Dietary iron deficiency | 2566.481 | 3212.915 | 1987.513 |
| Prevalence | Japan | Dietary iron deficiency | 7388.848 | 8734.247 | 6250.355 |
| Prevalence | Maldives | Iodine deficiency | 202.6308 | 261.548 | 158.2292 |
| Prevalence | Maldives | Vitamin A deficiency | 2948.436 | 3559.24 | 2340.179 |
| Prevalence | Maldives | Dietary iron deficiency | 11753.18 | 13529.02 | 9984.542 |
| Prevalence | Norway | Iodine deficiency | 755.5686 | 965.9209 | 588.5503 |
| Prevalence | Norway | Vitamin A deficiency | 399.4748 | 499.2764 | 324.3725 |
| Prevalence | Peru | Iodine deficiency | 74.68862 | 98.76378 | 54.91349 |
| Prevalence | Peru | Vitamin A deficiency | 6087.75 | 7231.62 | 5098.068 |
| Prevalence | Peru | Dietary iron deficiency | 10511.28 | 11993.32 | 9151.105 |
| Prevalence | Czechia | Iodine deficiency | 106.2104 | 136.3254 | 79.38406 |
| Prevalence | Czechia | Vitamin A deficiency | 5505.387 | 6331.709 | 4722.655 |
| Prevalence | Czechia | Dietary iron deficiency | 6885.543 | 8174.416 | 5636.564 |
| Prevalence | Republic of Korea | Iodine deficiency | 236.1301 | 300.0502 | 178.7684 |
| Prevalence | Republic of Korea | Vitamin A deficiency | 620.7092 | 750.3216 | 521.6364 |
| Prevalence | Republic of Korea | Dietary iron deficiency | 7936.151 | 9260.888 | 6786.419 |
| Prevalence | Pakistan | Iodine deficiency | 6223.115 | 7655.712 | 5041.894 |
| Prevalence | Pakistan | Vitamin A deficiency | 3521.685 | 4256.642 | 2883.567 |
| Prevalence | Pakistan | Dietary iron deficiency | 26563.29 | 28619.38 | 24562.26 |
| Prevalence | France | Iodine deficiency | 768.7095 | 977.597 | 598.9721 |
| Prevalence | France | Vitamin A deficiency | 172.5094 | 210.3543 | 141.6564 |
| Prevalence | France | Dietary iron deficiency | 1990.9 | 2851.17 | 1422.244 |
| Prevalence | Netherlands | Iodine deficiency | 722.9431 | 923.2836 | 556.8664 |
| Prevalence | Estonia | Iodine deficiency | 87.99138 | 114.5782 | 63.53788 |
| Prevalence | Netherlands | Vitamin A deficiency | 481.1534 | 594.3412 | 395.0142 |
| Prevalence | Netherlands | Dietary iron deficiency | 2381.78 | 3365.619 | 1708.241 |
| Prevalence | United Arab Emirates | Iodine deficiency | 1023.745 | 1257.265 | 792.0528 |
| Prevalence | United Arab Emirates | Vitamin A deficiency | 729.8427 | 919.2031 | 578.0731 |
| Prevalence | United Arab Emirates | Dietary iron deficiency | 8933.994 | 10542.2 | 7421.18 |
| Prevalence | Nauru | Iodine deficiency | 51.11455 | 68.33741 | 36.61396 |
| Prevalence | Nauru | Vitamin A deficiency | 11349.06 | 13719.79 | 9161.41 |
| Prevalence | Nauru | Dietary iron deficiency | 17308.45 | 19400.04 | 15471.63 |
| Prevalence | United Republic of Tanzania | Iodine deficiency | 452.9734 | 572.4896 | 355.7595 |
| Prevalence | Kuwait | Iodine deficiency | 347.2883 | 432.3048 | 269.6544 |
| Prevalence | Kuwait | Vitamin A deficiency | 745.6719 | 912.1799 | 619.932 |
| Prevalence | Kuwait | Dietary iron deficiency | 8580.089 | 9875.22 | 7407.013 |
| Prevalence | Sri Lanka | Iodine deficiency | 903.4176 | 1160.611 | 706.1341 |
| Prevalence | Sri Lanka | Vitamin A deficiency | 3499.452 | 4281.622 | 2892.138 |
| Prevalence | Sri Lanka | Dietary iron deficiency | 12237.32 | 13886.86 | 10646.68 |
| Prevalence | Grenada | Iodine deficiency | 227.7511 | 292.8491 | 171.8985 |
| Prevalence | Grenada | Vitamin A deficiency | 3614.875 | 4298.71 | 3008.893 |
| Prevalence | Grenada | Dietary iron deficiency | 14398.33 | 16359.14 | 12577.84 |
| Prevalence | Niue | Iodine deficiency | 49.78894 | 66.95645 | 35.2265 |
| Prevalence | Niue | Vitamin A deficiency | 5712.037 | 7092.964 | 4530.453 |
| Prevalence | Norway | Dietary iron deficiency | 2830.686 | 3874.782 | 2124.526 |
| Prevalence | Latvia | Iodine deficiency | 93.94821 | 122.4254 | 67.74278 |
| Prevalence | Latvia | Vitamin A deficiency | 757.1049 | 891.0874 | 642.8161 |
| Prevalence | Latvia | Dietary iron deficiency | 7856.636 | 9253.976 | 6593.499 |
| Prevalence | Kazakhstan | Iodine deficiency | 369.5407 | 466.0365 | 276.9558 |
| Prevalence | Kazakhstan | Vitamin A deficiency | 7611.972 | 9212.276 | 6200.297 |
| Prevalence | Kazakhstan | Dietary iron deficiency | 15592.09 | 17355.27 | 14014.89 |
| Prevalence | Portugal | Iodine deficiency | 1187.648 | 1524.944 | 917.3237 |
| Prevalence | Portugal | Vitamin A deficiency | 1015.76 | 1219.854 | 829.0114 |
| Prevalence | Portugal | Dietary iron deficiency | 3199.839 | 4239.109 | 2398.334 |
| Prevalence | Myanmar | Dietary iron deficiency | 21671.88 | 23443.76 | 20089.87 |
| Prevalence | Angola | Iodine deficiency | 7525.574 | 9342.463 | 6061.523 |
| Prevalence | Angola | Vitamin A deficiency | 14328.4 | 17269.88 | 11671.06 |
| Prevalence | Angola | Dietary iron deficiency | 15856.86 | 17666.23 | 14160.9 |
| Prevalence | China | Iodine deficiency | 1449.914 | 1821.404 | 1158.998 |
| Prevalence | China | Vitamin A deficiency | 2113.341 | 2553.636 | 1764.061 |
| Prevalence | Brazil | Iodine deficiency | 104.8201 | 138.0132 | 78.51031 |
| Prevalence | Brazil | Vitamin A deficiency | 10128.64 | 11793.54 | 8668.303 |
| Prevalence | Brazil | Dietary iron deficiency | 12810.22 | 14163.34 | 11420.4 |
| Prevalence | Niue | Dietary iron deficiency | 14320.04 | 16116.66 | 12532.17 |
| Prevalence | Uganda | Iodine deficiency | 1001.158 | 1272.619 | 787.9863 |
| Prevalence | Uganda | Vitamin A deficiency | 11785.58 | 13818.26 | 10055.06 |
| Prevalence | Uganda | Dietary iron deficiency | 14208 | 15680.1 | 12760.74 |
| Prevalence | Mauritania | Iodine deficiency | 3469.787 | 4385.751 | 2738.629 |
| Prevalence | Mauritania | Vitamin A deficiency | 10418.29 | 12756.34 | 8536.492 |
| Prevalence | Mauritania | Dietary iron deficiency | 20503.79 | 23044.59 | 14364.49 |
| Prevalence | Northern Mariana Islands | Dietary iron deficiency | 13131.76 | 15036.4 | 11332.82 |
| Prevalence | Burkina Faso | Iodine deficiency | 1074.872 | 1381.644 | 841.204 |
| Prevalence | Burkina Faso | Vitamin A deficiency | 27594.06 | 31725.92 | 23930.23 |
| Prevalence | Burkina Faso | Dietary iron deficiency | 23592.38 | 27796.94 | 20179.76 |
| Prevalence | North Macedonia | Iodine deficiency | 188.6554 | 243.4581 | 142.0044 |
| Prevalence | North Macedonia | Vitamin A deficiency | 12794.14 | 14829.76 | 11158.74 |
| Prevalence | North Macedonia | Dietary iron deficiency | 8776.064 | 10097.46 | 7545.002 |
| Prevalence | Jamaica | Iodine deficiency | 335.2688 | 435.9703 | 254.2946 |
| Prevalence | Jamaica | Vitamin A deficiency | 2504.458 | 2927.247 | 2122.729 |
| Prevalence | Jamaica | Dietary iron deficiency | 15301.62 | 17294.42 | 13425.95 |
| Prevalence | Dominica | Dietary iron deficiency | 14200.37 | 15941.65 | 12591.17 |
| Prevalence | Congo | Iodine deficiency | 11188.9 | 13535.18 | 9050.566 |
| Prevalence | Congo | Vitamin A deficiency | 27043.51 | 30947.28 | 23493.53 |
| Prevalence | Congo | Dietary iron deficiency | 20478.55 | 23712.75 | 16723.75 |
| Prevalence | United States of America | Iodine deficiency | 230.3479 | 292.666 | 175.4408 |
| Prevalence | United States of America | Vitamin A deficiency | 440.0833 | 541.2735 | 357.0883 |
| Prevalence | United States of America | Dietary iron deficiency | 4188.29 | 4832.694 | 3599.867 |
| Prevalence | Antigua and Barbuda | Iodine deficiency | 165.2188 | 212.4967 | 124.4142 |
| Prevalence | Antigua and Barbuda | Vitamin A deficiency | 1945.287 | 2294.083 | 1628.891 |
| Prevalence | Antigua and Barbuda | Dietary iron deficiency | 14005.54 | 15952.9 | 12169.31 |
| Prevalence | Barbados | Iodine deficiency | 448.5267 | 576.8148 | 344.7842 |
| Prevalence | Barbados | Vitamin A deficiency | 2031.763 | 2371.303 | 1719.501 |
| Prevalence | Barbados | Dietary iron deficiency | 10758.37 | 12558.08 | 9069.689 |
| Prevalence | Australia | Iodine deficiency | 202.3672 | 257.6967 | 152.9679 |
| Prevalence | Australia | Vitamin A deficiency | 77.34004 | 90.92446 | 66.03838 |
| Prevalence | Australia | Dietary iron deficiency | 4231.765 | 5453.34 | 3282.671 |
| Prevalence | China | Dietary iron deficiency | 4559.355 | 4952.525 | 4166.887 |
| Prevalence | Costa Rica | Iodine deficiency | 453.5325 | 585.0693 | 344.1282 |
| Prevalence | Costa Rica | Vitamin A deficiency | 2864.118 | 3417.047 | 2411.396 |
| Prevalence | Costa Rica | Dietary iron deficiency | 6318.701 | 7528.254 | 5174.502 |
| Prevalence | New Zealand | Iodine deficiency | 241.1955 | 306.6381 | 186.3139 |
| Prevalence | New Zealand | Vitamin A deficiency | 541.3629 | 650.7298 | 444.7329 |
| Prevalence | New Zealand | Dietary iron deficiency | 4632.222 | 5900.091 | 3573.843 |
| Prevalence | Germany | Iodine deficiency | 748.0054 | 959.0432 | 582.7816 |
| Prevalence | Germany | Vitamin A deficiency | 334.9781 | 399.4373 | 282.3568 |
| Prevalence | Germany | Dietary iron deficiency | 2528.478 | 3210.519 | 1924.923 |
| Prevalence | Democratic People's Republic of Korea | Iodine deficiency | 63.08935 | 83.16419 | 46.18563 |
| Prevalence | Democratic People's Republic of Korea | Vitamin A deficiency | 7105.379 | 8577.585 | 5858.878 |
| Prevalence | Democratic People's Republic of Korea | Dietary iron deficiency | 12566.2 | 13967.43 | 11328.28 |
| Prevalence | Turkmenistan | Iodine deficiency | 286.0892 | 363.9306 | 215.242 |
| Prevalence | Turkmenistan | Vitamin A deficiency | 2273.884 | 2740.357 | 1881.51 |
| Prevalence | Turkmenistan | Dietary iron deficiency | 13884.02 | 15495.9 | 12413.12 |
| Prevalence | Ireland | Iodine deficiency | 745.4708 | 956.2373 | 579.5629 |
| Prevalence | Ireland | Vitamin A deficiency | 479.6504 | 580.4343 | 383.6712 |
| Prevalence | Saint Lucia | Iodine deficiency | 125.3433 | 164.0687 | 94.2623 |
| Prevalence | Saint Lucia | Vitamin A deficiency | 3007.208 | 3490.052 | 2581.265 |
| Prevalence | Saint Lucia | Dietary iron deficiency | 15966.46 | 18307.04 | 14086.22 |
| Prevalence | Ireland | Dietary iron deficiency | 2418.901 | 3226.282 | 1735.422 |
| Prevalence | Democratic Republic of the Congo | Iodine deficiency | 16385 | 19590.16 | 13383.05 |
| Prevalence | Democratic Republic of the Congo | Vitamin A deficiency | 30146.05 | 34484.43 | 26304.29 |
| Prevalence | Democratic Republic of the Congo | Dietary iron deficiency | 18880.24 | 20476.15 | 17082.17 |
| Prevalence | Cote d'Ivoire | Iodine deficiency | 1074.505 | 1376.39 | 833.0015 |
| Prevalence | Cote d'Ivoire | Vitamin A deficiency | 14240.01 | 17152.56 | 11642.12 |
| Prevalence | Cote d'Ivoire | Dietary iron deficiency | 21276.46 | 23208.11 | 18977.42 |
| Prevalence | Trinidad and Tobago | Iodine deficiency | 345.3146 | 445.7427 | 261.4271 |
| Prevalence | Trinidad and Tobago | Vitamin A deficiency | 1982.991 | 2315.83 | 1691.944 |
| Prevalence | Singapore | Iodine deficiency | 236.5887 | 302.5065 | 180.2037 |
| Prevalence | Singapore | Vitamin A deficiency | 665.9455 | 796.3803 | 552.456 |
| Prevalence | Singapore | Dietary iron deficiency | 6683.913 | 7912.432 | 5561.153 |
| Prevalence | Bahrain | Iodine deficiency | 358.0973 | 449.3935 | 275.8309 |
| Prevalence | Bahrain | Vitamin A deficiency | 1459.857 | 1773.067 | 1201 |
| Prevalence | Bahrain | Dietary iron deficiency | 8571.76 | 10060.88 | 7073.498 |
| Prevalence | Trinidad and Tobago | Dietary iron deficiency | 15465.27 | 17611.92 | 13408.29 |
| Prevalence | San Marino | Iodine deficiency | 758.319 | 975.4685 | 585.4968 |
| Prevalence | San Marino | Vitamin A deficiency | 443.7076 | 532.0876 | 366.6617 |
| Prevalence | San Marino | Dietary iron deficiency | 2536.64 | 3342.46 | 1893.887 |
| Prevalence | Argentina | Iodine deficiency | 138.8264 | 179.6248 | 100.6128 |
| Prevalence | Argentina | Vitamin A deficiency | 7788.852 | 9183.883 | 6532.276 |
| Prevalence | Argentina | Dietary iron deficiency | 9306.442 | 10896.43 | 7879.744 |
| Prevalence | Saint Kitts and Nevis | Iodine deficiency | 162.9501 | 208.5124 | 121.9716 |
| Prevalence | Micronesia (Federated States of) | Iodine deficiency | 66.99081 | 89.23252 | 48.61278 |
| Prevalence | Micronesia (Federated States of) | Vitamin A deficiency | 34768.53 | 38842.13 | 30793.63 |
| Prevalence | Micronesia (Federated States of) | Dietary iron deficiency | 18672.5 | 20604.36 | 16589.18 |
| Prevalence | Poland | Iodine deficiency | 182.7043 | 235.186 | 137.3746 |
| Prevalence | Poland | Vitamin A deficiency | 5933.509 | 6864.496 | 5066.371 |
| Prevalence | Poland | Dietary iron deficiency | 8781.113 | 10372.77 | 7305.567 |
| Prevalence | Cyprus | Iodine deficiency | 799.4976 | 1029.358 | 618.9982 |
| Prevalence | Cyprus | Vitamin A deficiency | 645.6531 | 791.7477 | 524.8659 |
| Prevalence | Cyprus | Dietary iron deficiency | 2232.091 | 3012.193 | 1645.537 |
| Prevalence | Saint Kitts and Nevis | Vitamin A deficiency | 1914.566 | 2266.927 | 1601.517 |
| Prevalence | Saint Kitts and Nevis | Dietary iron deficiency | 12908.83 | 14896.23 | 11128.2 |
| Prevalence | Lesotho | Iodine deficiency | 3826.327 | 4880.2 | 3014.463 |
| Prevalence | Lesotho | Vitamin A deficiency | 15405.14 | 18346.03 | 13011.94 |
| Prevalence | Lesotho | Dietary iron deficiency | 14136.12 | 15423.44 | 12969.95 |
| Prevalence | Israel | Dietary iron deficiency | 3607.25 | 4639.481 | 2696.634 |
| Prevalence | United Kingdom | Iodine deficiency | 766.708 | 979.4747 | 598.1161 |
| Prevalence | Mexico | Iodine deficiency | 854.8969 | 1069.922 | 663.9858 |
| Prevalence | Mexico | Vitamin A deficiency | 7181.881 | 8558.128 | 5990.597 |
| Prevalence | Mexico | Dietary iron deficiency | 5442.081 | 5645.897 | 5263.186 |
| Prevalence | Ukraine | Iodine deficiency | 619.5002 | 783.4113 | 479.3573 |
| Prevalence | Egypt | Dietary iron deficiency | 10825.95 | 12430.37 | 9350.89 |
| Prevalence | United Kingdom | Vitamin A deficiency | 598.8678 | 737.247 | 485.2744 |
| Prevalence | United Kingdom | Dietary iron deficiency | 4143.068 | 5049.227 | 3431.136 |
| Prevalence | Fiji | Iodine deficiency | 52.13847 | 69.86521 | 37.23724 |
| Prevalence | Fiji | Vitamin A deficiency | 7759.075 | 9738.158 | 6053.246 |
| Prevalence | Fiji | Dietary iron deficiency | 23304.42 | 25365.44 | 21420.99 |
| Prevalence | Israel | Iodine deficiency | 791.7982 | 1005.312 | 613.698 |
| Prevalence | Israel | Vitamin A deficiency | 4414.096 | 5554.151 | 3439.742 |
| Prevalence | Nicaragua | Iodine deficiency | 696.1318 | 882.884 | 532.7542 |
| Prevalence | Nicaragua | Vitamin A deficiency | 1750.252 | 2099.561 | 1455.03 |
| Prevalence | Nicaragua | Dietary iron deficiency | 5570.351 | 6577.705 | 4677.225 |
| Prevalence | Slovakia | Iodine deficiency | 173.3661 | 222.9406 | 129.1111 |
| Prevalence | Slovakia | Vitamin A deficiency | 6072.27 | 7025.261 | 5178.874 |
| Prevalence | Slovakia | Dietary iron deficiency | 7665.487 | 9180.594 | 6287.917 |
| Prevalence | Palestine | Iodine deficiency | 403.67 | 502.3695 | 313.387 |
| Prevalence | Palestine | Vitamin A deficiency | 5435.633 | 6784.057 | 4444.949 |
| Prevalence | Palestine | Dietary iron deficiency | 8680.801 | 9929.704 | 7566.237 |
| Prevalence | Sierra Leone | Iodine deficiency | 1369.124 | 1736.953 | 1065.407 |
| Prevalence | Sierra Leone | Vitamin A deficiency | 23318.25 | 27130.19 | 19733.15 |
| Prevalence | Sierra Leone | Dietary iron deficiency | 21294.84 | 23121.46 | 19449.46 |
| Prevalence | Finland | Iodine deficiency | 761.1065 | 961.8546 | 590.7378 |
| Prevalence | Finland | Vitamin A deficiency | 527.5514 | 645.2803 | 434.1381 |
| Prevalence | Finland | Dietary iron deficiency | 2811.583 | 3923.834 | 1988.212 |
| Prevalence | Burundi | Iodine deficiency | 2762.158 | 3558.851 | 2175.503 |
| Prevalence | Burundi | Vitamin A deficiency | 22039.35 | 25405.87 | 18730.43 |
| Prevalence | Burundi | Dietary iron deficiency | 14941.32 | 16668.74 | 13425.11 |
| Prevalence | Oman | Dietary iron deficiency | 12963.3 | 15054.23 | 11000.04 |
| Prevalence | Belize | Iodine deficiency | 98.03854 | 128.7904 | 72.53165 |
| Prevalence | Belize | Vitamin A deficiency | 4673.406 | 5506.833 | 3900.079 |
| Prevalence | Belize | Dietary iron deficiency | 17442.2 | 19585.44 | 15272.99 |
| Prevalence | Italy | Iodine deficiency | 3253.466 | 4065.197 | 2604.728 |
| Prevalence | Italy | Vitamin A deficiency | 1415.512 | 1660.52 | 1206.425 |
| Prevalence | Italy | Dietary iron deficiency | 2446.138 | 3226.758 | 1785.678 |
| Prevalence | Andorra | Iodine deficiency | 730.2213 | 942.0918 | 577.0048 |
| Prevalence | Andorra | Vitamin A deficiency | 365.0513 | 437.4743 | 300.6646 |
| Prevalence | Andorra | Dietary iron deficiency | 2528.482 | 3367.538 | 1871.133 |
| Prevalence | Belgium | Iodine deficiency | 742.4103 | 952.9915 | 578.8774 |
| Prevalence | Belgium | Vitamin A deficiency | 536.0939 | 656.646 | 435.8247 |
| Prevalence | Belgium | Dietary iron deficiency | 2386.501 | 3188.491 | 1748.154 |
| Prevalence | Bangladesh | Iodine deficiency | 2977.206 | 3815.412 | 2366.077 |
| Prevalence | Bangladesh | Vitamin A deficiency | 4761.947 | 5647.548 | 4024.377 |
| Prevalence | Bangladesh | Dietary iron deficiency | 20982.39 | 22982.53 | 19108.6 |
| Prevalence | Romania | Iodine deficiency | 448.0048 | 563.3358 | 342.2795 |
| Prevalence | Equatorial Guinea | Iodine deficiency | 5559.728 | 6915.484 | 4432.68 |
| Prevalence | Equatorial Guinea | Vitamin A deficiency | 4574.185 | 5768.954 | 3671.029 |
| Prevalence | Equatorial Guinea | Dietary iron deficiency | 15879.3 | 19173.44 | 13062.37 |
| Prevalence | Romania | Vitamin A deficiency | 7124.726 | 8178.258 | 6167.448 |
| Prevalence | Romania | Dietary iron deficiency | 9558.98 | 11067.89 | 8070.091 |
| Prevalence | Guinea-Bissau | Iodine deficiency | 3603.556 | 4604.595 | 2886.501 |
| Prevalence | Guinea-Bissau | Vitamin A deficiency | 27544.15 | 31740.5 | 23660 |
| Prevalence | Timor-Leste | Iodine deficiency | 384.9199 | 498.8747 | 303.1399 |
| Prevalence | Timor-Leste | Vitamin A deficiency | 8083.782 | 10002.27 | 6551.794 |
| Prevalence | Timor-Leste | Dietary iron deficiency | 15477.05 | 17247.3 | 13862.2 |
| Prevalence | United States Virgin Islands | Iodine deficiency | 116.9426 | 153.5237 | 87.15746 |
| Prevalence | United States Virgin Islands | Vitamin A deficiency | 1070.232 | 1256.163 | 903.6089 |
| Prevalence | United States Virgin Islands | Dietary iron deficiency | 13019.58 | 15010.4 | 11188.64 |
| Prevalence | Guinea-Bissau | Dietary iron deficiency | 21981.38 | 23947.2 | 19911.07 |
| Prevalence | Cuba | Iodine deficiency | 331.2824 | 428.2561 | 252.7067 |
| Prevalence | Cuba | Vitamin A deficiency | 2264.937 | 2660.788 | 1936.662 |
| Prevalence | Cuba | Dietary iron deficiency | 11648.31 | 13462.78 | 9875.75 |
| Prevalence | Central African Republic | Iodine deficiency | 6778.07 | 8298.812 | 5440.443 |
| Prevalence | Central African Republic | Vitamin A deficiency | 33496.12 | 37726.42 | 29500.63 |
| Prevalence | Central African Republic | Dietary iron deficiency | 20084.36 | 22948.42 | 16828.92 |
| Prevalence | Tunisia | Iodine deficiency | 319.352 | 402.7943 | 247.39 |
| Prevalence | Tunisia | Vitamin A deficiency | 1754.96 | 2071.49 | 1468.023 |
| Prevalence | Tunisia | Dietary iron deficiency | 5793.338 | 6911.671 | 4726.011 |
| Prevalence | Benin | Iodine deficiency | 1029.365 | 1324.384 | 807.6993 |
| Prevalence | Benin | Vitamin A deficiency | 27262.28 | 31358.09 | 23522.25 |
| Prevalence | Benin | Dietary iron deficiency | 15692.28 | 17335.72 | 14090.32 |
| Prevalence | Papua New Guinea | Iodine deficiency | 66.38964 | 89.19001 | 48.43699 |
| Prevalence | Papua New Guinea | Vitamin A deficiency | 11735.76 | 14094.3 | 9736.368 |
| Prevalence | Papua New Guinea | Dietary iron deficiency | 18661.88 | 20351.17 | 16935.71 |
| Prevalence | India | Iodine deficiency | 5287.352 | 6593.122 | 4245.054 |
| Prevalence | India | Vitamin A deficiency | 8129.879 | 9719.208 | 6844.022 |
| Prevalence | India | Dietary iron deficiency | 28343.65 | 28888.06 | 27753.53 |
| Prevalence | Nepal | Iodine deficiency | 1279.489 | 1623.625 | 1000.592 |
| Prevalence | Nepal | Vitamin A deficiency | 5693.313 | 6728.387 | 4776.572 |
| Prevalence | Nepal | Dietary iron deficiency | 23499.82 | 25492.53 | 21630.65 |
| Prevalence | Georgia | Iodine deficiency | 293.5117 | 376.2898 | 223.2271 |
| Prevalence | Georgia | Vitamin A deficiency | 2335.211 | 2798.308 | 1971.623 |
| Prevalence | Georgia | Dietary iron deficiency | 13519.61 | 15049.9 | 12089.7 |
| Prevalence | Colombia | Iodine deficiency | 507.2762 | 645.4144 | 390.5291 |
| Prevalence | Colombia | Vitamin A deficiency | 2925.538 | 3459.789 | 2459.16 |
| Prevalence | Colombia | Dietary iron deficiency | 5349.088 | 6411.063 | 4413.818 |
| Prevalence | Chad | Iodine deficiency | 1245.987 | 1615.413 | 960.5427 |
| Prevalence | Chad | Vitamin A deficiency | 34260.53 | 38895.56 | 29897.54 |
| Prevalence | Chad | Dietary iron deficiency | 21535.22 | 23686.14 | 19342.81 |
| Prevalence | Vanuatu | Iodine deficiency | 553.063 | 736.0783 | 426.1071 |
| Prevalence | Vanuatu | Vitamin A deficiency | 28080.41 | 32084.44 | 24054.27 |
| Prevalence | Vanuatu | Dietary iron deficiency | 21129.03 | 23029.46 | 19311.8 |
| Prevalence | Venezuela (Bolivarian Republic of) | Iodine deficiency | 508.6533 | 650.7441 | 386.7198 |
| Prevalence | Honduras | Iodine deficiency | 712.5391 | 904.5828 | 546.3152 |
| Prevalence | Honduras | Vitamin A deficiency | 5901.577 | 7014.21 | 4946.865 |
| Prevalence | Honduras | Dietary iron deficiency | 10881.48 | 12315.73 | 9515.299 |
| Prevalence | Guinea | Iodine deficiency | 2772.692 | 3543.537 | 2156.057 |
| Prevalence | Guinea | Vitamin A deficiency | 24602.8 | 28472.22 | 20937.38 |
| Prevalence | Guinea | Dietary iron deficiency | 19044.78 | 20864.11 | 17055 |
| Prevalence | Ethiopia | Iodine deficiency | 10591.54 | 13123.08 | 8564.101 |
| Prevalence | Ethiopia | Vitamin A deficiency | 24329.13 | 28388.42 | 20909.75 |
| Prevalence | Ethiopia | Dietary iron deficiency | 14517.12 | 15312.09 | 13780.24 |
| Prevalence | Kenya | Iodine deficiency | 1205.585 | 1545.697 | 935.4165 |
| Prevalence | Kenya | Vitamin A deficiency | 29712.62 | 33973.7 | 26085.15 |
| Prevalence | Kenya | Dietary iron deficiency | 13073.25 | 13587.27 | 12525.88 |
| Prevalence | Venezuela (Bolivarian Republic of) | Vitamin A deficiency | 5461.028 | 6475.999 | 4549.232 |
| Prevalence | Venezuela (Bolivarian Republic of) | Dietary iron deficiency | 6204.273 | 7337.714 | 5148.579 |
| Prevalence | American Samoa | Iodine deficiency | 42.86198 | 57.99606 | 29.61981 |
| Prevalence | American Samoa | Vitamin A deficiency | 6562.33 | 8099.877 | 5256.222 |
| Prevalence | American Samoa | Dietary iron deficiency | 15390.42 | 17126.2 | 13706.52 |
| Prevalence | Mozambique | Iodine deficiency | 1142.952 | 1457.325 | 897.6407 |
| Prevalence | Mozambique | Vitamin A deficiency | 28865.43 | 33338.42 | 24964.81 |
| Prevalence | Mozambique | Dietary iron deficiency | 20609.54 | 22543.77 | 18843.03 |
| Prevalence | Guatemala | Iodine deficiency | 553.4027 | 703.5135 | 419.4232 |
| Prevalence | Guatemala | Vitamin A deficiency | 5880.068 | 7117.035 | 4913.624 |
| Prevalence | Sudan | Iodine deficiency | 1405.358 | 1723.622 | 1098.064 |
| Prevalence | Sudan | Vitamin A deficiency | 9067.546 | 11071.48 | 7431.238 |
| Prevalence | Guatemala | Dietary iron deficiency | 10966.73 | 12389.72 | 9632.826 |
| Prevalence | Sudan | Dietary iron deficiency | 13325.28 | 15016.85 | 11781.9 |
| Prevalence | Myanmar | Iodine deficiency | 444.5893 | 570.8185 | 342.9208 |
| Prevalence | Myanmar | Vitamin A deficiency | 5309.209 | 6548.45 | 4283.055 |
| Prevalence | Indonesia | Iodine deficiency | 328.5843 | 425.3142 | 253.6155 |
| Prevalence | Monaco | Iodine deficiency | 687.085 | 874.6105 | 530.3325 |
| Prevalence | Monaco | Vitamin A deficiency | 301.5993 | 367.065 | 249.0219 |
| Prevalence | Monaco | Dietary iron deficiency | 2356.716 | 3212.703 | 1715.808 |
| Prevalence | Belarus | Iodine deficiency | 131.2207 | 169.7529 | 95.05305 |
| Prevalence | Belarus | Vitamin A deficiency | 1286.971 | 1503.087 | 1099.852 |
| Prevalence | Indonesia | Vitamin A deficiency | 5770.57 | 6863.662 | 4762.1 |
| Prevalence | Indonesia | Dietary iron deficiency | 14101.47 | 15334.24 | 12926.56 |
| Prevalence | Estonia | Vitamin A deficiency | 713.553 | 827.2051 | 606.5046 |
| Prevalence | Estonia | Dietary iron deficiency | 6646.954 | 7997.466 | 5529.525 |
| Prevalence | Tonga | Iodine deficiency | 56.97352 | 76.08175 | 40.30629 |
| Prevalence | Tonga | Vitamin A deficiency | 12528.03 | 15246.97 | 10191.31 |
| Prevalence | Tonga | Dietary iron deficiency | 16938.95 | 18934.12 | 15127.77 |
| Prevalence | Somalia | Iodine deficiency | 21101.21 | 24959.65 | 17356.23 |
| Prevalence | Mali | Iodine deficiency | 897.2298 | 1153.915 | 703.0866 |
| Prevalence | Mali | Vitamin A deficiency | 30561.22 | 35008.25 | 26304.47 |
| Prevalence | Mali | Dietary iron deficiency | 27485.76 | 30354.15 | 24172.11 |
| Prevalence | Somalia | Vitamin A deficiency | 63640.11 | 67657.56 | 59279.85 |
| Prevalence | Gabon | Iodine deficiency | 2637.404 | 3294.613 | 2079.477 |
| Prevalence | Gabon | Vitamin A deficiency | 4611.673 | 5688.785 | 3770.01 |
| Prevalence | Gabon | Dietary iron deficiency | 22933.11 | 26009.28 | 19482.89 |
| Prevalence | Bahamas | Iodine deficiency | 148.0694 | 189.8346 | 111.4039 |
| Prevalence | Bahamas | Vitamin A deficiency | 1434.223 | 1682.365 | 1203.767 |
| Prevalence | Bahamas | Dietary iron deficiency | 15101.18 | 16929.96 | 13136.2 |
| Prevalence | Iceland | Iodine deficiency | 306.0991 | 391.2192 | 237.4376 |
| Prevalence | Iceland | Vitamin A deficiency | 500.9516 | 608.7013 | 414.122 |
| Prevalence | Iceland | Dietary iron deficiency | 2195.15 | 2954.877 | 1596.429 |
| Prevalence | Tajikistan | Iodine deficiency | 632.0973 | 808.4513 | 482.8595 |
| Prevalence | Tajikistan | Vitamin A deficiency | 8084.766 | 9556.331 | 6676.167 |
| Prevalence | Tajikistan | Dietary iron deficiency | 15077.82 | 16628.74 | 13448.34 |
| Prevalence | United Republic of Tanzania | Vitamin A deficiency | 13929.63 | 16214.66 | 11912.06 |
| Prevalence | Austria | Iodine deficiency | 761.3145 | 975.1708 | 596.9444 |
| Prevalence | Austria | Vitamin A deficiency | 565.0928 | 691.1856 | 470.3827 |
| Prevalence | Austria | Dietary iron deficiency | 2575.047 | 3442.351 | 1902.669 |
| Prevalence | Haiti | Iodine deficiency | 1224.301 | 1556.417 | 928.1655 |
| Prevalence | Haiti | Vitamin A deficiency | 13512.71 | 15853.4 | 11514.77 |
| Prevalence | Haiti | Dietary iron deficiency | 24490.18 | 26557.57 | 22593.4 |
| Prevalence | United Republic of Tanzania | Dietary iron deficiency | 23550.61 | 25548.48 | 21580.04 |
| Prevalence | Jordan | Iodine deficiency | 536.0547 | 666.8843 | 417.7198 |
| Prevalence | Jordan | Vitamin A deficiency | 5045.489 | 6131.246 | 4187.364 |
| Prevalence | Jordan | Dietary iron deficiency | 8638.355 | 9739.054 | 7580.141 |
| Prevalence | Algeria | Iodine deficiency | 569.8339 | 712.7148 | 451.315 |
| Prevalence | Algeria | Vitamin A deficiency | 2690.399 | 3276.271 | 2223.701 |
| Prevalence | Algeria | Dietary iron deficiency | 9127.203 | 10734.75 | 7624.494 |
| Prevalence | Palau | Iodine deficiency | 47.60842 | 63.82802 | 33.64617 |
| Prevalence | Russian Federation | Iodine deficiency | 167.9763 | 216.9627 | 124.744 |
| Prevalence | Russian Federation | Vitamin A deficiency | 199.5045 | 233.0428 | 169.9333 |
| Prevalence | Russian Federation | Dietary iron deficiency | 7321.575 | 8493.235 | 6274.005 |
| Prevalence | Puerto Rico | Iodine deficiency | 132.8601 | 172.5758 | 99.96117 |
| Prevalence | Puerto Rico | Vitamin A deficiency | 1204.482 | 1419.383 | 1024.807 |
| Prevalence | Puerto Rico | Dietary iron deficiency | 9316.522 | 10981.72 | 7759.654 |
| Prevalence | Dominica | Iodine deficiency | 281.2058 | 361.5841 | 214.6411 |
| Prevalence | Dominica | Vitamin A deficiency | 2129.113 | 2528.768 | 1789.019 |
| Prevalence | Suriname | Iodine deficiency | 232.0531 | 298.4611 | 176.3594 |
| Prevalence | Suriname | Vitamin A deficiency | 4317.255 | 5090.376 | 3567.129 |
| Prevalence | Suriname | Dietary iron deficiency | 16606.39 | 18606.63 | 14699.68 |
| Prevalence | Senegal | Iodine deficiency | 1787.183 | 2289.325 | 1372.533 |
| Prevalence | Senegal | Vitamin A deficiency | 14427.53 | 17808.84 | 11765.99 |
| Prevalence | Senegal | Dietary iron deficiency | 26043.82 | 27830.62 | 24175.18 |
| Prevalence | Ukraine | Vitamin A deficiency | 1357.395 | 1590.471 | 1158.006 |
| Prevalence | Ukraine | Dietary iron deficiency | 5857.986 | 6936.916 | 4937.021 |
| Prevalence | Malta | Iodine deficiency | 795.3882 | 1018.29 | 617.6028 |
| Prevalence | Malta | Vitamin A deficiency | 759.6067 | 942.0992 | 627.821 |
| Prevalence | Malta | Dietary iron deficiency | 3254.337 | 4225.537 | 2478.629 |
| Prevalence | Eritrea | Iodine deficiency | 541.9657 | 683.3233 | 424.8916 |
| Prevalence | Eritrea | Vitamin A deficiency | 22128.39 | 25563.98 | 18798.03 |
| Prevalence | Eritrea | Dietary iron deficiency | 22667.21 | 24728.45 | 20680.57 |
| Prevalence | Botswana | Iodine deficiency | 296.9685 | 373.4901 | 230.7627 |
| Prevalence | Botswana | Vitamin A deficiency | 9632.487 | 11592.91 | 7920.46 |
| Prevalence | Botswana | Dietary iron deficiency | 12523.89 | 14333.58 | 10887.85 |
| Prevalence | Morocco | Iodine deficiency | 1520.097 | 1862.297 | 1191.137 |
| Prevalence | Morocco | Vitamin A deficiency | 6117.33 | 7318.736 | 5055.529 |
| Prevalence | Morocco | Dietary iron deficiency | 11329.74 | 13061.66 | 9825.327 |
| Prevalence | Bosnia and Herzegovina | Iodine deficiency | 388.7829 | 496.4307 | 293.2947 |
| Prevalence | Bosnia and Herzegovina | Vitamin A deficiency | 8900.141 | 10266.41 | 7662.504 |
| Prevalence | Bosnia and Herzegovina | Dietary iron deficiency | 9986.017 | 11724.49 | 8313.492 |
| Prevalence | Tuvalu | Iodine deficiency | 64.22381 | 85.40138 | 46.36883 |
| Prevalence | Tuvalu | Vitamin A deficiency | 14979.88 | 17994.06 | 12572.78 |
| Prevalence | Tuvalu | Dietary iron deficiency | 19421.37 | 21512.54 | 17404.84 |
| Prevalence | South Sudan | Iodine deficiency | 1796.909 | 2284.291 | 1412.97 |
| Prevalence | South Sudan | Vitamin A deficiency | 20173.61 | 23709.22 | 17093.99 |
| Prevalence | South Sudan | Dietary iron deficiency | 21615.58 | 24343.38 | 18918.02 |
| Prevalence | Hungary | Iodine deficiency | 195.7673 | 251.7499 | 145.7608 |
| Prevalence | Hungary | Vitamin A deficiency | 6651.651 | 7631.892 | 5727.188 |
| Prevalence | Hungary | Dietary iron deficiency | 7134.309 | 8573.125 | 5794.608 |
| Prevalence | Mauritius | Iodine deficiency | 353.6409 | 458.1586 | 279.1479 |
| Prevalence | Mauritius | Vitamin A deficiency | 1311.458 | 1609.531 | 1059.559 |
| Prevalence | Mauritius | Dietary iron deficiency | 11853.46 | 13624.16 | 10203.85 |
| Prevalence | Zimbabwe | Iodine deficiency | 2987.818 | 3844.993 | 2316.828 |
| Prevalence | Zimbabwe | Vitamin A deficiency | 19669.56 | 22884.43 | 16646.11 |
| Prevalence | Zimbabwe | Dietary iron deficiency | 15572.13 | 17034.85 | 14138.77 |
| Prevalence | Guam | Iodine deficiency | 44.38991 | 59.804 | 30.95505 |
| Prevalence | Guam | Vitamin A deficiency | 3113.348 | 3874.458 | 2469.01 |
| Prevalence | Guam | Dietary iron deficiency | 14772.3 | 16724.89 | 12946.79 |
| Prevalence | Syrian Arab Republic | Vitamin A deficiency | 3722.355 | 4442.218 | 3067.316 |
| Prevalence | Syrian Arab Republic | Dietary iron deficiency | 9756.263 | 11420.54 | 8304.995 |
| Prevalence | Syrian Arab Republic | Iodine deficiency | 1294.621 | 1597.408 | 1014.134 |
| Prevalence | Serbia | Iodine deficiency | 137.4846 | 178.2831 | 100.6876 |
| Prevalence | Serbia | Vitamin A deficiency | 15871.77 | 17944.52 | 13945.39 |
| Prevalence | Serbia | Dietary iron deficiency | 8944.322 | 10603.31 | 7433.22 |
| Prevalence | Cabo Verde | Iodine deficiency | 1622.807 | 2072.977 | 1266.469 |
| Prevalence | Cabo Verde | Vitamin A deficiency | 5248.875 | 6489.234 | 4294.078 |
| Prevalence | Cabo Verde | Dietary iron deficiency | 17693.2 | 19682.11 | 15520.1 |
| Prevalence | Yemen | Iodine deficiency | 2901 | 3613.174 | 2292.598 |
| Prevalence | Yemen | Vitamin A deficiency | 15059.25 | 17887.4 | 12423.07 |
| Prevalence | Yemen | Dietary iron deficiency | 21985.92 | 23476.72 | 20449.79 |
| Prevalence | Sweden | Iodine deficiency | 798.1051 | 1026.269 | 620.6911 |
| Prevalence | Sweden | Vitamin A deficiency | 507.8366 | 622.633 | 415.4368 |
| Prevalence | Sweden | Dietary iron deficiency | 2814.062 | 3681.615 | 2026.445 |
| Prevalence | Greenland | Iodine deficiency | 212.6039 | 271.7189 | 161.5407 |
| Prevalence | Greenland | Vitamin A deficiency | 977.1553 | 1206.444 | 797.9537 |
| Prevalence | Greenland | Dietary iron deficiency | 7085.434 | 8384.968 | 5884.331 |
| Prevalence | Philippines | Iodine deficiency | 2355.95 | 2946.197 | 1848.425 |
| Prevalence | Philippines | Vitamin A deficiency | 9309.22 | 11135.15 | 7668.954 |
| Prevalence | Philippines | Dietary iron deficiency | 9712.318 | 10852.48 | 8595.133 |
| Prevalence | Bhutan | Iodine deficiency | 557.114 | 705.6865 | 436.5166 |
| Prevalence | Bhutan | Vitamin A deficiency | 5858.628 | 6905.898 | 4894.756 |
| Prevalence | Bhutan | Dietary iron deficiency | 32085.69 | 34028.13 | 30283.81 |
| Prevalence | Viet Nam | Iodine deficiency | 729.5449 | 921.9113 | 567.7602 |
| Prevalence | Viet Nam | Vitamin A deficiency | 1674.852 | 2240.9 | 1280.325 |
| Prevalence | Viet Nam | Dietary iron deficiency | 8397.228 | 9621.938 | 7362.076 |
| Prevalence | Marshall Islands | Iodine deficiency | 64.75921 | 87.3804 | 46.779 |
| Prevalence | Marshall Islands | Vitamin A deficiency | 22663.67 | 26326.44 | 19353.74 |
| Prevalence | Marshall Islands | Dietary iron deficiency | 19878.52 | 21831.61 | 17916.62 |
| Prevalence | Belarus | Dietary iron deficiency | 7325.56 | 8648.41 | 6114.813 |
| Prevalence | Iraq | Iodine deficiency | 1150.894 | 1417.587 | 903.5875 |
| Prevalence | Iraq | Vitamin A deficiency | 3209.164 | 3937.858 | 2619.096 |
| Prevalence | Iraq | Dietary iron deficiency | 8501.986 | 9931.247 | 7289.229 |
| Prevalence | Azerbaijan | Iodine deficiency | 206.8495 | 265.8923 | 152.7834 |
| Prevalence | Azerbaijan | Vitamin A deficiency | 2574.202 | 3099.158 | 2141.14 |
| Prevalence | Azerbaijan | Dietary iron deficiency | 14503.06 | 16028.24 | 13155.23 |
| Prevalence | Liberia | Iodine deficiency | 741.116 | 937.7911 | 580.8915 |
| Prevalence | Liberia | Vitamin A deficiency | 13461.13 | 16479.8 | 10787.56 |
| Prevalence | Liberia | Dietary iron deficiency | 14246.67 | 16527.42 | 11853.6 |
| Prevalence | Kiribati | Iodine deficiency | 81.98188 | 108.2532 | 59.47744 |
| Prevalence | Bermuda | Iodine deficiency | 108.9312 | 142.2946 | 81.05474 |
| Prevalence | Solomon Islands | Iodine deficiency | 63.88097 | 84.75695 | 45.44249 |
| Prevalence | Solomon Islands | Vitamin A deficiency | 27989.22 | 32287.78 | 23742.88 |
| Prevalence | Solomon Islands | Dietary iron deficiency | 19859.43 | 21846.82 | 17973.61 |
| Prevalence | Kiribati | Vitamin A deficiency | 26227.13 | 30289.12 | 22408.78 |
| Prevalence | Kiribati | Dietary iron deficiency | 23509.57 | 25452.54 | 21620.1 |
| Prevalence | Libya | Iodine deficiency | 1117.121 | 1377.849 | 876.6924 |
| Prevalence | Libya | Vitamin A deficiency | 1829.158 | 2213.545 | 1515.347 |
| Prevalence | Libya | Dietary iron deficiency | 9597.79 | 11137.64 | 8046.836 |
| Prevalence | Mongolia | Iodine deficiency | 324.9986 | 418.0255 | 245.8539 |
| Prevalence | Mongolia | Vitamin A deficiency | 1242.084 | 1511.345 | 1020.407 |
| Prevalence | Mongolia | Dietary iron deficiency | 13500.53 | 14911.4 | 12066.16 |
| Prevalence | Republic of Moldova | Iodine deficiency | 85.11684 | 111.3876 | 61.78416 |
| Prevalence | Republic of Moldova | Vitamin A deficiency | 1857.994 | 2177.424 | 1571.52 |
| Prevalence | Republic of Moldova | Dietary iron deficiency | 11592.6 | 12919.06 | 10297.47 |
| Prevalence | Croatia | Iodine deficiency | 177.265 | 227.6429 | 132.2718 |
| Prevalence | Sao Tome and Principe | Iodine deficiency | 852.853 | 1075.142 | 671.7865 |
| Prevalence | Sao Tome and Principe | Vitamin A deficiency | 13261.5 | 16156.53 | 10897.33 |
| Prevalence | Sao Tome and Principe | Dietary iron deficiency | 19107.01 | 21295.91 | 17034.29 |
| Prevalence | Croatia | Vitamin A deficiency | 7409.325 | 8556.062 | 6414.407 |
| Prevalence | Croatia | Dietary iron deficiency | 6406.559 | 7838.531 | 5111.937 |
| Prevalence | Qatar | Iodine deficiency | 295.1486 | 375.094 | 227.9049 |
| Prevalence | Saudi Arabia | Iodine deficiency | 139.2894 | 176.4572 | 106.1915 |
| Prevalence | Saudi Arabia | Vitamin A deficiency | 190.0431 | 229.044 | 156.4873 |
| Prevalence | Saudi Arabia | Dietary iron deficiency | 4493.913 | 5604.066 | 3506.398 |
| Prevalence | Samoa | Iodine deficiency | 40.5493 | 54.92467 | 27.88135 |
| Prevalence | Samoa | Vitamin A deficiency | 17957.84 | 21397.02 | 14963.12 |
| Prevalence | Samoa | Dietary iron deficiency | 13441.47 | 15256.61 | 11766.96 |
| Prevalence | Albania | Iodine deficiency | 187.0873 | 240.6359 | 139.6361 |
| Prevalence | Albania | Vitamin A deficiency | 11731.87 | 13478.61 | 10138.25 |
| Prevalence | Albania | Dietary iron deficiency | 10512.28 | 11658.55 | 9558.747 |
| Prevalence | Montenegro | Iodine deficiency | 183.9568 | 236.303 | 137.6446 |
| Prevalence | Montenegro | Vitamin A deficiency | 6333.41 | 7256.333 | 5458.873 |
| Prevalence | Montenegro | Dietary iron deficiency | 8364.958 | 9973.232 | 6992.067 |
| Prevalence | Denmark | Iodine deficiency | 525.6025 | 667.8172 | 411.4508 |
| Prevalence | Denmark | Vitamin A deficiency | 439.5851 | 533.7205 | 359.8166 |
| Prevalence | Denmark | Dietary iron deficiency | 3013.847 | 4127.401 | 2190.436 |
| Prevalence | Cook Islands | Iodine deficiency | 46.4234 | 62.44029 | 32.11037 |
| Prevalence | Cook Islands | Vitamin A deficiency | 3858.086 | 4773.769 | 3068.388 |
| Prevalence | Ghana | Iodine deficiency | 2224.086 | 2871.699 | 1729.661 |
| Prevalence | Ghana | Vitamin A deficiency | 15915.73 | 18795.72 | 13097.57 |
| Prevalence | Ghana | Dietary iron deficiency | 19509.18 | 21742.43 | 17419.48 |
| Prevalence | Armenia | Iodine deficiency | 1021.219 | 1268.052 | 789.8999 |
| Prevalence | Armenia | Vitamin A deficiency | 494.5752 | 579.7326 | 414.769 |
| Prevalence | Armenia | Dietary iron deficiency | 11122.42 | 12543.9 | 9854.02 |
| Prevalence | Switzerland | Iodine deficiency | 534.2722 | 682.9667 | 415.4744 |
| Prevalence | Switzerland | Vitamin A deficiency | 322.8196 | 396.447 | 264.3923 |
| Prevalence | Switzerland | Dietary iron deficiency | 2349.121 | 3166.286 | 1705.073 |
| Prevalence | Slovenia | Iodine deficiency | 171.0607 | 220.8366 | 126.9698 |
| Prevalence | Slovenia | Vitamin A deficiency | 4867.492 | 5622.748 | 4198.266 |
| Prevalence | Slovenia | Dietary iron deficiency | 6303.042 | 7623.93 | 5139.34 |
| Prevalence | Ecuador | Iodine deficiency | 76.23785 | 102.884 | 56.25668 |
| Prevalence | Ecuador | Vitamin A deficiency | 3770.762 | 4652.233 | 3036.987 |
| Prevalence | Ecuador | Dietary iron deficiency | 5041.368 | 5762.663 | 4369.106 |
| Prevalence | Greece | Iodine deficiency | 792.7871 | 1000.808 | 617.0084 |
| Prevalence | Greece | Vitamin A deficiency | 762.9608 | 932.8873 | 630.8775 |
| Prevalence | Greece | Dietary iron deficiency | 3083.438 | 4048.105 | 2307.397 |
| Prevalence | Uruguay | Iodine deficiency | 143.3552 | 183.6394 | 105.699 |
| Prevalence | Uruguay | Vitamin A deficiency | 5121.545 | 6222.503 | 4190.822 |
| Prevalence | Uruguay | Dietary iron deficiency | 7980.098 | 9564.692 | 6589.406 |
| Prevalence | Luxembourg | Iodine deficiency | 723.5373 | 913.3056 | 564.6019 |
| Prevalence | Luxembourg | Vitamin A deficiency | 408.1687 | 494.7124 | 331.0745 |
| Prevalence | Luxembourg | Dietary iron deficiency | 2491.294 | 3329.438 | 1830.909 |
| Prevalence | El Salvador | Iodine deficiency | 570.1394 | 721.6363 | 434.7394 |
| Prevalence | El Salvador | Vitamin A deficiency | 5828.645 | 6986.558 | 4813.197 |
| Prevalence | El Salvador | Dietary iron deficiency | 7697.57 | 8941.868 | 6506.96 |
| Prevalence | Kyrgyzstan | Iodine deficiency | 313.0028 | 404.4918 | 234.312 |
| Prevalence | Kyrgyzstan | Vitamin A deficiency | 4423.488 | 5355.656 | 3637.697 |
| Prevalence | Kyrgyzstan | Dietary iron deficiency | 16530.75 | 18291.22 | 14850.73 |
| Prevalence | Niger | Iodine deficiency | 3186.871 | 4100.202 | 2504.84 |
| Prevalence | Niger | Vitamin A deficiency | 43501.51 | 48083.42 | 38967.65 |
| Prevalence | Niger | Dietary iron deficiency | 20408.48 | 22734.71 | 18052.43 |
| Prevalence | Lebanon | Iodine deficiency | 1313.528 | 1625.775 | 1025.367 |
| Prevalence | Lebanon | Vitamin A deficiency | 1682.653 | 2043.834 | 1399.464 |
| Prevalence | Lebanon | Dietary iron deficiency | 5131.112 | 6372.566 | 4008.659 |
| Prevalence | Nigeria | Iodine deficiency | 1174.364 | 1501.956 | 917.9942 |
| Prevalence | Nigeria | Vitamin A deficiency | 5128.399 | 6114.312 | 4206.235 |
| Prevalence | Nigeria | Dietary iron deficiency | 21690.05 | 23582.46 | 19833.4 |
| Prevalence | Panama | Iodine deficiency | 258.4526 | 333.3402 | 198.36 |
| Prevalence | Panama | Vitamin A deficiency | 2811.884 | 3337.038 | 2339.63 |
| Prevalence | Panama | Dietary iron deficiency | 10093.91 | 11699.97 | 8676.501 |
| Prevalence | Uzbekistan | Iodine deficiency | 557.7951 | 718.2504 | 427.4786 |
| Prevalence | Uzbekistan | Vitamin A deficiency | 2979.091 | 3597.284 | 2484.751 |
| Prevalence | Malaysia | Iodine deficiency | 837.098 | 1064.249 | 658.6135 |
| Prevalence | Malaysia | Vitamin A deficiency | 546.7082 | 669.5191 | 445.2848 |
| Prevalence | Malaysia | Dietary iron deficiency | 13391.09 | 15011.57 | 11796.16 |
| Prevalence | Uzbekistan | Dietary iron deficiency | 22440.15 | 24582.24 | 20406.98 |
| Prevalence | Spain | Iodine deficiency | 767.8158 | 990.4008 | 595.4011 |
| Prevalence | Spain | Vitamin A deficiency | 667.8607 | 800.2035 | 566.1579 |
| Prevalence | Spain | Dietary iron deficiency | 4002.882 | 5224.775 | 3017.4 |
| Prevalence | Turkey | Iodine deficiency | 1059.586 | 1316.837 | 824.5131 |
| Prevalence | Turkey | Vitamin A deficiency | 3184.302 | 3724.763 | 2713.447 |
| Prevalence | Turkey | Dietary iron deficiency | 8521.483 | 9985.685 | 7185.79 |
| Prevalence | Oman | Iodine deficiency | 359.9646 | 449.2952 | 282.9064 |
| Prevalence | Oman | Vitamin A deficiency | 1300.799 | 1633.511 | 1050.088 |
| Prevalence | Guyana | Iodine deficiency | 485.1068 | 621.4809 | 364.0685 |
| Prevalence | Guyana | Vitamin A deficiency | 4953.899 | 5881.248 | 4161.422 |
| Prevalence | Guyana | Dietary iron deficiency | 20865.02 | 22718.72 | 19053.64 |
| Prevalence | Comoros | Iodine deficiency | 408.0702 | 512.8373 | 320.9879 |
| Prevalence | Comoros | Vitamin A deficiency | 19533.69 | 22517.14 | 16776.57 |
| Prevalence | Comoros | Dietary iron deficiency | 23303.99 | 25852.9 | 19995.8 |
| Prevalence | Qatar | Vitamin A deficiency | 725.7576 | 927.3606 | 575.058 |
| Prevalence | Qatar | Dietary iron deficiency | 5608.836 | 6985.341 | 4396.114 |
| Prevalence | Togo | Iodine deficiency | 2222.012 | 2823.575 | 1716.534 |
| Prevalence | Togo | Vitamin A deficiency | 18885.65 | 22054.03 | 15809.46 |
| Prevalence | Togo | Dietary iron deficiency | 20371.36 | 21906.09 | 18911.87 |
| Prevalence | Cameroon | Iodine deficiency | 768.6328 | 988.0913 | 602.2196 |
| Prevalence | Cameroon | Vitamin A deficiency | 27949.82 | 31961.96 | 23794.23 |
| Prevalence | Cameroon | Dietary iron deficiency | 15307.58 | 17236.52 | 13454.53 |
| Prevalence | Somalia | Dietary iron deficiency | 24814.25 | 26711.34 | 22865.12 |
| Prevalence | Thailand | Iodine deficiency | 473.3847 | 602.3162 | 374.7849 |
| Prevalence | Thailand | Vitamin A deficiency | 1594.68 | 1918.043 | 1341.931 |
| Prevalence | Thailand | Dietary iron deficiency | 8066.546 | 9462.12 | 6804.004 |
| Prevalence | Chile | Iodine deficiency | 139.9079 | 180.4375 | 103.071 |
| Prevalence | Chile | Vitamin A deficiency | 4106.518 | 4908.798 | 3404.172 |
| Prevalence | Chile | Dietary iron deficiency | 3074.296 | 3788.461 | 2460.838 |
| Prevalence | Saint Vincent and the Grenadines | Iodine deficiency | 261.0298 | 340.8816 | 197.789 |
| Prevalence | Saint Vincent and the Grenadines | Vitamin A deficiency | 3982.819 | 4724.69 | 3363.278 |
| Prevalence | Saint Vincent and the Grenadines | Dietary iron deficiency | 17025.39 | 19152.94 | 15127.91 |
| Prevalence | Djibouti | Iodine deficiency | 13294.98 | 16390.99 | 10703.67 |
| Prevalence | Djibouti | Vitamin A deficiency | 16576.49 | 19322.85 | 14006.6 |
| Prevalence | Djibouti | Dietary iron deficiency | 20868.15 | 23231.29 | 18408.79 |
| Prevalence | Afghanistan | Iodine deficiency | 1637.28 | 2008.711 | 1290.137 |
| Prevalence | Afghanistan | Vitamin A deficiency | 26013.77 | 30088.73 | 22250.86 |
| Prevalence | Afghanistan | Dietary iron deficiency | 9243.22 | 10311.8 | 8210.838 |
| Prevalence | Madagascar | Iodine deficiency | 2312.746 | 2926.525 | 1810.236 |
| Prevalence | Madagascar | Vitamin A deficiency | 21864.71 | 25124.57 | 18803.93 |
| Prevalence | Madagascar | Dietary iron deficiency | 20898.21 | 22858.31 | 18888.3 |
| Prevalence | Northern Mariana Islands | Iodine deficiency | 45.7013 | 61.43732 | 32.16095 |
| Prevalence | Northern Mariana Islands | Vitamin A deficiency | 4083.038 | 5089.132 | 3267.317 |
| Prevalence | Palau | Vitamin A deficiency | 5160.581 | 6343.552 | 4154.474 |
| Prevalence | Palau | Dietary iron deficiency | 13955.41 | 15987.2 | 12184.14 |
| Prevalence | Gambia | Iodine deficiency | 3547.642 | 4527.697 | 2819.68 |
| Prevalence | Gambia | Vitamin A deficiency | 22716.8 | 26722.24 | 19313.06 |
| Prevalence | Gambia | Dietary iron deficiency | 26027.32 | 27944.03 | 24103.64 |
| Prevalence | Rwanda | Iodine deficiency | 3037.938 | 3885.674 | 2371.665 |
| Prevalence | Rwanda | Vitamin A deficiency | 14592.39 | 17073.35 | 12290.56 |
| Prevalence | Rwanda | Dietary iron deficiency | 14664.84 | 16556.79 | 12561.05 |
| Prevalence | Malawi | Iodine deficiency | 2736.656 | 3493.672 | 2123.219 |
| Prevalence | Malawi | Vitamin A deficiency | 25987.25 | 29970.98 | 22397.78 |
| Prevalence | Malawi | Dietary iron deficiency | 22351.79 | 24294.14 | 20502.99 |
| Prevalence | Eswatini | Iodine deficiency | 937.2392 | 1209.937 | 725.2878 |
| Prevalence | Eswatini | Vitamin A deficiency | 8688.369 | 10730 | 7026.227 |
| Prevalence | Eswatini | Dietary iron deficiency | 11682.58 | 13180.32 | 10403.2 |
| Prevalence | Tokelau | Iodine deficiency | 52.90396 | 71.43047 | 37.25636 |
| Prevalence | Tokelau | Vitamin A deficiency | 10190.53 | 12598.05 | 8207.301 |
| Prevalence | Tokelau | Dietary iron deficiency | 16702.91 | 18690.08 | 14779.77 |
| Prevalence | Seychelles | Iodine deficiency | 302.0276 | 385.6715 | 236.8855 |
| Prevalence | Seychelles | Vitamin A deficiency | 1370.172 | 1707.768 | 1121.33 |
| Prevalence | Seychelles | Dietary iron deficiency | 11386.75 | 13259.85 | 9871.861 |
| Prevalence | Zambia | Iodine deficiency | 1146.676 | 1466.959 | 898.5897 |
| Prevalence | Zambia | Vitamin A deficiency | 13680.37 | 16283.86 | 11466.73 |
| Prevalence | Zambia | Dietary iron deficiency | 29783.78 | 31629.56 | 27773.93 |
| Prevalence | Bermuda | Vitamin A deficiency | 1210.927 | 1416.254 | 1037.319 |
| Prevalence | Bermuda | Dietary iron deficiency | 8401.243 | 10002.52 | 6970.381 |
| Prevalence | Namibia | Iodine deficiency | 592.4947 | 748.4143 | 463.8092 |
| Prevalence | Namibia | Vitamin A deficiency | 6569.442 | 7995.872 | 5370.48 |
| Prevalence | Namibia | Dietary iron deficiency | 12426.68 | 13761.65 | 10418.66 |
| Prevalence | South Africa | Iodine deficiency | 591.5468 | 747.3705 | 458.5449 |
| Prevalence | South Africa | Vitamin A deficiency | 4066.682 | 4986.806 | 3287.257 |
| Prevalence | South Africa | Dietary iron deficiency | 10142.78 | 11166.91 | 9253.375 |
| Prevalence | Lithuania | Iodine deficiency | 88.74062 | 116.4942 | 64.34154 |
| Prevalence | Lithuania | Vitamin A deficiency | 681.8058 | 800.9652 | 582.9476 |
| Prevalence | Lithuania | Dietary iron deficiency | 8204.495 | 9628.381 | 6868.987 |
| Prevalence | Paraguay | Iodine deficiency | 114.9279 | 149.2962 | 86.25961 |
| Prevalence | Paraguay | Vitamin A deficiency | 6379.223 | 7614.219 | 5318.967 |
| Prevalence | Paraguay | Dietary iron deficiency | 11620.03 | 13340.13 | 10146.61 |
| Prevalence | Cambodia | Iodine deficiency | 694.9249 | 885.4495 | 539.6592 |
| Prevalence | Cambodia | Vitamin A deficiency | 7755.021 | 9471.093 | 6235.502 |
| Prevalence | Cambodia | Dietary iron deficiency | 19740.23 | 21691.94 | 17839.64 |
| Prevalence | Cook Islands | Dietary iron deficiency | 13068.48 | 15236.21 | 11306.04 |
| Prevalence | Taiwan (Province of China) | Iodine deficiency | 89.85728 | 118.6816 | 66.12629 |
| Prevalence | Taiwan (Province of China) | Vitamin A deficiency | 647.0691 | 785.2294 | 531.9855 |
| Prevalence | Taiwan (Province of China) | Dietary iron deficiency | 5493.52 | 6564.943 | 4494.498 |
| Prevalence | Egypt | Iodine deficiency | 713.064 | 885.762 | 558.2078 |
| Prevalence | Egypt | Vitamin A deficiency | 1925.178 | 2320.363 | 1590.418 |
| Prevalence | Brunei Darussalam | Iodine deficiency | 239.8235 | 304.8773 | 183.1107 |
| Prevalence | Brunei Darussalam | Vitamin A deficiency | 1112.336 | 1358.553 | 898.5916 |
| Prevalence | Brunei Darussalam | Dietary iron deficiency | 10311.22 | 11983.35 | 8898.802 |
| Prevalence | Iran (Islamic Republic of) | Iodine deficiency | 285.1196 | 362.0154 | 220.1468 |
| Prevalence | Iran (Islamic Republic of) | Vitamin A deficiency | 1344.704 | 1651.792 | 1118.861 |
| Prevalence | Iran (Islamic Republic of) | Dietary iron deficiency | 5939.144 | 6956.517 | 4983.072 |

**Supplementary Table 4. Age-standarised prevalence, incidence, and DALYs rate of iodine deficiency, vitamin A deficiency, and dietary iron deficiency globally and for 21 GBD regions, 1990-2019.**

| Measure | Location | Micronutrient deficiency | Year | Rate | 95% Upper UI | 95% Lower UI |
| --- | --- | --- | --- | --- | --- | --- |
| DALYs | Andean Latin America | Dietary iron deficiency | 1990 | 487.497 | 715.7501 | 319.5288 |
| DALYs | Andean Latin America | Dietary iron deficiency | 1991 | 480.1477 | 708.0532 | 317.0087 |
| DALYs | Andean Latin America | Dietary iron deficiency | 1992 | 471.2961 | 693.0166 | 309.963 |
| DALYs | Andean Latin America | Dietary iron deficiency | 1993 | 461.534 | 676.7529 | 303.3084 |
| DALYs | Andean Latin America | Dietary iron deficiency | 1994 | 450.9056 | 657.7085 | 296.1983 |
| DALYs | Andean Latin America | Dietary iron deficiency | 1995 | 440.0031 | 644.7282 | 289.0268 |
| DALYs | Andean Latin America | Dietary iron deficiency | 1996 | 426.3484 | 627.3894 | 281.1942 |
| DALYs | Andean Latin America | Dietary iron deficiency | 1997 | 409.2109 | 603.5254 | 269.9233 |
| DALYs | Andean Latin America | Dietary iron deficiency | 1998 | 390.8133 | 572.6724 | 257.9113 |
| DALYs | Andean Latin America | Dietary iron deficiency | 1999 | 373.6217 | 549.1371 | 246.4401 |
| DALYs | Andean Latin America | Dietary iron deficiency | 2000 | 360.0237 | 531.7639 | 237.0258 |
| DALYs | Andean Latin America | Dietary iron deficiency | 2001 | 349.5022 | 515.2988 | 229.6064 |
| DALYs | Andean Latin America | Dietary iron deficiency | 2002 | 339.4605 | 498.4613 | 223.5511 |
| DALYs | Andean Latin America | Dietary iron deficiency | 2003 | 330.0619 | 487.1525 | 216.6158 |
| DALYs | Andean Latin America | Dietary iron deficiency | 2004 | 321.2259 | 473.7185 | 209.1526 |
| DALYs | Andean Latin America | Dietary iron deficiency | 2005 | 312.8085 | 460.2032 | 202.3371 |
| DALYs | Andean Latin America | Dietary iron deficiency | 2006 | 303.9703 | 447.3228 | 195.6207 |
| DALYs | Andean Latin America | Dietary iron deficiency | 2007 | 294.4493 | 433.2084 | 191.9172 |
| DALYs | Andean Latin America | Dietary iron deficiency | 2008 | 284.9808 | 420.5823 | 186.6023 |
| DALYs | Andean Latin America | Dietary iron deficiency | 2009 | 276.247 | 405.5069 | 180.0204 |
| DALYs | Andean Latin America | Dietary iron deficiency | 2010 | 269.0553 | 397.0201 | 176.1181 |
| DALYs | Andean Latin America | Dietary iron deficiency | 2011 | 262.3643 | 386.5754 | 171.2335 |
| DALYs | Andean Latin America | Dietary iron deficiency | 2012 | 255.1472 | 376.2953 | 166.5887 |
| DALYs | Andean Latin America | Dietary iron deficiency | 2013 | 248.0533 | 366.0991 | 161.4802 |
| DALYs | Andean Latin America | Dietary iron deficiency | 2014 | 241.6599 | 355.863 | 156.5584 |
| DALYs | Andean Latin America | Dietary iron deficiency | 2015 | 236.6022 | 351.7401 | 151.573 |
| DALYs | Andean Latin America | Dietary iron deficiency | 2016 | 230.9752 | 343.24 | 147.9229 |
| DALYs | Andean Latin America | Dietary iron deficiency | 2017 | 225.8744 | 337.4159 | 143.7975 |
| DALYs | Andean Latin America | Dietary iron deficiency | 2018 | 222.8439 | 333.728 | 140.482 |
| DALYs | Andean Latin America | Dietary iron deficiency | 2019 | 220.8831 | 331.7033 | 138.314 |
| DALYs | Andean Latin America | Iodine deficiency | 1990 | 1.352367 | 2.362165 | 0.74373 |
| DALYs | Andean Latin America | Iodine deficiency | 1991 | 1.59718 | 2.690664 | 0.938606 |
| DALYs | Andean Latin America | Iodine deficiency | 1992 | 1.786465 | 2.956139 | 1.079217 |
| DALYs | Andean Latin America | Iodine deficiency | 1993 | 1.917293 | 3.20204 | 1.153535 |
| DALYs | Andean Latin America | Iodine deficiency | 1994 | 1.992098 | 3.274518 | 1.183296 |
| DALYs | Andean Latin America | Iodine deficiency | 1995 | 2.01316 | 3.373839 | 1.137143 |
| DALYs | Andean Latin America | Iodine deficiency | 1996 | 1.931236 | 3.232582 | 1.128713 |
| DALYs | Andean Latin America | Iodine deficiency | 1997 | 1.739809 | 2.923992 | 1.026724 |
| DALYs | Andean Latin America | Iodine deficiency | 1998 | 1.513804 | 2.609265 | 0.863609 |
| DALYs | Andean Latin America | Iodine deficiency | 1999 | 1.323732 | 2.325387 | 0.723507 |
| DALYs | Andean Latin America | Iodine deficiency | 2000 | 1.238097 | 2.223564 | 0.653239 |
| DALYs | Andean Latin America | Iodine deficiency | 2001 | 1.229784 | 2.206372 | 0.643288 |
| DALYs | Andean Latin America | Iodine deficiency | 2002 | 1.219803 | 2.201546 | 0.634179 |
| DALYs | Andean Latin America | Iodine deficiency | 2003 | 1.208142 | 2.179732 | 0.621215 |
| DALYs | Andean Latin America | Iodine deficiency | 2004 | 1.198789 | 2.149477 | 0.610342 |
| DALYs | Andean Latin America | Iodine deficiency | 2005 | 1.187775 | 2.143903 | 0.60482 |
| DALYs | Andean Latin America | Iodine deficiency | 2006 | 1.178251 | 2.125445 | 0.606036 |
| DALYs | Andean Latin America | Iodine deficiency | 2007 | 1.166465 | 2.124917 | 0.592067 |
| DALYs | Andean Latin America | Iodine deficiency | 2008 | 1.15136 | 2.079163 | 0.59377 |
| DALYs | Andean Latin America | Iodine deficiency | 2009 | 1.135752 | 2.088429 | 0.578496 |
| DALYs | Andean Latin America | Iodine deficiency | 2010 | 1.121346 | 2.081044 | 0.569023 |
| DALYs | Andean Latin America | Iodine deficiency | 2011 | 1.105011 | 2.009258 | 0.566662 |
| DALYs | Andean Latin America | Iodine deficiency | 2012 | 1.083795 | 1.981764 | 0.557385 |
| DALYs | Andean Latin America | Iodine deficiency | 2013 | 1.065797 | 1.948159 | 0.549593 |
| DALYs | Andean Latin America | Iodine deficiency | 2014 | 1.048002 | 1.909505 | 0.542425 |
| DALYs | Andean Latin America | Iodine deficiency | 2015 | 1.03753 | 1.906895 | 0.526342 |
| DALYs | Andean Latin America | Iodine deficiency | 2016 | 1.032299 | 1.898456 | 0.523268 |
| DALYs | Andean Latin America | Iodine deficiency | 2017 | 1.026912 | 1.886741 | 0.52674 |
| DALYs | Andean Latin America | Iodine deficiency | 2018 | 1.023274 | 1.861645 | 0.513189 |
| DALYs | Andean Latin America | Iodine deficiency | 2019 | 1.015011 | 1.892365 | 0.508967 |
| DALYs | Andean Latin America | Vitamin A deficiency | 1990 | 19.28603 | 27.66836 | 12.71874 |
| DALYs | Andean Latin America | Vitamin A deficiency | 1991 | 18.9282 | 27.49589 | 12.54056 |
| DALYs | Andean Latin America | Vitamin A deficiency | 1992 | 18.56349 | 26.83122 | 12.26892 |
| DALYs | Andean Latin America | Vitamin A deficiency | 1993 | 18.2079 | 26.42435 | 11.98446 |
| DALYs | Andean Latin America | Vitamin A deficiency | 1994 | 17.90306 | 25.80444 | 11.79074 |
| DALYs | Andean Latin America | Vitamin A deficiency | 1995 | 17.627 | 26.0556 | 11.53716 |
| DALYs | Andean Latin America | Vitamin A deficiency | 1996 | 17.39218 | 25.43961 | 11.49275 |
| DALYs | Andean Latin America | Vitamin A deficiency | 1997 | 17.15437 | 25.24329 | 11.2233 |
| DALYs | Andean Latin America | Vitamin A deficiency | 1998 | 16.92115 | 24.66409 | 11.12045 |
| DALYs | Andean Latin America | Vitamin A deficiency | 1999 | 16.61431 | 24.52053 | 10.76908 |
| DALYs | Andean Latin America | Vitamin A deficiency | 2000 | 16.31809 | 23.99237 | 10.52973 |
| DALYs | Andean Latin America | Vitamin A deficiency | 2001 | 15.91835 | 23.39191 | 10.32674 |
| DALYs | Andean Latin America | Vitamin A deficiency | 2002 | 15.42148 | 22.40874 | 10.00718 |
| DALYs | Andean Latin America | Vitamin A deficiency | 2003 | 14.854 | 21.94088 | 9.681886 |
| DALYs | Andean Latin America | Vitamin A deficiency | 2004 | 14.30641 | 20.99144 | 9.296563 |
| DALYs | Andean Latin America | Vitamin A deficiency | 2005 | 13.81342 | 20.18341 | 9.029236 |
| DALYs | Andean Latin America | Vitamin A deficiency | 2006 | 13.3595 | 19.49126 | 8.6711 |
| DALYs | Andean Latin America | Vitamin A deficiency | 2007 | 12.89114 | 18.52927 | 8.340063 |
| DALYs | Andean Latin America | Vitamin A deficiency | 2008 | 12.40175 | 17.96621 | 8.047837 |
| DALYs | Andean Latin America | Vitamin A deficiency | 2009 | 11.96892 | 17.41432 | 7.753267 |
| DALYs | Andean Latin America | Vitamin A deficiency | 2010 | 11.59191 | 16.90315 | 7.629174 |
| DALYs | Andean Latin America | Vitamin A deficiency | 2011 | 11.27714 | 16.34304 | 7.310514 |
| DALYs | Andean Latin America | Vitamin A deficiency | 2012 | 10.94 | 15.92189 | 7.055976 |
| DALYs | Andean Latin America | Vitamin A deficiency | 2013 | 10.64145 | 15.52141 | 6.951448 |
| DALYs | Andean Latin America | Vitamin A deficiency | 2014 | 10.35242 | 14.91214 | 6.749906 |
| DALYs | Andean Latin America | Vitamin A deficiency | 2015 | 10.09551 | 14.67157 | 6.509306 |
| DALYs | Andean Latin America | Vitamin A deficiency | 2016 | 9.716548 | 14.10614 | 6.302273 |
| DALYs | Andean Latin America | Vitamin A deficiency | 2017 | 9.332789 | 13.68321 | 6.122173 |
| DALYs | Andean Latin America | Vitamin A deficiency | 2018 | 9.174519 | 13.39373 | 6.034356 |
| DALYs | Andean Latin America | Vitamin A deficiency | 2019 | 9.140168 | 13.34114 | 5.986759 |
| DALYs | Australasia | Dietary iron deficiency | 1990 | 100.8684 | 161.8896 | 62.37625 |
| DALYs | Australasia | Dietary iron deficiency | 1991 | 98.63669 | 156.7915 | 60.23627 |
| DALYs | Australasia | Dietary iron deficiency | 1992 | 96.43507 | 154.3737 | 58.57815 |
| DALYs | Australasia | Dietary iron deficiency | 1993 | 94.19248 | 151.7708 | 57.06749 |
| DALYs | Australasia | Dietary iron deficiency | 1994 | 92.04314 | 149.0096 | 54.91367 |
| DALYs | Australasia | Dietary iron deficiency | 1995 | 89.94739 | 146.4391 | 53.50958 |
| DALYs | Australasia | Dietary iron deficiency | 1996 | 87.80993 | 143.2026 | 51.47755 |
| DALYs | Australasia | Dietary iron deficiency | 1997 | 85.49352 | 139.2008 | 49.66735 |
| DALYs | Australasia | Dietary iron deficiency | 1998 | 83.17072 | 135.1747 | 47.99823 |
| DALYs | Australasia | Dietary iron deficiency | 1999 | 80.97598 | 130.8547 | 46.74355 |
| DALYs | Australasia | Dietary iron deficiency | 2000 | 79.08165 | 128.6699 | 45.33162 |
| DALYs | Australasia | Dietary iron deficiency | 2001 | 77.21818 | 124.8616 | 44.53409 |
| DALYs | Australasia | Dietary iron deficiency | 2002 | 75.28163 | 122.5641 | 43.3787 |
| DALYs | Australasia | Dietary iron deficiency | 2003 | 73.42088 | 121.8538 | 42.05155 |
| DALYs | Australasia | Dietary iron deficiency | 2004 | 71.78528 | 119.9971 | 40.68266 |
| DALYs | Australasia | Dietary iron deficiency | 2005 | 70.50829 | 117.5919 | 39.83083 |
| DALYs | Australasia | Dietary iron deficiency | 2006 | 69.55251 | 115.2797 | 39.16737 |
| DALYs | Australasia | Dietary iron deficiency | 2007 | 68.70657 | 113.5536 | 38.18665 |
| DALYs | Australasia | Dietary iron deficiency | 2008 | 67.95282 | 112.359 | 38.07209 |
| DALYs | Australasia | Dietary iron deficiency | 2009 | 67.2678 | 110.6758 | 37.61459 |
| DALYs | Australasia | Dietary iron deficiency | 2010 | 66.66957 | 108.9324 | 37.26096 |
| DALYs | Australasia | Dietary iron deficiency | 2011 | 66.01346 | 108.1608 | 37.21942 |
| DALYs | Australasia | Dietary iron deficiency | 2012 | 65.29085 | 106.1575 | 37.05329 |
| DALYs | Australasia | Dietary iron deficiency | 2013 | 64.63843 | 105.6484 | 36.42467 |
| DALYs | Australasia | Dietary iron deficiency | 2014 | 64.02819 | 104.4125 | 36.25819 |
| DALYs | Australasia | Dietary iron deficiency | 2015 | 63.59907 | 104.3258 | 35.51131 |
| DALYs | Australasia | Dietary iron deficiency | 2016 | 63.62394 | 103.5195 | 36.05179 |
| DALYs | Australasia | Dietary iron deficiency | 2017 | 63.70236 | 103.0682 | 36.29642 |
| DALYs | Australasia | Dietary iron deficiency | 2018 | 63.41918 | 103.2847 | 35.94974 |
| DALYs | Australasia | Dietary iron deficiency | 2019 | 63.06488 | 104.2207 | 35.9487 |
| DALYs | Australasia | Iodine deficiency | 1990 | 2.357842 | 4.437002 | 1.090227 |
| DALYs | Australasia | Iodine deficiency | 1991 | 2.354551 | 4.47359 | 1.077136 |
| DALYs | Australasia | Iodine deficiency | 1992 | 2.353603 | 4.470093 | 1.074429 |
| DALYs | Australasia | Iodine deficiency | 1993 | 2.346456 | 4.492611 | 1.058983 |
| DALYs | Australasia | Iodine deficiency | 1994 | 2.343743 | 4.472886 | 1.06315 |
| DALYs | Australasia | Iodine deficiency | 1995 | 2.342651 | 4.464993 | 1.064478 |
| DALYs | Australasia | Iodine deficiency | 1996 | 2.337804 | 4.408763 | 1.06913 |
| DALYs | Australasia | Iodine deficiency | 1997 | 2.335032 | 4.438362 | 1.068262 |
| DALYs | Australasia | Iodine deficiency | 1998 | 2.332524 | 4.43265 | 1.064554 |
| DALYs | Australasia | Iodine deficiency | 1999 | 2.329605 | 4.491341 | 1.048563 |
| DALYs | Australasia | Iodine deficiency | 2000 | 2.327816 | 4.431682 | 1.047683 |
| DALYs | Australasia | Iodine deficiency | 2001 | 2.320018 | 4.490789 | 1.061859 |
| DALYs | Australasia | Iodine deficiency | 2002 | 2.310824 | 4.348117 | 1.045406 |
| DALYs | Australasia | Iodine deficiency | 2003 | 2.303998 | 4.403699 | 1.0621 |
| DALYs | Australasia | Iodine deficiency | 2004 | 2.29267 | 4.33738 | 1.028604 |
| DALYs | Australasia | Iodine deficiency | 2005 | 2.28686 | 4.354863 | 1.047231 |
| DALYs | Australasia | Iodine deficiency | 2006 | 2.278604 | 4.349634 | 1.042269 |
| DALYs | Australasia | Iodine deficiency | 2007 | 2.273696 | 4.307712 | 1.028853 |
| DALYs | Australasia | Iodine deficiency | 2008 | 2.266367 | 4.370086 | 1.027329 |
| DALYs | Australasia | Iodine deficiency | 2009 | 2.259439 | 4.277293 | 1.032153 |
| DALYs | Australasia | Iodine deficiency | 2010 | 2.25552 | 4.34474 | 1.022228 |
| DALYs | Australasia | Iodine deficiency | 2011 | 2.248541 | 4.225219 | 1.025214 |
| DALYs | Australasia | Iodine deficiency | 2012 | 2.245233 | 4.246839 | 1.022682 |
| DALYs | Australasia | Iodine deficiency | 2013 | 2.239395 | 4.219866 | 1.02669 |
| DALYs | Australasia | Iodine deficiency | 2014 | 2.232549 | 4.271506 | 1.028006 |
| DALYs | Australasia | Iodine deficiency | 2015 | 2.226997 | 4.239134 | 1.013352 |
| DALYs | Australasia | Iodine deficiency | 2016 | 2.227612 | 4.232011 | 1.014216 |
| DALYs | Australasia | Iodine deficiency | 2017 | 2.226177 | 4.298375 | 1.003604 |
| DALYs | Australasia | Iodine deficiency | 2018 | 2.220394 | 4.247094 | 1.003226 |
| DALYs | Australasia | Iodine deficiency | 2019 | 2.213685 | 4.280211 | 1.011747 |
| DALYs | Australasia | Vitamin A deficiency | 1990 | 0.103096 | 0.191667 | 0.052779 |
| DALYs | Australasia | Vitamin A deficiency | 1991 | 0.096627 | 0.177262 | 0.049743 |
| DALYs | Australasia | Vitamin A deficiency | 1992 | 0.09006 | 0.168205 | 0.046253 |
| DALYs | Australasia | Vitamin A deficiency | 1993 | 0.08479 | 0.155193 | 0.042923 |
| DALYs | Australasia | Vitamin A deficiency | 1994 | 0.080712 | 0.148577 | 0.040217 |
| DALYs | Australasia | Vitamin A deficiency | 1995 | 0.077973 | 0.14329 | 0.038153 |
| DALYs | Australasia | Vitamin A deficiency | 1996 | 0.076167 | 0.138551 | 0.037427 |
| DALYs | Australasia | Vitamin A deficiency | 1997 | 0.074233 | 0.137798 | 0.035768 |
| DALYs | Australasia | Vitamin A deficiency | 1998 | 0.072781 | 0.13096 | 0.034955 |
| DALYs | Australasia | Vitamin A deficiency | 1999 | 0.072091 | 0.133985 | 0.034541 |
| DALYs | Australasia | Vitamin A deficiency | 2000 | 0.071261 | 0.128002 | 0.034189 |
| DALYs | Australasia | Vitamin A deficiency | 2001 | 0.071734 | 0.131984 | 0.034197 |
| DALYs | Australasia | Vitamin A deficiency | 2002 | 0.073638 | 0.135314 | 0.035637 |
| DALYs | Australasia | Vitamin A deficiency | 2003 | 0.075705 | 0.140478 | 0.03712 |
| DALYs | Australasia | Vitamin A deficiency | 2004 | 0.077868 | 0.14497 | 0.037771 |
| DALYs | Australasia | Vitamin A deficiency | 2005 | 0.078666 | 0.146078 | 0.037266 |
| DALYs | Australasia | Vitamin A deficiency | 2006 | 0.078169 | 0.146929 | 0.038771 |
| DALYs | Australasia | Vitamin A deficiency | 2007 | 0.077337 | 0.139506 | 0.036252 |
| DALYs | Australasia | Vitamin A deficiency | 2008 | 0.076118 | 0.141456 | 0.036815 |
| DALYs | Australasia | Vitamin A deficiency | 2009 | 0.074702 | 0.136893 | 0.035388 |
| DALYs | Australasia | Vitamin A deficiency | 2010 | 0.073348 | 0.138135 | 0.034784 |
| DALYs | Australasia | Vitamin A deficiency | 2011 | 0.070676 | 0.130369 | 0.033783 |
| DALYs | Australasia | Vitamin A deficiency | 2012 | 0.067314 | 0.125221 | 0.03313 |
| DALYs | Australasia | Vitamin A deficiency | 2013 | 0.063785 | 0.119292 | 0.030135 |
| DALYs | Australasia | Vitamin A deficiency | 2014 | 0.060424 | 0.108929 | 0.028783 |
| DALYs | Australasia | Vitamin A deficiency | 2015 | 0.057623 | 0.106104 | 0.028768 |
| DALYs | Australasia | Vitamin A deficiency | 2016 | 0.05561 | 0.104069 | 0.027446 |
| DALYs | Australasia | Vitamin A deficiency | 2017 | 0.054296 | 0.098646 | 0.026293 |
| DALYs | Australasia | Vitamin A deficiency | 2018 | 0.053021 | 0.100139 | 0.025863 |
| DALYs | Australasia | Vitamin A deficiency | 2019 | 0.051432 | 0.092569 | 0.026126 |
| DALYs | Caribbean | Dietary iron deficiency | 1990 | 405.1955 | 590.4741 | 268.8598 |
| DALYs | Caribbean | Dietary iron deficiency | 1991 | 402.8245 | 587.247 | 268.3821 |
| DALYs | Caribbean | Dietary iron deficiency | 1992 | 400.3111 | 584.049 | 266.2555 |
| DALYs | Caribbean | Dietary iron deficiency | 1993 | 397.6716 | 580.9496 | 263.8309 |
| DALYs | Caribbean | Dietary iron deficiency | 1994 | 395.0413 | 577.898 | 261.6511 |
| DALYs | Caribbean | Dietary iron deficiency | 1995 | 392.6315 | 575.3731 | 260.5759 |
| DALYs | Caribbean | Dietary iron deficiency | 1996 | 390.1873 | 570.9199 | 257.4825 |
| DALYs | Caribbean | Dietary iron deficiency | 1997 | 387.3299 | 566.0053 | 256.1207 |
| DALYs | Caribbean | Dietary iron deficiency | 1998 | 384.2351 | 561.4879 | 254.8618 |
| DALYs | Caribbean | Dietary iron deficiency | 1999 | 381.2791 | 557.8873 | 252.3696 |
| DALYs | Caribbean | Dietary iron deficiency | 2000 | 378.632 | 555.232 | 250.1177 |
| DALYs | Caribbean | Dietary iron deficiency | 2001 | 375.9034 | 550.1271 | 247.569 |
| DALYs | Caribbean | Dietary iron deficiency | 2002 | 372.8828 | 546.6247 | 245.5192 |
| DALYs | Caribbean | Dietary iron deficiency | 2003 | 369.8923 | 540.3458 | 242.9114 |
| DALYs | Caribbean | Dietary iron deficiency | 2004 | 367.5169 | 538.2683 | 239.8338 |
| DALYs | Caribbean | Dietary iron deficiency | 2005 | 365.948 | 535.4032 | 238.5454 |
| DALYs | Caribbean | Dietary iron deficiency | 2006 | 365.1176 | 533.1408 | 238.4613 |
| DALYs | Caribbean | Dietary iron deficiency | 2007 | 364.269 | 534.4478 | 237.8509 |
| DALYs | Caribbean | Dietary iron deficiency | 2008 | 363.6756 | 532.5607 | 236.2467 |
| DALYs | Caribbean | Dietary iron deficiency | 2009 | 363.6564 | 534.383 | 236.4213 |
| DALYs | Caribbean | Dietary iron deficiency | 2010 | 363.4696 | 533.9128 | 235.7829 |
| DALYs | Caribbean | Dietary iron deficiency | 2011 | 364.122 | 536.0798 | 236.3104 |
| DALYs | Caribbean | Dietary iron deficiency | 2012 | 366.2311 | 539.9104 | 236.8561 |
| DALYs | Caribbean | Dietary iron deficiency | 2013 | 368.5669 | 540.9072 | 237.2951 |
| DALYs | Caribbean | Dietary iron deficiency | 2014 | 370.6757 | 544.8802 | 241.3448 |
| DALYs | Caribbean | Dietary iron deficiency | 2015 | 372.3081 | 549.1745 | 243.6436 |
| DALYs | Caribbean | Dietary iron deficiency | 2016 | 373.4815 | 550.2848 | 244.3486 |
| DALYs | Caribbean | Dietary iron deficiency | 2017 | 373.9216 | 549.7661 | 245.022 |
| DALYs | Caribbean | Dietary iron deficiency | 2018 | 373.0551 | 549.6536 | 245.036 |
| DALYs | Caribbean | Dietary iron deficiency | 2019 | 371.0928 | 548.6746 | 242.6936 |
| DALYs | Caribbean | Iodine deficiency | 1990 | 13.87743 | 22.53951 | 7.516332 |
| DALYs | Caribbean | Iodine deficiency | 1991 | 14.29363 | 23.24293 | 7.702629 |
| DALYs | Caribbean | Iodine deficiency | 1992 | 14.63649 | 24.10134 | 7.996063 |
| DALYs | Caribbean | Iodine deficiency | 1993 | 14.92752 | 24.19988 | 8.237119 |
| DALYs | Caribbean | Iodine deficiency | 1994 | 15.13162 | 24.76874 | 8.411999 |
| DALYs | Caribbean | Iodine deficiency | 1995 | 15.26558 | 24.86086 | 8.395875 |
| DALYs | Caribbean | Iodine deficiency | 1996 | 15.21498 | 24.59677 | 8.346512 |
| DALYs | Caribbean | Iodine deficiency | 1997 | 14.96823 | 24.17792 | 8.138913 |
| DALYs | Caribbean | Iodine deficiency | 1998 | 14.62703 | 23.67171 | 7.979084 |
| DALYs | Caribbean | Iodine deficiency | 1999 | 14.31915 | 23.13889 | 7.768529 |
| DALYs | Caribbean | Iodine deficiency | 2000 | 14.10875 | 22.81058 | 7.693146 |
| DALYs | Caribbean | Iodine deficiency | 2001 | 13.96895 | 22.62273 | 7.532688 |
| DALYs | Caribbean | Iodine deficiency | 2002 | 13.7382 | 22.23695 | 7.464299 |
| DALYs | Caribbean | Iodine deficiency | 2003 | 13.48395 | 21.77351 | 7.204893 |
| DALYs | Caribbean | Iodine deficiency | 2004 | 13.22068 | 21.46307 | 6.96653 |
| DALYs | Caribbean | Iodine deficiency | 2005 | 13.00414 | 21.04702 | 6.894126 |
| DALYs | Caribbean | Iodine deficiency | 2006 | 12.83039 | 20.82594 | 6.830205 |
| DALYs | Caribbean | Iodine deficiency | 2007 | 12.6844 | 20.62539 | 6.778072 |
| DALYs | Caribbean | Iodine deficiency | 2008 | 12.54062 | 20.32194 | 6.656992 |
| DALYs | Caribbean | Iodine deficiency | 2009 | 12.41399 | 20.14569 | 6.629698 |
| DALYs | Caribbean | Iodine deficiency | 2010 | 12.20785 | 19.76902 | 6.655438 |
| DALYs | Caribbean | Iodine deficiency | 2011 | 11.96377 | 19.61942 | 6.563132 |
| DALYs | Caribbean | Iodine deficiency | 2012 | 11.68523 | 18.89109 | 6.33875 |
| DALYs | Caribbean | Iodine deficiency | 2013 | 11.3713 | 18.62507 | 6.23615 |
| DALYs | Caribbean | Iodine deficiency | 2014 | 11.10602 | 18.24389 | 6.071249 |
| DALYs | Caribbean | Iodine deficiency | 2015 | 10.93466 | 17.92847 | 5.935985 |
| DALYs | Caribbean | Iodine deficiency | 2016 | 10.80031 | 17.82183 | 5.807579 |
| DALYs | Caribbean | Iodine deficiency | 2017 | 10.73196 | 17.82214 | 5.768203 |
| DALYs | Caribbean | Iodine deficiency | 2018 | 10.71515 | 17.79924 | 5.709957 |
| DALYs | Caribbean | Iodine deficiency | 2019 | 10.69397 | 17.68143 | 5.75315 |
| DALYs | Caribbean | Vitamin A deficiency | 1990 | 16.71436 | 24.05657 | 10.9643 |
| DALYs | Caribbean | Vitamin A deficiency | 1991 | 16.51898 | 23.97853 | 10.65129 |
| DALYs | Caribbean | Vitamin A deficiency | 1992 | 16.31165 | 23.6427 | 10.55465 |
| DALYs | Caribbean | Vitamin A deficiency | 1993 | 16.1068 | 23.50537 | 10.37315 |
| DALYs | Caribbean | Vitamin A deficiency | 1994 | 15.90786 | 23.08086 | 10.21671 |
| DALYs | Caribbean | Vitamin A deficiency | 1995 | 15.7011 | 22.86212 | 10.08781 |
| DALYs | Caribbean | Vitamin A deficiency | 1996 | 15.50254 | 22.44442 | 9.955999 |
| DALYs | Caribbean | Vitamin A deficiency | 1997 | 15.26764 | 22.31584 | 9.859003 |
| DALYs | Caribbean | Vitamin A deficiency | 1998 | 15.03461 | 21.86807 | 9.673737 |
| DALYs | Caribbean | Vitamin A deficiency | 1999 | 14.82253 | 21.75283 | 9.611257 |
| DALYs | Caribbean | Vitamin A deficiency | 2000 | 14.64545 | 21.62679 | 9.423313 |
| DALYs | Caribbean | Vitamin A deficiency | 2001 | 14.48912 | 21.27422 | 9.340401 |
| DALYs | Caribbean | Vitamin A deficiency | 2002 | 14.30337 | 20.95661 | 9.264926 |
| DALYs | Caribbean | Vitamin A deficiency | 2003 | 14.14072 | 20.79437 | 9.171694 |
| DALYs | Caribbean | Vitamin A deficiency | 2004 | 14.00468 | 20.4745 | 9.012038 |
| DALYs | Caribbean | Vitamin A deficiency | 2005 | 13.95064 | 20.41805 | 8.898689 |
| DALYs | Caribbean | Vitamin A deficiency | 2006 | 13.88812 | 20.2996 | 8.9651 |
| DALYs | Caribbean | Vitamin A deficiency | 2007 | 13.77501 | 20.14766 | 8.814963 |
| DALYs | Caribbean | Vitamin A deficiency | 2008 | 13.63992 | 20.0288 | 8.794636 |
| DALYs | Caribbean | Vitamin A deficiency | 2009 | 13.52103 | 20.09708 | 8.591888 |
| DALYs | Caribbean | Vitamin A deficiency | 2010 | 13.42176 | 19.88565 | 8.605659 |
| DALYs | Caribbean | Vitamin A deficiency | 2011 | 13.35583 | 19.80508 | 8.505685 |
| DALYs | Caribbean | Vitamin A deficiency | 2012 | 13.3547 | 20.05056 | 8.638554 |
| DALYs | Caribbean | Vitamin A deficiency | 2013 | 13.34548 | 19.85177 | 8.588946 |
| DALYs | Caribbean | Vitamin A deficiency | 2014 | 13.3534 | 20.00319 | 8.637599 |
| DALYs | Caribbean | Vitamin A deficiency | 2015 | 13.30826 | 20.12031 | 8.589593 |
| DALYs | Caribbean | Vitamin A deficiency | 2016 | 13.17491 | 19.72159 | 8.570378 |
| DALYs | Caribbean | Vitamin A deficiency | 2017 | 13.00993 | 19.59689 | 8.523205 |
| DALYs | Caribbean | Vitamin A deficiency | 2018 | 12.88645 | 19.25982 | 8.473919 |
| DALYs | Caribbean | Vitamin A deficiency | 2019 | 12.75534 | 19.05356 | 8.289168 |
| DALYs | Central Latin America | Dietary iron deficiency | 1990 | 218.3855 | 324.1607 | 143.1726 |
| DALYs | Central Latin America | Dietary iron deficiency | 1991 | 210.441 | 313.0888 | 137.7769 |
| DALYs | Central Latin America | Dietary iron deficiency | 1992 | 202.8685 | 301.891 | 133.2906 |
| DALYs | Central Latin America | Dietary iron deficiency | 1993 | 195.818 | 291.6311 | 128.6888 |
| DALYs | Central Latin America | Dietary iron deficiency | 1994 | 189.3379 | 281.1119 | 124.7404 |
| DALYs | Central Latin America | Dietary iron deficiency | 1995 | 183.5906 | 272.1867 | 120.6897 |
| DALYs | Central Latin America | Dietary iron deficiency | 1996 | 178.3089 | 265.193 | 117.2362 |
| DALYs | Central Latin America | Dietary iron deficiency | 1997 | 173.2183 | 258.0623 | 113.7711 |
| DALYs | Central Latin America | Dietary iron deficiency | 1998 | 168.4678 | 250.11 | 110.1836 |
| DALYs | Central Latin America | Dietary iron deficiency | 1999 | 164.2462 | 244.111 | 107.6396 |
| DALYs | Central Latin America | Dietary iron deficiency | 2000 | 160.684 | 238.5793 | 105.1744 |
| DALYs | Central Latin America | Dietary iron deficiency | 2001 | 157.7109 | 234.1283 | 102.7743 |
| DALYs | Central Latin America | Dietary iron deficiency | 2002 | 155.0666 | 229.998 | 100.589 |
| DALYs | Central Latin America | Dietary iron deficiency | 2003 | 152.6636 | 227.0363 | 98.85096 |
| DALYs | Central Latin America | Dietary iron deficiency | 2004 | 150.4645 | 223.7133 | 97.18058 |
| DALYs | Central Latin America | Dietary iron deficiency | 2005 | 148.4052 | 220.1729 | 95.95725 |
| DALYs | Central Latin America | Dietary iron deficiency | 2006 | 146.2195 | 216.8105 | 94.66512 |
| DALYs | Central Latin America | Dietary iron deficiency | 2007 | 143.8135 | 213.4216 | 93.1266 |
| DALYs | Central Latin America | Dietary iron deficiency | 2008 | 141.4741 | 209.4241 | 92.01625 |
| DALYs | Central Latin America | Dietary iron deficiency | 2009 | 139.4466 | 206.6032 | 91.28259 |
| DALYs | Central Latin America | Dietary iron deficiency | 2010 | 137.9997 | 203.9205 | 90.36126 |
| DALYs | Central Latin America | Dietary iron deficiency | 2011 | 137.0399 | 202.2707 | 90.13021 |
| DALYs | Central Latin America | Dietary iron deficiency | 2012 | 136.2654 | 200.0345 | 90.14415 |
| DALYs | Central Latin America | Dietary iron deficiency | 2013 | 135.573 | 199.7131 | 89.84551 |
| DALYs | Central Latin America | Dietary iron deficiency | 2014 | 134.9025 | 198.6789 | 89.65296 |
| DALYs | Central Latin America | Dietary iron deficiency | 2015 | 134.1481 | 197.5003 | 89.08045 |
| DALYs | Central Latin America | Dietary iron deficiency | 2016 | 133.3038 | 195.4957 | 88.38668 |
| DALYs | Central Latin America | Dietary iron deficiency | 2017 | 132.2214 | 193.0871 | 87.33187 |
| DALYs | Central Latin America | Dietary iron deficiency | 2018 | 130.412 | 190.1654 | 86.23633 |
| DALYs | Central Latin America | Dietary iron deficiency | 2019 | 127.8364 | 186.1762 | 84.57321 |
| DALYs | Central Latin America | Iodine deficiency | 1990 | 10.77801 | 18.39686 | 5.872518 |
| DALYs | Central Latin America | Iodine deficiency | 1991 | 10.78702 | 18.50565 | 5.853805 |
| DALYs | Central Latin America | Iodine deficiency | 1992 | 10.81122 | 18.46266 | 5.928914 |
| DALYs | Central Latin America | Iodine deficiency | 1993 | 10.83362 | 18.50716 | 5.982627 |
| DALYs | Central Latin America | Iodine deficiency | 1994 | 10.87053 | 18.56781 | 5.969178 |
| DALYs | Central Latin America | Iodine deficiency | 1995 | 10.89177 | 18.55365 | 6.016884 |
| DALYs | Central Latin America | Iodine deficiency | 1996 | 11.00698 | 18.87201 | 6.039633 |
| DALYs | Central Latin America | Iodine deficiency | 1997 | 11.24336 | 19.1569 | 6.242183 |
| DALYs | Central Latin America | Iodine deficiency | 1998 | 11.50972 | 19.52494 | 6.364322 |
| DALYs | Central Latin America | Iodine deficiency | 1999 | 11.70927 | 19.87365 | 6.354744 |
| DALYs | Central Latin America | Iodine deficiency | 2000 | 11.77936 | 19.9523 | 6.286212 |
| DALYs | Central Latin America | Iodine deficiency | 2001 | 11.36056 | 19.31509 | 6.396065 |
| DALYs | Central Latin America | Iodine deficiency | 2002 | 10.38766 | 18.09402 | 5.766867 |
| DALYs | Central Latin America | Iodine deficiency | 2003 | 9.186098 | 16.45419 | 4.87733 |
| DALYs | Central Latin America | Iodine deficiency | 2004 | 8.087325 | 14.91998 | 3.916306 |
| DALYs | Central Latin America | Iodine deficiency | 2005 | 7.606906 | 14.25615 | 3.477026 |
| DALYs | Central Latin America | Iodine deficiency | 2006 | 7.565088 | 14.17428 | 3.472138 |
| DALYs | Central Latin America | Iodine deficiency | 2007 | 7.498702 | 14.0726 | 3.430543 |
| DALYs | Central Latin America | Iodine deficiency | 2008 | 7.430262 | 13.91714 | 3.397737 |
| DALYs | Central Latin America | Iodine deficiency | 2009 | 7.371129 | 13.80403 | 3.378724 |
| DALYs | Central Latin America | Iodine deficiency | 2010 | 7.337393 | 13.73213 | 3.370347 |
| DALYs | Central Latin America | Iodine deficiency | 2011 | 7.576023 | 13.99659 | 3.597401 |
| DALYs | Central Latin America | Iodine deficiency | 2012 | 8.168616 | 14.80432 | 4.144558 |
| DALYs | Central Latin America | Iodine deficiency | 2013 | 8.825673 | 15.58743 | 4.708736 |
| DALYs | Central Latin America | Iodine deficiency | 2014 | 9.365546 | 16.27985 | 5.028426 |
| DALYs | Central Latin America | Iodine deficiency | 2015 | 9.598388 | 16.74465 | 5.137025 |
| DALYs | Central Latin America | Iodine deficiency | 2016 | 9.806667 | 17.13009 | 5.301719 |
| DALYs | Central Latin America | Iodine deficiency | 2017 | 9.980439 | 17.32395 | 5.402498 |
| DALYs | Central Latin America | Iodine deficiency | 2018 | 9.960126 | 17.26578 | 5.390203 |
| DALYs | Central Latin America | Iodine deficiency | 2019 | 9.889994 | 17.18787 | 5.252783 |
| DALYs | Central Latin America | Vitamin A deficiency | 1990 | 14.99107 | 21.26501 | 9.895729 |
| DALYs | Central Latin America | Vitamin A deficiency | 1991 | 14.43583 | 20.51599 | 9.497151 |
| DALYs | Central Latin America | Vitamin A deficiency | 1992 | 13.88691 | 19.88821 | 9.141624 |
| DALYs | Central Latin America | Vitamin A deficiency | 1993 | 13.36591 | 19.04382 | 8.911025 |
| DALYs | Central Latin America | Vitamin A deficiency | 1994 | 12.89868 | 18.46054 | 8.604377 |
| DALYs | Central Latin America | Vitamin A deficiency | 1995 | 12.49206 | 17.91211 | 8.29179 |
| DALYs | Central Latin America | Vitamin A deficiency | 1996 | 12.09882 | 17.45309 | 8.06864 |
| DALYs | Central Latin America | Vitamin A deficiency | 1997 | 11.70041 | 16.55863 | 7.762112 |
| DALYs | Central Latin America | Vitamin A deficiency | 1998 | 11.32346 | 16.04121 | 7.469469 |
| DALYs | Central Latin America | Vitamin A deficiency | 1999 | 10.98075 | 15.64899 | 7.260892 |
| DALYs | Central Latin America | Vitamin A deficiency | 2000 | 10.68828 | 15.24758 | 7.039839 |
| DALYs | Central Latin America | Vitamin A deficiency | 2001 | 10.43298 | 14.85604 | 6.874563 |
| DALYs | Central Latin America | Vitamin A deficiency | 2002 | 10.19646 | 14.56482 | 6.763133 |
| DALYs | Central Latin America | Vitamin A deficiency | 2003 | 9.972332 | 14.26765 | 6.593803 |
| DALYs | Central Latin America | Vitamin A deficiency | 2004 | 9.771522 | 14.07215 | 6.431458 |
| DALYs | Central Latin America | Vitamin A deficiency | 2005 | 9.602006 | 13.91654 | 6.330045 |
| DALYs | Central Latin America | Vitamin A deficiency | 2006 | 9.432295 | 13.61678 | 6.26537 |
| DALYs | Central Latin America | Vitamin A deficiency | 2007 | 9.247937 | 13.20887 | 6.125293 |
| DALYs | Central Latin America | Vitamin A deficiency | 2008 | 9.057649 | 13.04074 | 5.980373 |
| DALYs | Central Latin America | Vitamin A deficiency | 2009 | 8.866647 | 12.75983 | 5.874377 |
| DALYs | Central Latin America | Vitamin A deficiency | 2010 | 8.709508 | 12.67108 | 5.723196 |
| DALYs | Central Latin America | Vitamin A deficiency | 2011 | 8.54628 | 12.39044 | 5.666412 |
| DALYs | Central Latin America | Vitamin A deficiency | 2012 | 8.373224 | 12.13607 | 5.547509 |
| DALYs | Central Latin America | Vitamin A deficiency | 2013 | 8.208714 | 11.90312 | 5.473088 |
| DALYs | Central Latin America | Vitamin A deficiency | 2014 | 8.047272 | 11.57835 | 5.358311 |
| DALYs | Central Latin America | Vitamin A deficiency | 2015 | 7.911395 | 11.33203 | 5.26918 |
| DALYs | Central Latin America | Vitamin A deficiency | 2016 | 7.835898 | 11.28998 | 5.21799 |
| DALYs | Central Latin America | Vitamin A deficiency | 2017 | 7.778664 | 11.24441 | 5.225334 |
| DALYs | Central Latin America | Vitamin A deficiency | 2018 | 7.703612 | 11.06632 | 5.107394 |
| DALYs | Central Latin America | Vitamin A deficiency | 2019 | 7.633278 | 10.96918 | 5.056648 |
| DALYs | Central Sub-Saharan Africa | Dietary iron deficiency | 1990 | 574.3086 | 837.9614 | 379.769 |
| DALYs | Central Sub-Saharan Africa | Dietary iron deficiency | 1991 | 574.0607 | 838.1746 | 380.5682 |
| DALYs | Central Sub-Saharan Africa | Dietary iron deficiency | 1992 | 573.4084 | 835.1673 | 383.5873 |
| DALYs | Central Sub-Saharan Africa | Dietary iron deficiency | 1993 | 572.4315 | 833.5848 | 383.5859 |
| DALYs | Central Sub-Saharan Africa | Dietary iron deficiency | 1994 | 570.9441 | 832.4148 | 382.7481 |
| DALYs | Central Sub-Saharan Africa | Dietary iron deficiency | 1995 | 569.0362 | 827.1652 | 380.2147 |
| DALYs | Central Sub-Saharan Africa | Dietary iron deficiency | 1996 | 566.7258 | 821.8875 | 379.9456 |
| DALYs | Central Sub-Saharan Africa | Dietary iron deficiency | 1997 | 563.8338 | 820.5716 | 379.4099 |
| DALYs | Central Sub-Saharan Africa | Dietary iron deficiency | 1998 | 560.5712 | 817.4258 | 376.0541 |
| DALYs | Central Sub-Saharan Africa | Dietary iron deficiency | 1999 | 556.5302 | 811.8474 | 374.6474 |
| DALYs | Central Sub-Saharan Africa | Dietary iron deficiency | 2000 | 551.916 | 806.2463 | 371.2838 |
| DALYs | Central Sub-Saharan Africa | Dietary iron deficiency | 2001 | 542.9782 | 792.3444 | 362.1425 |
| DALYs | Central Sub-Saharan Africa | Dietary iron deficiency | 2002 | 528.8733 | 772.9089 | 352.3154 |
| DALYs | Central Sub-Saharan Africa | Dietary iron deficiency | 2003 | 513.2925 | 751.9269 | 341.1479 |
| DALYs | Central Sub-Saharan Africa | Dietary iron deficiency | 2004 | 500.3023 | 733.7414 | 332.084 |
| DALYs | Central Sub-Saharan Africa | Dietary iron deficiency | 2005 | 493.2526 | 723.0622 | 326.9891 |
| DALYs | Central Sub-Saharan Africa | Dietary iron deficiency | 2006 | 491.9771 | 719.1936 | 324.9079 |
| DALYs | Central Sub-Saharan Africa | Dietary iron deficiency | 2007 | 492.8948 | 718.2412 | 324.7865 |
| DALYs | Central Sub-Saharan Africa | Dietary iron deficiency | 2008 | 494.6285 | 720.0676 | 325.2017 |
| DALYs | Central Sub-Saharan Africa | Dietary iron deficiency | 2009 | 496.5263 | 720.0355 | 327.302 |
| DALYs | Central Sub-Saharan Africa | Dietary iron deficiency | 2010 | 497.4284 | 720.4996 | 325.6648 |
| DALYs | Central Sub-Saharan Africa | Dietary iron deficiency | 2011 | 497.9177 | 723.7576 | 326.3522 |
| DALYs | Central Sub-Saharan Africa | Dietary iron deficiency | 2012 | 498.6034 | 720.6189 | 327.1514 |
| DALYs | Central Sub-Saharan Africa | Dietary iron deficiency | 2013 | 498.7243 | 723.6678 | 326.8038 |
| DALYs | Central Sub-Saharan Africa | Dietary iron deficiency | 2014 | 497.6584 | 722.0236 | 327.4267 |
| DALYs | Central Sub-Saharan Africa | Dietary iron deficiency | 2015 | 494.8755 | 715.8479 | 324.9221 |
| DALYs | Central Sub-Saharan Africa | Dietary iron deficiency | 2016 | 486.292 | 704.4261 | 321.9779 |
| DALYs | Central Sub-Saharan Africa | Dietary iron deficiency | 2017 | 477.0224 | 695.88 | 314.1746 |
| DALYs | Central Sub-Saharan Africa | Dietary iron deficiency | 2018 | 472.1798 | 685.8568 | 305.7937 |
| DALYs | Central Sub-Saharan Africa | Dietary iron deficiency | 2019 | 468.4445 | 681.8172 | 297.7672 |
| DALYs | Central Sub-Saharan Africa | Iodine deficiency | 1990 | 220.0395 | 394.7677 | 114.1069 |
| DALYs | Central Sub-Saharan Africa | Iodine deficiency | 1991 | 218.3361 | 390.6163 | 113.6987 |
| DALYs | Central Sub-Saharan Africa | Iodine deficiency | 1992 | 217.4566 | 390.5007 | 112.887 |
| DALYs | Central Sub-Saharan Africa | Iodine deficiency | 1993 | 217.4207 | 387.6556 | 112.316 |
| DALYs | Central Sub-Saharan Africa | Iodine deficiency | 1994 | 218.0912 | 396.6622 | 111.8224 |
| DALYs | Central Sub-Saharan Africa | Iodine deficiency | 1995 | 219.4638 | 400.8624 | 111.2587 |
| DALYs | Central Sub-Saharan Africa | Iodine deficiency | 1996 | 222.2834 | 405.7003 | 112.5913 |
| DALYs | Central Sub-Saharan Africa | Iodine deficiency | 1997 | 226.6129 | 412.261 | 115.644 |
| DALYs | Central Sub-Saharan Africa | Iodine deficiency | 1998 | 231.4108 | 421.5115 | 119.2501 |
| DALYs | Central Sub-Saharan Africa | Iodine deficiency | 1999 | 235.4323 | 426.2196 | 122.0384 |
| DALYs | Central Sub-Saharan Africa | Iodine deficiency | 2000 | 237.5721 | 426.6337 | 123.2537 |
| DALYs | Central Sub-Saharan Africa | Iodine deficiency | 2001 | 237.8834 | 431.0443 | 124.187 |
| DALYs | Central Sub-Saharan Africa | Iodine deficiency | 2002 | 237.1409 | 431.6221 | 123.4583 |
| DALYs | Central Sub-Saharan Africa | Iodine deficiency | 2003 | 235.6156 | 426.6368 | 123.4837 |
| DALYs | Central Sub-Saharan Africa | Iodine deficiency | 2004 | 233.428 | 421.8009 | 122.3053 |
| DALYs | Central Sub-Saharan Africa | Iodine deficiency | 2005 | 230.7366 | 413.8276 | 121.0615 |
| DALYs | Central Sub-Saharan Africa | Iodine deficiency | 2006 | 227.3214 | 409.6644 | 119.1797 |
| DALYs | Central Sub-Saharan Africa | Iodine deficiency | 2007 | 222.8236 | 400.1863 | 118.0379 |
| DALYs | Central Sub-Saharan Africa | Iodine deficiency | 2008 | 217.7946 | 390.2569 | 114.6433 |
| DALYs | Central Sub-Saharan Africa | Iodine deficiency | 2009 | 212.6325 | 381.264 | 113.827 |
| DALYs | Central Sub-Saharan Africa | Iodine deficiency | 2010 | 207.6816 | 371.6361 | 110.7788 |
| DALYs | Central Sub-Saharan Africa | Iodine deficiency | 2011 | 201.4537 | 360.9709 | 108.6965 |
| DALYs | Central Sub-Saharan Africa | Iodine deficiency | 2012 | 192.6687 | 346.4551 | 103.4121 |
| DALYs | Central Sub-Saharan Africa | Iodine deficiency | 2013 | 183.2521 | 333.9784 | 97.58506 |
| DALYs | Central Sub-Saharan Africa | Iodine deficiency | 2014 | 174.7161 | 317.2154 | 91.25902 |
| DALYs | Central Sub-Saharan Africa | Iodine deficiency | 2015 | 168.6662 | 309.8893 | 87.95216 |
| DALYs | Central Sub-Saharan Africa | Iodine deficiency | 2016 | 164.8728 | 302.4584 | 85.61291 |
| DALYs | Central Sub-Saharan Africa | Iodine deficiency | 2017 | 162.0249 | 298.1066 | 82.81934 |
| DALYs | Central Sub-Saharan Africa | Iodine deficiency | 2018 | 160.0959 | 295.8066 | 82.06785 |
| DALYs | Central Sub-Saharan Africa | Iodine deficiency | 2019 | 158.7541 | 291.7537 | 81.95789 |
| DALYs | Central Sub-Saharan Africa | Vitamin A deficiency | 1990 | 73.73112 | 103.1471 | 48.76156 |
| DALYs | Central Sub-Saharan Africa | Vitamin A deficiency | 1991 | 72.88803 | 103.2626 | 47.97044 |
| DALYs | Central Sub-Saharan Africa | Vitamin A deficiency | 1992 | 72.29871 | 101.9522 | 47.39995 |
| DALYs | Central Sub-Saharan Africa | Vitamin A deficiency | 1993 | 71.85666 | 101.3072 | 47.51467 |
| DALYs | Central Sub-Saharan Africa | Vitamin A deficiency | 1994 | 71.52584 | 100.7203 | 47.10072 |
| DALYs | Central Sub-Saharan Africa | Vitamin A deficiency | 1995 | 71.31665 | 100.4299 | 47.38491 |
| DALYs | Central Sub-Saharan Africa | Vitamin A deficiency | 1996 | 71.58945 | 101.0181 | 47.51687 |
| DALYs | Central Sub-Saharan Africa | Vitamin A deficiency | 1997 | 72.50523 | 102.086 | 48.91865 |
| DALYs | Central Sub-Saharan Africa | Vitamin A deficiency | 1998 | 73.66528 | 104.0429 | 49.46839 |
| DALYs | Central Sub-Saharan Africa | Vitamin A deficiency | 1999 | 74.88418 | 105.3698 | 50.19889 |
| DALYs | Central Sub-Saharan Africa | Vitamin A deficiency | 2000 | 75.83997 | 106.9105 | 51.53844 |
| DALYs | Central Sub-Saharan Africa | Vitamin A deficiency | 2001 | 76.72798 | 107.6303 | 51.90111 |
| DALYs | Central Sub-Saharan Africa | Vitamin A deficiency | 2002 | 77.80864 | 109.5679 | 52.93289 |
| DALYs | Central Sub-Saharan Africa | Vitamin A deficiency | 2003 | 78.81528 | 109.8877 | 53.43112 |
| DALYs | Central Sub-Saharan Africa | Vitamin A deficiency | 2004 | 79.50609 | 111.5617 | 54.51654 |
| DALYs | Central Sub-Saharan Africa | Vitamin A deficiency | 2005 | 79.58918 | 111.9529 | 54.08128 |
| DALYs | Central Sub-Saharan Africa | Vitamin A deficiency | 2006 | 78.98742 | 111.0448 | 54.08896 |
| DALYs | Central Sub-Saharan Africa | Vitamin A deficiency | 2007 | 77.89104 | 110.1113 | 53.15921 |
| DALYs | Central Sub-Saharan Africa | Vitamin A deficiency | 2008 | 76.50764 | 108.3105 | 52.25863 |
| DALYs | Central Sub-Saharan Africa | Vitamin A deficiency | 2009 | 74.90415 | 105.6184 | 51.21283 |
| DALYs | Central Sub-Saharan Africa | Vitamin A deficiency | 2010 | 73.32136 | 103.6661 | 49.90971 |
| DALYs | Central Sub-Saharan Africa | Vitamin A deficiency | 2011 | 71.10507 | 101.0144 | 48.30004 |
| DALYs | Central Sub-Saharan Africa | Vitamin A deficiency | 2012 | 67.71627 | 95.86364 | 45.54022 |
| DALYs | Central Sub-Saharan Africa | Vitamin A deficiency | 2013 | 63.88507 | 91.06054 | 42.7498 |
| DALYs | Central Sub-Saharan Africa | Vitamin A deficiency | 2014 | 60.09762 | 85.13854 | 40.45953 |
| DALYs | Central Sub-Saharan Africa | Vitamin A deficiency | 2015 | 56.97065 | 81.24174 | 38.03748 |
| DALYs | Central Sub-Saharan Africa | Vitamin A deficiency | 2016 | 54.3336 | 77.23276 | 36.21689 |
| DALYs | Central Sub-Saharan Africa | Vitamin A deficiency | 2017 | 52.11264 | 74.42358 | 34.5688 |
| DALYs | Central Sub-Saharan Africa | Vitamin A deficiency | 2018 | 50.38064 | 71.8228 | 33.39526 |
| DALYs | Central Sub-Saharan Africa | Vitamin A deficiency | 2019 | 49.08387 | 69.87537 | 32.59157 |
| DALYs | East Asia | Dietary iron deficiency | 1990 | 299.3029 | 430.5871 | 197.7619 |
| DALYs | East Asia | Dietary iron deficiency | 1991 | 292.8213 | 419.9899 | 193.8645 |
| DALYs | East Asia | Dietary iron deficiency | 1992 | 285.4883 | 408.7089 | 187.9773 |
| DALYs | East Asia | Dietary iron deficiency | 1993 | 277.3703 | 396.7239 | 182.7439 |
| DALYs | East Asia | Dietary iron deficiency | 1994 | 268.7168 | 383.539 | 177.2798 |
| DALYs | East Asia | Dietary iron deficiency | 1995 | 259.6078 | 370.8633 | 172.1536 |
| DALYs | East Asia | Dietary iron deficiency | 1996 | 249.0538 | 357.5678 | 164.9515 |
| DALYs | East Asia | Dietary iron deficiency | 1997 | 236.5696 | 342.0472 | 157.0527 |
| DALYs | East Asia | Dietary iron deficiency | 1998 | 223.2449 | 323.4987 | 148.2215 |
| DALYs | East Asia | Dietary iron deficiency | 1999 | 210.1522 | 305.7316 | 139.1141 |
| DALYs | East Asia | Dietary iron deficiency | 2000 | 198.4693 | 289.062 | 130.9829 |
| DALYs | East Asia | Dietary iron deficiency | 2001 | 187.7848 | 275.4745 | 123.5803 |
| DALYs | East Asia | Dietary iron deficiency | 2002 | 177.1262 | 261.5229 | 116.0402 |
| DALYs | East Asia | Dietary iron deficiency | 2003 | 166.7136 | 245.3307 | 108.7744 |
| DALYs | East Asia | Dietary iron deficiency | 2004 | 156.824 | 231.1356 | 102.0965 |
| DALYs | East Asia | Dietary iron deficiency | 2005 | 147.7775 | 218.5391 | 96.93001 |
| DALYs | East Asia | Dietary iron deficiency | 2006 | 138.7796 | 206.0047 | 90.70868 |
| DALYs | East Asia | Dietary iron deficiency | 2007 | 129.4683 | 191.9607 | 85.03916 |
| DALYs | East Asia | Dietary iron deficiency | 2008 | 120.6505 | 178.1123 | 79.50407 |
| DALYs | East Asia | Dietary iron deficiency | 2009 | 113.1065 | 166.9507 | 73.92849 |
| DALYs | East Asia | Dietary iron deficiency | 2010 | 107.6283 | 159.8324 | 69.98966 |
| DALYs | East Asia | Dietary iron deficiency | 2011 | 103.7758 | 155.4317 | 67.22406 |
| DALYs | East Asia | Dietary iron deficiency | 2012 | 100.4913 | 151.1574 | 64.55645 |
| DALYs | East Asia | Dietary iron deficiency | 2013 | 97.64533 | 147.4562 | 62.4649 |
| DALYs | East Asia | Dietary iron deficiency | 2014 | 95.1538 | 143.3863 | 60.82891 |
| DALYs | East Asia | Dietary iron deficiency | 2015 | 92.90558 | 140.2617 | 59.15382 |
| DALYs | East Asia | Dietary iron deficiency | 2016 | 90.90812 | 136.6758 | 57.82519 |
| DALYs | East Asia | Dietary iron deficiency | 2017 | 89.01085 | 134.2961 | 56.6333 |
| DALYs | East Asia | Dietary iron deficiency | 2018 | 86.91033 | 131.2984 | 55.39331 |
| DALYs | East Asia | Dietary iron deficiency | 2019 | 84.68404 | 128.6792 | 53.78085 |
| DALYs | East Asia | Iodine deficiency | 1990 | 19.17712 | 32.61072 | 10.87264 |
| DALYs | East Asia | Iodine deficiency | 1991 | 18.95066 | 32.78658 | 10.53963 |
| DALYs | East Asia | Iodine deficiency | 1992 | 18.72488 | 32.82509 | 10.43918 |
| DALYs | East Asia | Iodine deficiency | 1993 | 18.51169 | 32.85636 | 10.14613 |
| DALYs | East Asia | Iodine deficiency | 1994 | 18.35061 | 32.91017 | 9.823445 |
| DALYs | East Asia | Iodine deficiency | 1995 | 18.31149 | 33.14884 | 9.812437 |
| DALYs | East Asia | Iodine deficiency | 1996 | 18.07618 | 33.10143 | 9.650096 |
| DALYs | East Asia | Iodine deficiency | 1997 | 17.50149 | 32.84659 | 9.020055 |
| DALYs | East Asia | Iodine deficiency | 1998 | 16.85584 | 32.27261 | 8.322755 |
| DALYs | East Asia | Iodine deficiency | 1999 | 16.3662 | 32.00847 | 7.806634 |
| DALYs | East Asia | Iodine deficiency | 2000 | 16.31586 | 31.93692 | 7.728942 |
| DALYs | East Asia | Iodine deficiency | 2001 | 16.94206 | 33.29196 | 7.980243 |
| DALYs | East Asia | Iodine deficiency | 2002 | 18.04475 | 35.33384 | 8.42264 |
| DALYs | East Asia | Iodine deficiency | 2003 | 19.26981 | 37.88697 | 8.914454 |
| DALYs | East Asia | Iodine deficiency | 2004 | 20.26199 | 39.86813 | 9.242384 |
| DALYs | East Asia | Iodine deficiency | 2005 | 20.67002 | 40.61927 | 9.48768 |
| DALYs | East Asia | Iodine deficiency | 2006 | 20.45022 | 40.13276 | 9.370908 |
| DALYs | East Asia | Iodine deficiency | 2007 | 19.90097 | 38.98783 | 9.121474 |
| DALYs | East Asia | Iodine deficiency | 2008 | 19.18612 | 37.64209 | 8.829036 |
| DALYs | East Asia | Iodine deficiency | 2009 | 18.4735 | 36.5014 | 8.475721 |
| DALYs | East Asia | Iodine deficiency | 2010 | 17.92175 | 35.35996 | 8.215232 |
| DALYs | East Asia | Iodine deficiency | 2011 | 17.52054 | 34.4692 | 8.035986 |
| DALYs | East Asia | Iodine deficiency | 2012 | 17.13038 | 33.74462 | 7.831367 |
| DALYs | East Asia | Iodine deficiency | 2013 | 16.76303 | 32.99784 | 7.689414 |
| DALYs | East Asia | Iodine deficiency | 2014 | 16.42723 | 32.30151 | 7.534135 |
| DALYs | East Asia | Iodine deficiency | 2015 | 16.12348 | 31.63235 | 7.420177 |
| DALYs | East Asia | Iodine deficiency | 2016 | 15.74658 | 30.91689 | 7.299402 |
| DALYs | East Asia | Iodine deficiency | 2017 | 15.42186 | 30.15008 | 7.121932 |
| DALYs | East Asia | Iodine deficiency | 2018 | 15.24039 | 29.95812 | 7.029894 |
| DALYs | East Asia | Iodine deficiency | 2019 | 15.08314 | 29.67767 | 6.952665 |
| DALYs | East Asia | Vitamin A deficiency | 1990 | 10.80269 | 15.79852 | 6.953698 |
| DALYs | East Asia | Vitamin A deficiency | 1991 | 10.28333 | 15.00294 | 6.694507 |
| DALYs | East Asia | Vitamin A deficiency | 1992 | 9.785375 | 14.10405 | 6.390579 |
| DALYs | East Asia | Vitamin A deficiency | 1993 | 9.315566 | 13.60765 | 6.13511 |
| DALYs | East Asia | Vitamin A deficiency | 1994 | 8.877122 | 12.86423 | 5.86316 |
| DALYs | East Asia | Vitamin A deficiency | 1995 | 8.483016 | 12.29636 | 5.689943 |
| DALYs | East Asia | Vitamin A deficiency | 1996 | 8.091243 | 11.6441 | 5.416101 |
| DALYs | East Asia | Vitamin A deficiency | 1997 | 7.703621 | 11.06601 | 5.148109 |
| DALYs | East Asia | Vitamin A deficiency | 1998 | 7.320512 | 10.49268 | 4.88902 |
| DALYs | East Asia | Vitamin A deficiency | 1999 | 6.969305 | 10.0199 | 4.636775 |
| DALYs | East Asia | Vitamin A deficiency | 2000 | 6.65719 | 9.53541 | 4.427743 |
| DALYs | East Asia | Vitamin A deficiency | 2001 | 6.351142 | 9.079803 | 4.228796 |
| DALYs | East Asia | Vitamin A deficiency | 2002 | 6.01626 | 8.62304 | 4.003264 |
| DALYs | East Asia | Vitamin A deficiency | 2003 | 5.68421 | 8.200907 | 3.785243 |
| DALYs | East Asia | Vitamin A deficiency | 2004 | 5.366042 | 7.705474 | 3.568181 |
| DALYs | East Asia | Vitamin A deficiency | 2005 | 5.086421 | 7.356846 | 3.370364 |
| DALYs | East Asia | Vitamin A deficiency | 2006 | 4.833232 | 7.005019 | 3.18451 |
| DALYs | East Asia | Vitamin A deficiency | 2007 | 4.594827 | 6.672299 | 3.030943 |
| DALYs | East Asia | Vitamin A deficiency | 2008 | 4.373058 | 6.407664 | 2.845739 |
| DALYs | East Asia | Vitamin A deficiency | 2009 | 4.189448 | 6.169319 | 2.71156 |
| DALYs | East Asia | Vitamin A deficiency | 2010 | 4.052874 | 6.047258 | 2.593884 |
| DALYs | East Asia | Vitamin A deficiency | 2011 | 3.950255 | 5.89681 | 2.533966 |
| DALYs | East Asia | Vitamin A deficiency | 2012 | 3.866383 | 5.744483 | 2.485798 |
| DALYs | East Asia | Vitamin A deficiency | 2013 | 3.798989 | 5.616654 | 2.459421 |
| DALYs | East Asia | Vitamin A deficiency | 2014 | 3.733589 | 5.546303 | 2.398032 |
| DALYs | East Asia | Vitamin A deficiency | 2015 | 3.664134 | 5.454884 | 2.381569 |
| DALYs | East Asia | Vitamin A deficiency | 2016 | 3.583129 | 5.33942 | 2.275599 |
| DALYs | East Asia | Vitamin A deficiency | 2017 | 3.512826 | 5.217483 | 2.269381 |
| DALYs | East Asia | Vitamin A deficiency | 2018 | 3.560735 | 5.299701 | 2.28085 |
| DALYs | East Asia | Vitamin A deficiency | 2019 | 3.752499 | 5.64015 | 2.401719 |
| DALYs | Eastern Sub-Saharan Africa | Dietary iron deficiency | 1990 | 598.4216 | 864.655 | 401.5662 |
| DALYs | Eastern Sub-Saharan Africa | Dietary iron deficiency | 1991 | 598.1525 | 864.4671 | 400.0388 |
| DALYs | Eastern Sub-Saharan Africa | Dietary iron deficiency | 1992 | 598.1735 | 863.2567 | 399.9624 |
| DALYs | Eastern Sub-Saharan Africa | Dietary iron deficiency | 1993 | 597.4042 | 863.9011 | 398.8493 |
| DALYs | Eastern Sub-Saharan Africa | Dietary iron deficiency | 1994 | 595.2747 | 859.3318 | 396.7105 |
| DALYs | Eastern Sub-Saharan Africa | Dietary iron deficiency | 1995 | 592.3753 | 856.3827 | 394.6953 |
| DALYs | Eastern Sub-Saharan Africa | Dietary iron deficiency | 1996 | 587.8928 | 848.1116 | 391.7148 |
| DALYs | Eastern Sub-Saharan Africa | Dietary iron deficiency | 1997 | 581.5542 | 838.4155 | 386.8407 |
| DALYs | Eastern Sub-Saharan Africa | Dietary iron deficiency | 1998 | 574.5278 | 825.985 | 382.1272 |
| DALYs | Eastern Sub-Saharan Africa | Dietary iron deficiency | 1999 | 567.7067 | 816.8906 | 377.6436 |
| DALYs | Eastern Sub-Saharan Africa | Dietary iron deficiency | 2000 | 562.034 | 808.2428 | 373.2533 |
| DALYs | Eastern Sub-Saharan Africa | Dietary iron deficiency | 2001 | 557.0883 | 804.807 | 370.5365 |
| DALYs | Eastern Sub-Saharan Africa | Dietary iron deficiency | 2002 | 551.9286 | 800.6979 | 367.6417 |
| DALYs | Eastern Sub-Saharan Africa | Dietary iron deficiency | 2003 | 546.7639 | 792.4038 | 364.5218 |
| DALYs | Eastern Sub-Saharan Africa | Dietary iron deficiency | 2004 | 542.1165 | 784.5904 | 360.9227 |
| DALYs | Eastern Sub-Saharan Africa | Dietary iron deficiency | 2005 | 538.5031 | 780.2297 | 358.5261 |
| DALYs | Eastern Sub-Saharan Africa | Dietary iron deficiency | 2006 | 534.7805 | 775.3471 | 354.7261 |
| DALYs | Eastern Sub-Saharan Africa | Dietary iron deficiency | 2007 | 530.2275 | 769.0354 | 351.2014 |
| DALYs | Eastern Sub-Saharan Africa | Dietary iron deficiency | 2008 | 525.5853 | 761.3541 | 348.4059 |
| DALYs | Eastern Sub-Saharan Africa | Dietary iron deficiency | 2009 | 521.7019 | 757.243 | 345.3415 |
| DALYs | Eastern Sub-Saharan Africa | Dietary iron deficiency | 2010 | 519.3345 | 753.9429 | 344.0594 |
| DALYs | Eastern Sub-Saharan Africa | Dietary iron deficiency | 2011 | 518.4122 | 752.6843 | 343.7896 |
| DALYs | Eastern Sub-Saharan Africa | Dietary iron deficiency | 2012 | 518.0799 | 752.8246 | 343.6141 |
| DALYs | Eastern Sub-Saharan Africa | Dietary iron deficiency | 2013 | 517.9179 | 752.9962 | 344.4272 |
| DALYs | Eastern Sub-Saharan Africa | Dietary iron deficiency | 2014 | 517.6605 | 753.5446 | 344.6874 |
| DALYs | Eastern Sub-Saharan Africa | Dietary iron deficiency | 2015 | 516.79 | 753.0942 | 345.6584 |
| DALYs | Eastern Sub-Saharan Africa | Dietary iron deficiency | 2016 | 512.3168 | 748.0547 | 341.1891 |
| DALYs | Eastern Sub-Saharan Africa | Dietary iron deficiency | 2017 | 506.3157 | 741.0279 | 337.0981 |
| DALYs | Eastern Sub-Saharan Africa | Dietary iron deficiency | 2018 | 501.1456 | 729.053 | 334.8979 |
| DALYs | Eastern Sub-Saharan Africa | Dietary iron deficiency | 2019 | 495.2482 | 718.5852 | 330.9654 |
| DALYs | Eastern Sub-Saharan Africa | Iodine deficiency | 1990 | 91.98595 | 157.0157 | 52.73523 |
| DALYs | Eastern Sub-Saharan Africa | Iodine deficiency | 1991 | 93.62999 | 159.4672 | 53.5835 |
| DALYs | Eastern Sub-Saharan Africa | Iodine deficiency | 1992 | 95.07052 | 161.7192 | 54.5897 |
| DALYs | Eastern Sub-Saharan Africa | Iodine deficiency | 1993 | 96.28559 | 163.1615 | 55.44241 |
| DALYs | Eastern Sub-Saharan Africa | Iodine deficiency | 1994 | 96.86261 | 163.5488 | 55.56147 |
| DALYs | Eastern Sub-Saharan Africa | Iodine deficiency | 1995 | 96.88541 | 163.8006 | 55.48811 |
| DALYs | Eastern Sub-Saharan Africa | Iodine deficiency | 1996 | 96.10725 | 162.5402 | 55.38138 |
| DALYs | Eastern Sub-Saharan Africa | Iodine deficiency | 1997 | 94.6517 | 159.5231 | 54.43937 |
| DALYs | Eastern Sub-Saharan Africa | Iodine deficiency | 1998 | 92.99438 | 157.4584 | 53.63614 |
| DALYs | Eastern Sub-Saharan Africa | Iodine deficiency | 1999 | 91.38657 | 154.6163 | 52.96834 |
| DALYs | Eastern Sub-Saharan Africa | Iodine deficiency | 2000 | 90.246 | 153.182 | 51.76724 |
| DALYs | Eastern Sub-Saharan Africa | Iodine deficiency | 2001 | 89.92403 | 152.1903 | 51.84243 |
| DALYs | Eastern Sub-Saharan Africa | Iodine deficiency | 2002 | 90.2261 | 153.5548 | 51.67466 |
| DALYs | Eastern Sub-Saharan Africa | Iodine deficiency | 2003 | 90.77874 | 154.6771 | 52.10609 |
| DALYs | Eastern Sub-Saharan Africa | Iodine deficiency | 2004 | 91.22485 | 156.5791 | 52.03583 |
| DALYs | Eastern Sub-Saharan Africa | Iodine deficiency | 2005 | 91.22544 | 156.6301 | 51.75805 |
| DALYs | Eastern Sub-Saharan Africa | Iodine deficiency | 2006 | 91.11725 | 157.7089 | 51.75536 |
| DALYs | Eastern Sub-Saharan Africa | Iodine deficiency | 2007 | 91.21316 | 158.1298 | 51.21833 |
| DALYs | Eastern Sub-Saharan Africa | Iodine deficiency | 2008 | 91.29492 | 159.0227 | 50.96946 |
| DALYs | Eastern Sub-Saharan Africa | Iodine deficiency | 2009 | 91.10825 | 159.6444 | 50.37491 |
| DALYs | Eastern Sub-Saharan Africa | Iodine deficiency | 2010 | 90.39007 | 159.2507 | 49.66154 |
| DALYs | Eastern Sub-Saharan Africa | Iodine deficiency | 2011 | 87.86882 | 155.6932 | 48.13105 |
| DALYs | Eastern Sub-Saharan Africa | Iodine deficiency | 2012 | 83.24352 | 148.2792 | 45.59009 |
| DALYs | Eastern Sub-Saharan Africa | Iodine deficiency | 2013 | 77.6552 | 137.9572 | 42.41223 |
| DALYs | Eastern Sub-Saharan Africa | Iodine deficiency | 2014 | 72.27278 | 128.4556 | 39.44003 |
| DALYs | Eastern Sub-Saharan Africa | Iodine deficiency | 2015 | 68.25685 | 121.3622 | 37.27256 |
| DALYs | Eastern Sub-Saharan Africa | Iodine deficiency | 2016 | 65.38391 | 117.0231 | 35.73519 |
| DALYs | Eastern Sub-Saharan Africa | Iodine deficiency | 2017 | 63.07758 | 112.9637 | 34.47291 |
| DALYs | Eastern Sub-Saharan Africa | Iodine deficiency | 2018 | 61.47458 | 109.8862 | 33.66497 |
| DALYs | Eastern Sub-Saharan Africa | Iodine deficiency | 2019 | 60.38311 | 107.5678 | 33.17218 |
| DALYs | Eastern Sub-Saharan Africa | Vitamin A deficiency | 1990 | 69.26614 | 97.67177 | 47.83032 |
| DALYs | Eastern Sub-Saharan Africa | Vitamin A deficiency | 1991 | 68.6342 | 96.83862 | 47.2069 |
| DALYs | Eastern Sub-Saharan Africa | Vitamin A deficiency | 1992 | 68.16487 | 96.56233 | 46.88211 |
| DALYs | Eastern Sub-Saharan Africa | Vitamin A deficiency | 1993 | 67.81051 | 95.793 | 46.52295 |
| DALYs | Eastern Sub-Saharan Africa | Vitamin A deficiency | 1994 | 67.33958 | 94.87449 | 46.3031 |
| DALYs | Eastern Sub-Saharan Africa | Vitamin A deficiency | 1995 | 66.81379 | 94.42332 | 45.97383 |
| DALYs | Eastern Sub-Saharan Africa | Vitamin A deficiency | 1996 | 66.17264 | 93.57611 | 45.34617 |
| DALYs | Eastern Sub-Saharan Africa | Vitamin A deficiency | 1997 | 65.4373 | 92.19596 | 45.07689 |
| DALYs | Eastern Sub-Saharan Africa | Vitamin A deficiency | 1998 | 64.63067 | 91.09499 | 44.2828 |
| DALYs | Eastern Sub-Saharan Africa | Vitamin A deficiency | 1999 | 63.75983 | 89.75019 | 43.90351 |
| DALYs | Eastern Sub-Saharan Africa | Vitamin A deficiency | 2000 | 62.83388 | 88.47397 | 43.28543 |
| DALYs | Eastern Sub-Saharan Africa | Vitamin A deficiency | 2001 | 61.6863 | 86.83622 | 42.50866 |
| DALYs | Eastern Sub-Saharan Africa | Vitamin A deficiency | 2002 | 60.22947 | 85.02949 | 41.45651 |
| DALYs | Eastern Sub-Saharan Africa | Vitamin A deficiency | 2003 | 58.59485 | 82.56257 | 40.54533 |
| DALYs | Eastern Sub-Saharan Africa | Vitamin A deficiency | 2004 | 56.98251 | 80.42149 | 39.38025 |
| DALYs | Eastern Sub-Saharan Africa | Vitamin A deficiency | 2005 | 55.50792 | 78.48785 | 38.45475 |
| DALYs | Eastern Sub-Saharan Africa | Vitamin A deficiency | 2006 | 54.06403 | 76.46024 | 37.56121 |
| DALYs | Eastern Sub-Saharan Africa | Vitamin A deficiency | 2007 | 52.43467 | 74.13079 | 36.50767 |
| DALYs | Eastern Sub-Saharan Africa | Vitamin A deficiency | 2008 | 50.76103 | 71.67323 | 35.2901 |
| DALYs | Eastern Sub-Saharan Africa | Vitamin A deficiency | 2009 | 49.09881 | 69.47491 | 34.38244 |
| DALYs | Eastern Sub-Saharan Africa | Vitamin A deficiency | 2010 | 47.5862 | 67.10468 | 33.17447 |
| DALYs | Eastern Sub-Saharan Africa | Vitamin A deficiency | 2011 | 46.11598 | 64.74028 | 32.02755 |
| DALYs | Eastern Sub-Saharan Africa | Vitamin A deficiency | 2012 | 44.58682 | 63.13312 | 30.9049 |
| DALYs | Eastern Sub-Saharan Africa | Vitamin A deficiency | 2013 | 43.07281 | 60.88201 | 29.88238 |
| DALYs | Eastern Sub-Saharan Africa | Vitamin A deficiency | 2014 | 41.61235 | 58.76296 | 28.88629 |
| DALYs | Eastern Sub-Saharan Africa | Vitamin A deficiency | 2015 | 40.29178 | 57.23759 | 27.8022 |
| DALYs | Eastern Sub-Saharan Africa | Vitamin A deficiency | 2016 | 38.99505 | 55.20696 | 26.83095 |
| DALYs | Eastern Sub-Saharan Africa | Vitamin A deficiency | 2017 | 37.81301 | 53.62282 | 26.01085 |
| DALYs | Eastern Sub-Saharan Africa | Vitamin A deficiency | 2018 | 36.80377 | 52.10829 | 25.20482 |
| DALYs | Eastern Sub-Saharan Africa | Vitamin A deficiency | 2019 | 35.89595 | 50.84737 | 24.48537 |
| DALYs | Global | Dietary iron deficiency | 1990 | 458.5358 | 657.3919 | 308.8454 |
| DALYs | Global | Dietary iron deficiency | 1991 | 456.6104 | 654.9451 | 307.9288 |
| DALYs | Global | Dietary iron deficiency | 1992 | 454.3528 | 652.0833 | 306.5409 |
| DALYs | Global | Dietary iron deficiency | 1993 | 451.9747 | 649.6023 | 304.7111 |
| DALYs | Global | Dietary iron deficiency | 1994 | 449.5575 | 647.1189 | 303.0456 |
| DALYs | Global | Dietary iron deficiency | 1995 | 447.2263 | 644.213 | 301.0856 |
| DALYs | Global | Dietary iron deficiency | 1996 | 444.7687 | 640.5014 | 298.8206 |
| DALYs | Global | Dietary iron deficiency | 1997 | 441.8936 | 635.5126 | 296.6723 |
| DALYs | Global | Dietary iron deficiency | 1998 | 438.8405 | 630.6256 | 294.9334 |
| DALYs | Global | Dietary iron deficiency | 1999 | 435.836 | 625.461 | 293.498 |
| DALYs | Global | Dietary iron deficiency | 2000 | 433.0119 | 620.5633 | 291.8738 |
| DALYs | Global | Dietary iron deficiency | 2001 | 429.7477 | 615.8804 | 289.3065 |
| DALYs | Global | Dietary iron deficiency | 2002 | 425.6324 | 610.2241 | 286.3729 |
| DALYs | Global | Dietary iron deficiency | 2003 | 421.2226 | 603.4239 | 283.6653 |
| DALYs | Global | Dietary iron deficiency | 2004 | 417.1077 | 597.4486 | 281.265 |
| DALYs | Global | Dietary iron deficiency | 2005 | 413.819 | 592.5996 | 279.5002 |
| DALYs | Global | Dietary iron deficiency | 2006 | 410.8478 | 588.6966 | 277.4061 |
| DALYs | Global | Dietary iron deficiency | 2007 | 407.5949 | 585.6243 | 275.1762 |
| DALYs | Global | Dietary iron deficiency | 2008 | 404.4947 | 581.37 | 272.8702 |
| DALYs | Global | Dietary iron deficiency | 2009 | 401.9274 | 577.916 | 270.5348 |
| DALYs | Global | Dietary iron deficiency | 2010 | 400.1173 | 575.9823 | 269.1323 |
| DALYs | Global | Dietary iron deficiency | 2011 | 398.8693 | 574.1561 | 267.8515 |
| DALYs | Global | Dietary iron deficiency | 2012 | 397.7082 | 572.6893 | 267.1498 |
| DALYs | Global | Dietary iron deficiency | 2013 | 396.6503 | 572 | 266.2916 |
| DALYs | Global | Dietary iron deficiency | 2014 | 395.6864 | 570.5814 | 265.7798 |
| DALYs | Global | Dietary iron deficiency | 2015 | 394.6269 | 569.392 | 265.575 |
| DALYs | Global | Dietary iron deficiency | 2016 | 393.2438 | 568.6337 | 264.8928 |
| DALYs | Global | Dietary iron deficiency | 2017 | 391.229 | 566.2842 | 262.976 |
| DALYs | Global | Dietary iron deficiency | 2018 | 387.9294 | 560.7653 | 260.5189 |
| DALYs | Global | Dietary iron deficiency | 2019 | 383.3828 | 553.4581 | 257.0536 |
| DALYs | Global | Iodine deficiency | 1990 | 46.8457 | 76.01194 | 28.64907 |
| DALYs | Global | Iodine deficiency | 1991 | 45.95789 | 74.52893 | 28.08828 |
| DALYs | Global | Iodine deficiency | 1992 | 45.05073 | 73.02274 | 27.49434 |
| DALYs | Global | Iodine deficiency | 1993 | 44.16685 | 71.56659 | 27.07899 |
| DALYs | Global | Iodine deficiency | 1994 | 43.33879 | 69.96861 | 26.68011 |
| DALYs | Global | Iodine deficiency | 1995 | 42.61684 | 68.78965 | 26.31597 |
| DALYs | Global | Iodine deficiency | 1996 | 41.70715 | 67.27838 | 25.63178 |
| DALYs | Global | Iodine deficiency | 1997 | 40.4808 | 65.1673 | 24.81479 |
| DALYs | Global | Iodine deficiency | 1998 | 39.21751 | 63.2819 | 24.11405 |
| DALYs | Global | Iodine deficiency | 1999 | 38.19529 | 61.56252 | 23.34278 |
| DALYs | Global | Iodine deficiency | 2000 | 37.72022 | 60.80874 | 23.01316 |
| DALYs | Global | Iodine deficiency | 2001 | 37.6739 | 61.08238 | 22.96347 |
| DALYs | Global | Iodine deficiency | 2002 | 37.71193 | 61.20142 | 23.00959 |
| DALYs | Global | Iodine deficiency | 2003 | 37.79068 | 61.92848 | 22.90277 |
| DALYs | Global | Iodine deficiency | 2004 | 37.84418 | 62.24312 | 22.76815 |
| DALYs | Global | Iodine deficiency | 2005 | 37.82222 | 62.38719 | 22.68771 |
| DALYs | Global | Iodine deficiency | 2006 | 37.54692 | 61.90288 | 22.47828 |
| DALYs | Global | Iodine deficiency | 2007 | 36.97186 | 61.11741 | 22.16399 |
| DALYs | Global | Iodine deficiency | 2008 | 36.28425 | 59.94802 | 21.66954 |
| DALYs | Global | Iodine deficiency | 2009 | 35.66836 | 59.10658 | 21.20645 |
| DALYs | Global | Iodine deficiency | 2010 | 35.27527 | 58.63853 | 20.92985 |
| DALYs | Global | Iodine deficiency | 2011 | 34.85968 | 58.05321 | 20.57691 |
| DALYs | Global | Iodine deficiency | 2012 | 34.12412 | 57.30199 | 20.07906 |
| DALYs | Global | Iodine deficiency | 2013 | 33.26057 | 56.40164 | 19.48257 |
| DALYs | Global | Iodine deficiency | 2014 | 32.50861 | 55.50377 | 18.67584 |
| DALYs | Global | Iodine deficiency | 2015 | 32.02073 | 54.88281 | 18.35961 |
| DALYs | Global | Iodine deficiency | 2016 | 31.53436 | 54.21696 | 17.98475 |
| DALYs | Global | Iodine deficiency | 2017 | 31.13762 | 53.72532 | 17.70699 |
| DALYs | Global | Iodine deficiency | 2018 | 30.96511 | 53.42635 | 17.59933 |
| DALYs | Global | Iodine deficiency | 2019 | 30.69678 | 53.12892 | 17.32121 |
| DALYs | Global | Vitamin A deficiency | 1990 | 31.94694 | 45.29591 | 22.11394 |
| DALYs | Global | Vitamin A deficiency | 1991 | 31.31326 | 44.39571 | 21.53861 |
| DALYs | Global | Vitamin A deficiency | 1992 | 30.7401 | 43.6555 | 21.18196 |
| DALYs | Global | Vitamin A deficiency | 1993 | 30.24169 | 42.93707 | 20.92769 |
| DALYs | Global | Vitamin A deficiency | 1994 | 29.82245 | 42.33291 | 20.69718 |
| DALYs | Global | Vitamin A deficiency | 1995 | 29.49179 | 41.8809 | 20.39137 |
| DALYs | Global | Vitamin A deficiency | 1996 | 29.21344 | 41.24975 | 20.20179 |
| DALYs | Global | Vitamin A deficiency | 1997 | 28.96664 | 41.00584 | 20.11696 |
| DALYs | Global | Vitamin A deficiency | 1998 | 28.73335 | 40.52078 | 19.92239 |
| DALYs | Global | Vitamin A deficiency | 1999 | 28.51669 | 40.17551 | 19.70865 |
| DALYs | Global | Vitamin A deficiency | 2000 | 28.30732 | 39.7922 | 19.51693 |
| DALYs | Global | Vitamin A deficiency | 2001 | 28.00485 | 39.29583 | 19.29745 |
| DALYs | Global | Vitamin A deficiency | 2002 | 27.55924 | 38.72778 | 19.01497 |
| DALYs | Global | Vitamin A deficiency | 2003 | 27.02159 | 37.85088 | 18.59125 |
| DALYs | Global | Vitamin A deficiency | 2004 | 26.46691 | 37.05647 | 18.22877 |
| DALYs | Global | Vitamin A deficiency | 2005 | 25.95727 | 36.33537 | 17.89107 |
| DALYs | Global | Vitamin A deficiency | 2006 | 25.40737 | 35.60461 | 17.4639 |
| DALYs | Global | Vitamin A deficiency | 2007 | 24.73674 | 34.72389 | 16.98899 |
| DALYs | Global | Vitamin A deficiency | 2008 | 23.99892 | 33.69267 | 16.4356 |
| DALYs | Global | Vitamin A deficiency | 2009 | 23.25055 | 32.52642 | 15.86199 |
| DALYs | Global | Vitamin A deficiency | 2010 | 22.56024 | 31.51853 | 15.31278 |
| DALYs | Global | Vitamin A deficiency | 2011 | 21.8422 | 30.43526 | 14.81723 |
| DALYs | Global | Vitamin A deficiency | 2012 | 21.01923 | 29.3521 | 14.26685 |
| DALYs | Global | Vitamin A deficiency | 2013 | 20.17551 | 28.0892 | 13.75199 |
| DALYs | Global | Vitamin A deficiency | 2014 | 19.37836 | 27.1074 | 13.16061 |
| DALYs | Global | Vitamin A deficiency | 2015 | 18.68561 | 26.29797 | 12.69819 |
| DALYs | Global | Vitamin A deficiency | 2016 | 18.07453 | 25.22324 | 12.2689 |
| DALYs | Global | Vitamin A deficiency | 2017 | 17.56915 | 24.45465 | 11.97243 |
| DALYs | Global | Vitamin A deficiency | 2018 | 17.19203 | 23.91902 | 11.70354 |
| DALYs | Global | Vitamin A deficiency | 2019 | 16.91073 | 23.47367 | 11.52898 |
| DALYs | High-income Asia Pacific | Dietary iron deficiency | 1990 | 272.64 | 404.5728 | 178.0188 |
| DALYs | High-income Asia Pacific | Dietary iron deficiency | 1991 | 262.5826 | 391.0876 | 171.0937 |
| DALYs | High-income Asia Pacific | Dietary iron deficiency | 1992 | 252.6743 | 379.0743 | 163.4715 |
| DALYs | High-income Asia Pacific | Dietary iron deficiency | 1993 | 243.1269 | 363.8472 | 156.9373 |
| DALYs | High-income Asia Pacific | Dietary iron deficiency | 1994 | 234.1596 | 350.7274 | 150.5181 |
| DALYs | High-income Asia Pacific | Dietary iron deficiency | 1995 | 225.9142 | 337.7096 | 145.4795 |
| DALYs | High-income Asia Pacific | Dietary iron deficiency | 1996 | 217.9373 | 328.741 | 140.8 |
| DALYs | High-income Asia Pacific | Dietary iron deficiency | 1997 | 209.8935 | 316.4133 | 136.1209 |
| DALYs | High-income Asia Pacific | Dietary iron deficiency | 1998 | 202.0074 | 303.4852 | 130.7693 |
| DALYs | High-income Asia Pacific | Dietary iron deficiency | 1999 | 194.5671 | 293.4194 | 124.9259 |
| DALYs | High-income Asia Pacific | Dietary iron deficiency | 2000 | 187.7259 | 285.343 | 121.3994 |
| DALYs | High-income Asia Pacific | Dietary iron deficiency | 2001 | 180.9696 | 275.2975 | 116.905 |
| DALYs | High-income Asia Pacific | Dietary iron deficiency | 2002 | 173.9587 | 266.6564 | 111.4674 |
| DALYs | High-income Asia Pacific | Dietary iron deficiency | 2003 | 167.1943 | 255.7227 | 107.1676 |
| DALYs | High-income Asia Pacific | Dietary iron deficiency | 2004 | 161.253 | 247.9644 | 102.1132 |
| DALYs | High-income Asia Pacific | Dietary iron deficiency | 2005 | 156.5859 | 241.4824 | 98.53119 |
| DALYs | High-income Asia Pacific | Dietary iron deficiency | 2006 | 152.8894 | 234.7305 | 95.68807 |
| DALYs | High-income Asia Pacific | Dietary iron deficiency | 2007 | 149.6012 | 229.5495 | 93.58128 |
| DALYs | High-income Asia Pacific | Dietary iron deficiency | 2008 | 146.6797 | 227.3605 | 91.16794 |
| DALYs | High-income Asia Pacific | Dietary iron deficiency | 2009 | 144.1334 | 223.1748 | 89.92794 |
| DALYs | High-income Asia Pacific | Dietary iron deficiency | 2010 | 141.9159 | 218.9635 | 88.63867 |
| DALYs | High-income Asia Pacific | Dietary iron deficiency | 2011 | 139.8961 | 215.5418 | 87.49624 |
| DALYs | High-income Asia Pacific | Dietary iron deficiency | 2012 | 137.8601 | 212.0126 | 86.42774 |
| DALYs | High-income Asia Pacific | Dietary iron deficiency | 2013 | 136.0068 | 208.7602 | 84.13554 |
| DALYs | High-income Asia Pacific | Dietary iron deficiency | 2014 | 134.3647 | 207.2851 | 83.53844 |
| DALYs | High-income Asia Pacific | Dietary iron deficiency | 2015 | 133.1115 | 207.1342 | 83.25132 |
| DALYs | High-income Asia Pacific | Dietary iron deficiency | 2016 | 132.046 | 205.0773 | 82.93157 |
| DALYs | High-income Asia Pacific | Dietary iron deficiency | 2017 | 131.2211 | 202.2094 | 82.30622 |
| DALYs | High-income Asia Pacific | Dietary iron deficiency | 2018 | 130.6648 | 201.3017 | 81.87554 |
| DALYs | High-income Asia Pacific | Dietary iron deficiency | 2019 | 130.1655 | 199.8149 | 80.8179 |
| DALYs | High-income Asia Pacific | Iodine deficiency | 1990 | 3.277357 | 6.204451 | 1.488712 |
| DALYs | High-income Asia Pacific | Iodine deficiency | 1991 | 3.247061 | 6.161308 | 1.466775 |
| DALYs | High-income Asia Pacific | Iodine deficiency | 1992 | 3.216217 | 6.085107 | 1.455694 |
| DALYs | High-income Asia Pacific | Iodine deficiency | 1993 | 3.187084 | 6.015644 | 1.445193 |
| DALYs | High-income Asia Pacific | Iodine deficiency | 1994 | 3.160936 | 6.009299 | 1.432992 |
| DALYs | High-income Asia Pacific | Iodine deficiency | 1995 | 3.138385 | 5.92601 | 1.417431 |
| DALYs | High-income Asia Pacific | Iodine deficiency | 1996 | 3.116988 | 5.90718 | 1.410505 |
| DALYs | High-income Asia Pacific | Iodine deficiency | 1997 | 3.099666 | 5.885036 | 1.402424 |
| DALYs | High-income Asia Pacific | Iodine deficiency | 1998 | 3.08082 | 5.872362 | 1.395686 |
| DALYs | High-income Asia Pacific | Iodine deficiency | 1999 | 3.061829 | 5.831836 | 1.389706 |
| DALYs | High-income Asia Pacific | Iodine deficiency | 2000 | 3.040865 | 5.80712 | 1.367286 |
| DALYs | High-income Asia Pacific | Iodine deficiency | 2001 | 3.018653 | 5.79989 | 1.360582 |
| DALYs | High-income Asia Pacific | Iodine deficiency | 2002 | 2.991791 | 5.712076 | 1.350825 |
| DALYs | High-income Asia Pacific | Iodine deficiency | 2003 | 2.965731 | 5.639638 | 1.343713 |
| DALYs | High-income Asia Pacific | Iodine deficiency | 2004 | 2.93784 | 5.602723 | 1.332474 |
| DALYs | High-income Asia Pacific | Iodine deficiency | 2005 | 2.914334 | 5.514474 | 1.315174 |
| DALYs | High-income Asia Pacific | Iodine deficiency | 2006 | 2.893797 | 5.486584 | 1.309325 |
| DALYs | High-income Asia Pacific | Iodine deficiency | 2007 | 2.876066 | 5.456204 | 1.299803 |
| DALYs | High-income Asia Pacific | Iodine deficiency | 2008 | 2.856789 | 5.401346 | 1.292337 |
| DALYs | High-income Asia Pacific | Iodine deficiency | 2009 | 2.838197 | 5.382353 | 1.272342 |
| DALYs | High-income Asia Pacific | Iodine deficiency | 2010 | 2.82144 | 5.345769 | 1.274483 |
| DALYs | High-income Asia Pacific | Iodine deficiency | 2011 | 2.80258 | 5.297512 | 1.264813 |
| DALYs | High-income Asia Pacific | Iodine deficiency | 2012 | 2.783328 | 5.291951 | 1.263986 |
| DALYs | High-income Asia Pacific | Iodine deficiency | 2013 | 2.764708 | 5.248757 | 1.256462 |
| DALYs | High-income Asia Pacific | Iodine deficiency | 2014 | 2.748567 | 5.204501 | 1.244126 |
| DALYs | High-income Asia Pacific | Iodine deficiency | 2015 | 2.739246 | 5.190959 | 1.235099 |
| DALYs | High-income Asia Pacific | Iodine deficiency | 2016 | 2.739865 | 5.21782 | 1.239911 |
| DALYs | High-income Asia Pacific | Iodine deficiency | 2017 | 2.739978 | 5.23921 | 1.2346 |
| DALYs | High-income Asia Pacific | Iodine deficiency | 2018 | 2.733548 | 5.19966 | 1.24056 |
| DALYs | High-income Asia Pacific | Iodine deficiency | 2019 | 2.720066 | 5.214325 | 1.224449 |
| DALYs | High-income Asia Pacific | Vitamin A deficiency | 1990 | 1.14234 | 1.844934 | 0.640777 |
| DALYs | High-income Asia Pacific | Vitamin A deficiency | 1991 | 1.061953 | 1.704025 | 0.598304 |
| DALYs | High-income Asia Pacific | Vitamin A deficiency | 1992 | 0.985777 | 1.615549 | 0.56091 |
| DALYs | High-income Asia Pacific | Vitamin A deficiency | 1993 | 0.91638 | 1.498479 | 0.521423 |
| DALYs | High-income Asia Pacific | Vitamin A deficiency | 1994 | 0.853027 | 1.437171 | 0.477502 |
| DALYs | High-income Asia Pacific | Vitamin A deficiency | 1995 | 0.800763 | 1.321854 | 0.440802 |
| DALYs | High-income Asia Pacific | Vitamin A deficiency | 1996 | 0.751894 | 1.232273 | 0.413776 |
| DALYs | High-income Asia Pacific | Vitamin A deficiency | 1997 | 0.70451 | 1.171491 | 0.382018 |
| DALYs | High-income Asia Pacific | Vitamin A deficiency | 1998 | 0.66098 | 1.091078 | 0.360224 |
| DALYs | High-income Asia Pacific | Vitamin A deficiency | 1999 | 0.622379 | 1.051332 | 0.342059 |
| DALYs | High-income Asia Pacific | Vitamin A deficiency | 2000 | 0.594689 | 0.990362 | 0.322637 |
| DALYs | High-income Asia Pacific | Vitamin A deficiency | 2001 | 0.577007 | 0.95774 | 0.316216 |
| DALYs | High-income Asia Pacific | Vitamin A deficiency | 2002 | 0.563892 | 0.94742 | 0.313157 |
| DALYs | High-income Asia Pacific | Vitamin A deficiency | 2003 | 0.552492 | 0.90818 | 0.305507 |
| DALYs | High-income Asia Pacific | Vitamin A deficiency | 2004 | 0.541434 | 0.894461 | 0.300862 |
| DALYs | High-income Asia Pacific | Vitamin A deficiency | 2005 | 0.52958 | 0.884507 | 0.286881 |
| DALYs | High-income Asia Pacific | Vitamin A deficiency | 2006 | 0.517547 | 0.868926 | 0.281091 |
| DALYs | High-income Asia Pacific | Vitamin A deficiency | 2007 | 0.502968 | 0.836779 | 0.278388 |
| DALYs | High-income Asia Pacific | Vitamin A deficiency | 2008 | 0.488258 | 0.803515 | 0.263114 |
| DALYs | High-income Asia Pacific | Vitamin A deficiency | 2009 | 0.474961 | 0.784399 | 0.25591 |
| DALYs | High-income Asia Pacific | Vitamin A deficiency | 2010 | 0.464104 | 0.793433 | 0.246768 |
| DALYs | High-income Asia Pacific | Vitamin A deficiency | 2011 | 0.449689 | 0.760531 | 0.244518 |
| DALYs | High-income Asia Pacific | Vitamin A deficiency | 2012 | 0.434606 | 0.728179 | 0.2356 |
| DALYs | High-income Asia Pacific | Vitamin A deficiency | 2013 | 0.41956 | 0.704927 | 0.230985 |
| DALYs | High-income Asia Pacific | Vitamin A deficiency | 2014 | 0.404245 | 0.685843 | 0.212498 |
| DALYs | High-income Asia Pacific | Vitamin A deficiency | 2015 | 0.392456 | 0.676356 | 0.204398 |
| DALYs | High-income Asia Pacific | Vitamin A deficiency | 2016 | 0.383252 | 0.663426 | 0.206672 |
| DALYs | High-income Asia Pacific | Vitamin A deficiency | 2017 | 0.373671 | 0.650101 | 0.207534 |
| DALYs | High-income Asia Pacific | Vitamin A deficiency | 2018 | 0.364523 | 0.630503 | 0.18992 |
| DALYs | High-income Asia Pacific | Vitamin A deficiency | 2019 | 0.353912 | 0.596833 | 0.187514 |
| DALYs | High-income North America | Dietary iron deficiency | 1990 | 64.48399 | 99.49497 | 40.41451 |
| DALYs | High-income North America | Dietary iron deficiency | 1991 | 63.44327 | 96.61904 | 39.66897 |
| DALYs | High-income North America | Dietary iron deficiency | 1992 | 62.2779 | 94.24796 | 38.85504 |
| DALYs | High-income North America | Dietary iron deficiency | 1993 | 61.03564 | 92.74497 | 38.0445 |
| DALYs | High-income North America | Dietary iron deficiency | 1994 | 59.76576 | 91.63581 | 37.17465 |
| DALYs | High-income North America | Dietary iron deficiency | 1995 | 58.50101 | 89.95821 | 36.35982 |
| DALYs | High-income North America | Dietary iron deficiency | 1996 | 56.83555 | 86.82103 | 35.47092 |
| DALYs | High-income North America | Dietary iron deficiency | 1997 | 54.58085 | 83.12689 | 34.06738 |
| DALYs | High-income North America | Dietary iron deficiency | 1998 | 52.16952 | 79.18748 | 32.74386 |
| DALYs | High-income North America | Dietary iron deficiency | 1999 | 50.07734 | 75.47009 | 31.42693 |
| DALYs | High-income North America | Dietary iron deficiency | 2000 | 48.73855 | 73.45967 | 30.73406 |
| DALYs | High-income North America | Dietary iron deficiency | 2001 | 48.02491 | 72.15497 | 30.48124 |
| DALYs | High-income North America | Dietary iron deficiency | 2002 | 47.54169 | 71.44603 | 30.54003 |
| DALYs | High-income North America | Dietary iron deficiency | 2003 | 47.28695 | 71.31456 | 30.31132 |
| DALYs | High-income North America | Dietary iron deficiency | 2004 | 47.28909 | 71.94413 | 30.28136 |
| DALYs | High-income North America | Dietary iron deficiency | 2005 | 47.54198 | 72.97281 | 30.24988 |
| DALYs | High-income North America | Dietary iron deficiency | 2006 | 48.43072 | 74.7203 | 30.73196 |
| DALYs | High-income North America | Dietary iron deficiency | 2007 | 50.06558 | 76.98259 | 31.90691 |
| DALYs | High-income North America | Dietary iron deficiency | 2008 | 52.04116 | 80.08468 | 33.04423 |
| DALYs | High-income North America | Dietary iron deficiency | 2009 | 54.0131 | 83.15632 | 34.26676 |
| DALYs | High-income North America | Dietary iron deficiency | 2010 | 55.60819 | 85.8721 | 35.15701 |
| DALYs | High-income North America | Dietary iron deficiency | 2011 | 57.02453 | 88.3322 | 36.26826 |
| DALYs | High-income North America | Dietary iron deficiency | 2012 | 58.56761 | 91.1043 | 36.99405 |
| DALYs | High-income North America | Dietary iron deficiency | 2013 | 60.05089 | 93.87359 | 37.5665 |
| DALYs | High-income North America | Dietary iron deficiency | 2014 | 61.30068 | 96.00528 | 37.94343 |
| DALYs | High-income North America | Dietary iron deficiency | 2015 | 62.12985 | 96.78851 | 38.519 |
| DALYs | High-income North America | Dietary iron deficiency | 2016 | 62.52541 | 98.21165 | 38.61318 |
| DALYs | High-income North America | Dietary iron deficiency | 2017 | 62.67693 | 98.90655 | 38.73411 |
| DALYs | High-income North America | Dietary iron deficiency | 2018 | 62.73586 | 97.93631 | 38.96172 |
| DALYs | High-income North America | Dietary iron deficiency | 2019 | 62.71599 | 97.70534 | 38.96364 |
| DALYs | High-income North America | Iodine deficiency | 1990 | 2.455343 | 4.684265 | 1.114831 |
| DALYs | High-income North America | Iodine deficiency | 1991 | 2.453026 | 4.681896 | 1.113739 |
| DALYs | High-income North America | Iodine deficiency | 1992 | 2.451985 | 4.648189 | 1.116293 |
| DALYs | High-income North America | Iodine deficiency | 1993 | 2.451474 | 4.626103 | 1.108269 |
| DALYs | High-income North America | Iodine deficiency | 1994 | 2.451294 | 4.63695 | 1.11572 |
| DALYs | High-income North America | Iodine deficiency | 1995 | 2.452461 | 4.652048 | 1.116733 |
| DALYs | High-income North America | Iodine deficiency | 1996 | 2.456323 | 4.676034 | 1.106282 |
| DALYs | High-income North America | Iodine deficiency | 1997 | 2.463192 | 4.668187 | 1.117132 |
| DALYs | High-income North America | Iodine deficiency | 1998 | 2.471195 | 4.663614 | 1.122034 |
| DALYs | High-income North America | Iodine deficiency | 1999 | 2.47721 | 4.706906 | 1.119682 |
| DALYs | High-income North America | Iodine deficiency | 2000 | 2.479272 | 4.692975 | 1.121577 |
| DALYs | High-income North America | Iodine deficiency | 2001 | 2.477286 | 4.698131 | 1.122909 |
| DALYs | High-income North America | Iodine deficiency | 2002 | 2.473957 | 4.701103 | 1.120492 |
| DALYs | High-income North America | Iodine deficiency | 2003 | 2.468743 | 4.689712 | 1.118618 |
| DALYs | High-income North America | Iodine deficiency | 2004 | 2.463274 | 4.657956 | 1.119036 |
| DALYs | High-income North America | Iodine deficiency | 2005 | 2.461449 | 4.692886 | 1.11585 |
| DALYs | High-income North America | Iodine deficiency | 2006 | 2.4607 | 4.675736 | 1.114343 |
| DALYs | High-income North America | Iodine deficiency | 2007 | 2.459771 | 4.699755 | 1.1092 |
| DALYs | High-income North America | Iodine deficiency | 2008 | 2.459451 | 4.668294 | 1.110406 |
| DALYs | High-income North America | Iodine deficiency | 2009 | 2.459256 | 4.661756 | 1.117987 |
| DALYs | High-income North America | Iodine deficiency | 2010 | 2.457192 | 4.647321 | 1.117332 |
| DALYs | High-income North America | Iodine deficiency | 2011 | 2.454049 | 4.651369 | 1.111309 |
| DALYs | High-income North America | Iodine deficiency | 2012 | 2.448379 | 4.6329 | 1.108699 |
| DALYs | High-income North America | Iodine deficiency | 2013 | 2.442126 | 4.64668 | 1.110301 |
| DALYs | High-income North America | Iodine deficiency | 2014 | 2.437236 | 4.638441 | 1.106256 |
| DALYs | High-income North America | Iodine deficiency | 2015 | 2.434654 | 4.62929 | 1.10366 |
| DALYs | High-income North America | Iodine deficiency | 2016 | 2.436022 | 4.625079 | 1.109709 |
| DALYs | High-income North America | Iodine deficiency | 2017 | 2.438605 | 4.631343 | 1.105205 |
| DALYs | High-income North America | Iodine deficiency | 2018 | 2.434674 | 4.622087 | 1.107228 |
| DALYs | High-income North America | Iodine deficiency | 2019 | 2.425731 | 4.607667 | 1.10089 |
| DALYs | High-income North America | Vitamin A deficiency | 1990 | 0.293651 | 0.494205 | 0.162804 |
| DALYs | High-income North America | Vitamin A deficiency | 1991 | 0.28533 | 0.473277 | 0.155687 |
| DALYs | High-income North America | Vitamin A deficiency | 1992 | 0.277202 | 0.456561 | 0.152275 |
| DALYs | High-income North America | Vitamin A deficiency | 1993 | 0.268348 | 0.443429 | 0.146537 |
| DALYs | High-income North America | Vitamin A deficiency | 1994 | 0.25981 | 0.428788 | 0.140832 |
| DALYs | High-income North America | Vitamin A deficiency | 1995 | 0.250908 | 0.42407 | 0.134267 |
| DALYs | High-income North America | Vitamin A deficiency | 1996 | 0.240946 | 0.406703 | 0.132969 |
| DALYs | High-income North America | Vitamin A deficiency | 1997 | 0.229228 | 0.375151 | 0.126929 |
| DALYs | High-income North America | Vitamin A deficiency | 1998 | 0.217949 | 0.3602 | 0.117861 |
| DALYs | High-income North America | Vitamin A deficiency | 1999 | 0.208222 | 0.347306 | 0.111261 |
| DALYs | High-income North America | Vitamin A deficiency | 2000 | 0.200277 | 0.335378 | 0.107301 |
| DALYs | High-income North America | Vitamin A deficiency | 2001 | 0.193696 | 0.327341 | 0.107499 |
| DALYs | High-income North America | Vitamin A deficiency | 2002 | 0.186456 | 0.312445 | 0.10273 |
| DALYs | High-income North America | Vitamin A deficiency | 2003 | 0.180176 | 0.302284 | 0.096873 |
| DALYs | High-income North America | Vitamin A deficiency | 2004 | 0.174757 | 0.291275 | 0.09351 |
| DALYs | High-income North America | Vitamin A deficiency | 2005 | 0.171092 | 0.280626 | 0.092378 |
| DALYs | High-income North America | Vitamin A deficiency | 2006 | 0.169531 | 0.273783 | 0.092451 |
| DALYs | High-income North America | Vitamin A deficiency | 2007 | 0.170152 | 0.281028 | 0.091194 |
| DALYs | High-income North America | Vitamin A deficiency | 2008 | 0.171263 | 0.280643 | 0.091763 |
| DALYs | High-income North America | Vitamin A deficiency | 2009 | 0.171406 | 0.277918 | 0.092128 |
| DALYs | High-income North America | Vitamin A deficiency | 2010 | 0.170891 | 0.280063 | 0.089367 |
| DALYs | High-income North America | Vitamin A deficiency | 2011 | 0.169129 | 0.282943 | 0.092249 |
| DALYs | High-income North America | Vitamin A deficiency | 2012 | 0.167281 | 0.278575 | 0.089898 |
| DALYs | High-income North America | Vitamin A deficiency | 2013 | 0.164577 | 0.273574 | 0.087481 |
| DALYs | High-income North America | Vitamin A deficiency | 2014 | 0.162248 | 0.274985 | 0.086167 |
| DALYs | High-income North America | Vitamin A deficiency | 2015 | 0.159594 | 0.271816 | 0.084101 |
| DALYs | High-income North America | Vitamin A deficiency | 2016 | 0.156464 | 0.268997 | 0.082949 |
| DALYs | High-income North America | Vitamin A deficiency | 2017 | 0.153417 | 0.263979 | 0.078076 |
| DALYs | High-income North America | Vitamin A deficiency | 2018 | 0.151461 | 0.272208 | 0.076578 |
| DALYs | High-income North America | Vitamin A deficiency | 2019 | 0.149326 | 0.26754 | 0.075083 |
| DALYs | North Africa and Middle East | Dietary iron deficiency | 1990 | 346.8886 | 509.8469 | 233.9637 |
| DALYs | North Africa and Middle East | Dietary iron deficiency | 1991 | 341.3982 | 502.2967 | 229.6514 |
| DALYs | North Africa and Middle East | Dietary iron deficiency | 1992 | 335.8445 | 492.3181 | 225.5847 |
| DALYs | North Africa and Middle East | Dietary iron deficiency | 1993 | 330.2419 | 485.0727 | 221.3587 |
| DALYs | North Africa and Middle East | Dietary iron deficiency | 1994 | 324.6547 | 474.5061 | 217.3249 |
| DALYs | North Africa and Middle East | Dietary iron deficiency | 1995 | 319.0638 | 467.6517 | 213.1513 |
| DALYs | North Africa and Middle East | Dietary iron deficiency | 1996 | 313.2295 | 457.7944 | 209.2254 |
| DALYs | North Africa and Middle East | Dietary iron deficiency | 1997 | 306.9834 | 450.067 | 205.3871 |
| DALYs | North Africa and Middle East | Dietary iron deficiency | 1998 | 300.5825 | 441.0954 | 200.5981 |
| DALYs | North Africa and Middle East | Dietary iron deficiency | 1999 | 294.5464 | 433.9421 | 196.6166 |
| DALYs | North Africa and Middle East | Dietary iron deficiency | 2000 | 289.1702 | 427.7771 | 192.9785 |
| DALYs | North Africa and Middle East | Dietary iron deficiency | 2001 | 284.0807 | 417.3116 | 190.0429 |
| DALYs | North Africa and Middle East | Dietary iron deficiency | 2002 | 278.926 | 408.5432 | 186.525 |
| DALYs | North Africa and Middle East | Dietary iron deficiency | 2003 | 274.028 | 399.4512 | 183.7026 |
| DALYs | North Africa and Middle East | Dietary iron deficiency | 2004 | 269.8279 | 394.1924 | 180.2865 |
| DALYs | North Africa and Middle East | Dietary iron deficiency | 2005 | 266.5147 | 389.9405 | 179.0346 |
| DALYs | North Africa and Middle East | Dietary iron deficiency | 2006 | 263.758 | 385.3478 | 176.493 |
| DALYs | North Africa and Middle East | Dietary iron deficiency | 2007 | 260.9672 | 382.9493 | 174.2334 |
| DALYs | North Africa and Middle East | Dietary iron deficiency | 2008 | 258.2108 | 377.9633 | 171.7929 |
| DALYs | North Africa and Middle East | Dietary iron deficiency | 2009 | 255.6426 | 373.754 | 170.4425 |
| DALYs | North Africa and Middle East | Dietary iron deficiency | 2010 | 253.2949 | 371.8518 | 169.228 |
| DALYs | North Africa and Middle East | Dietary iron deficiency | 2011 | 251.1161 | 368.5708 | 167.0667 |
| DALYs | North Africa and Middle East | Dietary iron deficiency | 2012 | 248.8928 | 365.8975 | 165.2001 |
| DALYs | North Africa and Middle East | Dietary iron deficiency | 2013 | 246.619 | 363.7287 | 163.8104 |
| DALYs | North Africa and Middle East | Dietary iron deficiency | 2014 | 244.198 | 359.4369 | 161.5296 |
| DALYs | North Africa and Middle East | Dietary iron deficiency | 2015 | 241.486 | 357.4313 | 159.6986 |
| DALYs | North Africa and Middle East | Dietary iron deficiency | 2016 | 237.8401 | 352.9954 | 157.5076 |
| DALYs | North Africa and Middle East | Dietary iron deficiency | 2017 | 233.5625 | 347.5653 | 155.1086 |
| DALYs | North Africa and Middle East | Dietary iron deficiency | 2018 | 228.9638 | 341.3779 | 151.7109 |
| DALYs | North Africa and Middle East | Dietary iron deficiency | 2019 | 223.9235 | 332.6483 | 147.5601 |
| DALYs | North Africa and Middle East | Iodine deficiency | 1990 | 38.34971 | 57.53505 | 23.7243 |
| DALYs | North Africa and Middle East | Iodine deficiency | 1991 | 38.83311 | 57.60977 | 23.98297 |
| DALYs | North Africa and Middle East | Iodine deficiency | 1992 | 39.29803 | 58.54425 | 24.42933 |
| DALYs | North Africa and Middle East | Iodine deficiency | 1993 | 39.59349 | 59.18353 | 24.6935 |
| DALYs | North Africa and Middle East | Iodine deficiency | 1994 | 39.6158 | 59.36378 | 24.7754 |
| DALYs | North Africa and Middle East | Iodine deficiency | 1995 | 39.46715 | 58.80899 | 24.51047 |
| DALYs | North Africa and Middle East | Iodine deficiency | 1996 | 39.12267 | 58.35538 | 24.15911 |
| DALYs | North Africa and Middle East | Iodine deficiency | 1997 | 38.59916 | 57.36762 | 24.07163 |
| DALYs | North Africa and Middle East | Iodine deficiency | 1998 | 37.92823 | 56.64173 | 23.53214 |
| DALYs | North Africa and Middle East | Iodine deficiency | 1999 | 37.13005 | 55.73849 | 23.17494 |
| DALYs | North Africa and Middle East | Iodine deficiency | 2000 | 36.32232 | 54.33011 | 22.55986 |
| DALYs | North Africa and Middle East | Iodine deficiency | 2001 | 35.35875 | 53.60086 | 21.82617 |
| DALYs | North Africa and Middle East | Iodine deficiency | 2002 | 34.09242 | 51.25882 | 20.92055 |
| DALYs | North Africa and Middle East | Iodine deficiency | 2003 | 32.63459 | 49.56966 | 20.04368 |
| DALYs | North Africa and Middle East | Iodine deficiency | 2004 | 31.21789 | 47.35936 | 19.30868 |
| DALYs | North Africa and Middle East | Iodine deficiency | 2005 | 30.0587 | 45.69656 | 18.41848 |
| DALYs | North Africa and Middle East | Iodine deficiency | 2006 | 28.94024 | 44.38606 | 17.89191 |
| DALYs | North Africa and Middle East | Iodine deficiency | 2007 | 27.75644 | 42.46957 | 17.26578 |
| DALYs | North Africa and Middle East | Iodine deficiency | 2008 | 26.5755 | 40.64562 | 16.60288 |
| DALYs | North Africa and Middle East | Iodine deficiency | 2009 | 25.50352 | 39.14774 | 15.69599 |
| DALYs | North Africa and Middle East | Iodine deficiency | 2010 | 24.6545 | 37.89725 | 15.19724 |
| DALYs | North Africa and Middle East | Iodine deficiency | 2011 | 23.91111 | 36.80055 | 14.76006 |
| DALYs | North Africa and Middle East | Iodine deficiency | 2012 | 23.13803 | 35.43428 | 14.29924 |
| DALYs | North Africa and Middle East | Iodine deficiency | 2013 | 22.38747 | 34.42159 | 13.66354 |
| DALYs | North Africa and Middle East | Iodine deficiency | 2014 | 21.76412 | 33.40075 | 13.35877 |
| DALYs | North Africa and Middle East | Iodine deficiency | 2015 | 21.34372 | 33.06952 | 13.13788 |
| DALYs | North Africa and Middle East | Iodine deficiency | 2016 | 21.15992 | 32.76289 | 12.86941 |
| DALYs | North Africa and Middle East | Iodine deficiency | 2017 | 21.20677 | 32.96019 | 12.98763 |
| DALYs | North Africa and Middle East | Iodine deficiency | 2018 | 21.37236 | 33.01187 | 13.06126 |
| DALYs | North Africa and Middle East | Iodine deficiency | 2019 | 21.61594 | 33.4199 | 13.25562 |
| DALYs | North Africa and Middle East | Vitamin A deficiency | 1990 | 20.06976 | 28.80601 | 13.53071 |
| DALYs | North Africa and Middle East | Vitamin A deficiency | 1991 | 19.55403 | 28.07917 | 13.17683 |
| DALYs | North Africa and Middle East | Vitamin A deficiency | 1992 | 19.16559 | 27.50246 | 12.96546 |
| DALYs | North Africa and Middle East | Vitamin A deficiency | 1993 | 18.77131 | 27.05191 | 12.64789 |
| DALYs | North Africa and Middle East | Vitamin A deficiency | 1994 | 18.3766 | 26.46463 | 12.38412 |
| DALYs | North Africa and Middle East | Vitamin A deficiency | 1995 | 18.04864 | 25.99335 | 12.1451 |
| DALYs | North Africa and Middle East | Vitamin A deficiency | 1996 | 17.78072 | 25.56035 | 12.01092 |
| DALYs | North Africa and Middle East | Vitamin A deficiency | 1997 | 17.56199 | 25.22356 | 11.85999 |
| DALYs | North Africa and Middle East | Vitamin A deficiency | 1998 | 17.34558 | 24.93554 | 11.63848 |
| DALYs | North Africa and Middle East | Vitamin A deficiency | 1999 | 17.13012 | 24.71977 | 11.58764 |
| DALYs | North Africa and Middle East | Vitamin A deficiency | 2000 | 16.95779 | 24.3762 | 11.35839 |
| DALYs | North Africa and Middle East | Vitamin A deficiency | 2001 | 16.79603 | 24.26843 | 11.30636 |
| DALYs | North Africa and Middle East | Vitamin A deficiency | 2002 | 16.61242 | 23.93242 | 11.14 |
| DALYs | North Africa and Middle East | Vitamin A deficiency | 2003 | 16.35391 | 23.43672 | 10.90555 |
| DALYs | North Africa and Middle East | Vitamin A deficiency | 2004 | 16.06687 | 22.89165 | 10.70875 |
| DALYs | North Africa and Middle East | Vitamin A deficiency | 2005 | 15.77911 | 22.49314 | 10.53524 |
| DALYs | North Africa and Middle East | Vitamin A deficiency | 2006 | 15.37554 | 21.81837 | 10.35438 |
| DALYs | North Africa and Middle East | Vitamin A deficiency | 2007 | 14.88991 | 21.10586 | 9.981874 |
| DALYs | North Africa and Middle East | Vitamin A deficiency | 2008 | 14.35195 | 20.36206 | 9.606604 |
| DALYs | North Africa and Middle East | Vitamin A deficiency | 2009 | 13.79493 | 19.55729 | 9.249771 |
| DALYs | North Africa and Middle East | Vitamin A deficiency | 2010 | 13.32464 | 18.9137 | 8.914423 |
| DALYs | North Africa and Middle East | Vitamin A deficiency | 2011 | 12.88597 | 18.33433 | 8.554324 |
| DALYs | North Africa and Middle East | Vitamin A deficiency | 2012 | 12.44014 | 17.4926 | 8.290528 |
| DALYs | North Africa and Middle East | Vitamin A deficiency | 2013 | 11.99391 | 16.95291 | 7.982837 |
| DALYs | North Africa and Middle East | Vitamin A deficiency | 2014 | 11.55648 | 16.40221 | 7.679277 |
| DALYs | North Africa and Middle East | Vitamin A deficiency | 2015 | 11.16825 | 15.81688 | 7.39441 |
| DALYs | North Africa and Middle East | Vitamin A deficiency | 2016 | 10.85177 | 15.39282 | 7.198864 |
| DALYs | North Africa and Middle East | Vitamin A deficiency | 2017 | 10.58904 | 15.04478 | 7.071419 |
| DALYs | North Africa and Middle East | Vitamin A deficiency | 2018 | 10.36269 | 14.70013 | 6.927337 |
| DALYs | North Africa and Middle East | Vitamin A deficiency | 2019 | 10.16594 | 14.32115 | 6.740012 |
| DALYs | Oceania | Dietary iron deficiency | 1990 | 562.6458 | 811.2329 | 368.5276 |
| DALYs | Oceania | Dietary iron deficiency | 1991 | 559.3316 | 808.9662 | 369.5383 |
| DALYs | Oceania | Dietary iron deficiency | 1992 | 556.2487 | 803.1612 | 369.2567 |
| DALYs | Oceania | Dietary iron deficiency | 1993 | 552.95 | 797.2031 | 365.6931 |
| DALYs | Oceania | Dietary iron deficiency | 1994 | 549.439 | 791.4464 | 362.9154 |
| DALYs | Oceania | Dietary iron deficiency | 1995 | 545.9447 | 788.8736 | 359.8458 |
| DALYs | Oceania | Dietary iron deficiency | 1996 | 542.1114 | 781.9837 | 358.1311 |
| DALYs | Oceania | Dietary iron deficiency | 1997 | 537.4116 | 776.0271 | 355.8048 |
| DALYs | Oceania | Dietary iron deficiency | 1998 | 532.5322 | 769.9984 | 354.397 |
| DALYs | Oceania | Dietary iron deficiency | 1999 | 528.2134 | 766.4806 | 352.2846 |
| DALYs | Oceania | Dietary iron deficiency | 2000 | 525.7321 | 764.0726 | 349.2665 |
| DALYs | Oceania | Dietary iron deficiency | 2001 | 525.0381 | 766.368 | 350.1607 |
| DALYs | Oceania | Dietary iron deficiency | 2002 | 525.429 | 763.3353 | 352.7384 |
| DALYs | Oceania | Dietary iron deficiency | 2003 | 526.207 | 762.7848 | 353.1451 |
| DALYs | Oceania | Dietary iron deficiency | 2004 | 527.0125 | 763.6184 | 353.5296 |
| DALYs | Oceania | Dietary iron deficiency | 2005 | 527.3546 | 762.6526 | 354.6532 |
| DALYs | Oceania | Dietary iron deficiency | 2006 | 527.4179 | 763.8687 | 354.6927 |
| DALYs | Oceania | Dietary iron deficiency | 2007 | 527.5986 | 763.8079 | 352.4081 |
| DALYs | Oceania | Dietary iron deficiency | 2008 | 527.5174 | 766.6995 | 351.7499 |
| DALYs | Oceania | Dietary iron deficiency | 2009 | 527.3347 | 764.1234 | 347.4511 |
| DALYs | Oceania | Dietary iron deficiency | 2010 | 526.7434 | 762.6031 | 346.6224 |
| DALYs | Oceania | Dietary iron deficiency | 2011 | 525.8659 | 757.773 | 349.1013 |
| DALYs | Oceania | Dietary iron deficiency | 2012 | 524.4258 | 755.5867 | 347.4969 |
| DALYs | Oceania | Dietary iron deficiency | 2013 | 522.7324 | 754.394 | 342.7912 |
| DALYs | Oceania | Dietary iron deficiency | 2014 | 520.7214 | 754.4155 | 344.2202 |
| DALYs | Oceania | Dietary iron deficiency | 2015 | 518.5292 | 750.8982 | 343.5439 |
| DALYs | Oceania | Dietary iron deficiency | 2016 | 514.7247 | 745.4374 | 340.6425 |
| DALYs | Oceania | Dietary iron deficiency | 2017 | 510.4169 | 735.0952 | 336.4481 |
| DALYs | Oceania | Dietary iron deficiency | 2018 | 507.1058 | 734.1811 | 332.6088 |
| DALYs | Oceania | Dietary iron deficiency | 2019 | 503.6413 | 729.6086 | 327.9347 |
| DALYs | Oceania | Iodine deficiency | 1990 | 4.1712 | 6.506499 | 2.584033 |
| DALYs | Oceania | Iodine deficiency | 1991 | 4.185929 | 6.581185 | 2.576043 |
| DALYs | Oceania | Iodine deficiency | 1992 | 4.198761 | 6.547246 | 2.618182 |
| DALYs | Oceania | Iodine deficiency | 1993 | 4.207823 | 6.539646 | 2.628288 |
| DALYs | Oceania | Iodine deficiency | 1994 | 4.213877 | 6.501591 | 2.615367 |
| DALYs | Oceania | Iodine deficiency | 1995 | 4.206813 | 6.578237 | 2.586205 |
| DALYs | Oceania | Iodine deficiency | 1996 | 4.197181 | 6.499945 | 2.548964 |
| DALYs | Oceania | Iodine deficiency | 1997 | 4.178117 | 6.458294 | 2.565275 |
| DALYs | Oceania | Iodine deficiency | 1998 | 4.149191 | 6.465737 | 2.534304 |
| DALYs | Oceania | Iodine deficiency | 1999 | 4.122754 | 6.322084 | 2.495367 |
| DALYs | Oceania | Iodine deficiency | 2000 | 4.098872 | 6.418299 | 2.456896 |
| DALYs | Oceania | Iodine deficiency | 2001 | 4.089131 | 6.320692 | 2.479967 |
| DALYs | Oceania | Iodine deficiency | 2002 | 4.07329 | 6.298654 | 2.458432 |
| DALYs | Oceania | Iodine deficiency | 2003 | 4.052078 | 6.241624 | 2.488383 |
| DALYs | Oceania | Iodine deficiency | 2004 | 4.033935 | 6.273373 | 2.482802 |
| DALYs | Oceania | Iodine deficiency | 2005 | 4.009205 | 6.177945 | 2.427842 |
| DALYs | Oceania | Iodine deficiency | 2006 | 3.931792 | 6.048515 | 2.383902 |
| DALYs | Oceania | Iodine deficiency | 2007 | 3.826932 | 5.880948 | 2.354599 |
| DALYs | Oceania | Iodine deficiency | 2008 | 3.670493 | 5.690332 | 2.255275 |
| DALYs | Oceania | Iodine deficiency | 2009 | 3.493083 | 5.364629 | 2.131569 |
| DALYs | Oceania | Iodine deficiency | 2010 | 3.326714 | 5.145719 | 2.023217 |
| DALYs | Oceania | Iodine deficiency | 2011 | 3.140552 | 4.907673 | 1.948817 |
| DALYs | Oceania | Iodine deficiency | 2012 | 2.911982 | 4.584566 | 1.814053 |
| DALYs | Oceania | Iodine deficiency | 2013 | 2.678232 | 4.20771 | 1.646042 |
| DALYs | Oceania | Iodine deficiency | 2014 | 2.471288 | 3.94501 | 1.473278 |
| DALYs | Oceania | Iodine deficiency | 2015 | 2.324438 | 3.756801 | 1.358573 |
| DALYs | Oceania | Iodine deficiency | 2016 | 2.215816 | 3.639042 | 1.297396 |
| DALYs | Oceania | Iodine deficiency | 2017 | 2.140208 | 3.4701 | 1.259948 |
| DALYs | Oceania | Iodine deficiency | 2018 | 2.103034 | 3.441205 | 1.233682 |
| DALYs | Oceania | Iodine deficiency | 2019 | 2.070227 | 3.363903 | 1.212134 |
| DALYs | Oceania | Vitamin A deficiency | 1990 | 26.96118 | 39.25605 | 17.73155 |
| DALYs | Oceania | Vitamin A deficiency | 1991 | 25.93942 | 37.34209 | 17.07097 |
| DALYs | Oceania | Vitamin A deficiency | 1992 | 25.02522 | 36.03654 | 16.54259 |
| DALYs | Oceania | Vitamin A deficiency | 1993 | 24.19469 | 34.8215 | 15.85803 |
| DALYs | Oceania | Vitamin A deficiency | 1994 | 23.52028 | 33.58675 | 15.42953 |
| DALYs | Oceania | Vitamin A deficiency | 1995 | 23.03027 | 33.19241 | 15.19187 |
| DALYs | Oceania | Vitamin A deficiency | 1996 | 22.63251 | 32.65195 | 14.78264 |
| DALYs | Oceania | Vitamin A deficiency | 1997 | 22.27142 | 32.41426 | 14.74577 |
| DALYs | Oceania | Vitamin A deficiency | 1998 | 21.94692 | 31.94303 | 14.44361 |
| DALYs | Oceania | Vitamin A deficiency | 1999 | 21.70304 | 31.84626 | 14.11018 |
| DALYs | Oceania | Vitamin A deficiency | 2000 | 21.62321 | 31.71236 | 14.19557 |
| DALYs | Oceania | Vitamin A deficiency | 2001 | 21.93189 | 31.99934 | 14.27054 |
| DALYs | Oceania | Vitamin A deficiency | 2002 | 22.69473 | 33.30085 | 14.65852 |
| DALYs | Oceania | Vitamin A deficiency | 2003 | 23.59967 | 34.36873 | 15.14487 |
| DALYs | Oceania | Vitamin A deficiency | 2004 | 24.36081 | 35.85565 | 15.64252 |
| DALYs | Oceania | Vitamin A deficiency | 2005 | 24.70784 | 36.36506 | 16.15378 |
| DALYs | Oceania | Vitamin A deficiency | 2006 | 24.54165 | 36.36654 | 15.87504 |
| DALYs | Oceania | Vitamin A deficiency | 2007 | 24.16634 | 35.24385 | 15.63316 |
| DALYs | Oceania | Vitamin A deficiency | 2008 | 23.63909 | 34.45027 | 15.19505 |
| DALYs | Oceania | Vitamin A deficiency | 2009 | 23.08963 | 33.48093 | 14.77766 |
| DALYs | Oceania | Vitamin A deficiency | 2010 | 22.5821 | 33.06632 | 14.67786 |
| DALYs | Oceania | Vitamin A deficiency | 2011 | 22.13182 | 31.8523 | 14.0018 |
| DALYs | Oceania | Vitamin A deficiency | 2012 | 21.67359 | 31.4899 | 13.88798 |
| DALYs | Oceania | Vitamin A deficiency | 2013 | 21.17863 | 30.68124 | 13.53777 |
| DALYs | Oceania | Vitamin A deficiency | 2014 | 20.66334 | 29.94345 | 13.18569 |
| DALYs | Oceania | Vitamin A deficiency | 2015 | 20.16808 | 29.54798 | 12.71379 |
| DALYs | Oceania | Vitamin A deficiency | 2016 | 19.60987 | 28.42905 | 12.28964 |
| DALYs | Oceania | Vitamin A deficiency | 2017 | 19.0886 | 27.66664 | 11.91351 |
| DALYs | Oceania | Vitamin A deficiency | 2018 | 18.68913 | 27.45525 | 11.41871 |
| DALYs | Oceania | Vitamin A deficiency | 2019 | 18.30426 | 27.04038 | 11.18823 |
| DALYs | South Asia | Dietary iron deficiency | 1990 | 1001.463 | 1414.308 | 680.6326 |
| DALYs | South Asia | Dietary iron deficiency | 1991 | 998.8041 | 1411.303 | 677.8389 |
| DALYs | South Asia | Dietary iron deficiency | 1992 | 996.2907 | 1404.954 | 675.0444 |
| DALYs | South Asia | Dietary iron deficiency | 1993 | 993.9498 | 1398.724 | 674.9814 |
| DALYs | South Asia | Dietary iron deficiency | 1994 | 991.8051 | 1403.558 | 674.2738 |
| DALYs | South Asia | Dietary iron deficiency | 1995 | 989.892 | 1404.183 | 674.1296 |
| DALYs | South Asia | Dietary iron deficiency | 1996 | 989.1586 | 1401.85 | 673.5281 |
| DALYs | South Asia | Dietary iron deficiency | 1997 | 989.7788 | 1402.381 | 674.7009 |
| DALYs | South Asia | Dietary iron deficiency | 1998 | 990.4523 | 1403.817 | 675.1735 |
| DALYs | South Asia | Dietary iron deficiency | 1999 | 989.9676 | 1401.202 | 674.959 |
| DALYs | South Asia | Dietary iron deficiency | 2000 | 987.0619 | 1399.116 | 672.8801 |
| DALYs | South Asia | Dietary iron deficiency | 2001 | 980.7058 | 1391.533 | 668.1287 |
| DALYs | South Asia | Dietary iron deficiency | 2002 | 971.5384 | 1380.384 | 661.6315 |
| DALYs | South Asia | Dietary iron deficiency | 2003 | 960.8807 | 1365.024 | 653.537 |
| DALYs | South Asia | Dietary iron deficiency | 2004 | 950.215 | 1351.173 | 644.8141 |
| DALYs | South Asia | Dietary iron deficiency | 2005 | 940.9295 | 1340.229 | 637.6781 |
| DALYs | South Asia | Dietary iron deficiency | 2006 | 933.0836 | 1329.458 | 631.4223 |
| DALYs | South Asia | Dietary iron deficiency | 2007 | 925.8248 | 1319.57 | 626.6759 |
| DALYs | South Asia | Dietary iron deficiency | 2008 | 918.8762 | 1310.377 | 620.893 |
| DALYs | South Asia | Dietary iron deficiency | 2009 | 911.9299 | 1300.668 | 615.3473 |
| DALYs | South Asia | Dietary iron deficiency | 2010 | 904.6689 | 1290.366 | 609.9555 |
| DALYs | South Asia | Dietary iron deficiency | 2011 | 896.5994 | 1278.045 | 605.6523 |
| DALYs | South Asia | Dietary iron deficiency | 2012 | 887.7788 | 1266.741 | 600.3544 |
| DALYs | South Asia | Dietary iron deficiency | 2013 | 878.8709 | 1254.98 | 595.4801 |
| DALYs | South Asia | Dietary iron deficiency | 2014 | 870.4945 | 1242.917 | 590.7411 |
| DALYs | South Asia | Dietary iron deficiency | 2015 | 863.3498 | 1233.808 | 585.6202 |
| DALYs | South Asia | Dietary iron deficiency | 2016 | 857.4855 | 1225.732 | 582.075 |
| DALYs | South Asia | Dietary iron deficiency | 2017 | 850.7303 | 1217.948 | 578.3207 |
| DALYs | South Asia | Dietary iron deficiency | 2018 | 840.2186 | 1201.546 | 571.2608 |
| DALYs | South Asia | Dietary iron deficiency | 2019 | 826.1593 | 1180.578 | 561.9452 |
| DALYs | South Asia | Iodine deficiency | 1990 | 150.0122 | 240.6187 | 93.95805 |
| DALYs | South Asia | Iodine deficiency | 1991 | 144.8787 | 231.4925 | 90.7118 |
| DALYs | South Asia | Iodine deficiency | 1992 | 139.7386 | 222.4177 | 87.86887 |
| DALYs | South Asia | Iodine deficiency | 1993 | 134.7258 | 214.5635 | 84.76177 |
| DALYs | South Asia | Iodine deficiency | 1994 | 129.9881 | 205.5997 | 81.86922 |
| DALYs | South Asia | Iodine deficiency | 1995 | 125.6425 | 199.1178 | 79.2303 |
| DALYs | South Asia | Iodine deficiency | 1996 | 120.6699 | 191.4619 | 76.44655 |
| DALYs | South Asia | Iodine deficiency | 1997 | 114.6793 | 181.0104 | 72.08082 |
| DALYs | South Asia | Iodine deficiency | 1998 | 108.7051 | 170.883 | 68.73138 |
| DALYs | South Asia | Iodine deficiency | 1999 | 103.8489 | 162.2368 | 65.32064 |
| DALYs | South Asia | Iodine deficiency | 2000 | 101.1624 | 157.4675 | 63.64001 |
| DALYs | South Asia | Iodine deficiency | 2001 | 100.3434 | 156.8273 | 63.304 |
| DALYs | South Asia | Iodine deficiency | 2002 | 100.2021 | 156.3598 | 62.93458 |
| DALYs | South Asia | Iodine deficiency | 2003 | 100.3608 | 156.6051 | 62.74712 |
| DALYs | South Asia | Iodine deficiency | 2004 | 100.3818 | 157.1628 | 62.77236 |
| DALYs | South Asia | Iodine deficiency | 2005 | 99.86885 | 156.8604 | 62.07272 |
| DALYs | South Asia | Iodine deficiency | 2006 | 98.36227 | 155.525 | 61.11535 |
| DALYs | South Asia | Iodine deficiency | 2007 | 96.03581 | 151.5312 | 59.68831 |
| DALYs | South Asia | Iodine deficiency | 2008 | 93.44871 | 147.5776 | 57.89596 |
| DALYs | South Asia | Iodine deficiency | 2009 | 91.1692 | 144.4163 | 56.62876 |
| DALYs | South Asia | Iodine deficiency | 2010 | 89.69462 | 142.7147 | 55.31751 |
| DALYs | South Asia | Iodine deficiency | 2011 | 88.15736 | 140.8175 | 54.18967 |
| DALYs | South Asia | Iodine deficiency | 2012 | 85.61965 | 138.4579 | 52.43045 |
| DALYs | South Asia | Iodine deficiency | 2013 | 82.7513 | 136.0936 | 50.33515 |
| DALYs | South Asia | Iodine deficiency | 2014 | 80.34011 | 134.0768 | 48.08382 |
| DALYs | South Asia | Iodine deficiency | 2015 | 78.76285 | 132.4579 | 46.444 |
| DALYs | South Asia | Iodine deficiency | 2016 | 77.0378 | 130.4675 | 45.11629 |
| DALYs | South Asia | Iodine deficiency | 2017 | 75.43666 | 128.3531 | 44.04779 |
| DALYs | South Asia | Iodine deficiency | 2018 | 74.38411 | 126.8499 | 43.39066 |
| DALYs | South Asia | Iodine deficiency | 2019 | 72.7765 | 125.5206 | 42.18628 |
| DALYs | South Asia | Vitamin A deficiency | 1990 | 55.93855 | 79.55818 | 38.42627 |
| DALYs | South Asia | Vitamin A deficiency | 1991 | 54.72836 | 77.96993 | 37.60906 |
| DALYs | South Asia | Vitamin A deficiency | 1992 | 53.56013 | 75.87277 | 36.59 |
| DALYs | South Asia | Vitamin A deficiency | 1993 | 52.45143 | 73.91695 | 35.87991 |
| DALYs | South Asia | Vitamin A deficiency | 1994 | 51.41334 | 72.80659 | 35.20454 |
| DALYs | South Asia | Vitamin A deficiency | 1995 | 50.46079 | 71.96149 | 34.43104 |
| DALYs | South Asia | Vitamin A deficiency | 1996 | 49.55546 | 70.60035 | 33.90361 |
| DALYs | South Asia | Vitamin A deficiency | 1997 | 48.67887 | 69.30902 | 33.26103 |
| DALYs | South Asia | Vitamin A deficiency | 1998 | 47.7914 | 68.10648 | 32.64944 |
| DALYs | South Asia | Vitamin A deficiency | 1999 | 46.89146 | 67.10769 | 32.04489 |
| DALYs | South Asia | Vitamin A deficiency | 2000 | 45.96121 | 65.59718 | 31.33612 |
| DALYs | South Asia | Vitamin A deficiency | 2001 | 44.75438 | 64.37709 | 30.6491 |
| DALYs | South Asia | Vitamin A deficiency | 2002 | 43.20938 | 61.72754 | 29.4139 |
| DALYs | South Asia | Vitamin A deficiency | 2003 | 41.49743 | 59.01701 | 28.37816 |
| DALYs | South Asia | Vitamin A deficiency | 2004 | 39.82625 | 56.85247 | 27.07159 |
| DALYs | South Asia | Vitamin A deficiency | 2005 | 38.3486 | 54.59959 | 26.02286 |
| DALYs | South Asia | Vitamin A deficiency | 2006 | 36.99647 | 52.49261 | 25.08686 |
| DALYs | South Asia | Vitamin A deficiency | 2007 | 35.61101 | 50.71374 | 24.02106 |
| DALYs | South Asia | Vitamin A deficiency | 2008 | 34.16936 | 48.65295 | 23.00471 |
| DALYs | South Asia | Vitamin A deficiency | 2009 | 32.72246 | 46.85105 | 21.96583 |
| DALYs | South Asia | Vitamin A deficiency | 2010 | 31.2926 | 44.78179 | 20.90333 |
| DALYs | South Asia | Vitamin A deficiency | 2011 | 29.7639 | 42.46686 | 19.86883 |
| DALYs | South Asia | Vitamin A deficiency | 2012 | 28.07217 | 40.24346 | 18.66954 |
| DALYs | South Asia | Vitamin A deficiency | 2013 | 26.36452 | 37.52101 | 17.50757 |
| DALYs | South Asia | Vitamin A deficiency | 2014 | 24.78451 | 35.37838 | 16.38308 |
| DALYs | South Asia | Vitamin A deficiency | 2015 | 23.46575 | 33.4119 | 15.42208 |
| DALYs | South Asia | Vitamin A deficiency | 2016 | 22.43097 | 31.8066 | 14.79918 |
| DALYs | South Asia | Vitamin A deficiency | 2017 | 21.57776 | 30.68413 | 14.21956 |
| DALYs | South Asia | Vitamin A deficiency | 2018 | 20.85674 | 29.99403 | 13.77003 |
| DALYs | South Asia | Vitamin A deficiency | 2019 | 20.24183 | 29.09586 | 13.41151 |
| DALYs | Southeast Asia | Dietary iron deficiency | 1990 | 483.2708 | 705.0497 | 315.5658 |
| DALYs | Southeast Asia | Dietary iron deficiency | 1991 | 477.9988 | 698.4181 | 312.463 |
| DALYs | Southeast Asia | Dietary iron deficiency | 1992 | 472.128 | 691.2104 | 308.6469 |
| DALYs | Southeast Asia | Dietary iron deficiency | 1993 | 465.9416 | 684.6437 | 304.4757 |
| DALYs | Southeast Asia | Dietary iron deficiency | 1994 | 459.4713 | 674.6743 | 300.717 |
| DALYs | Southeast Asia | Dietary iron deficiency | 1995 | 452.8745 | 666.2874 | 296.6331 |
| DALYs | Southeast Asia | Dietary iron deficiency | 1996 | 445.5238 | 657.4348 | 292.0857 |
| DALYs | Southeast Asia | Dietary iron deficiency | 1997 | 437.0272 | 643.3035 | 286.8199 |
| DALYs | Southeast Asia | Dietary iron deficiency | 1998 | 427.8323 | 628.5442 | 281.0465 |
| DALYs | Southeast Asia | Dietary iron deficiency | 1999 | 418.4424 | 614.8275 | 275.0389 |
| DALYs | Southeast Asia | Dietary iron deficiency | 2000 | 409.3482 | 600.2744 | 269.3459 |
| DALYs | Southeast Asia | Dietary iron deficiency | 2001 | 399.6524 | 586.6794 | 262.7146 |
| DALYs | Southeast Asia | Dietary iron deficiency | 2002 | 388.6792 | 570.0502 | 255.6526 |
| DALYs | Southeast Asia | Dietary iron deficiency | 2003 | 377.1363 | 553.3387 | 248.5794 |
| DALYs | Southeast Asia | Dietary iron deficiency | 2004 | 365.6903 | 535.5914 | 240.1517 |
| DALYs | Southeast Asia | Dietary iron deficiency | 2005 | 355.0513 | 519.6112 | 232.2357 |
| DALYs | Southeast Asia | Dietary iron deficiency | 2006 | 344.2141 | 503.6396 | 224.8727 |
| DALYs | Southeast Asia | Dietary iron deficiency | 2007 | 332.5921 | 488.561 | 217.3494 |
| DALYs | Southeast Asia | Dietary iron deficiency | 2008 | 321.5806 | 472.2753 | 210.4432 |
| DALYs | Southeast Asia | Dietary iron deficiency | 2009 | 312.0986 | 459.5196 | 203.6223 |
| DALYs | Southeast Asia | Dietary iron deficiency | 2010 | 304.4891 | 448.6855 | 198.711 |
| DALYs | Southeast Asia | Dietary iron deficiency | 2011 | 298.4611 | 440.8318 | 194.4952 |
| DALYs | Southeast Asia | Dietary iron deficiency | 2012 | 293.2614 | 433.4927 | 191.5087 |
| DALYs | Southeast Asia | Dietary iron deficiency | 2013 | 288.7755 | 427.9765 | 189.4486 |
| DALYs | Southeast Asia | Dietary iron deficiency | 2014 | 284.8842 | 422.8266 | 187.5181 |
| DALYs | Southeast Asia | Dietary iron deficiency | 2015 | 281.4695 | 419.5248 | 184.0827 |
| DALYs | Southeast Asia | Dietary iron deficiency | 2016 | 278.5133 | 415.0397 | 181.3343 |
| DALYs | Southeast Asia | Dietary iron deficiency | 2017 | 275.3703 | 410.8043 | 179.8661 |
| DALYs | Southeast Asia | Dietary iron deficiency | 2018 | 270.8769 | 403.0647 | 176.8924 |
| DALYs | Southeast Asia | Dietary iron deficiency | 2019 | 265.1488 | 396.2572 | 173.2858 |
| DALYs | Southeast Asia | Iodine deficiency | 1990 | 33.047 | 50.73473 | 21.05484 |
| DALYs | Southeast Asia | Iodine deficiency | 1991 | 32.10815 | 49.01457 | 20.4581 |
| DALYs | Southeast Asia | Iodine deficiency | 1992 | 31.14933 | 47.63236 | 19.86719 |
| DALYs | Southeast Asia | Iodine deficiency | 1993 | 30.23597 | 46.0846 | 19.14404 |
| DALYs | Southeast Asia | Iodine deficiency | 1994 | 29.34082 | 44.67197 | 18.50965 |
| DALYs | Southeast Asia | Iodine deficiency | 1995 | 28.50404 | 43.56862 | 17.95695 |
| DALYs | Southeast Asia | Iodine deficiency | 1996 | 27.93723 | 42.55188 | 17.53231 |
| DALYs | Southeast Asia | Iodine deficiency | 1997 | 27.67549 | 42.47898 | 17.24509 |
| DALYs | Southeast Asia | Iodine deficiency | 1998 | 27.51534 | 42.47188 | 17.13934 |
| DALYs | Southeast Asia | Iodine deficiency | 1999 | 27.22949 | 42.42232 | 16.88349 |
| DALYs | Southeast Asia | Iodine deficiency | 2000 | 26.63422 | 41.66289 | 16.33875 |
| DALYs | Southeast Asia | Iodine deficiency | 2001 | 24.67141 | 38.95141 | 15.48735 |
| DALYs | Southeast Asia | Iodine deficiency | 2002 | 21.22228 | 34.86964 | 12.88004 |
| DALYs | Southeast Asia | Iodine deficiency | 2003 | 17.3765 | 30.61145 | 9.626866 |
| DALYs | Southeast Asia | Iodine deficiency | 2004 | 14.08807 | 26.73373 | 7.072223 |
| DALYs | Southeast Asia | Iodine deficiency | 2005 | 12.41972 | 24.45501 | 5.745806 |
| DALYs | Southeast Asia | Iodine deficiency | 2006 | 12.0341 | 23.49273 | 5.579856 |
| DALYs | Southeast Asia | Iodine deficiency | 2007 | 11.6087 | 22.6923 | 5.386016 |
| DALYs | Southeast Asia | Iodine deficiency | 2008 | 11.18316 | 21.91076 | 5.167076 |
| DALYs | Southeast Asia | Iodine deficiency | 2009 | 10.78112 | 21.04577 | 4.983391 |
| DALYs | Southeast Asia | Iodine deficiency | 2010 | 10.40491 | 20.37074 | 4.819789 |
| DALYs | Southeast Asia | Iodine deficiency | 2011 | 10.55372 | 20.29278 | 5.112961 |
| DALYs | Southeast Asia | Iodine deficiency | 2012 | 11.34991 | 20.94562 | 5.896533 |
| DALYs | Southeast Asia | Iodine deficiency | 2013 | 12.33144 | 21.53001 | 7.021981 |
| DALYs | Southeast Asia | Iodine deficiency | 2014 | 13.14195 | 22.16028 | 7.684337 |
| DALYs | Southeast Asia | Iodine deficiency | 2015 | 13.37677 | 22.34352 | 7.625339 |
| DALYs | Southeast Asia | Iodine deficiency | 2016 | 12.9236 | 21.67438 | 7.386933 |
| DALYs | Southeast Asia | Iodine deficiency | 2017 | 12.44942 | 21.04316 | 6.946679 |
| DALYs | Southeast Asia | Iodine deficiency | 2018 | 12.21342 | 20.57527 | 6.877388 |
| DALYs | Southeast Asia | Iodine deficiency | 2019 | 11.95423 | 20.34309 | 6.675506 |
| DALYs | Southeast Asia | Vitamin A deficiency | 1990 | 38.43171 | 56.14757 | 26.05586 |
| DALYs | Southeast Asia | Vitamin A deficiency | 1991 | 36.85253 | 53.96177 | 25.0565 |
| DALYs | Southeast Asia | Vitamin A deficiency | 1992 | 35.3146 | 51.53648 | 24.05971 |
| DALYs | Southeast Asia | Vitamin A deficiency | 1993 | 33.89007 | 49.69651 | 22.81747 |
| DALYs | Southeast Asia | Vitamin A deficiency | 1994 | 32.58216 | 47.88332 | 21.95648 |
| DALYs | Southeast Asia | Vitamin A deficiency | 1995 | 31.40839 | 46.36388 | 21.05458 |
| DALYs | Southeast Asia | Vitamin A deficiency | 1996 | 30.34027 | 44.44827 | 20.39354 |
| DALYs | Southeast Asia | Vitamin A deficiency | 1997 | 29.32841 | 43.07498 | 19.75718 |
| DALYs | Southeast Asia | Vitamin A deficiency | 1998 | 28.36854 | 41.56235 | 19.02223 |
| DALYs | Southeast Asia | Vitamin A deficiency | 1999 | 27.46157 | 40.24613 | 18.52447 |
| DALYs | Southeast Asia | Vitamin A deficiency | 2000 | 26.60369 | 38.8668 | 17.73358 |
| DALYs | Southeast Asia | Vitamin A deficiency | 2001 | 25.75428 | 37.40853 | 17.19577 |
| DALYs | Southeast Asia | Vitamin A deficiency | 2002 | 24.85166 | 36.15179 | 16.63791 |
| DALYs | Southeast Asia | Vitamin A deficiency | 2003 | 23.95151 | 34.82677 | 16.03779 |
| DALYs | Southeast Asia | Vitamin A deficiency | 2004 | 23.05201 | 33.69481 | 15.44771 |
| DALYs | Southeast Asia | Vitamin A deficiency | 2005 | 22.19856 | 32.55584 | 14.83297 |
| DALYs | Southeast Asia | Vitamin A deficiency | 2006 | 21.31096 | 31.13516 | 14.2952 |
| DALYs | Southeast Asia | Vitamin A deficiency | 2007 | 20.35408 | 29.75747 | 13.59356 |
| DALYs | Southeast Asia | Vitamin A deficiency | 2008 | 19.42628 | 28.39118 | 12.92726 |
| DALYs | Southeast Asia | Vitamin A deficiency | 2009 | 18.57485 | 27.11804 | 12.26951 |
| DALYs | Southeast Asia | Vitamin A deficiency | 2010 | 17.8259 | 26.10785 | 11.84949 |
| DALYs | Southeast Asia | Vitamin A deficiency | 2011 | 17.08171 | 24.89086 | 11.32767 |
| DALYs | Southeast Asia | Vitamin A deficiency | 2012 | 16.29716 | 23.72795 | 10.88589 |
| DALYs | Southeast Asia | Vitamin A deficiency | 2013 | 15.53307 | 22.51017 | 10.3124 |
| DALYs | Southeast Asia | Vitamin A deficiency | 2014 | 14.82677 | 21.43544 | 9.796579 |
| DALYs | Southeast Asia | Vitamin A deficiency | 2015 | 14.24436 | 20.52707 | 9.362144 |
| DALYs | Southeast Asia | Vitamin A deficiency | 2016 | 13.76556 | 19.86093 | 9.070521 |
| DALYs | Southeast Asia | Vitamin A deficiency | 2017 | 13.36011 | 19.30756 | 8.824826 |
| DALYs | Southeast Asia | Vitamin A deficiency | 2018 | 13.00134 | 18.81097 | 8.582502 |
| DALYs | Southeast Asia | Vitamin A deficiency | 2019 | 12.68035 | 18.33643 | 8.350099 |
| DALYs | Southern Latin America | Dietary iron deficiency | 1990 | 224.7149 | 336.4081 | 143.2406 |
| DALYs | Southern Latin America | Dietary iron deficiency | 1991 | 218.7628 | 327.1242 | 139.4 |
| DALYs | Southern Latin America | Dietary iron deficiency | 1992 | 212.9843 | 319.7331 | 136.248 |
| DALYs | Southern Latin America | Dietary iron deficiency | 1993 | 207.3406 | 311.3259 | 132.0514 |
| DALYs | Southern Latin America | Dietary iron deficiency | 1994 | 202.0012 | 303.3671 | 128.3404 |
| DALYs | Southern Latin America | Dietary iron deficiency | 1995 | 196.82 | 296.1601 | 125.4336 |
| DALYs | Southern Latin America | Dietary iron deficiency | 1996 | 191.621 | 288.8058 | 121.7897 |
| DALYs | Southern Latin America | Dietary iron deficiency | 1997 | 186.0882 | 279.4518 | 117.7715 |
| DALYs | Southern Latin America | Dietary iron deficiency | 1998 | 180.7702 | 271.4522 | 114.4711 |
| DALYs | Southern Latin America | Dietary iron deficiency | 1999 | 175.6056 | 263.7171 | 111.144 |
| DALYs | Southern Latin America | Dietary iron deficiency | 2000 | 170.8316 | 254.5676 | 108.5931 |
| DALYs | Southern Latin America | Dietary iron deficiency | 2001 | 165.6673 | 247.4235 | 105.3761 |
| DALYs | Southern Latin America | Dietary iron deficiency | 2002 | 159.7396 | 239.4284 | 101.7967 |
| DALYs | Southern Latin America | Dietary iron deficiency | 2003 | 154.0191 | 232.265 | 97.31214 |
| DALYs | Southern Latin America | Dietary iron deficiency | 2004 | 149.2531 | 226.1415 | 94.4983 |
| DALYs | Southern Latin America | Dietary iron deficiency | 2005 | 146.2106 | 220.7417 | 90.9206 |
| DALYs | Southern Latin America | Dietary iron deficiency | 2006 | 144.2306 | 219.8183 | 89.8708 |
| DALYs | Southern Latin America | Dietary iron deficiency | 2007 | 142.528 | 216.6701 | 88.49665 |
| DALYs | Southern Latin America | Dietary iron deficiency | 2008 | 141.1108 | 215.8546 | 87.71869 |
| DALYs | Southern Latin America | Dietary iron deficiency | 2009 | 139.8126 | 212.6575 | 86.71042 |
| DALYs | Southern Latin America | Dietary iron deficiency | 2010 | 138.7024 | 211.465 | 86.11973 |
| DALYs | Southern Latin America | Dietary iron deficiency | 2011 | 137.8048 | 211.1878 | 85.91716 |
| DALYs | Southern Latin America | Dietary iron deficiency | 2012 | 136.9372 | 212.6916 | 85.31827 |
| DALYs | Southern Latin America | Dietary iron deficiency | 2013 | 136.0871 | 213.2822 | 84.72751 |
| DALYs | Southern Latin America | Dietary iron deficiency | 2014 | 135.2684 | 213.9333 | 84.51285 |
| DALYs | Southern Latin America | Dietary iron deficiency | 2015 | 134.433 | 211.2065 | 84.05296 |
| DALYs | Southern Latin America | Dietary iron deficiency | 2016 | 133.3904 | 208.9045 | 83.78815 |
| DALYs | Southern Latin America | Dietary iron deficiency | 2017 | 132.2908 | 207.3162 | 83.19867 |
| DALYs | Southern Latin America | Dietary iron deficiency | 2018 | 131.2933 | 204.5839 | 81.69602 |
| DALYs | Southern Latin America | Dietary iron deficiency | 2019 | 130.1816 | 202.4831 | 80.28264 |
| DALYs | Southern Latin America | Iodine deficiency | 1990 | 1.951695 | 3.691708 | 0.878696 |
| DALYs | Southern Latin America | Iodine deficiency | 1991 | 1.939709 | 3.604571 | 0.864905 |
| DALYs | Southern Latin America | Iodine deficiency | 1992 | 1.926831 | 3.645411 | 0.853513 |
| DALYs | Southern Latin America | Iodine deficiency | 1993 | 1.910154 | 3.636453 | 0.857687 |
| DALYs | Southern Latin America | Iodine deficiency | 1994 | 1.893934 | 3.625777 | 0.858555 |
| DALYs | Southern Latin America | Iodine deficiency | 1995 | 1.872536 | 3.594225 | 0.83584 |
| DALYs | Southern Latin America | Iodine deficiency | 1996 | 1.847212 | 3.563544 | 0.820207 |
| DALYs | Southern Latin America | Iodine deficiency | 1997 | 1.813095 | 3.444793 | 0.818987 |
| DALYs | Southern Latin America | Iodine deficiency | 1998 | 1.777217 | 3.384599 | 0.792647 |
| DALYs | Southern Latin America | Iodine deficiency | 1999 | 1.743599 | 3.336348 | 0.783634 |
| DALYs | Southern Latin America | Iodine deficiency | 2000 | 1.718754 | 3.267094 | 0.772417 |
| DALYs | Southern Latin America | Iodine deficiency | 2001 | 1.701303 | 3.28066 | 0.778146 |
| DALYs | Southern Latin America | Iodine deficiency | 2002 | 1.68672 | 3.22311 | 0.766545 |
| DALYs | Southern Latin America | Iodine deficiency | 2003 | 1.673457 | 3.172549 | 0.756973 |
| DALYs | Southern Latin America | Iodine deficiency | 2004 | 1.665147 | 3.170369 | 0.751339 |
| DALYs | Southern Latin America | Iodine deficiency | 2005 | 1.649992 | 3.150952 | 0.734932 |
| DALYs | Southern Latin America | Iodine deficiency | 2006 | 1.632884 | 3.110016 | 0.7335 |
| DALYs | Southern Latin America | Iodine deficiency | 2007 | 1.618657 | 3.095829 | 0.737034 |
| DALYs | Southern Latin America | Iodine deficiency | 2008 | 1.598598 | 3.0118 | 0.725903 |
| DALYs | Southern Latin America | Iodine deficiency | 2009 | 1.584412 | 3.035175 | 0.713772 |
| DALYs | Southern Latin America | Iodine deficiency | 2010 | 1.570548 | 3.027683 | 0.698154 |
| DALYs | Southern Latin America | Iodine deficiency | 2011 | 1.553076 | 2.992964 | 0.693566 |
| DALYs | Southern Latin America | Iodine deficiency | 2012 | 1.538404 | 2.95047 | 0.69098 |
| DALYs | Southern Latin America | Iodine deficiency | 2013 | 1.523095 | 2.914981 | 0.684859 |
| DALYs | Southern Latin America | Iodine deficiency | 2014 | 1.510369 | 2.85623 | 0.691681 |
| DALYs | Southern Latin America | Iodine deficiency | 2015 | 1.500923 | 2.881551 | 0.681268 |
| DALYs | Southern Latin America | Iodine deficiency | 2016 | 1.501512 | 2.885452 | 0.676514 |
| DALYs | Southern Latin America | Iodine deficiency | 2017 | 1.50118 | 2.82828 | 0.678395 |
| DALYs | Southern Latin America | Iodine deficiency | 2018 | 1.496443 | 2.812707 | 0.673608 |
| DALYs | Southern Latin America | Iodine deficiency | 2019 | 1.489626 | 2.826719 | 0.673305 |
| DALYs | Southern Latin America | Vitamin A deficiency | 1990 | 10.13657 | 15.64777 | 6.049633 |
| DALYs | Southern Latin America | Vitamin A deficiency | 1991 | 9.856039 | 15.01405 | 5.832276 |
| DALYs | Southern Latin America | Vitamin A deficiency | 1992 | 9.560191 | 14.5497 | 5.706847 |
| DALYs | Southern Latin America | Vitamin A deficiency | 1993 | 9.253585 | 13.94356 | 5.515487 |
| DALYs | Southern Latin America | Vitamin A deficiency | 1994 | 8.979979 | 13.60713 | 5.34758 |
| DALYs | Southern Latin America | Vitamin A deficiency | 1995 | 8.687352 | 13.16961 | 5.250238 |
| DALYs | Southern Latin America | Vitamin A deficiency | 1996 | 8.346164 | 12.66138 | 4.995088 |
| DALYs | Southern Latin America | Vitamin A deficiency | 1997 | 7.936509 | 12.18124 | 4.796826 |
| DALYs | Southern Latin America | Vitamin A deficiency | 1998 | 7.507477 | 11.56176 | 4.499058 |
| DALYs | Southern Latin America | Vitamin A deficiency | 1999 | 7.122107 | 11.07685 | 4.212704 |
| DALYs | Southern Latin America | Vitamin A deficiency | 2000 | 6.827947 | 10.77004 | 4.051127 |
| DALYs | Southern Latin America | Vitamin A deficiency | 2001 | 6.619296 | 10.25087 | 3.957984 |
| DALYs | Southern Latin America | Vitamin A deficiency | 2002 | 6.412682 | 10.05164 | 3.783933 |
| DALYs | Southern Latin America | Vitamin A deficiency | 2003 | 6.237989 | 9.790831 | 3.654491 |
| DALYs | Southern Latin America | Vitamin A deficiency | 2004 | 6.07715 | 9.697343 | 3.584566 |
| DALYs | Southern Latin America | Vitamin A deficiency | 2005 | 5.936927 | 9.358543 | 3.448226 |
| DALYs | Southern Latin America | Vitamin A deficiency | 2006 | 5.833305 | 9.481568 | 3.409884 |
| DALYs | Southern Latin America | Vitamin A deficiency | 2007 | 5.719732 | 9.225138 | 3.293582 |
| DALYs | Southern Latin America | Vitamin A deficiency | 2008 | 5.619229 | 9.278117 | 3.272141 |
| DALYs | Southern Latin America | Vitamin A deficiency | 2009 | 5.503078 | 9.051854 | 3.13778 |
| DALYs | Southern Latin America | Vitamin A deficiency | 2010 | 5.406473 | 9.11681 | 3.07146 |
| DALYs | Southern Latin America | Vitamin A deficiency | 2011 | 5.29384 | 8.830359 | 3.050184 |
| DALYs | Southern Latin America | Vitamin A deficiency | 2012 | 5.145266 | 8.64444 | 3.009716 |
| DALYs | Southern Latin America | Vitamin A deficiency | 2013 | 4.99288 | 8.416339 | 2.947564 |
| DALYs | Southern Latin America | Vitamin A deficiency | 2014 | 4.825017 | 8.021124 | 2.817084 |
| DALYs | Southern Latin America | Vitamin A deficiency | 2015 | 4.686471 | 7.985797 | 2.683216 |
| DALYs | Southern Latin America | Vitamin A deficiency | 2016 | 4.541421 | 7.669345 | 2.598633 |
| DALYs | Southern Latin America | Vitamin A deficiency | 2017 | 4.410681 | 7.327263 | 2.497532 |
| DALYs | Southern Latin America | Vitamin A deficiency | 2018 | 4.315022 | 7.232643 | 2.438038 |
| DALYs | Southern Latin America | Vitamin A deficiency | 2019 | 4.23866 | 7.08663 | 2.372335 |
| DALYs | Southern Sub-Saharan Africa | Dietary iron deficiency | 1990 | 357.2175 | 521.8857 | 235.5124 |
| DALYs | Southern Sub-Saharan Africa | Dietary iron deficiency | 1991 | 352.2539 | 514.5966 | 232.7977 |
| DALYs | Southern Sub-Saharan Africa | Dietary iron deficiency | 1992 | 348.4548 | 509.1099 | 230.0895 |
| DALYs | Southern Sub-Saharan Africa | Dietary iron deficiency | 1993 | 345.6978 | 507.7337 | 227.7664 |
| DALYs | Southern Sub-Saharan Africa | Dietary iron deficiency | 1994 | 344.0432 | 506.5527 | 227.1729 |
| DALYs | Southern Sub-Saharan Africa | Dietary iron deficiency | 1995 | 343.5669 | 502.8448 | 226.3573 |
| DALYs | Southern Sub-Saharan Africa | Dietary iron deficiency | 1996 | 345.2899 | 502.7865 | 227.0199 |
| DALYs | Southern Sub-Saharan Africa | Dietary iron deficiency | 1997 | 349.487 | 507.3265 | 230.117 |
| DALYs | Southern Sub-Saharan Africa | Dietary iron deficiency | 1998 | 354.7948 | 515.6089 | 234.3555 |
| DALYs | Southern Sub-Saharan Africa | Dietary iron deficiency | 1999 | 360.0397 | 525.5083 | 237.7602 |
| DALYs | Southern Sub-Saharan Africa | Dietary iron deficiency | 2000 | 363.8327 | 530.5601 | 239.2488 |
| DALYs | Southern Sub-Saharan Africa | Dietary iron deficiency | 2001 | 367.0976 | 534.047 | 242.5091 |
| DALYs | Southern Sub-Saharan Africa | Dietary iron deficiency | 2002 | 370.8632 | 537.0178 | 245.6898 |
| DALYs | Southern Sub-Saharan Africa | Dietary iron deficiency | 2003 | 374.2312 | 542.3522 | 248.5423 |
| DALYs | Southern Sub-Saharan Africa | Dietary iron deficiency | 2004 | 376.3153 | 540.3523 | 249.7881 |
| DALYs | Southern Sub-Saharan Africa | Dietary iron deficiency | 2005 | 376.0525 | 539.4675 | 249.2424 |
| DALYs | Southern Sub-Saharan Africa | Dietary iron deficiency | 2006 | 372.1865 | 534.2923 | 246.6636 |
| DALYs | Southern Sub-Saharan Africa | Dietary iron deficiency | 2007 | 365.1182 | 523.692 | 240.5184 |
| DALYs | Southern Sub-Saharan Africa | Dietary iron deficiency | 2008 | 356.4612 | 513.803 | 235.4251 |
| DALYs | Southern Sub-Saharan Africa | Dietary iron deficiency | 2009 | 347.6579 | 502.5783 | 231.4267 |
| DALYs | Southern Sub-Saharan Africa | Dietary iron deficiency | 2010 | 340.3548 | 491.4557 | 227.9046 |
| DALYs | Southern Sub-Saharan Africa | Dietary iron deficiency | 2011 | 333.6619 | 478.2302 | 223.5692 |
| DALYs | Southern Sub-Saharan Africa | Dietary iron deficiency | 2012 | 326.1477 | 466.9546 | 218.8106 |
| DALYs | Southern Sub-Saharan Africa | Dietary iron deficiency | 2013 | 318.4293 | 456.4943 | 212.7257 |
| DALYs | Southern Sub-Saharan Africa | Dietary iron deficiency | 2014 | 311.3245 | 446.8089 | 207.9246 |
| DALYs | Southern Sub-Saharan Africa | Dietary iron deficiency | 2015 | 305.3415 | 439.4774 | 204.5837 |
| DALYs | Southern Sub-Saharan Africa | Dietary iron deficiency | 2016 | 300.8479 | 435.9644 | 201.6107 |
| DALYs | Southern Sub-Saharan Africa | Dietary iron deficiency | 2017 | 297.2227 | 430.3552 | 199.4051 |
| DALYs | Southern Sub-Saharan Africa | Dietary iron deficiency | 2018 | 294.0737 | 425.7347 | 197.9991 |
| DALYs | Southern Sub-Saharan Africa | Dietary iron deficiency | 2019 | 291.3118 | 421.3044 | 195.6297 |
| DALYs | Southern Sub-Saharan Africa | Iodine deficiency | 1990 | 21.83228 | 39.1754 | 11.48431 |
| DALYs | Southern Sub-Saharan Africa | Iodine deficiency | 1991 | 20.55501 | 36.65314 | 10.80441 |
| DALYs | Southern Sub-Saharan Africa | Iodine deficiency | 1992 | 19.34342 | 34.8091 | 10.20339 |
| DALYs | Southern Sub-Saharan Africa | Iodine deficiency | 1993 | 18.25682 | 32.72917 | 9.59534 |
| DALYs | Southern Sub-Saharan Africa | Iodine deficiency | 1994 | 17.38891 | 31.25875 | 9.232512 |
| DALYs | Southern Sub-Saharan Africa | Iodine deficiency | 1995 | 16.79361 | 30.67197 | 8.774154 |
| DALYs | Southern Sub-Saharan Africa | Iodine deficiency | 1996 | 16.32014 | 29.74921 | 8.576498 |
| DALYs | Southern Sub-Saharan Africa | Iodine deficiency | 1997 | 15.80306 | 28.58517 | 8.219412 |
| DALYs | Southern Sub-Saharan Africa | Iodine deficiency | 1998 | 15.30317 | 27.78743 | 8.013716 |
| DALYs | Southern Sub-Saharan Africa | Iodine deficiency | 1999 | 14.86968 | 27.20598 | 7.771499 |
| DALYs | Southern Sub-Saharan Africa | Iodine deficiency | 2000 | 14.56364 | 26.50183 | 7.517654 |
| DALYs | Southern Sub-Saharan Africa | Iodine deficiency | 2001 | 14.21855 | 26.06695 | 7.273045 |
| DALYs | Southern Sub-Saharan Africa | Iodine deficiency | 2002 | 13.73305 | 25.42287 | 6.96003 |
| DALYs | Southern Sub-Saharan Africa | Iodine deficiency | 2003 | 13.21954 | 24.81688 | 6.56456 |
| DALYs | Southern Sub-Saharan Africa | Iodine deficiency | 2004 | 12.75835 | 24.20293 | 6.244333 |
| DALYs | Southern Sub-Saharan Africa | Iodine deficiency | 2005 | 12.53385 | 23.7589 | 6.100178 |
| DALYs | Southern Sub-Saharan Africa | Iodine deficiency | 2006 | 12.49738 | 23.74259 | 6.080411 |
| DALYs | Southern Sub-Saharan Africa | Iodine deficiency | 2007 | 12.49991 | 23.86319 | 6.089715 |
| DALYs | Southern Sub-Saharan Africa | Iodine deficiency | 2008 | 12.51343 | 23.9697 | 6.066404 |
| DALYs | Southern Sub-Saharan Africa | Iodine deficiency | 2009 | 12.51679 | 23.99044 | 6.08142 |
| DALYs | Southern Sub-Saharan Africa | Iodine deficiency | 2010 | 12.48772 | 24.03335 | 6.074178 |
| DALYs | Southern Sub-Saharan Africa | Iodine deficiency | 2011 | 12.39622 | 23.65387 | 5.97162 |
| DALYs | Southern Sub-Saharan Africa | Iodine deficiency | 2012 | 12.26108 | 23.4017 | 5.872438 |
| DALYs | Southern Sub-Saharan Africa | Iodine deficiency | 2013 | 12.10969 | 23.26591 | 5.840203 |
| DALYs | Southern Sub-Saharan Africa | Iodine deficiency | 2014 | 11.9748 | 22.99128 | 5.801145 |
| DALYs | Southern Sub-Saharan Africa | Iodine deficiency | 2015 | 11.88922 | 22.79116 | 5.733657 |
| DALYs | Southern Sub-Saharan Africa | Iodine deficiency | 2016 | 11.82184 | 22.75776 | 5.71311 |
| DALYs | Southern Sub-Saharan Africa | Iodine deficiency | 2017 | 11.769 | 22.6825 | 5.627942 |
| DALYs | Southern Sub-Saharan Africa | Iodine deficiency | 2018 | 11.71782 | 22.70016 | 5.629002 |
| DALYs | Southern Sub-Saharan Africa | Iodine deficiency | 2019 | 11.61112 | 22.30443 | 5.567019 |
| DALYs | Southern Sub-Saharan Africa | Vitamin A deficiency | 1990 | 29.884 | 42.73539 | 19.55995 |
| DALYs | Southern Sub-Saharan Africa | Vitamin A deficiency | 1991 | 29.16462 | 41.46685 | 19.25305 |
| DALYs | Southern Sub-Saharan Africa | Vitamin A deficiency | 1992 | 28.45997 | 40.23662 | 18.76875 |
| DALYs | Southern Sub-Saharan Africa | Vitamin A deficiency | 1993 | 27.77854 | 39.41251 | 18.44701 |
| DALYs | Southern Sub-Saharan Africa | Vitamin A deficiency | 1994 | 27.14451 | 39.13593 | 18.04496 |
| DALYs | Southern Sub-Saharan Africa | Vitamin A deficiency | 1995 | 26.61643 | 38.24627 | 17.78626 |
| DALYs | Southern Sub-Saharan Africa | Vitamin A deficiency | 1996 | 26.12099 | 37.84517 | 17.55242 |
| DALYs | Southern Sub-Saharan Africa | Vitamin A deficiency | 1997 | 25.56581 | 37.02266 | 17.20426 |
| DALYs | Southern Sub-Saharan Africa | Vitamin A deficiency | 1998 | 25.01048 | 35.74419 | 16.89427 |
| DALYs | Southern Sub-Saharan Africa | Vitamin A deficiency | 1999 | 24.53527 | 35.4476 | 16.61241 |
| DALYs | Southern Sub-Saharan Africa | Vitamin A deficiency | 2000 | 24.16232 | 34.77901 | 16.38489 |
| DALYs | Southern Sub-Saharan Africa | Vitamin A deficiency | 2001 | 23.95481 | 34.70259 | 16.25921 |
| DALYs | Southern Sub-Saharan Africa | Vitamin A deficiency | 2002 | 23.85533 | 34.53311 | 16.16799 |
| DALYs | Southern Sub-Saharan Africa | Vitamin A deficiency | 2003 | 23.8536 | 34.57365 | 16.18667 |
| DALYs | Southern Sub-Saharan Africa | Vitamin A deficiency | 2004 | 23.79772 | 34.30344 | 16.11968 |
| DALYs | Southern Sub-Saharan Africa | Vitamin A deficiency | 2005 | 23.6458 | 34.08223 | 15.98291 |
| DALYs | Southern Sub-Saharan Africa | Vitamin A deficiency | 2006 | 23.33444 | 33.67562 | 15.73585 |
| DALYs | Southern Sub-Saharan Africa | Vitamin A deficiency | 2007 | 22.94283 | 33.10233 | 15.48645 |
| DALYs | Southern Sub-Saharan Africa | Vitamin A deficiency | 2008 | 22.48683 | 32.39694 | 14.88844 |
| DALYs | Southern Sub-Saharan Africa | Vitamin A deficiency | 2009 | 22.01877 | 31.67074 | 14.74193 |
| DALYs | Southern Sub-Saharan Africa | Vitamin A deficiency | 2010 | 21.57343 | 31.01629 | 14.36064 |
| DALYs | Southern Sub-Saharan Africa | Vitamin A deficiency | 2011 | 21.05116 | 30.42845 | 13.96169 |
| DALYs | Southern Sub-Saharan Africa | Vitamin A deficiency | 2012 | 20.35577 | 29.33031 | 13.47588 |
| DALYs | Southern Sub-Saharan Africa | Vitamin A deficiency | 2013 | 19.57165 | 28.15059 | 12.89129 |
| DALYs | Southern Sub-Saharan Africa | Vitamin A deficiency | 2014 | 18.81919 | 26.96991 | 12.41044 |
| DALYs | Southern Sub-Saharan Africa | Vitamin A deficiency | 2015 | 18.17196 | 26.16157 | 11.98105 |
| DALYs | Southern Sub-Saharan Africa | Vitamin A deficiency | 2016 | 17.6334 | 25.55837 | 11.60245 |
| DALYs | Southern Sub-Saharan Africa | Vitamin A deficiency | 2017 | 17.19222 | 24.7601 | 11.3314 |
| DALYs | Southern Sub-Saharan Africa | Vitamin A deficiency | 2018 | 16.7985 | 24.06615 | 11.18797 |
| DALYs | Southern Sub-Saharan Africa | Vitamin A deficiency | 2019 | 16.41595 | 23.5797 | 10.99755 |
| DALYs | Tropical Latin America | Dietary iron deficiency | 1990 | 472.4722 | 706.6138 | 304.7561 |
| DALYs | Tropical Latin America | Dietary iron deficiency | 1991 | 463.0873 | 685.329 | 297.3151 |
| DALYs | Tropical Latin America | Dietary iron deficiency | 1992 | 453.7722 | 666.5904 | 289.8483 |
| DALYs | Tropical Latin America | Dietary iron deficiency | 1993 | 444.86 | 656.3035 | 283.1597 |
| DALYs | Tropical Latin America | Dietary iron deficiency | 1994 | 436.3394 | 646.4754 | 278.4087 |
| DALYs | Tropical Latin America | Dietary iron deficiency | 1995 | 428.3711 | 634.7537 | 275.094 |
| DALYs | Tropical Latin America | Dietary iron deficiency | 1996 | 420.5749 | 622.1463 | 270.8512 |
| DALYs | Tropical Latin America | Dietary iron deficiency | 1997 | 412.5383 | 609.0545 | 265.9015 |
| DALYs | Tropical Latin America | Dietary iron deficiency | 1998 | 404.6499 | 595.7908 | 261.1578 |
| DALYs | Tropical Latin America | Dietary iron deficiency | 1999 | 397.1165 | 582.1548 | 257.107 |
| DALYs | Tropical Latin America | Dietary iron deficiency | 2000 | 390.2851 | 571.0046 | 253.1866 |
| DALYs | Tropical Latin America | Dietary iron deficiency | 2001 | 383.5859 | 560.5306 | 249.9371 |
| DALYs | Tropical Latin America | Dietary iron deficiency | 2002 | 376.6863 | 548.013 | 244.9701 |
| DALYs | Tropical Latin America | Dietary iron deficiency | 2003 | 370.0876 | 540.2937 | 240.9757 |
| DALYs | Tropical Latin America | Dietary iron deficiency | 2004 | 364.033 | 535.4569 | 237.9465 |
| DALYs | Tropical Latin America | Dietary iron deficiency | 2005 | 359.1458 | 528.0612 | 234.671 |
| DALYs | Tropical Latin America | Dietary iron deficiency | 2006 | 354.9743 | 521.6575 | 230.7809 |
| DALYs | Tropical Latin America | Dietary iron deficiency | 2007 | 351.0749 | 519.6787 | 227.6832 |
| DALYs | Tropical Latin America | Dietary iron deficiency | 2008 | 347.3207 | 516.5578 | 224.4527 |
| DALYs | Tropical Latin America | Dietary iron deficiency | 2009 | 343.645 | 514.7007 | 222.6121 |
| DALYs | Tropical Latin America | Dietary iron deficiency | 2010 | 340.1202 | 509.7966 | 219.9852 |
| DALYs | Tropical Latin America | Dietary iron deficiency | 2011 | 336.4454 | 504.186 | 216.824 |
| DALYs | Tropical Latin America | Dietary iron deficiency | 2012 | 332.5637 | 496.8968 | 213.7789 |
| DALYs | Tropical Latin America | Dietary iron deficiency | 2013 | 328.7351 | 493.6348 | 211.4184 |
| DALYs | Tropical Latin America | Dietary iron deficiency | 2014 | 325.2442 | 492.5263 | 210.6337 |
| DALYs | Tropical Latin America | Dietary iron deficiency | 2015 | 322.2136 | 490.5251 | 209.3392 |
| DALYs | Tropical Latin America | Dietary iron deficiency | 2016 | 319.9821 | 490.6918 | 207.5545 |
| DALYs | Tropical Latin America | Dietary iron deficiency | 2017 | 317.6599 | 485.2967 | 205.0806 |
| DALYs | Tropical Latin America | Dietary iron deficiency | 2018 | 313.8441 | 478.8759 | 202.0612 |
| DALYs | Tropical Latin America | Dietary iron deficiency | 2019 | 308.6589 | 467.3954 | 198.1752 |
| DALYs | Tropical Latin America | Iodine deficiency | 1990 | 1.429193 | 2.606608 | 0.719767 |
| DALYs | Tropical Latin America | Iodine deficiency | 1991 | 1.428304 | 2.647214 | 0.716586 |
| DALYs | Tropical Latin America | Iodine deficiency | 1992 | 1.425733 | 2.624444 | 0.70805 |
| DALYs | Tropical Latin America | Iodine deficiency | 1993 | 1.42135 | 2.615103 | 0.704333 |
| DALYs | Tropical Latin America | Iodine deficiency | 1994 | 1.417785 | 2.632891 | 0.697356 |
| DALYs | Tropical Latin America | Iodine deficiency | 1995 | 1.412474 | 2.593324 | 0.703395 |
| DALYs | Tropical Latin America | Iodine deficiency | 1996 | 1.393586 | 2.593073 | 0.686442 |
| DALYs | Tropical Latin America | Iodine deficiency | 1997 | 1.354423 | 2.526161 | 0.645744 |
| DALYs | Tropical Latin America | Iodine deficiency | 1998 | 1.308981 | 2.478597 | 0.616775 |
| DALYs | Tropical Latin America | Iodine deficiency | 1999 | 1.270021 | 2.411147 | 0.585378 |
| DALYs | Tropical Latin America | Iodine deficiency | 2000 | 1.249894 | 2.370831 | 0.571334 |
| DALYs | Tropical Latin America | Iodine deficiency | 2001 | 1.243101 | 2.348229 | 0.57205 |
| DALYs | Tropical Latin America | Iodine deficiency | 2002 | 1.235486 | 2.353082 | 0.577542 |
| DALYs | Tropical Latin America | Iodine deficiency | 2003 | 1.227075 | 2.31382 | 0.5667 |
| DALYs | Tropical Latin America | Iodine deficiency | 2004 | 1.219003 | 2.319649 | 0.562441 |
| DALYs | Tropical Latin America | Iodine deficiency | 2005 | 1.211531 | 2.302436 | 0.559999 |
| DALYs | Tropical Latin America | Iodine deficiency | 2006 | 1.20194 | 2.276335 | 0.557794 |
| DALYs | Tropical Latin America | Iodine deficiency | 2007 | 1.185026 | 2.243216 | 0.540157 |
| DALYs | Tropical Latin America | Iodine deficiency | 2008 | 1.167389 | 2.209228 | 0.529249 |
| DALYs | Tropical Latin America | Iodine deficiency | 2009 | 1.152226 | 2.209643 | 0.51763 |
| DALYs | Tropical Latin America | Iodine deficiency | 2010 | 1.142886 | 2.20277 | 0.509851 |
| DALYs | Tropical Latin America | Iodine deficiency | 2011 | 1.137267 | 2.200466 | 0.500492 |
| DALYs | Tropical Latin America | Iodine deficiency | 2012 | 1.130499 | 2.170992 | 0.50263 |
| DALYs | Tropical Latin America | Iodine deficiency | 2013 | 1.124999 | 2.157267 | 0.498218 |
| DALYs | Tropical Latin America | Iodine deficiency | 2014 | 1.119692 | 2.162733 | 0.49527 |
| DALYs | Tropical Latin America | Iodine deficiency | 2015 | 1.116346 | 2.160685 | 0.496429 |
| DALYs | Tropical Latin America | Iodine deficiency | 2016 | 1.1193 | 2.166814 | 0.501238 |
| DALYs | Tropical Latin America | Iodine deficiency | 2017 | 1.121355 | 2.170352 | 0.499849 |
| DALYs | Tropical Latin America | Iodine deficiency | 2018 | 1.120453 | 2.166564 | 0.499026 |
| DALYs | Tropical Latin America | Iodine deficiency | 2019 | 1.11944 | 2.149418 | 0.500467 |
| DALYs | Tropical Latin America | Vitamin A deficiency | 1990 | 22.99453 | 34.00787 | 14.52315 |
| DALYs | Tropical Latin America | Vitamin A deficiency | 1991 | 22.103 | 32.37725 | 13.99326 |
| DALYs | Tropical Latin America | Vitamin A deficiency | 1992 | 21.28579 | 31.06898 | 13.63406 |
| DALYs | Tropical Latin America | Vitamin A deficiency | 1993 | 20.54675 | 29.87334 | 13.01403 |
| DALYs | Tropical Latin America | Vitamin A deficiency | 1994 | 19.90722 | 29.31923 | 12.64841 |
| DALYs | Tropical Latin America | Vitamin A deficiency | 1995 | 19.3784 | 28.28557 | 12.34318 |
| DALYs | Tropical Latin America | Vitamin A deficiency | 1996 | 19.01993 | 27.65468 | 12.18565 |
| DALYs | Tropical Latin America | Vitamin A deficiency | 1997 | 18.80092 | 27.56525 | 12.07377 |
| DALYs | Tropical Latin America | Vitamin A deficiency | 1998 | 18.63869 | 27.00424 | 12.01794 |
| DALYs | Tropical Latin America | Vitamin A deficiency | 1999 | 18.43643 | 26.56946 | 11.78112 |
| DALYs | Tropical Latin America | Vitamin A deficiency | 2000 | 18.11038 | 26.09913 | 11.65598 |
| DALYs | Tropical Latin America | Vitamin A deficiency | 2001 | 17.60953 | 25.0659 | 11.33447 |
| DALYs | Tropical Latin America | Vitamin A deficiency | 2002 | 17.00185 | 24.34092 | 11.02212 |
| DALYs | Tropical Latin America | Vitamin A deficiency | 2003 | 16.34785 | 23.343 | 10.56313 |
| DALYs | Tropical Latin America | Vitamin A deficiency | 2004 | 15.70689 | 22.72032 | 10.08213 |
| DALYs | Tropical Latin America | Vitamin A deficiency | 2005 | 15.15662 | 22.05388 | 9.714759 |
| DALYs | Tropical Latin America | Vitamin A deficiency | 2006 | 14.67535 | 21.35594 | 9.439463 |
| DALYs | Tropical Latin America | Vitamin A deficiency | 2007 | 14.19953 | 20.74142 | 9.078107 |
| DALYs | Tropical Latin America | Vitamin A deficiency | 2008 | 13.74069 | 20.03281 | 8.803545 |
| DALYs | Tropical Latin America | Vitamin A deficiency | 2009 | 13.31585 | 19.5826 | 8.581332 |
| DALYs | Tropical Latin America | Vitamin A deficiency | 2010 | 12.91912 | 18.96373 | 8.225863 |
| DALYs | Tropical Latin America | Vitamin A deficiency | 2011 | 12.54965 | 18.79959 | 8.00855 |
| DALYs | Tropical Latin America | Vitamin A deficiency | 2012 | 12.19456 | 18.03665 | 7.671316 |
| DALYs | Tropical Latin America | Vitamin A deficiency | 2013 | 11.85683 | 17.77309 | 7.503798 |
| DALYs | Tropical Latin America | Vitamin A deficiency | 2014 | 11.55225 | 17.22054 | 7.33296 |
| DALYs | Tropical Latin America | Vitamin A deficiency | 2015 | 11.28188 | 16.67908 | 7.091233 |
| DALYs | Tropical Latin America | Vitamin A deficiency | 2016 | 11.06243 | 16.37266 | 6.859008 |
| DALYs | Tropical Latin America | Vitamin A deficiency | 2017 | 10.8502 | 16.16203 | 6.688174 |
| DALYs | Tropical Latin America | Vitamin A deficiency | 2018 | 10.55069 | 15.62939 | 6.465095 |
| DALYs | Tropical Latin America | Vitamin A deficiency | 2019 | 10.17052 | 15.05081 | 6.224623 |
| DALYs | Western Sub-Saharan Africa | Dietary iron deficiency | 1990 | 579.7259 | 853.955 | 386.7009 |
| DALYs | Western Sub-Saharan Africa | Dietary iron deficiency | 1991 | 581.9244 | 859.1376 | 388.715 |
| DALYs | Western Sub-Saharan Africa | Dietary iron deficiency | 1992 | 584.0346 | 861.697 | 390.0182 |
| DALYs | Western Sub-Saharan Africa | Dietary iron deficiency | 1993 | 586.0333 | 861.5967 | 390.2614 |
| DALYs | Western Sub-Saharan Africa | Dietary iron deficiency | 1994 | 587.9707 | 861.1589 | 393.6131 |
| DALYs | Western Sub-Saharan Africa | Dietary iron deficiency | 1995 | 589.2906 | 868.8131 | 394.494 |
| DALYs | Western Sub-Saharan Africa | Dietary iron deficiency | 1996 | 590.7126 | 871.4307 | 396.2734 |
| DALYs | Western Sub-Saharan Africa | Dietary iron deficiency | 1997 | 592.2731 | 873.1556 | 396.5378 |
| DALYs | Western Sub-Saharan Africa | Dietary iron deficiency | 1998 | 593.63 | 875.0458 | 396.1659 |
| DALYs | Western Sub-Saharan Africa | Dietary iron deficiency | 1999 | 594.5774 | 877.7239 | 395.9968 |
| DALYs | Western Sub-Saharan Africa | Dietary iron deficiency | 2000 | 595.1738 | 879.8122 | 395.8611 |
| DALYs | Western Sub-Saharan Africa | Dietary iron deficiency | 2001 | 594.5815 | 874.4951 | 399.0924 |
| DALYs | Western Sub-Saharan Africa | Dietary iron deficiency | 2002 | 593.106 | 867.235 | 398.2316 |
| DALYs | Western Sub-Saharan Africa | Dietary iron deficiency | 2003 | 591.2136 | 865.1506 | 399.6377 |
| DALYs | Western Sub-Saharan Africa | Dietary iron deficiency | 2004 | 589.317 | 858.8659 | 399.5383 |
| DALYs | Western Sub-Saharan Africa | Dietary iron deficiency | 2005 | 587.9997 | 851.1949 | 397.2254 |
| DALYs | Western Sub-Saharan Africa | Dietary iron deficiency | 2006 | 587.1824 | 845.9485 | 397.3361 |
| DALYs | Western Sub-Saharan Africa | Dietary iron deficiency | 2007 | 586.6034 | 847.9409 | 396.9855 |
| DALYs | Western Sub-Saharan Africa | Dietary iron deficiency | 2008 | 586.6422 | 851.3412 | 395.5233 |
| DALYs | Western Sub-Saharan Africa | Dietary iron deficiency | 2009 | 587.4433 | 855.6787 | 391.6992 |
| DALYs | Western Sub-Saharan Africa | Dietary iron deficiency | 2010 | 589.2024 | 860.878 | 390.9868 |
| DALYs | Western Sub-Saharan Africa | Dietary iron deficiency | 2011 | 594.5552 | 865.1202 | 395.8933 |
| DALYs | Western Sub-Saharan Africa | Dietary iron deficiency | 2012 | 604.0404 | 879.2858 | 403.0137 |
| DALYs | Western Sub-Saharan Africa | Dietary iron deficiency | 2013 | 615.0277 | 897.8745 | 411.5079 |
| DALYs | Western Sub-Saharan Africa | Dietary iron deficiency | 2014 | 624.8826 | 913.4914 | 415.424 |
| DALYs | Western Sub-Saharan Africa | Dietary iron deficiency | 2015 | 631.0633 | 922.3546 | 420.2939 |
| DALYs | Western Sub-Saharan Africa | Dietary iron deficiency | 2016 | 635.406 | 930.5049 | 426.2329 |
| DALYs | Western Sub-Saharan Africa | Dietary iron deficiency | 2017 | 636.3773 | 930.4942 | 425.009 |
| DALYs | Western Sub-Saharan Africa | Dietary iron deficiency | 2018 | 631.6519 | 919.2498 | 423.1402 |
| DALYs | Western Sub-Saharan Africa | Dietary iron deficiency | 2019 | 624.0087 | 905.286 | 415.3169 |
| DALYs | Western Sub-Saharan Africa | Iodine deficiency | 1990 | 33.31903 | 56.22404 | 19.16431 |
| DALYs | Western Sub-Saharan Africa | Iodine deficiency | 1991 | 35.11068 | 58.94532 | 20.37518 |
| DALYs | Western Sub-Saharan Africa | Iodine deficiency | 1992 | 36.41899 | 61.19943 | 21.0379 |
| DALYs | Western Sub-Saharan Africa | Iodine deficiency | 1993 | 37.32218 | 62.31494 | 21.72364 |
| DALYs | Western Sub-Saharan Africa | Iodine deficiency | 1994 | 37.85363 | 63.39651 | 22.02271 |
| DALYs | Western Sub-Saharan Africa | Iodine deficiency | 1995 | 37.97683 | 63.49505 | 21.88026 |
| DALYs | Western Sub-Saharan Africa | Iodine deficiency | 1996 | 37.3552 | 62.42972 | 21.54874 |
| DALYs | Western Sub-Saharan Africa | Iodine deficiency | 1997 | 35.95759 | 60.14477 | 20.89978 |
| DALYs | Western Sub-Saharan Africa | Iodine deficiency | 1998 | 34.21651 | 57.06966 | 19.95398 |
| DALYs | Western Sub-Saharan Africa | Iodine deficiency | 1999 | 32.62669 | 54.57047 | 18.96728 |
| DALYs | Western Sub-Saharan Africa | Iodine deficiency | 2000 | 31.69918 | 52.55988 | 18.55687 |
| DALYs | Western Sub-Saharan Africa | Iodine deficiency | 2001 | 31.06202 | 51.61788 | 18.42538 |
| DALYs | Western Sub-Saharan Africa | Iodine deficiency | 2002 | 30.10843 | 50.30449 | 17.73286 |
| DALYs | Western Sub-Saharan Africa | Iodine deficiency | 2003 | 29.02442 | 48.62133 | 16.95137 |
| DALYs | Western Sub-Saharan Africa | Iodine deficiency | 2004 | 28.00266 | 47.10892 | 16.02083 |
| DALYs | Western Sub-Saharan Africa | Iodine deficiency | 2005 | 27.26114 | 46.09241 | 15.88108 |
| DALYs | Western Sub-Saharan Africa | Iodine deficiency | 2006 | 26.59714 | 44.70016 | 15.49957 |
| DALYs | Western Sub-Saharan Africa | Iodine deficiency | 2007 | 25.74639 | 43.51847 | 14.86561 |
| DALYs | Western Sub-Saharan Africa | Iodine deficiency | 2008 | 24.85281 | 42.24629 | 14.14045 |
| DALYs | Western Sub-Saharan Africa | Iodine deficiency | 2009 | 24.03645 | 40.98875 | 13.72506 |
| DALYs | Western Sub-Saharan Africa | Iodine deficiency | 2010 | 23.44535 | 40.35293 | 13.23002 |
| DALYs | Western Sub-Saharan Africa | Iodine deficiency | 2011 | 22.94915 | 39.67447 | 13.12234 |
| DALYs | Western Sub-Saharan Africa | Iodine deficiency | 2012 | 22.3487 | 38.79175 | 12.71424 |
| DALYs | Western Sub-Saharan Africa | Iodine deficiency | 2013 | 21.70493 | 37.82159 | 12.32267 |
| DALYs | Western Sub-Saharan Africa | Iodine deficiency | 2014 | 21.1248 | 36.82756 | 11.8732 |
| DALYs | Western Sub-Saharan Africa | Iodine deficiency | 2015 | 20.69368 | 36.09301 | 11.73009 |
| DALYs | Western Sub-Saharan Africa | Iodine deficiency | 2016 | 20.34906 | 35.67158 | 11.34102 |
| DALYs | Western Sub-Saharan Africa | Iodine deficiency | 2017 | 20.06549 | 35.12917 | 11.23509 |
| DALYs | Western Sub-Saharan Africa | Iodine deficiency | 2018 | 19.87525 | 34.79394 | 11.15571 |
| DALYs | Western Sub-Saharan Africa | Iodine deficiency | 2019 | 19.7108 | 34.52655 | 11.08249 |
| DALYs | Western Sub-Saharan Africa | Vitamin A deficiency | 1990 | 74.44744 | 104.174 | 50.41043 |
| DALYs | Western Sub-Saharan Africa | Vitamin A deficiency | 1991 | 72.8948 | 101.9246 | 49.45084 |
| DALYs | Western Sub-Saharan Africa | Vitamin A deficiency | 1992 | 71.37484 | 99.66691 | 48.5098 |
| DALYs | Western Sub-Saharan Africa | Vitamin A deficiency | 1993 | 69.926 | 97.82228 | 47.58588 |
| DALYs | Western Sub-Saharan Africa | Vitamin A deficiency | 1994 | 68.59878 | 95.98313 | 46.92291 |
| DALYs | Western Sub-Saharan Africa | Vitamin A deficiency | 1995 | 67.42225 | 94.18261 | 45.86751 |
| DALYs | Western Sub-Saharan Africa | Vitamin A deficiency | 1996 | 66.28458 | 92.29797 | 45.25674 |
| DALYs | Western Sub-Saharan Africa | Vitamin A deficiency | 1997 | 65.13323 | 90.36062 | 44.31643 |
| DALYs | Western Sub-Saharan Africa | Vitamin A deficiency | 1998 | 64.03311 | 88.88242 | 43.79407 |
| DALYs | Western Sub-Saharan Africa | Vitamin A deficiency | 1999 | 63.01472 | 87.13296 | 43.16755 |
| DALYs | Western Sub-Saharan Africa | Vitamin A deficiency | 2000 | 62.11461 | 86.19909 | 42.61376 |
| DALYs | Western Sub-Saharan Africa | Vitamin A deficiency | 2001 | 61.13567 | 85.18349 | 41.80256 |
| DALYs | Western Sub-Saharan Africa | Vitamin A deficiency | 2002 | 59.93079 | 83.44277 | 40.83944 |
| DALYs | Western Sub-Saharan Africa | Vitamin A deficiency | 2003 | 58.57236 | 81.90313 | 39.87804 |
| DALYs | Western Sub-Saharan Africa | Vitamin A deficiency | 2004 | 57.18642 | 80.01936 | 38.86762 |
| DALYs | Western Sub-Saharan Africa | Vitamin A deficiency | 2005 | 55.87299 | 78.32673 | 38.15898 |
| DALYs | Western Sub-Saharan Africa | Vitamin A deficiency | 2006 | 54.35863 | 76.21515 | 37.28279 |
| DALYs | Western Sub-Saharan Africa | Vitamin A deficiency | 2007 | 52.43393 | 73.34586 | 35.77593 |
| DALYs | Western Sub-Saharan Africa | Vitamin A deficiency | 2008 | 50.3959 | 70.94133 | 34.45382 |
| DALYs | Western Sub-Saharan Africa | Vitamin A deficiency | 2009 | 48.4358 | 68.00089 | 33.19999 |
| DALYs | Western Sub-Saharan Africa | Vitamin A deficiency | 2010 | 46.83372 | 66.12987 | 31.97301 |
| DALYs | Western Sub-Saharan Africa | Vitamin A deficiency | 2011 | 45.48752 | 64.11408 | 31.09604 |
| DALYs | Western Sub-Saharan Africa | Vitamin A deficiency | 2012 | 44.16207 | 62.10221 | 30.21201 |
| DALYs | Western Sub-Saharan Africa | Vitamin A deficiency | 2013 | 42.89681 | 59.97161 | 29.29832 |
| DALYs | Western Sub-Saharan Africa | Vitamin A deficiency | 2014 | 41.68898 | 58.03148 | 28.50666 |
| DALYs | Western Sub-Saharan Africa | Vitamin A deficiency | 2015 | 40.56791 | 56.73128 | 27.76574 |
| DALYs | Western Sub-Saharan Africa | Vitamin A deficiency | 2016 | 39.35588 | 54.9865 | 26.93445 |
| DALYs | Western Sub-Saharan Africa | Vitamin A deficiency | 2017 | 38.28561 | 53.5489 | 26.30111 |
| DALYs | Western Sub-Saharan Africa | Vitamin A deficiency | 2018 | 37.55959 | 52.59527 | 25.73482 |
| DALYs | Western Sub-Saharan Africa | Vitamin A deficiency | 2019 | 37.04333 | 51.75803 | 25.35384 |
| Incidence | Andean Latin America | Dietary iron deficiency | 1990 | No data | No data | No data |
| Incidence | Andean Latin America | Dietary iron deficiency | 1991 | No data | No data | No data |
| Incidence | Andean Latin America | Dietary iron deficiency | 1992 | No data | No data | No data |
| Incidence | Andean Latin America | Dietary iron deficiency | 1993 | No data | No data | No data |
| Incidence | Andean Latin America | Dietary iron deficiency | 1994 | No data | No data | No data |
| Incidence | Andean Latin America | Dietary iron deficiency | 1995 | No data | No data | No data |
| Incidence | Andean Latin America | Dietary iron deficiency | 1996 | No data | No data | No data |
| Incidence | Andean Latin America | Dietary iron deficiency | 1997 | No data | No data | No data |
| Incidence | Andean Latin America | Dietary iron deficiency | 1998 | No data | No data | No data |
| Incidence | Andean Latin America | Dietary iron deficiency | 1999 | No data | No data | No data |
| Incidence | Andean Latin America | Dietary iron deficiency | 2000 | No data | No data | No data |
| Incidence | Andean Latin America | Dietary iron deficiency | 2001 | No data | No data | No data |
| Incidence | Andean Latin America | Dietary iron deficiency | 2002 | No data | No data | No data |
| Incidence | Andean Latin America | Dietary iron deficiency | 2003 | No data | No data | No data |
| Incidence | Andean Latin America | Dietary iron deficiency | 2004 | No data | No data | No data |
| Incidence | Andean Latin America | Dietary iron deficiency | 2005 | No data | No data | No data |
| Incidence | Andean Latin America | Dietary iron deficiency | 2006 | No data | No data | No data |
| Incidence | Andean Latin America | Dietary iron deficiency | 2007 | No data | No data | No data |
| Incidence | Andean Latin America | Dietary iron deficiency | 2008 | No data | No data | No data |
| Incidence | Andean Latin America | Dietary iron deficiency | 2009 | No data | No data | No data |
| Incidence | Andean Latin America | Dietary iron deficiency | 2010 | No data | No data | No data |
| Incidence | Andean Latin America | Dietary iron deficiency | 2011 | No data | No data | No data |
| Incidence | Andean Latin America | Dietary iron deficiency | 2012 | No data | No data | No data |
| Incidence | Andean Latin America | Dietary iron deficiency | 2013 | No data | No data | No data |
| Incidence | Andean Latin America | Dietary iron deficiency | 2014 | No data | No data | No data |
| Incidence | Andean Latin America | Dietary iron deficiency | 2015 | No data | No data | No data |
| Incidence | Andean Latin America | Dietary iron deficiency | 2016 | No data | No data | No data |
| Incidence | Andean Latin America | Dietary iron deficiency | 2017 | No data | No data | No data |
| Incidence | Andean Latin America | Dietary iron deficiency | 2018 | No data | No data | No data |
| Incidence | Andean Latin America | Dietary iron deficiency | 2019 | No data | No data | No data |
| Incidence | Andean Latin America | Iodine deficiency | 1990 | 5.555107 | 7.109236 | 4.243203 |
| Incidence | Andean Latin America | Iodine deficiency | 1991 | 5.579457 | 7.154393 | 4.24687 |
| Incidence | Andean Latin America | Iodine deficiency | 1992 | 5.596793 | 7.198959 | 4.256806 |
| Incidence | Andean Latin America | Iodine deficiency | 1993 | 5.607646 | 7.222236 | 4.251672 |
| Incidence | Andean Latin America | Iodine deficiency | 1994 | 5.612543 | 7.235224 | 4.249668 |
| Incidence | Andean Latin America | Iodine deficiency | 1995 | 5.612 | 7.23975 | 4.250858 |
| Incidence | Andean Latin America | Iodine deficiency | 1996 | 5.599127 | 7.219867 | 4.242287 |
| Incidence | Andean Latin America | Iodine deficiency | 1997 | 5.572049 | 7.17914 | 4.227423 |
| Incidence | Andean Latin America | Iodine deficiency | 1998 | 5.538928 | 7.122936 | 4.214142 |
| Incidence | Andean Latin America | Iodine deficiency | 1999 | 5.507966 | 7.085997 | 4.185459 |
| Incidence | Andean Latin America | Iodine deficiency | 2000 | 5.487307 | 7.069427 | 4.172266 |
| Incidence | Andean Latin America | Iodine deficiency | 2001 | 5.475218 | 7.049178 | 4.14365 |
| Incidence | Andean Latin America | Iodine deficiency | 2002 | 5.463678 | 7.04366 | 4.126495 |
| Incidence | Andean Latin America | Iodine deficiency | 2003 | 5.451486 | 7.033212 | 4.11094 |
| Incidence | Andean Latin America | Iodine deficiency | 2004 | 5.43748 | 7.03212 | 4.098474 |
| Incidence | Andean Latin America | Iodine deficiency | 2005 | 5.420496 | 7.015167 | 4.075098 |
| Incidence | Andean Latin America | Iodine deficiency | 2006 | 5.390157 | 6.975963 | 4.042513 |
| Incidence | Andean Latin America | Iodine deficiency | 2007 | 5.341935 | 6.923292 | 4.013269 |
| Incidence | Andean Latin America | Iodine deficiency | 2008 | 5.283486 | 6.818433 | 3.964867 |
| Incidence | Andean Latin America | Iodine deficiency | 2009 | 5.222413 | 6.722777 | 3.914115 |
| Incidence | Andean Latin America | Iodine deficiency | 2010 | 5.166242 | 6.663933 | 3.867263 |
| Incidence | Andean Latin America | Iodine deficiency | 2011 | 5.106664 | 6.585861 | 3.813341 |
| Incidence | Andean Latin America | Iodine deficiency | 2012 | 5.037042 | 6.494482 | 3.752665 |
| Incidence | Andean Latin America | Iodine deficiency | 2013 | 4.967359 | 6.419376 | 3.70186 |
| Incidence | Andean Latin America | Iodine deficiency | 2014 | 4.908066 | 6.346299 | 3.669643 |
| Incidence | Andean Latin America | Iodine deficiency | 2015 | 4.869349 | 6.28625 | 3.640657 |
| Incidence | Andean Latin America | Iodine deficiency | 2016 | 4.850778 | 6.26586 | 3.658127 |
| Incidence | Andean Latin America | Iodine deficiency | 2017 | 4.838112 | 6.250003 | 3.679418 |
| Incidence | Andean Latin America | Iodine deficiency | 2018 | 4.817636 | 6.245927 | 3.634301 |
| Incidence | Andean Latin America | Iodine deficiency | 2019 | 4.787616 | 6.230483 | 3.594987 |
| Incidence | Andean Latin America | Vitamin A deficiency | 1990 | 12877.69 | 14260.37 | 11631.86 |
| Incidence | Andean Latin America | Vitamin A deficiency | 1991 | 12534.17 | 13858.39 | 11307.42 |
| Incidence | Andean Latin America | Vitamin A deficiency | 1992 | 12231.14 | 13463.68 | 11053.99 |
| Incidence | Andean Latin America | Vitamin A deficiency | 1993 | 12310.84 | 13498.58 | 11186.93 |
| Incidence | Andean Latin America | Vitamin A deficiency | 1994 | 12200.53 | 13364.05 | 11106.49 |
| Incidence | Andean Latin America | Vitamin A deficiency | 1995 | 12037.04 | 13172.19 | 10973.17 |
| Incidence | Andean Latin America | Vitamin A deficiency | 1996 | 11940.37 | 13092.38 | 10912.67 |
| Incidence | Andean Latin America | Vitamin A deficiency | 1997 | 11552.52 | 12654.48 | 10559.88 |
| Incidence | Andean Latin America | Vitamin A deficiency | 1998 | 11429.25 | 12516.33 | 10464.74 |
| Incidence | Andean Latin America | Vitamin A deficiency | 1999 | 11467.64 | 12594.76 | 10497.52 |
| Incidence | Andean Latin America | Vitamin A deficiency | 2000 | 11202.82 | 12302.36 | 10254.8 |
| Incidence | Andean Latin America | Vitamin A deficiency | 2001 | 10925.39 | 11973.15 | 9970.754 |
| Incidence | Andean Latin America | Vitamin A deficiency | 2002 | 10467.82 | 11457.11 | 9539.314 |
| Incidence | Andean Latin America | Vitamin A deficiency | 2003 | 10047.41 | 11000.18 | 9105.501 |
| Incidence | Andean Latin America | Vitamin A deficiency | 2004 | 9643.909 | 10615.67 | 8702.907 |
| Incidence | Andean Latin America | Vitamin A deficiency | 2005 | 9336.425 | 10286.62 | 8411.9 |
| Incidence | Andean Latin America | Vitamin A deficiency | 2006 | 9029.242 | 9937.412 | 8134.746 |
| Incidence | Andean Latin America | Vitamin A deficiency | 2007 | 8688.482 | 9596.927 | 7793.689 |
| Incidence | Andean Latin America | Vitamin A deficiency | 2008 | 8460.429 | 9372.862 | 7616.259 |
| Incidence | Andean Latin America | Vitamin A deficiency | 2009 | 8315.088 | 9254.48 | 7466.304 |
| Incidence | Andean Latin America | Vitamin A deficiency | 2010 | 8089.557 | 9034.122 | 7256.02 |
| Incidence | Andean Latin America | Vitamin A deficiency | 2011 | 7711.509 | 8591.394 | 6904.446 |
| Incidence | Andean Latin America | Vitamin A deficiency | 2012 | 7398.061 | 8269.884 | 6641.317 |
| Incidence | Andean Latin America | Vitamin A deficiency | 2013 | 7174.193 | 8021.518 | 6422.28 |
| Incidence | Andean Latin America | Vitamin A deficiency | 2014 | 7168.565 | 8001.396 | 6432.497 |
| Incidence | Andean Latin America | Vitamin A deficiency | 2015 | 6900.405 | 7707.501 | 6191.656 |
| Incidence | Andean Latin America | Vitamin A deficiency | 2016 | 6472.752 | 7247.491 | 5780.88 |
| Incidence | Andean Latin America | Vitamin A deficiency | 2017 | 6277.833 | 7041.24 | 5608.282 |
| Incidence | Andean Latin America | Vitamin A deficiency | 2018 | 6071.865 | 6809.143 | 5406.45 |
| Incidence | Andean Latin America | Vitamin A deficiency | 2019 | 5904.692 | 6633.166 | 5259.364 |
| Incidence | Australasia | Dietary iron deficiency | 1990 | No data | No data | No data |
| Incidence | Australasia | Dietary iron deficiency | 1991 | No data | No data | No data |
| Incidence | Australasia | Dietary iron deficiency | 1992 | No data | No data | No data |
| Incidence | Australasia | Dietary iron deficiency | 1993 | No data | No data | No data |
| Incidence | Australasia | Dietary iron deficiency | 1994 | No data | No data | No data |
| Incidence | Australasia | Dietary iron deficiency | 1995 | No data | No data | No data |
| Incidence | Australasia | Dietary iron deficiency | 1996 | No data | No data | No data |
| Incidence | Australasia | Dietary iron deficiency | 1997 | No data | No data | No data |
| Incidence | Australasia | Dietary iron deficiency | 1998 | No data | No data | No data |
| Incidence | Australasia | Dietary iron deficiency | 1999 | No data | No data | No data |
| Incidence | Australasia | Dietary iron deficiency | 2000 | No data | No data | No data |
| Incidence | Australasia | Dietary iron deficiency | 2001 | No data | No data | No data |
| Incidence | Australasia | Dietary iron deficiency | 2002 | No data | No data | No data |
| Incidence | Australasia | Dietary iron deficiency | 2003 | No data | No data | No data |
| Incidence | Australasia | Dietary iron deficiency | 2004 | No data | No data | No data |
| Incidence | Australasia | Dietary iron deficiency | 2005 | No data | No data | No data |
| Incidence | Australasia | Dietary iron deficiency | 2006 | No data | No data | No data |
| Incidence | Australasia | Dietary iron deficiency | 2007 | No data | No data | No data |
| Incidence | Australasia | Dietary iron deficiency | 2008 | No data | No data | No data |
| Incidence | Australasia | Dietary iron deficiency | 2009 | No data | No data | No data |
| Incidence | Australasia | Dietary iron deficiency | 2010 | No data | No data | No data |
| Incidence | Australasia | Dietary iron deficiency | 2011 | No data | No data | No data |
| Incidence | Australasia | Dietary iron deficiency | 2012 | No data | No data | No data |
| Incidence | Australasia | Dietary iron deficiency | 2013 | No data | No data | No data |
| Incidence | Australasia | Dietary iron deficiency | 2014 | No data | No data | No data |
| Incidence | Australasia | Dietary iron deficiency | 2015 | No data | No data | No data |
| Incidence | Australasia | Dietary iron deficiency | 2016 | No data | No data | No data |
| Incidence | Australasia | Dietary iron deficiency | 2017 | No data | No data | No data |
| Incidence | Australasia | Dietary iron deficiency | 2018 | No data | No data | No data |
| Incidence | Australasia | Dietary iron deficiency | 2019 | No data | No data | No data |
| Incidence | Australasia | Iodine deficiency | 1990 | 13.1484 | 16.5989 | 10.22664 |
| Incidence | Australasia | Iodine deficiency | 1991 | 13.1303 | 16.52549 | 10.22584 |
| Incidence | Australasia | Iodine deficiency | 1992 | 13.11225 | 16.521 | 10.25856 |
| Incidence | Australasia | Iodine deficiency | 1993 | 13.09562 | 16.5236 | 10.20942 |
| Incidence | Australasia | Iodine deficiency | 1994 | 13.08185 | 16.49381 | 10.20811 |
| Incidence | Australasia | Iodine deficiency | 1995 | 13.07223 | 16.46385 | 10.21913 |
| Incidence | Australasia | Iodine deficiency | 1996 | 13.06071 | 16.45522 | 10.23355 |
| Incidence | Australasia | Iodine deficiency | 1997 | 13.04165 | 16.36188 | 10.23192 |
| Incidence | Australasia | Iodine deficiency | 1998 | 13.01841 | 16.31644 | 10.18327 |
| Incidence | Australasia | Iodine deficiency | 1999 | 12.99425 | 16.25341 | 10.16837 |
| Incidence | Australasia | Iodine deficiency | 2000 | 12.97254 | 16.3005 | 10.15721 |
| Incidence | Australasia | Iodine deficiency | 2001 | 12.94197 | 16.23587 | 10.10206 |
| Incidence | Australasia | Iodine deficiency | 2002 | 12.89577 | 16.16325 | 10.00247 |
| Incidence | Australasia | Iodine deficiency | 2003 | 12.84406 | 16.1011 | 9.984082 |
| Incidence | Australasia | Iodine deficiency | 2004 | 12.79702 | 16.08671 | 9.953424 |
| Incidence | Australasia | Iodine deficiency | 2005 | 12.76475 | 16.09236 | 9.858111 |
| Incidence | Australasia | Iodine deficiency | 2006 | 12.73786 | 16.03096 | 9.884834 |
| Incidence | Australasia | Iodine deficiency | 2007 | 12.70386 | 15.97574 | 9.842075 |
| Incidence | Australasia | Iodine deficiency | 2008 | 12.66811 | 15.90085 | 9.855745 |
| Incidence | Australasia | Iodine deficiency | 2009 | 12.63604 | 15.90292 | 9.826447 |
| Incidence | Australasia | Iodine deficiency | 2010 | 12.61292 | 15.87492 | 9.80766 |
| Incidence | Australasia | Iodine deficiency | 2011 | 12.58862 | 15.84757 | 9.778577 |
| Incidence | Australasia | Iodine deficiency | 2012 | 12.55456 | 15.83126 | 9.748433 |
| Incidence | Australasia | Iodine deficiency | 2013 | 12.51789 | 15.75177 | 9.717953 |
| Incidence | Australasia | Iodine deficiency | 2014 | 12.48591 | 15.70444 | 9.702655 |
| Incidence | Australasia | Iodine deficiency | 2015 | 12.46567 | 15.7387 | 9.675693 |
| Incidence | Australasia | Iodine deficiency | 2016 | 12.45492 | 15.62623 | 9.711868 |
| Incidence | Australasia | Iodine deficiency | 2017 | 12.44603 | 15.664 | 9.699966 |
| Incidence | Australasia | Iodine deficiency | 2018 | 12.43295 | 15.56219 | 9.714172 |
| Incidence | Australasia | Iodine deficiency | 2019 | 12.4139 | 15.49886 | 9.648999 |
| Incidence | Australasia | Vitamin A deficiency | 1990 | 237.4131 | 270.0315 | 210.3835 |
| Incidence | Australasia | Vitamin A deficiency | 1991 | 229.7535 | 261.627 | 204.5252 |
| Incidence | Australasia | Vitamin A deficiency | 1992 | 222.9731 | 253.2891 | 198.6064 |
| Incidence | Australasia | Vitamin A deficiency | 1993 | 210.3583 | 237.9614 | 187.3938 |
| Incidence | Australasia | Vitamin A deficiency | 1994 | 199.3927 | 225.5696 | 177.0366 |
| Incidence | Australasia | Vitamin A deficiency | 1995 | 190.3252 | 214.8742 | 168.5669 |
| Incidence | Australasia | Vitamin A deficiency | 1996 | 179.7668 | 203.6679 | 159.3727 |
[truncated: 460,570 more chars]
